# Checklist for the insect fauna of two East Sea Islands (Ulleungdo Is. and Dokdo Is.) in the Republic of Korea

**DOI:** 10.3897/BDJ.12.e129360

**Published:** 2024-12-19

**Authors:** Dong-Yeol Lee, Ilyeong Jeong, Seonmin Kim, Jae Won Choi, Min Hyeok Won, Donguk Kim, Dongmin Kim, Young-Kun Kim, Jiseung Jeon, Jihun Ryu, WooJun Bang, Jun Hyuk Chang, Kwang Shik Choi

**Affiliations:** 1 Department of Biology, College of Natural Sciences, Kyungpook National University, Daegu, Republic of Korea Department of Biology, College of Natural Sciences, Kyungpook National University Daegu Republic of Korea; 2 Research Institute for Dok-do and Ulleungdo Island, Kyungpook National University, Daegu, Republic of Korea Research Institute for Dok-do and Ulleungdo Island, Kyungpook National University Daegu Republic of Korea; 3 National Institute of Ecology, Seocheon, Chungcheongnam-do, Republic of Korea National Institute of Ecology Seocheon, Chungcheongnam-do Republic of Korea; 4 Wetland Research Team, Wetland Center, National Institute of Ecology, Changnyeong, Gyeongsangnam-do, Republic of Korea Wetland Research Team, Wetland Center, National Institute of Ecology Changnyeong, Gyeongsangnam-do Republic of Korea; 5 School of Life Sciences, BK21 FOUR KNU Creative BioResearch Group, Kyungpook National University, Daegu, Republic of Korea School of Life Sciences, BK21 FOUR KNU Creative BioResearch Group, Kyungpook National University Daegu Republic of Korea; 6 Department of Applied Biology, Kyungpook National University, Daegu, Republic of Korea Department of Applied Biology, Kyungpook National University Daegu Republic of Korea; 7 School of Applied Biosciences, Kyungpook National University, Daegu, Republic of Korea School of Applied Biosciences, Kyungpook National University Daegu Republic of Korea; 8 School of Biological Sciences, Seoul National University, Seoul, Republic of Korea School of Biological Sciences, Seoul National University Seoul Republic of Korea; 9 Institute for Phylogenomics and Evolution, Kyungpook National University, Daegu, Republic of Korea Institute for Phylogenomics and Evolution, Kyungpook National University Daegu Republic of Korea

**Keywords:** Ulleungdo Island, Dokdo Island, East Sea, insect fauna, species diversity

## Abstract

**Background:**

Ulleungdo and Dokdo, located in the East Sea, are volcanic islands with high ecological value due to their unique biodiversity. Although research on the insect fauna on these two Islands has been conducted from the early 19^th^ century to recent times, limitations exist due to several issues, including misidentifications and historical errors. This study addresses these issues by conducting a comprehensive insect survey from 2020 to 2023, re-identifying misidentified specimens and compiling references to create an updated and accurate checklist of insect species for Ulleungdo and Dokdo.

**New information:**

The checklists include 20 orders, 227 families and 1694 species, including 28 previously unrecorded species for Ulleungdo Island: *Neoitamuscothurnatusunivittatus* (Loew, 1871), *Elachipterainsignis* (Thomson, 1869), *Elachipterasibirica* (Loew, 1858), *Muscinalevida* (Harris, 1780), *Orchisiacostata* (Meigen, 1826), *Ensinasonchi* (Linnaeus, 1767), *Armigeressubalbatus* (Coquillett, 1898), *Aedeskoreicus* (Edwards, 1917), *Aedesgalloisi* Yamada, 1921, *Aedesalbopictus* (Skuse, 1894), *Aedestogoi* (Theobald, 1907), *Stenolophusdifficilis* (Hope, 1845), *Dasytesvulgaris* Nakane, 1963, *Attaluselongatulus* Lewis, 1895, *Elacatiskraatzi* Reitter, 1879, *Polygraphusjezoensis* Niisima, 1909, *Scolytoplatypustycon* Blandford, 1893, *Ambrosiophilusatratus* (Eichhoff, 1876), *Psilarthroidesczerskyi* (Zaslavskij, 1956), *Tomoxianipponica* Kôno, 1928, *Adelphocorisdemissus* Horvath, 1905, *Corythuchamarmorata* (Uhler, 1878), *Spilarctiaseriatopunctatasuzukii* Inoue & Maenami, 1963, *Hypopyravespertilio* (Fabricius, 1787), *Simpliciarectalis* (Eversmann, 1842), *Acronictahercules* (Felder & Rogenhofer, 1874), *Analetiapostica* (Hampson, 1905) and *Eudemisbrevisetosa* Oku, 2005; 10 orders, 80 families and 201 species, including three unrecorded species for Dokdo Island: *Arhopaloscelisbifasciata* (Kraatz, 1879), *Nabisstenoferus* Hsiao, 1964 and *Haplogonatopusoratorius* (Westwood, 1833).

## Introduction

Islands are highly valuable biological repositories, with their ecological and evolutionary significance, occupying a pivotal position in the biological understanding of biodiversity and species differentiation (Whittaker et al. 2017). The isolation and unique evolutionary paths of species inhabiting islands lead to high endemism, making islands ecologically and genetically critical hotspots (Adsersen 1995, Eliasson 1995). However, the ecosystems of isolated islands often lack certain major biotic groups compared to continental ecosystems, making them susceptible to external threats such as invasive species (Vitousek 1988). Further, they also tend to have lower biodiversity due to limited area (Mauchamp 2002, Koh and Kil 2006). Moreover, recent advancements in accessibility to islands have reduced their ecological isolation, leading to a degradation in the interplay between evolutionary and ecological dynamics that support island biodiversity (Causton et al. 2006, Chi et al. 2021). Additionally, island ecosystems are currently confronted with multiple threats, including deforestation, resource depletion and global climate change (Kueffer 2010, Bellard et al. 2014, van Kleunen et al. 2015). Therefore, to accurately recognise and respond to these changes in island biodiversity caused by these factors, it is essential to monitor island biodiversity, meticulously assessing the risks of biodiversity loss and ecosystem degradation (Ricketts et al. 2005, Caujapé-Castells et al. 2010). For these reasons, the importance of monitoring island ecosystems is increasingly recognised. Continuous and comprehensive monitoring is essential for understanding the rate and patterns of these ecological changes and can provide crucial information for developing conservation strategies and mitigation measures (Borges et al. 2018).

Ulleungdo and Dokdo, located in the East Sea and formed between 2.5 million and 4.6 million years ago through marine volcanic eruptions, serve as prime examples of the dynamic beauty and ecological significance of oceanic islands (Sohn and Park 1994, Jeon 2005). These Islands were formed by solidifying lava from the seafloor and have never been connected to the mainland, not even during the last maximum glaciation period (LGM) that occurred between 18,000 and 25,000 years ago (Lee and Nam 2003). This isolation has provided a crucial cornerstone for the evolution of species, cultivating a unique ecosystem that has intrigued scientists for decades (Sun et al. 1996, Weigelt et al. 2016).

Ulleungdo, located about 130 km east of mainland Korea, is a volcanic island covering an area of approximately 73 km², featuring the main island with Seonginbong Peak rising to 985 m and several smaller islets (Yoon et al. 2013). A significant portion of the island's forests is designated and managed as a forest genetic resource reserve by the Korea Forest Service, ensuring their protection and preservation (Lee et al. 2007). Additionally, Ulleungdo is geographically located at the confluence of warm and cold ocean currents, exhibiting unique vegetation structures and biogeographical characteristics. Thus, it is recognised as an ecologically significant area, with 31 out of 494 native vascular plant species being endemic, representing a flora endemism rate of 7.9% (Kim et al. 1996, Lee 2008, Andersen 2019, Chung et al. 2020).

In contrast, Dokdo is 216.8 km from the Korean mainland, 87.4 km from Ulleungdo and 157.6 km from Oki Island. Further, it is a volcanic island of a relatively smaller area (187,554 m²) than Ulleungdo Island, comprising two main islands, Dongdo and Seodo, along with 89 smaller islets and rocks (Hwang and Park 2007). Dokdo possesses a highly vulnerable ground environment characterised by severe soil erosion and loss due to strong winds and high precipitation. It also experiences minimal plant settlement and vegetation development due to excessive exposure to direct sunlight, physical disturbance by wind and exposure to high-salt sea breezes (Yu and Song 2006). Due to these characteristics, the growth environment on Dokdo is significantly poorer compared to typical terrestrial environments, making recovery following damage difficult (Park 2014). Therefore, since 1982, the Cultural Heritage Administration of the Republic of Korea has designated Dokdo as a natural monument (No. 336; Article 25) and implemented strict access restrictions to conserve its biodiversity and ecosystem. Thus, the natural resources of Dokdo are preserved from anthropogenic damage due to its geographical location and limited accessibility (Hwang 2023).

Investigations on the insect fauna of Ulleungdo were first conducted by Cho (1929), who documented four families and 16 species of Lepidoptera. Subsequent comprehensive faunistic surveys expanded the knowledge of various taxonomic groups, reporting 10 orders, 58 families and 116 species in 1955 (Cho 1955), 62 families and 137 species in 1965 (Cho 1965), 95 families and 345 species in 1971 (Kim 1971), 125 families and 574 species in 1981 (Lee and Kwon 1981), 131 families and 614 species in 1993 (ME 1993), 141 families and 691 species in 1996 (Kwon et al. 1996), 154 families and 828 species in 2001 (Lee and Jung 2001), 153 families and 819 species in 2006 (Lee et al. 2006), 81 families and 242 species in 2012 (Lim and Lee 2012) and 179 families and 1177 species in 2013 (Lim et al. 2013). In addition, several surveys of specific areas or insect taxa were conducted (Masaki 1934, Seok 1938, Park and Kano 1963, Park et al. 1979, Choi 1986, Byun et al. 1996, Kim 1999, Oh et al. 2013, Park et al. 2014, Nam et al. 2018, Choi et al. 2022, Won et al. 2023).

Since the initial insect survey conducted by Jolivet (1974), the Korean government has periodically monitored Dokdo for various complex reasons, including geopolitical factors. Over approximately 50 years, from 1978 to 2023, many entomologists and national institutions have been mobilised to survey the insect fauna of Dokdo. These extensive research studies have documented a total of 195 species of insects across 74 families and 10 orders (Yoon 1978, Lee and Kwon 1981, An 2000, Lee and Jung 2001, Kim 2004, Park and Suh 2005, Kim and Yeom 2006, An 2008, Lee et al. 2009, Park et al. 2010, Park et al. 2011, Park et al. 2012, Bae 2013, Park et al. 2013, Choi 2015, Park and Jang 2016, Lee 2016, Park et al. 2017, Choi 2017, Hwang et al. 2017, Choi 2018, Ryu et al. 2021, Bang et al. 2022).

The entomological surveys conducted on Ulleungdo and Dokdo to date have established an overall base for understanding the insect fauna of these islands. However, there are limits that require improvement, including taxonomic errors, reference errors and numerous unidentified specimens. In addition, the most recent insect survey conducted on Ulleungdo by Won et al. (2023) was only conducted on a specific area, so there is a limit to showing overall insect diversity. Therefore, this study aims to update the insect database of the two islands by conducting field surveys on Ulleungdo and Dokdo from 2020 to 2023, correcting taxonomic and recording errors and re-identifying existing misidentified samples and historical compilation of vast literature sources.

## Materials and methods

### Sample collection

**Figure 1.**
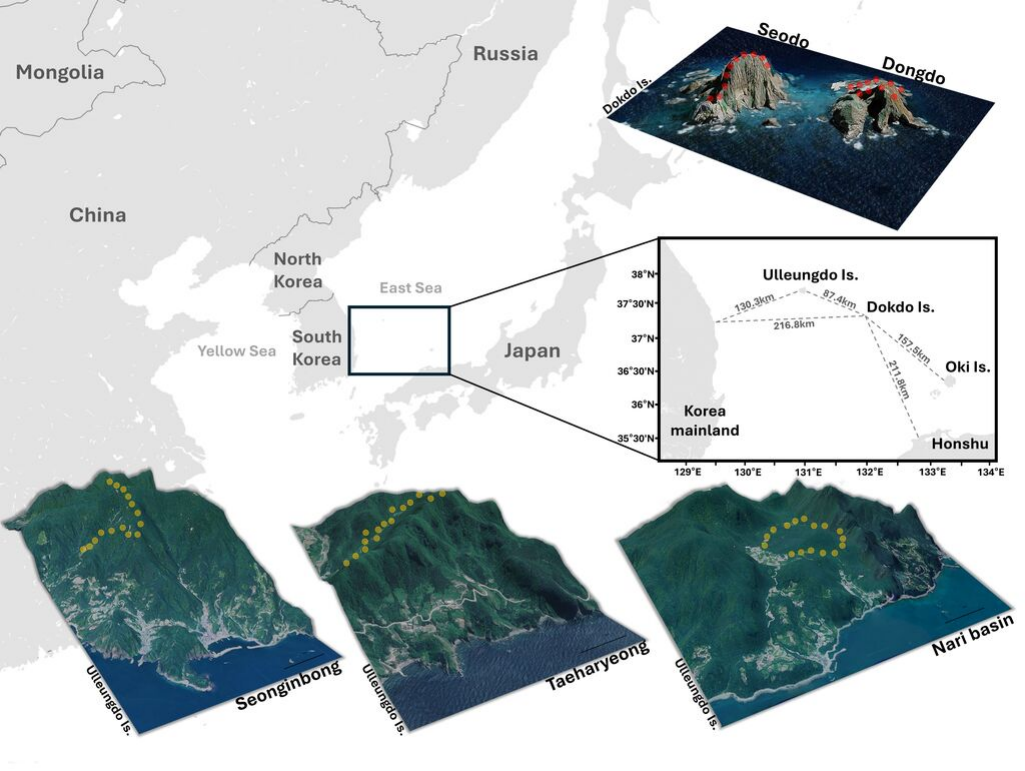
Geographical map of Ulleungdo and Dokdo islands with insets showing collection sites. Insets provide a detailed view of Ulleungdo with Seonginbong, Taeharyeong and Nari Basin, as well as the two main islands (Dongdo and Seodo) of Dokdo. Yellow points are the collection sites established at Ulleungdo and red points are those established at Dokdo.

In previous studies, consistent and intensive sampling using a variety of collection methods across different areas of Ulleungdo had not been conducted. Therefore, in this study, we selected three main areas in Ulleungdo with high ecological value —Seonginbong (37°29'52"N, 130°52'03"E), Taeharyeong (37°29'47"N, 130°50'06"E) and Nari Basin (37°30'55"N, 130°51'35"E) — and identified 15 specific sampling sites within these areas, based on observed ecological variation, such as changes in vegetation and altitude (Fig. 1).

Pitfall traps and molasses traps were set up at all 15 sites. For each pitfall trap, we used a plastic cup (9.5 cm in diameter, 14 cm in height) that was buried level with the ground and filled with an attractant. Three types of attractants — molasses, pork and small octopus — were used, with the molasses being the same as that used in the molasses traps. At each site, four traps per attractant type were installed, totalling 60 traps across the 15 sites and these were left in place for 48 hours. For light traps, we selected three sites with significant ecological variation amongst the 15 sampling sites. Using a portable generator, we set up a mercury lamp emitting ultraviolet light and conducted collection after sunset, allowing 30 minutes per site. Sweeping was conducted using an insect net (pole length: 2.5 m; net diameter: 50 cm; net length: 110 cm). We performed continuous sampling not only at the 15 designated sites, but also along the routes between sites. Collected specimens were temporarily stored in conical tubes using an insect aspirator. In addition, to investigate previously unexplored areas near human settlements, we deployed BG-sentinel traps (Biogents, Regensburg, Germany) and black light traps (BioTrap, Seoul, ROK) around residential areas. These traps were set up for a period of five days to collect specimens from areas that had not been sampled in previous studies. Conversely, due to the terrain’s specificity and conservation concerns on Dokdo, 10 sites were selected at each collection area (Dongdo and Seodo), where only sweeping nets were used for collection. Detailed information on the altitude, coordinates and collection methods used at each sampling site is provided in Tables 1, 2.

### Data collection

For the creation of a species checklist, only species-level data were included through a historical compilation. This comprehensive review covered not only faunistic studies from the early 20^th^ century to the present, but also extensive literature on the Insecta of Ulleungdo and Dokdo (Masaki 1936, Lee 1987, Lyu 2008, Kim et al. 2009, Bae 2010, Bae 2011, Hong et al. 2011, Kim 2011, Park 2011, Schöller 2011, Choi 2012, Hong et al. 2012, Jung 2012a, Jung 2012b, Kim 2012, Lee and Ahn 2012, Cho and Lee 2012, Lee et al. 2012, Park et al. 2012, Bae 2013, Kim and Kim 2013, Park 2013, Yoo et al. 2013, Kang et al. 2013, Bae 2014, Choi 2014, Cho 2014, Han et al. 2014, Kim and Kim 2014, Lee 2014, Park et al. 2014, Lee et al. 2017, Lee et al. 2018, Jung 2018, Lee and Ahn 2018, Lee and Ahn 2019, Cho 2019, Lee et al. 2019, Lee and Choi 2019, Suh 2019, Kwon and Kwon 2020, Kwon et al. 2020, Roca-Cusachs and Jung 2020, NIBR 2021, Sohn et al. 2021, Ryu and Choi 2022, HNIBR 2022, Hwang 2023, Lee et al. 2023, Kim et al. 2023). Additionally, occurrence records and specimen data were utilised alongside data from national institutions of Korea (HNIBR: Honam National Institute of Biological Resources; Info: Korea National Arboretum; ME: Korean Ministry of Environment; NIBR: National Institute of Biological Resources; NSMK: National Science Museum of Korea) previously published in the Global Biodiversity Information Facility (GBIF) (Kwon 2021, Kwon 2022, Info and Park 2023, Info 2023, NSMK 2023b, NSMK 2023a, Choi 2023). Additionally, insect specimens collected from 2014 to 2019 from Ulleungdo and Dokdo and stored at Kyungpook National University (KNU) were identified or re-examined to create the checklist.

## Checklists

### Insect Checklist of Ulleungdo Island

#### Blattodea

6347448E-8AFC-5BF6-B262-929B1242A4A1

#### Blattidae

08C679D0-5B21-5C76-A1A1-FF520B67C835

#### Periplaneta fuliginosa

Serville, 1839

404B039C-5594-5EF6-B64D-FBBD059503CF

##### Notes

Cho (1955), Cho (1965), Lee and Kwon (1981), ME (1993), Kwon et al. (1996), Lee and Jung (2001)

#### Periplaneta japonica

Karny, 1908

0E35B398-12C0-578A-8FBC-754FB39A609E

##### Notes

Lee and Jung (2001)

#### Ectobiidae

999EAF5E-6FC7-5454-A772-94580E3141E3

#### Blattella germanica

(Linnaeus, 1767)

0F2B8579-4E56-52E8-AB10-17E99E6762CD

##### Notes

Cho (1955), Cho (1965), Lee and Kwon (1981), ME (1993), Kwon et al. (1996), Lee and Jung (2001), Lim and Lee (2012)

#### Blattella nipponica

Asahina, 1963

A8345655-00D1-505D-84B3-C68B571C03FA

##### Notes

ME (1993), Kwon et al. (1996), Lee and Jung (2001), Lim et al. (2013), Nam et al. (2018), Won et al. (2023)

#### Episymploce brevipes

(Walker, 1871)

2BE5BAEE-A073-5CDD-A6E9-B039C7B67439

##### Notes

Cho (1955), Cho (1965), Lee and Kwon (1981), ME (1993), Kwon et al. (1996), Lee and Jung (2001)

#### Rhinotermitidae

0ED73E1B-DF51-5BEC-9BAD-F7978A7A3C81

#### Reticulitermes speratus kyushuensis

Morimoto, 1968

E7B2F0EB-20E2-5DC0-A576-0E941C12E028

##### Notes

HNIBR (2022)

#### Coleoptera

FEED5727-E69F-5AB1-A87F-C0E887448CE7

#### Anthicidae

7C8FF00E-BA41-59BA-8360-FD1BEE10B987

#### Anthelephila imperatrix

LaFerté-Sénectère, 1849

548FC69E-ACC4-59AC-9A21-A396A0467347

##### Notes

Oh et al. (2013), Nam et al. (2018), HNIBR (2022), Info and Park (2023)

#### Nitorus trigibber

(Marseul, 1876)

DDC1A595-2A56-5760-8B43-1E892A8433BE

##### Notes

Kwon et al. (1996), Lee and Jung (2001), HNIBR (2022)

#### Stricticollis valgipes

(Marseul, 1875)

3E41A9B1-E251-582F-AE07-B21F72C5EA7B

##### Notes

Nam et al. (2018)

#### Anthribidae

2B983D7D-3A9E-528F-8A3C-9B012E1A930F

#### Araecerus tarsalis

Sharp, 1891

7E402945-E91B-51C6-8118-028948949A8E

##### Notes

Park et al. (2012)

#### Opanthribus tessellatus

(Boheman, 1829)

D4F14477-A130-5C9A-9382-051A27446602

##### Notes

Lim and Lee (2012), HNIBR (2022)

#### Platystomos sellatus longicrus

Park, Hong, Woo & Kwon, 2001

D76DE3DE-F737-5221-B18E-A42166F5B502

##### Notes

Info (2023)

#### Tropideres naevulus

Faust, 1887

73C8A616-4668-51B0-A8F0-9CFD0E102417

##### Notes

Park et al. (2012)

#### Attelabidae

FE48705F-E839-5507-996D-CEE1C6CE35C0

#### Apoderus coryli

(Linnaeus, 1758)

0586913D-1B48-5D00-A807-CC046B266F11

##### Notes

Cho (1955), Cho (1965), Lee and Kwon (1981), ME (1993), Kwon et al. (1996), Lee and Jung (2001), HNIBR (2022)

#### Apoderus jekelii

(Roelofs, 1874)

35956D40-B8D8-5C52-B091-4D28FB359511

##### Notes

Cho (1955), Cho (1965), Lee and Kwon (1981), ME (1993), Kwon et al. (1996), Lee and Jung (2001), HNIBR (2022)

#### Aspidobyctiscus lacunipennis

(Jekel, 1860)

583952F3-690C-5E39-99E3-A67DF9EC3EBB

##### Notes

Won et al. (2023)

#### Byctiscus congener

(Jekel, 1860)

5C2A1962-1CC8-5370-B630-143603FF2330

##### Notes

Lim et al. (2013)

#### Buprestidae

D771B809-D74F-597A-A09B-4F0B7B5A3FF0

#### Agrilus chujoi

Kurosawa, 1985

59980ED9-F559-529C-BE36-A44DD4BCF744

##### Notes

Kwon et al. (1996), Lee and Jung (2001), Lee and Ahn (2012), HNIBR (2022)

#### Agrilus decoloratus

Kerremans, 1892

A3AB1417-BC3E-56AF-A7FC-AADE203821A8

##### Notes

Kim (1971), Lee and Kwon (1981), ME (1993), Kwon et al. (1996), Lee and Jung (2001), HNIBR (2022)

#### Agrilus discalis

Saunders, 1873

87CB355D-D18A-5AB4-B9AD-93D88EA9A646

##### Notes

Cho (1955), Cho (1965), Lee and Kwon (1981), ME (1993), Kwon et al. (1996), Lee and Jung (2001), Lee and Ahn (2012), Lim et al. (2013)

#### Agrilus marginicollis

Saunders, 1873

CA7F4691-E880-551C-992A-F0C301F70A35

##### Notes

Lee and Ahn (2012), HNIBR (2022)

#### Agrilus plasoni plasoni

Obenberger, 1917

3E433504-AE20-51D7-8F59-6C6FAE71B3A9

##### Notes

Lee and Ahn (2012)

#### Anthaxia proteus

Saunders, 1873

E07193FD-1511-5638-A69F-59EC3B865800

##### Notes

Lim et al. (2013)

#### Buprestis haemorrhoidalis japanensis

Saunders, 1873

49EDF1E0-080D-57EE-A33A-958F7A287DFF

##### Notes

Lee and Kwon (1981), ME (1993), Kwon et al. (1996), Lee and Jung (2001), HNIBR (2022)

#### Chalcophora japonica japonica

(Gory, 1840)

A30A97F2-FD6B-55BB-9FC9-B007890EF8C6

##### Notes

Oh et al. (2013), HNIBR (2022)

#### Nalanda wenigi wenigi

(Obenberger, 1927)

7DCAD0A3-9CBD-5EDF-9150-3E93FC11BCFE

##### Notes

Nam et al. (2018)

#### Trachys griseofasciatus

Saunders, 1873

868BC2C8-8447-5594-8130-A66E05D6DBB4

##### Notes

Kwon et al. (1996), Lee and Jung (2001), Lim et al. (2013)

#### Trachys minutus minutus

(Linnaeus, 1758)

F4B3E156-9797-5A1E-A6C4-D1C232B5861D

##### Notes

Lim and Lee (2012), HNIBR (2022)

#### Trachys variolaris

Saunders, 1873

86C85266-FD75-5BCC-9568-069166470C4B

##### Notes

Lim and Lee (2012), HNIBR (2022)

#### Trachys yanoi

Kurosawa, 1959

AB3F77C0-913E-5B18-8408-F42FDDBAAC00

##### Notes

HNIBR (2022)

#### Byrrhidae

BABED087-1FB5-5556-8816-A2B716A4AF9C

#### Simplocaria hispidula

Fairmaire, 1886

ECA20347-5130-5757-922F-A57639228380

##### Notes

Lim and Lee (2012), HNIBR (2022)

#### Byturidae

ED3E69D6-FC2F-55DA-AE0F-601B03ABF211

#### Byturus tomentosus

(De Geer, 1774)

0794DA31-9315-58D6-AE8D-41F4BF2761BA

##### Notes

Lim and Lee (2012), HNIBR (2022)

#### Cantharidae

2757D3BA-F5EF-5457-BDDA-872454F3BCDF

#### Lycocerus vitellinus

(Kiesenwetter, 1874)

09181645-7710-5F4F-8766-CC6E54F97DB1

##### Notes

Lim and Lee (2012), Lim et al. (2013), Nam et al. (2018), HNIBR (2022)

#### Carabidae

D5BEA4B6-FAAE-5BE8-B712-5A39498BF3B2

#### Acupalpus inornatus

Bates, 1873

9A2787C3-A13F-5F79-8D25-755B3569A17B

##### Notes

Nam et al. (2018)

#### Amara chalcites

Dejean, 1828

432361AC-F9CF-5BA0-835A-ADE7180AA302

##### Notes

Lee and Kwon (1981), ME (1993), Kwon et al. (1996), Lee and Jung (2001), HNIBR (2022)

#### Amara gigantea

(Motschulsky, 1844)

B781DB34-374B-5DEB-A14B-61E00A05D956

##### Notes

Lim et al. (2013), Won et al. (2023)

#### Amara hiogoensis

(Bates, 1873)

F50A0BD4-A0DF-5A53-9C27-0E39BBD6EAD5

##### Notes

Kwon et al. (1996), Lee and Jung (2001), Lim and Lee (2012), HNIBR (2022)

#### Amara ussuriensis

Lutshnik, 1935

C07528A6-1291-5260-8B92-068363128D3F

##### Notes

HNIBR (2022), Won et al. (2023)

#### Anisodactylus punctatipennis

Morawitz, 1862

DCEC2792-6AC1-576F-9028-370117A1DD86

##### Notes

Lee and Jung (2001), HNIBR (2022)

#### Anisodactylus signatus

(Panzer, 1796)

3BCDFC89-15B0-5190-AF2E-08CE11D6D8B5

##### Notes

Lee and Kwon (1981), ME (1993), Kwon et al. (1996), Lim and Lee (2012), Lim et al. (2013), Nam et al. (2018), HNIBR (2022)

#### Anisodactylus tricuspidatus

Morawitz, 1863

78CEADBC-259E-56CB-9780-D4103DE18D82

##### Notes

Lee and Jung (2001), HNIBR (2022)

#### Bradycellus laeticolor

Bates, 1873

1312D115-AF28-579D-9775-9581C22ED2B2

##### Notes

Nam et al. (2018)

#### Calleida lepida

L. Redtenbacher, 1868

728BB201-C4AD-5930-986D-1B8AE8BCE760

##### Notes

Lim and Lee (2012), HNIBR (2022)

#### Calleida onoha

Bates, 1873

8B7F8BF6-932A-59A2-91B7-218366567840

##### Notes

Lim and Lee (2012), Lim et al. (2013), Park et al. (2014), HNIBR (2022)

#### Calosoma inquisitor

(Linnaeus, 1758)

E4EC1B7D-C7F5-5BD8-AE09-6460BA061C0F

##### Notes

Lee and Kwon (1981), ME (1993), Kwon et al. (1996), Lee and Jung (2001), Lim and Lee (2012), HNIBR (2022)

#### Calosoma maximowiczi

(Morawitz, 1863)

A418A32D-4B35-5F95-BC35-054FE6C8133A

##### Notes

Cho (1955), Cho (1965), Lee and Kwon (1981), ME (1993), Kwon et al. (1996), Lee and Jung (2001), HNIBR (2022)

#### Chlaenius costiger costiger

Chaudoir, 1856

2E1E1AB6-DD42-515D-9D36-7C0328B0AA22

##### Notes

Lim and Lee (2012), Lim et al. (2013), Oh et al. (2013), Park et al. (2014), HNIBR (2022)

#### Chlaenius micans

(Fabricius, 1792)

9DACDF97-4382-5C0A-A498-C3BBB22A3907

##### Notes

Lim et al. (2013)

#### Chlaenius pallipes

(Gebler, 1823)

34D1FC9B-A0A3-5D04-B0AA-ACA363B78B44

##### Notes

Lim and Lee (2012), Lim et al. (2013), HNIBR (2022)

#### Chlaenius virgulifer

Chaudoir, 1876

2D03AB58-DAFC-5B52-9497-A76C10016E93

##### Notes

Lee and Jung (2001), Oh et al. (2013), HNIBR (2022)

#### Craspedonotus tibialis

Schaum, 1863

CC1946E5-06A7-55A7-AECF-73E87871E4CD

##### Notes

Cho (1955), Cho (1965), ME (1993), Kwon et al. (1996), Lim and Lee (2012), HNIBR (2022)

#### Dicranoncus femoralis

Chaudoir, 1850

12BEF2D5-B701-5E64-9B5C-2F74B4BE2F2F

##### Notes

Lim and Lee (2012), HNIBR (2022)

#### Diplocheila zeelandica

(Redtenbacher, 1868)

F1DD8113-DEC6-5BD1-B54B-9A6724D473F2

##### Notes

Lim and Lee (2012), HNIBR (2022)

#### Dolichus coreicus

Jedlička, 1936

89466F29-AC05-508F-B21F-211F611C10E3

##### Notes

Lim et al. (2013)

#### Dolichus halensis halensis

(Schaller, 1783)

27825AF6-7336-5E48-8D52-B80F76C09CAF

##### Notes

Lee and Kwon (1981), ME (1993), Kwon et al. (1996), Nam et al. (2018), HNIBR (2022)

#### Gyrochaetostylus atricomes

(Bates, 1873)

D47B2118-C164-5938-B342-C5880780480E

##### Notes

Won et al. (2023)

#### Harpalus capito

Morawitz, 1862

7EAEE241-0922-500B-A853-D540D1447E72

##### Notes

Lim et al. (2013), Won et al. (2023)

#### Harpalus chalcentus

Bates, 1873

0C8BE5D7-5913-5E72-847E-828F2639B9E1

##### Notes

Lee and Kwon (1981), ME (1993), Kwon et al. (1996), Lee and Jung (2001), Lim and Lee (2012), HNIBR (2022), Won et al. (2023)

#### Harpalus crates

Bates, 1883

607223A0-6516-5101-A7A7-F0AF9E69FD93

##### Notes

Lim et al. (2013)

#### Harpalus eous

Tschitscherine, 1901

41B8A538-E771-5EF2-9203-17D98B7144E2

##### Notes

Nam et al. (2018)

#### Harpalus griseus

(Panzer, 1796)

F8D5C6D9-2E77-539D-829C-9522AC869BD4

##### Notes

Nam et al. (2018)

#### Harpalus tinctulus luteicornoides

Breit, 1913

6F7BF5F4-0DB8-5FA1-948A-8BC87FC10B0A

##### Notes

Won et al. (2023)

#### Lebidia octoguttata

Morawitz, 1862

C422F853-52A9-54C1-A4BC-C6A45E41A7E0

##### Notes

Lee and Kwon (1981), ME (1993), Kwon et al. (1996), Lee and Jung (2001), Lim and Lee (2012), Lim et al. (2013), HNIBR (2022)

#### Lesticus magnus

(Motschulsky, 1860)

4B2A5C4D-E185-53FB-AF54-2A82C798D8E6

##### Notes

Kwon et al. (1996), Lee and Jung (2001), Nam et al. (2018), Won et al. (2023)

#### Metacolpodes buchannani

(Hope, 1831)

83BDFBC9-A286-5BBE-9BE6-43452DA8A2D9

##### Notes

Lee and Kwon (1981), ME (1993), Kwon et al. (1996), Lee and Jung (2001), HNIBR (2022), Won et al. (2023)

#### Nipponoharpalus discrepans

(Morawitz, 1862)

6CC5D714-F63B-58F5-8F41-37A74D2A086F

##### Notes

Won et al. (2023)

#### Notiophilus impressifrons

Morawitz, 1862

899C80EA-9F98-55C1-98B6-7539C4BFB5BD

##### Notes

Kwon et al. (1996), Lim and Lee (2012)

#### Panagaeus japonicus

Chaudoir, 1861

D1591C73-9999-59F5-BF17-DD851383659E

##### Notes

Lee and Jung (2001), HNIBR (2022)

#### Parena cavipennis

(Bates, 1873)

5982D65B-1625-5A22-B772-2C7F3EE24F2F

##### Notes

Kwon et al. (1996), Lee and Jung (2001), Lim and Lee (2012), Park et al. (2014), HNIBR (2022)

#### Poecilus coerulescens encopoleus

(Solsky, 1873)

8E6C8F7F-F44F-5656-9B53-52226BEE9D2D

##### Notes

Kwon et al. (1996), HNIBR (2022)

#### Poecilus fortipes

(Chaudoir, 1850)

9361E31E-825E-5A3B-B535-C8C49C73A834

##### Notes

Lee and Jung (2001), HNIBR (2022)

#### Pterostichus eschscholtzii

(Germar, 1823)

98F2C0DB-5331-51A8-AAB8-B6505354372F

##### Notes

Cho (1955), Cho (1965), Lee and Kwon (1981), ME (1993), Kwon et al. (1996), Lee and Jung (2001), HNIBR (2022)

#### Stenolophus difficilis

(Hope, 1845)

48042E34-68A6-57A7-9EA0-60C1B3996C2C

##### Materials

- **Type status:** Other material. **Occurrence:** recordedBy: Lee, D. Y.; sex: 1 male; lifeStage: adult; occurrenceID: 57F09465-ED9D-50F6-9805-6CE529A900C9; **Location:** island: Ulleungdo Island; country: Republic of Korea; stateProvince: Gyeongsangbuk-do; county: Ulleung-gun; locality: Nari basin, Nari-ri, Bukmyeon; **Event:** samplingProtocol: Sweeping net; eventDate: 6/11/2021

##### Notes

This is the first record of this species from Ulleungdo Island.

Distribution: Japan, Vietnam (Paik et al. 2006), Korea (National Institute of Biological Resources 2019)

#### Synuchus cycloderus

(Bates, 1873)

9C06C8E3-AEE8-5FF5-996A-88403F975DFA

##### Notes

Lim and Lee (2012), HNIBR (2022)

#### Synuchus dulcigradus

(Bates, 1873)

3931E5E3-E048-5CC0-9FA3-94C157A6A579

##### Notes

Kwon (2022)

#### Synuchus melantho

(Bates, 1883)

115E005E-077B-5A62-A39F-86624FA27063

##### Notes

Nam et al. (2018)

#### Tachyura fuscicauda

(Bates, 1873)

15AFB88E-729B-53A1-ADD9-B54F38947CC3

##### Notes

Oh et al. (2013), HNIBR (2022)

#### Tinoderus singularis

(Bates, 1873)

89E58093-C2FD-567D-A7D5-57BCF9CE35C2

##### Notes

Lim et al. (2013), Nam et al. (2018)

#### Trechus ephippiatus

Bates, 1873

1D4B3483-E5D6-523F-BC07-05A6E6E77A58

##### Notes

Oh et al. (2013), Nam et al. (2018), HNIBR (2022)

#### Trigonotoma lewisii

Bates, 1873

1085F6C8-9DB2-59B7-B8DC-AD4F3A6A8FFD

##### Notes

Lim and Lee (2012), HNIBR (2022)

#### Cerambycidae

C41F80A1-1EF5-5941-851D-01700DFB5B8F

#### Acalolepta sejuncta

(Bates, 1873)

3BF3470C-83A3-5A6F-8E1E-3D48BB9BDD2D

##### Notes

Won et al. (2023)

#### Acalolepta seunghwani

Danilevsky, 2013

E2C2E54C-3142-5CD3-9476-D5711287E20B

##### Notes

Lee and Kwon (1981), Lee (1987), ME (1993), Kwon et al. (1996), Lee and Jung (2001), HNIBR (2022)

#### Acanthocinus carinulatus

(Gebler, 1833)

4266DC6E-4195-585F-ABD9-FB34CE01FD04

##### Notes

Kwon (2022)

#### Aegosoma sinicum sinicum

White, 1853

9FC6080C-2FFB-5A61-A4D6-499C411013B7

##### Notes

Cho (1955), Cho (1965), Lee and Kwon (1981), Lee (1987), ME (1993), Kwon et al. (1996), Lee and Jung (2001), HNIBR (2022)

#### Agapanthia amurensis

Kraatz, 1879

32F68DE3-D8C2-5A04-BD2C-D5905F101CBF

##### Notes

Kwon (2022)

#### Anaesthetobrium luteipenne

Pic, 1923

8A9529A3-0F52-5318-95CA-B8A788AE7777

##### Notes

Lee and Kwon (1981), Lee (1987), ME (1993), Kwon et al. (1996), Lee and Jung (2001), HNIBR (2022)

#### Anaglyptus colobotheoides

(Bates, 1884)

EEB97E47-514A-5978-A39F-D68B6342AC4C

##### Notes

HNIBR (2022), Won et al. (2023)

#### Anoplophora chinensis

(Förster, 1771)

CCAA8340-E2F1-5E5B-A815-84320DEE9F65

##### Notes

Lim et al. (2013), HNIBR (2022)

#### Arhopaloscelis bifasciata

(Kraatz, 1879)

7D87E2E5-34C1-5D80-8D08-54EDC9E3F602

##### Notes

Lee (1987), Kwon et al. (1996), Lee and Jung (2001), Lim et al. (2013), HNIBR (2022), Won et al. (2023)

#### Arhopalus rusticus rusticus

(Linaeus, 1758)

03B71609-1B9B-5F5C-8A3F-FED1CB19452F

##### Notes

Lee and Kwon (1981), Lee (1987), ME (1993), Kwon et al. (1996), Lee and Jung (2001), HNIBR (2022), Won et al. (2023)

#### Asemum striatum

(Linnaeus, 1758)

C0197306-20BB-5567-AB5D-35CC82C31120

##### Notes

Lim et al. (2013)

#### Ceresium holophaeum

Bates, 1873

D6CB9527-5C79-5758-806C-60940D797E81

##### Notes

Lee and Kwon (1981), Lee (1987), ME (1993), Kwon et al. (1996), Lee and Jung (2001), HNIBR (2022)

#### Chlorophorus diadema diadema

(Motschulsky, 1854)

642DC2D6-4319-5C5A-ACAF-882437C02100

##### Notes

Cho (1955), Cho (1965), Lee and Kwon (1981), Lee (1987), ME (1993), Kwon et al. (1996), Lee and Jung (2001), HNIBR (2022)

#### Chlorophorus motschulskyi

(Ganglbauer, 1886)

90E94C6A-FE74-5AC5-AF0F-851146A7B146

##### Notes

Cho (1955), Cho (1965), Lee and Kwon (1981), ME (1993), Kwon et al. (1996), Lee and Jung (2001), HNIBR (2022)

#### Chlorophorus muscosus

(Bates, 1873)

F0FAF49F-61DD-532E-B0D7-A73E750BF00F

##### Notes

Cho (1955), Cho (1965), Lee and Kwon (1981), Lee (1987), ME (1993), Kwon et al. (1996), Lee and Jung (2001), HNIBR (2022)

#### Chlorophorus simillimus

(Kraatz, 1879)

69C7E234-44A6-56FD-9C3E-99E3113E50F2

##### Notes

Lee and Kwon (1981), ME (1993), Kwon et al. (1996), Lee and Jung (2001), HNIBR (2022)

#### Egesina bifasciana bifasciana

(Matsushita, 1933)

C72173BB-3C80-5679-BEFC-8EEEF4580E85

##### Notes

Lee (1987), Kwon et al. (1996), Lee and Jung (2001), HNIBR (2022), Won et al. (2023)

#### Eutetrapha sedecimpunctata sedecimpunctata

(Motschulsky, 1860)

A76FF54D-85CC-55E0-BE49-CFC194AA2BBD

##### Notes

Cho (1955), Cho (1965), Lee and Kwon (1981), ME (1993), Kwon et al. (1996), Lee and Jung (2001), HNIBR (2022)

#### Exocentrus fisheri

Gressitt, 1935

B9B29D7E-FB2A-5737-9DEC-D4BEE084EB27

##### Notes

Lim et al. (2013)

#### Exocentrus guttulatus ussuricus

Tsherepanov, 1973

2F5FD32A-5BF7-543B-AD96-93282B6796CF

##### Notes

Lee and Jung (2001), HNIBR (2022)

#### Exocentrus lineatus

Bates, 1873

AF410158-FFF8-58FB-BC20-EEAEAB5A8810

##### Notes

Lee and Kwon (1981), ME (1993), Kwon et al. (1996), Lee and Jung (2001), HNIBR (2022)

#### Macroleptura thoracica

(Creutzer, 1799)

AD09E246-0936-579E-9CC6-69B6C3FDECFD

##### Notes

Cho (1955), Cho (1965), Lee and Kwon (1981), ME (1993), Kwon et al. (1996), Lee and Jung (2001), HNIBR (2022)

#### Mesosa hirsuta continentalis

Hayashi, 1964

FA52B87D-7614-536F-BBDF-FC241E1CD9A1

##### Notes

Kwon (2022)

#### Mesosa longipennis

Bates, 1873

713DC910-289B-5FA8-8B7F-C96DD10BC2F1

##### Notes

Kim (1971), Lee and Kwon (1981), ME (1993), Kwon et al. (1996), Lee and Jung (2001), HNIBR (2022)

#### Mimectatina divaricata divaricata

(Bates, 1884)

360811A9-ABA6-5832-8E56-7E712CFBD3D2

##### Notes

Lee (1987), Kwon et al. (1996), Lee and Jung (2001), HNIBR (2022), Won et al. (2023)

#### Obrium obscuripenne obscuripenne

Pic, 1904

A85112C2-E7DE-5A9A-9EEB-F1E805EEB74B

##### Notes

Info (2023)

#### Phymatodes infasciatus

(Pic, 1935)

5317DB36-8BF0-57BA-821A-5D84E3CD1020

##### Notes

Lee (1987), Kwon et al. (1996), Lee and Jung (2001), HNIBR (2022)

#### Pidonia debilis

(Kraatz, 1879)

9A4B88F2-162B-59C3-ACB9-D4CFA7E5532D

##### Notes

Kim (1971), Lee and Kwon (1981), ME (1993), Kwon et al. (1996), Lee and Jung (2001), HNIBR (2022)

#### Prionus insularis insularis

Motschulsky, 1857

535AE35C-2B57-5224-B316-090795E3963B

##### Notes

Cho (1955), Cho (1965), Lee and Kwon (1981), Lee (1987), ME (1993), Kwon et al. (1996), Lee and Jung (2001), HNIBR (2022)

#### Psacothea hilaris hilaris

(Poscoe, 1857)

BC8EC9C0-73E8-559B-ABA8-A094DBE80DC6

##### Notes

Cho (1955), Cho (1965), Lee and Kwon (1981), Lee (1987), ME (1993), Kwon et al. (1996), Lee and Jung (2001), Oh et al. (2013), HNIBR (2022)

#### Psephactus remiger remiger

Harold, 1879

39850B88-6B11-5766-816A-9AC3A50F4ACB

##### Notes

Lim and Lee (2012), HNIBR (2022)

#### Pterolophia caudata

(Bates, 1873)

150C4A23-BAE4-5A50-94E1-ADA3461FC1A4

##### Notes

Lee (1987), Kwon et al. (1996), Lee and Jung (2001)

#### Pterolophia granulata

(Motschulsky, 1866)

E53C8737-0541-59D0-8DE2-BFC4C3116C97

##### Notes

Cho (1955), Cho (1965), Lee and Kwon (1981), Lee (1987), ME (1993), Kwon et al. (1996), Lee and Jung (2001), Lim et al. (2013), Nam et al. (2018)

#### Pterolophia multinotata

(Pic, 1931)

7084161D-E492-58CD-8990-2DF77A077898

##### Notes

Lee (1987), Kwon et al. (1996), Lee and Jung (2001), Lim et al. (2013), HNIBR (2022)

#### Rhagium inquisitor rugipenne

Reitter, 1898

130B5BC0-9944-5BA0-97E2-DC13BF75558C

##### Notes

Cho (1955), Cho (1965), Lee and Kwon (1981), ME (1993), Kwon et al. (1996), Lee and Jung (2001), Nam et al. (2018), HNIBR (2022)

#### Ropica coreana

Breuning, 1980

BBEF9410-CFDC-5774-870A-BD16B4D3CC8C

##### Notes

Lee (1987), Kwon et al. (1996), Lee and Jung (2001), HNIBR (2022)

#### Saperda octomaculata

Blessig, 1873

DEFE5484-BBC1-5870-9777-9C717470EDDF

##### Notes

Won et al. (2023)

#### Stenygrinum quadrinotatum

Bates, 1873

3CBD77A6-4B32-5FB8-800E-BFE683D35F9B

##### Notes

Cho (1955), Cho (1965), Lee and Kwon (1981), ME (1993), Kwon et al. (1996), Lee and Jung (2001), HNIBR (2022)

#### Stictoleptura rubra

(Linnaeus, 1758)

E9B6C990-825B-5528-8972-B6A2CD9AE1FB

##### Notes

Lee and Kwon (1981), Lee (1987), ME (1993), Kwon et al. (1996), Lee and Jung (2001), HNIBR (2022)

#### Teratoclytus plavilstshikovi

Zaitzev, 1937

C0D9388D-BBAD-5894-86EA-8845B3391F94

##### Notes

Lee (1987), Kwon et al. (1996), Lee and Jung (2001), HNIBR (2022)

#### Xylotrechus chinensis

(Chevrolat, 1852)

06A3A630-8348-546D-8994-13BE3194B593

##### Notes

Cho (1955), Cho (1965), Lee and Kwon (1981), Lee (1987), ME (1993), Kwon et al. (1996), Lee and Jung (2001), HNIBR (2022)

#### Xylotrechus clarinus

Bates, 1884

E35443DC-FF50-5CFD-AF5C-18EF74972D31

##### Notes

Kwon et al. (1996), Lee and Jung (2001), HNIBR (2022)

#### Xystrocera globosa

(Olivier, 1795)

4D2B1EAB-36AF-5CF8-8AF5-989DD65904BE

##### Notes

Lee and Kwon (1981), ME (1993), Kwon et al. (1996), Lee and Jung (2001), HNIBR (2022)

#### Chrysomelidae

F6E9023D-C59A-575D-AB6F-E4AE241A2EE5

#### Agelasa nigriceps

Motschulsky, 1861

F8F663BF-7DD2-5D02-A70A-348842E832BB

##### Notes

Lim et al. (2013)

#### Altica caerulescens

(Baly, 1874)

5DF32611-30BD-5402-93A2-0E5ADAEF73A3

##### Notes

Lee and Kwon (1981), ME (1993), Kwon et al. (1996), Lee and Jung (2001), Lim et al. (2013), HNIBR (2022)

#### Altica fragariae

(Nakane, 1955)

D6B99F53-6BFD-5B16-BA82-F04401FBFDDC

##### Notes

Nam et al. (2018)

#### Altica oleracea oleracea

(Linnaeus, 1758)

7A9C5BFB-C54B-5C0B-8172-59144C93E163

##### Notes

Won et al. (2023)

#### Altica viridicyanea

(Baly, 1874)

1F0D4E91-5925-5673-81F3-03A06295B1FB

##### Notes

Lim et al. (2013)

#### Argopistes biplagiatus

Motschulsky, 1860

F34CAC6A-B8FC-5424-A70D-63645A41F966

##### Notes

Kim (1971), Lee and Kwon (1981), ME (1993), Kwon et al. (1996), Lee and Jung (2001), Lim et al. (2013)

#### Argopistes tsekooni

Chen, 1934

F50E11DC-E9EF-5016-88A0-EEB487231DD6

##### Notes

Kim (1971), Lee and Kwon (1981), ME (1993), Kwon et al. (1996), Lee and Jung (2001), HNIBR (2022), Won et al. (2023)

#### Aulacophora indica

(Gmelin, 1790)

70F4FDF3-605F-5A2F-82A1-C2A383D10B9B

##### Notes

Lee and Kwon (1981), ME (1993), Kwon et al. (1996), Lee and Jung (2001), HNIBR (2022)

#### Basilepta punctifrons

An, 1988

64D015E1-B10C-590F-820D-ABAFFF71E4F8

##### Notes

Lim and Lee (2012), HNIBR (2022)

#### Bruchidius japonicus

(Harold, 1878)

36623DEB-905B-50FB-826D-E2B001485B26

##### Notes

Won et al. (2023)

#### Callosobruchus chinensis

(Linnaeus, 1758)

12748F85-66AA-570C-9A01-D4C2E500C1B9

##### Notes

Lee and Jung (2001), Lim and Lee (2012), HNIBR (2022)

#### Cassida nebulosa

Linnaeus, 1758

0CA79B7B-E996-5A9F-95DC-E4E5FDC6D599

##### Notes

Kwon et al. (1996), Lee and Jung (2001), Lim and Lee (2012), HNIBR (2022)

#### Cassida piperata

Hope, 1842

33DA6CEA-45A0-5194-893A-8029E1D8261F

##### Notes

Kwon et al. (1996), Lee and Jung (2001), Lim and Lee (2012), Lim et al. (2013), Oh et al. (2013), HNIBR (2022)

#### Chaetocnema concinna

(Marsham, 1802)

C87596D9-2DB7-52B4-84E7-64FA065406A6

##### Notes

Kwon et al. (1996), Lee and Jung (2001), HNIBR (2022)

#### Chaetocnema concinnicollis

(Baly, 1874)

5BBB7998-866B-51F8-8933-4BF43620EA6C

##### Notes

ME (1993), Kwon et al. (1996), HNIBR (2022)

#### Chrysolina aurichalcea

(Mannerheim, 1825)

E42BBA17-0444-5718-9EBD-227315B473E5

##### Notes

Lee and Kwon (1981), ME (1993), Kwon et al. (1996), Lee and Jung (2001), Lim et al. (2013), Oh et al. (2013), HNIBR (2022)

#### Chrysolina exanthematica exanthematica

(Wiedemann, 1821)

C6080581-F6FA-53CB-92F0-973F7A0F92C3

##### Notes

Lim et al. (2013)

#### Coptocephala orientalis

Baly, 1873

5C0DD2DA-2821-5AAD-8CE7-B9784B330F2B

##### Notes

Lim et al. (2013)

#### Cryptocephalus bilineatus

(Linnaeus, 1767)

3654C29B-262C-59C2-8FB2-53D73E789F4F

##### Notes

Kwon et al. (1996), Lee and Jung (2001)

#### Cryptocephalus fulvus fuscolineatus

Chûjô, 1940

DD349CB0-612C-517D-975F-F525E9973ACE

##### Notes

Lee and Kwon (1981), ME (1993), Kwon et al. (1996), Lee and Jung (2001), HNIBR (2022)

#### Cryptocephalus peliopterus peliopterus

Solsky, 1872

3703FE52-73B7-5321-80F2-E1B6ECC2D46C

##### Notes

Cho (1955), Cho (1965), Lee and Jung (2001), HNIBR (2022)

#### Cryptocephalus pseudopopuli

Schöller, 2011

467D72CE-7150-52CB-9A03-3AE23D415BB1

##### Notes

Schöller (2011)

#### Demotina fasciata

Baly, 1874

A78AF8C5-E8E0-53F0-BE95-A24ED025F0F6

##### Notes

Lim et al. (2013)

#### Demotina modesta

Baly, 1874

B4684DDC-077E-5005-8F33-C491141905CE

##### Notes

Won et al. (2023)

#### Galerucella grisescens

(Joannis, 1865)

EAB4D77B-5F9C-52C9-9D95-3D573A60B960

##### Notes

Lee and Kwon (1981), ME (1993), Kwon et al. (1996), Lee and Jung (2001), Lim and Lee (2012), HNIBR (2022)

#### Gallerucida bifasciata

Motschulsky, 1861

543A411B-9C06-5C3A-B2A6-3498A397DA80

##### Notes

Cho (1955), Cho (1965), Lee and Kwon (1981), Lee (1987), ME (1993), Kwon et al. (1996), Lee and Jung (2001), Lim and Lee (2012), Lim et al. (2013), Oh et al. (2013), HNIBR (2022), Won et al. (2023)

#### Heteraspis lewisii

(Baly, 1874)

129134BF-6619-5A8C-8152-4276D78C8D1F

##### Notes

Lee and Kwon (1981), ME (1993), Kwon et al. (1996), Lee and Jung (2001), HNIBR (2022)

#### Lema concinnipennis

Baly, 1865

CAE60071-1259-57D6-BCE6-3D32C3E8345F

##### Notes

Kim (1971), Lee and Kwon (1981), ME (1993), Kwon et al. (1996), Lee and Jung (2001), HNIBR (2022), Won et al. (2023)

#### Lema diversa

Baly, 1873

91245820-613F-57F9-955D-D1D4F4ECD39F

##### Notes

Lee and Kwon (1981), ME (1993), Kwon et al. (1996), Lee and Jung (2001), Lim and Lee (2012), Lim et al. (2013), HNIBR (2022)

#### Longitarsus aphthonoides

Weise, 1887

7F95FEB4-E71B-5121-8079-6A88FDA9E61A

##### Notes

Kwon et al. (1996), Lee and Jung (2001), HNIBR (2022)

#### Longitarsus succineus

(Foudras, 1860)

A748BC51-2018-5CD3-8244-EB9110B1D48F

##### Notes

Nam et al. (2018)

#### Monolepta quadriguttata

(Motschulsky, 1860)

FBA85B36-80CA-5004-84B8-12953DFCE131

##### Notes

Nam et al. (2018)

#### Neofidia fulvus

(Baly, 1978)

ED181C68-C67D-5A15-BA58-399912E29463

##### Notes

Lim and Lee (2012), HNIBR (2022)

#### Oomorphoides cupreatus

(Baly, 1873)

A1C08EAF-13C6-5C1F-8D65-940C7FCEEC1F

##### Notes

Lim and Lee (2012), HNIBR (2022)

#### Ophraella communa

Le Sage, 1986

C9538620-2279-57A2-9BEE-A7C199A597D9

##### Notes

Kwon (2022)

#### Oulema erichsonii

(Suffrian, 1841)

13818050-039A-5ADF-8209-D021FC423741

##### Notes

Lim et al. (2013)

#### Oulema viridula

(Gressitt, 1942)

55C84284-34C7-5694-841D-99BE1975FD0B

##### Notes

Lim et al. (2013)

#### Pagria consimilis

(Baly, 1874)

3A98B11F-C17A-5C02-AD7F-F9170DAA641B

##### Notes

Lim et al. (2013), Won et al. (2023)

#### Paridea angulicollis

(Motschulsky, 1854)

A8496B3D-73C9-58FF-8807-15F1916D8EDF

##### Notes

Won et al. (2023)

#### Phygasia fulvipennis

(Baly, 1874)

1723DE83-45D6-54CF-8118-DFFC2EA98900

##### Notes

Lim and Lee (2012), HNIBR (2022)

#### Phyllotreta vittula

(Redtenbacher, 1849)

E9AAA9F0-C1B4-5400-ACA3-D18136FC5F13

##### Notes

Kwon et al. (1996), Lee and Jung (2001), HNIBR (2022)

#### Physosmaragdina nigrifrons

(Hope, 1843)

9CE5454F-FFD8-5F62-ACA4-75A336C63FE7

##### Notes

Kim (1971), Lee and Kwon (1981), ME (1993), Lee and Jung (2001), Kwon et al. (1996), Lim et al. (2013), Oh et al. (2013), HNIBR (2022)

#### Plagiodera versicolora

(Laicharting, 1781)

E49D03FD-2C5A-5A76-9981-42FD628AC67E

##### Notes

Cho (1955), Cho (1965), Lee and Kwon (1981), ME (1993), Kwon et al. (1996), Lee and Jung (2001), HNIBR (2022)

#### Plagiosterna aenea aenea

(Linnaeus, 1758)

74B21850-9197-5002-BF4F-D894E687ED2C

##### Notes

Info (2023)

#### Psylliodes punctifrons

Baly, 1874

2188AC00-6C69-5E19-A2FA-80142E974726

##### Notes

Kwon et al. (1996), Lee and Jung (2001), HNIBR (2022)

#### Pyrrhalta humeralis

(Chen, 1942)

44895554-7050-5841-976B-731F1C182399

##### Notes

Lim et al. (2013)

#### Pyrrhalta viburni

(Paykull, 1799)

935F61F1-F708-5AB4-8960-475013356D4D

##### Notes

Cho (1955), Cho (1965), Lee and Kwon (1981), ME (1993), Kwon et al. (1996), Lee and Jung (2001), HNIBR (2022)

#### Syneta adamsi

Baly, 1877

4683A2BC-8FB2-5ACD-A973-FE0FCAE53F64

##### Notes

Won et al. (2023)

#### Taphinellina minuta

(Joannis, 1865)

D1144FBD-96E9-5B54-8F9B-E450F2797CCC

##### Notes

Lim et al. (2013)

#### Cicindelidae

E2784ED9-207D-53A7-A8C9-84D6B921FABB

#### Cicindela chinensis

De Geer, 1774

5A5462AC-D642-5520-B584-D485DA1D7FCC

##### Notes

Lee and Kwon (1981), ME (1993), Kwon et al. (1996), Lee and Jung (2001), HNIBR (2022)

#### Cylindera elisae

(Motschulsky, 1859)

CF288B7A-F672-522D-A8F6-0BE8E53D1B76

##### Notes

Cho (1955), Cho (1965), Lee and Kwon (1981), ME (1993), Kwon et al. (1996), Lee and Jung (2001), HNIBR (2022)

#### Coccinellidae

48FA7FF0-9143-5850-B3F6-032C053514C1

#### Calvia muiri

(Timberlake, 1943)

0543C3F9-CEFC-5863-98F8-955824DEFBDC

##### Notes

Lee and Jung (2001), Lim and Lee (2012), HNIBR (2022), Won et al. (2023)

#### Chilocorus kuwanae

Silvestri, 1909

76A97953-24CC-5936-B3AC-A8614CA96B8E

##### Notes

Lee and Jung (2001), Lim and Lee (2012), HNIBR (2022)

#### Coccinella septempunctata

Linnaeus, 1758

BA4ED9F1-E877-515E-8E1E-9C62F8082D54

##### Notes

Cho (1965), Lee and Kwon (1981), ME (1993), Kwon et al. (1996), Lee and Jung (2001), Lim and Lee (2012), Lim et al. (2013), Oh et al. (2013), HNIBR (2022)

#### Epilachna quadricollis

(Dieke, 1947)

1811302D-BA6B-54E2-8D17-BFE0257AFEF9

##### Notes

Won et al. (2023)

#### Harmonia axyridis

(Pallas, 1773)

87E188CB-9F73-552E-AF20-E221887DA2A3

##### Notes

Cho (1955), Cho (1965), Lee and Kwon (1981), ME (1993), Kwon et al. (1996), Lee and Jung (2001), Lim and Lee (2012), Lim et al. (2013), Oh et al. (2013), HNIBR (2022), Won et al. (2023)

#### Harmonia yedoensis

(Takizawa, 1917)

C6DA05F5-C9C8-52EE-B823-B5D7A64F92B0

##### Notes

Lim et al. (2013)

#### Henosepilachna vigintioctomaculata

(Motschulsky, 1858)

01403215-3AA6-57D9-90B1-BDF5F1171619

##### Notes

Cho (1955), Cho (1965), Lee and Kwon (1981), ME (1993), Kwon et al. (1996), Lee and Jung (2001), Oh et al. (2013), Lim and Lee (2012), Lim et al. (2013), HNIBR (2022)

#### Hyperaspis asiatica

Lewis, 1896

C47D3B67-B55A-5626-B47F-EDAD5329CEF3

##### Notes

Kwon et al. (1996), Lee and Jung (2001), HNIBR (2022)

#### Illeis koebelei koebelei

Timberlake, 1943

760BB03E-330B-529F-A024-A38A64FE15BE

##### Notes

Kwon et al. (1996), Lee and Jung (2001), Lim and Lee (2012), Lim et al. (2013), HNIBR (2022), Won et al. (2023)

#### Myzia oblongoguttata

(Linnaeus, 1758)

92BACC06-6B8F-5B19-BA66-3BC69D8E7F10

##### Notes

Lee and Jung (2001), HNIBR (2022)

#### Propylea japonica

(Thunberg, 1781)

A00A79BA-3BAC-5E71-A76E-C7EBD00DFFBD

##### Notes

Kim (1971), Lee and Kwon (1981), ME (1993), Kwon et al. (1996), Lee and Jung (2001), Lim and Lee (2012), Lim et al. (2013), HNIBR (2022)

#### Propylea quatuordecimpunctata

(Linnaeus, 1758)

C43E50C3-2007-5A04-B26A-9EEEC92D0E2C

##### Notes

Kim (1971), Lim et al. (2013)

#### Serangium japonicum

Chapin, 1940

B8D1C407-284F-5D7C-AE43-DAAA15A2A10A

##### Notes

Nam et al. (2018)

#### Vibidia duodecimguttata

(Poda, 1761)

2BC603D8-2D91-5934-8B6E-F3E3238D2BD1

##### Notes

Lim and Lee (2012), Oh et al. (2013), HNIBR (2022)

#### Curculionidae

C1B393E0-701A-5C41-955C-1B5CD51614C4

#### Ambrosiophilus atratus

(Eichhoff, 1876)

D992B266-F1FD-5B7D-AA56-369769F7AEDE

##### Materials

- **Type status:** Other material. **Occurrence:** recordedBy: Lee, D. Y.; sex: 1 male; lifeStage: adult; occurrenceID: 33D97097-1562-57FE-8390-5C74CD46598A; **Location:** island: Ulleungdo Island; country: Republic of Korea; stateProvince: Gyeongsangbuk-do; county: Ulleung-gun; locality: Seonginbong, Dodong-ri, Ulleungeup; minimumElevationInMeters: 300; maximumElevationInMeters: 500; **Event:** samplingProtocol: Sweeping net; eventDate: 28-08-2020

##### Notes

It was previously misidentified as *Saprositesjaponicus* Waterhouse, 1875 by Won et al. (2023). This is the first record of this species from Ulleungdo Island.

Distribution: Burma, China, Indonesia, Japan, Korea, New Guinea, Philippines, Taiwan (Park 2016), Canada, Italy (Faccoli 2008)

#### Anthinobaris dispilota

(Solsky, 1870)

EFEA604C-0496-5B91-833B-92EA5464407D

##### Notes

Hong et al. (2011), Nam et al. (2018)

#### Barinomorphus antennatus

Morimoto, 1962

C4EC1B6C-3139-5C38-8E30-B4ACBEAEE5F3

##### Notes

Lim et al. (2013)

#### Catapionus obscurus

Sharp, 1896

11528563-8564-51E5-8E14-1E68C9AED317

##### Notes

Kwon et al. (1996), Lee and Jung (2001), HNIBR (2022)

#### Ceutorhynchus albosuturalis

(Roelofs, 1875)

8E44A1A6-BD42-5405-8914-7744BDC3D6EF

##### Notes

Hong et al. (2011), HNIBR (2022)

#### Ceutorhynchus ibukianus

Hustache, 1916

04B0B993-1FA4-5FCD-831C-8E2DA2E60AF2

##### Notes

Nam et al. (2018)

#### Cnestus mutilatus

(Blandford, 1894)

D199C25C-CE50-5B3A-ADCE-D26D82E88322

##### Notes

Lim et al. (2013)

#### Cosmobaris scolopacea

(Germar, 1819)

FCD2FFE0-CD13-56E1-AC11-88F89511A8C8

##### Notes

Kwon et al. (1996), Lee and Jung (2001), HNIBR (2022)

#### Curculio distinguendus

(Roelofs, 1875)

FA6229BD-3C7E-56C7-906E-D6EF5DB7627E

##### Notes

Lim et al. (2013)

#### Dryotribus mimeticus

Horn, 1873

EC205482-438D-5309-A9E6-C9D1A78AC2C8

##### Notes

Hong et al. (2012)

#### Hylobius haroldi

Faust, 1882

CFD4EC0D-2DB3-5740-80B7-CA995A8F5D81

##### Notes

Hong et al. (2011), Lim and Lee (2012), Lim et al. (2013), HNIBR (2022)

#### Hylobius pinastri

(Gyllenhal, 1813)

99C2DB68-842A-5D3B-BEAD-027F26B17F29

##### Notes

Info (2023)

#### Hypera graeseri

Faust, 1887

9CB4C90D-9283-5F32-9403-2E7D8B5DCED9

##### Notes

Lim and Lee (2012), HNIBR (2022)

#### Hypera postica

(Gyllenhal, 1813)

61F919C9-37C0-5B0E-B03E-DEC1F7CDDE4C

##### Notes

Lim and Lee (2012), HNIBR (2022)

#### Lepidepistomodes griseoides

(Zumpt, 1937)

DDC27B19-F752-5972-8D73-BDE0EED1C377

##### Notes

Kwon (2022)

#### Lixus acutipennis

(Roelofs, 1873)

E3CA3711-49BF-5536-BF5B-7374CC3EE5B9

##### Notes

Lee and Kwon (1981), ME (1993), Kwon et al. (1996), Lee and Jung (2001), Lim and Lee (2012), HNIBR (2022)

#### Lixus fasciculatus

Boheman, 1835

BB082FDA-4148-5D31-86A2-036105859403

##### Notes

Lim and Lee (2012), HNIBR (2022)

#### Mecopomorphus amurensis

(Heyden, 1884)

2B51429E-3517-5E2F-BAA5-1428E2B0C7AB

##### Notes

Hong et al. (2011)

#### Myosides seriehispidus

Roelofs, 1873

75747B21-9532-5606-9EA4-A45AC80F197E

##### Notes

Lim et al. (2013), Han et al. (2014), Nam et al. (2018)

#### Orchestes harunire

(Morimoto, 1984)

1B209E70-0706-5366-90A0-EF0FDB96D597

##### Notes

Hong et al. (2012)

#### Orchestes horii

(Kôno, 1937)

B5F7086E-D7AC-5D25-8933-FB310EAB1BAC

##### Notes

Hong et al. (2012)

#### Orchestes lateritius

(Morimoto, 1984)

112DCFA1-71CA-5E6C-9A3E-98DBB53A578C

##### Notes

Hong et al. (2012)

#### Orchestes mutabilis

Boheman, 1843

BE2EDD7B-3A67-5EFB-8B29-BAB54845ECA0

##### Notes

Hong et al. (2012)

#### Orchestes sanguinipes

Roelofs, 1874

9CD52B13-C65A-5079-9930-93BB74DAD178

##### Notes

Nam et al. (2018)

#### Orochlesis takaosanus

Kôno, 1932

18A01DA8-1A57-5498-A834-E5AD865958B9

##### Notes

Hong et al. (2011)

#### Pellobaris melancholica

(Roelofs, 1875)

5F5946D9-E83E-59AC-B28B-0A2220D309D4

##### Notes

Hong et al. (2011)

#### Polygraphus jezoensis

Niisima, 1909

DF8D5778-20A8-525F-9AEB-D92CE86F844D

##### Materials

- **Type status:** Other material. **Occurrence:** recordedBy: Lee, D. Y.; sex: 1 male; lifeStage: adult; occurrenceID: 00A10C1D-F6BE-5E74-8040-31558F724E99; **Location:** island: Ulleungdo Island; country: Republic of Korea; stateProvince: Gyeongsangbuk-do; county: Ulleung-gun; locality: Seonginbong, Dodong-ri, Ulleungeup; minimumElevationInMeters: 300; maximumElevationInMeters: 500; **Event:** samplingProtocol: Sweeping net; eventDate: 7/7/2020

##### Notes

This is the first record of this species from Ulleungdo Island.

Distribution: Korea (Park 2018), Japan, Russia (Nobuchi 1979)

#### Pseudoedophrys hilleri

(Faust, 1889)

CB8DE56A-9DCA-5E2E-BF37-4F6C2A88533D

##### Notes

Lim et al. (2013), Han et al. (2014), Won et al. (2023)

#### Psilarthroides czerskyi

(Zaslavskij, 1956)

3D3AA070-427B-5F08-A8BA-C7D20D7636DF

##### Materials

- **Type status:** Other material. **Occurrence:** recordedBy: Lee, D. Y.; sex: 1 female; lifeStage: adult; occurrenceID: 89481697-EB92-569C-B19A-C2151273226F; **Location:** island: Ulleungdo Island; country: Republic of Korea; stateProvince: Gyeongsangbuk-do; county: Ulleung-gun; locality: Taeharyeong, Namseo-ri, Seomyeon; **Event:** samplingProtocol: Sweeping net; eventDate: 8/17/2021

##### Notes

This is the first record of this species from Ulleungdo Island.

Distribution: China, Korea (Morimoto and Lee 1992), Japan (Morimoto and Miyakawa 1985), Russia (Zaslavskij 1956)

#### Ptochidius tessellatus

(Motschulsky, 1858)

B09F7E4F-4693-516F-B935-F06809722156

##### Notes

Han et al. (2014)

#### Rhinoncus cribricollis

Hustache, 1916

53662BC0-524B-5A34-ABEF-1199484727B2

##### Notes

Lee and Jung (2001), Hong et al. (2011), Lim et al. (2013), HNIBR (2022)

#### Rhinoncus sibiricus

Faust, 1893

027D439B-5715-5B47-87CF-F7E474C1933D

##### Notes

Hong et al. (2011)

#### Scepticus griseus

(Roelofs, 1873)

EA9222EE-26FE-5ED4-A7EB-67944201C8D7

##### Notes

Kwon et al. (1996), Lee and Jung (2001), Lim et al. (2013), Nam et al. (2018), HNIBR (2022)

#### Scepticus uniformis

Kôno, 1930

BB0B0350-55D2-5099-9326-DC5A56C05C7E

##### Notes

Kwon et al. (1996), Lee and Jung (2001), HNIBR (2022)

#### Scleropteroides hypocrita

(Hustache, 1916)

817F9B63-1BCA-59D5-8770-3EF6D068CAE6

##### Notes

Hong et al. (2011), HNIBR (2022)

#### Scolytoplatypus mikado

Blandford, 1893

56FE81C4-31A3-55ED-8912-D26865072542

##### Notes

Kwon et al. (1996), Lee and Jung (2001), HNIBR (2022)

#### Scolytoplatypus tycon

Blandford, 1893

4A1A28C2-61B3-50F4-B2F5-C987D63DBEA2

##### Materials

- **Type status:** Other material. **Occurrence:** recordedBy: Lee, D. Y.; sex: 1 male, 2 female; lifeStage: adult; occurrenceID: F5978829-78B1-5B5E-8C00-F3D1D5378625; **Location:** island: Ulleungdo Island; country: Republic of Korea; stateProvince: Gyeongsangbuk-do; county: Ulleung-gun; locality: Seonginbong, Dodong-ri, Ulleungeup; minimumElevationInMeters: 300; maximumElevationInMeters: 500; **Event:** samplingProtocol: Sweeping net; eventDate: 8/28/2020

##### Notes

This is the first record of this species from Ulleungdo Island.

Distribution: China, Korea, Japan, Russia, Taiwan (Nobuchi 1967)

#### Sitona lineatus

(Linnaeus, 1758)

820E0869-243D-5FD7-A284-DDE331F6AD67

##### Notes

Lee and Jung (2001), HNIBR (2022)

#### Xenomimetes destructor

Wollaston, 1873

53303F40-7E47-5557-80E1-67382F91908A

##### Notes

Hong et al. (2012)

#### Xyleborinus saxeseni

Reitter, 1913

CFCF8EAE-06F1-5D0F-9C80-EE5379CC89F2

##### Notes

Nam et al. (2018)

#### Dermestidae

2BE50A0F-C045-5058-91FF-BA6A81FB3DFF

#### Dermestes tessellatocollis tessellatocollis

Motschulsky, 1860

217D8F9D-9EA3-524A-A0CC-047AA20DF2EC

##### Notes

Lee and Jung (2001), HNIBR (2022)

#### Thaumaglossa rufocapillata

Redtenbacher, 1867

52D45701-253E-5F4C-A2E5-5BB29E7C4523

##### Notes

Info (2023)

#### Dryophthoridae

C412611C-9A03-5736-AED1-3FE1D917FC75

#### Sipalinus gigas

(Fabricius, 1775)

4F9C0ED6-A909-5480-9F12-2E9373FBA536

##### Notes

Lim and Lee (2012), Lim et al. (2013), HNIBR (2022)

#### Dytiscidae

8470DCA0-7F83-5581-9ED7-F6280ACC01D3

#### Agabus japonicus

Sharp, 1873

B37C3593-7773-591C-BE99-AB6B8C81A5BC

##### Notes

Lim and Lee (2012), Lee and Ahn (2018), HNIBR (2022)

#### Agabus regimbarti

Zaitzev, 1906

EF6CEE94-0000-5BC4-BD31-85792F5F65FE

##### Notes

Lim and Lee (2012), HNIBR (2022)

#### Copelatus weymarni

Balfour-Browne, 1947

FB7D8F42-A57D-5192-A505-7402E1F7A907

##### Notes

Kwon (2022)

#### Cybister brevis

Aubé, 1838

2D06C90D-F282-59C5-8F37-5BEC39C6F071

##### Notes

Kwon (2022)

#### Eretes griseus

(Fabricius, 1781)

69E352FD-3161-5CF4-AF3A-9A1CC3D4CB89

##### Notes

ME (1993), Kwon et al. (1996), Lee and Jung (2001), HNIBR (2022)

#### Laccophilus difficilis

Sharp, 1873

46C2153D-FDB0-5E53-B250-2B39685BDED6

##### Notes

Lim and Lee (2012), HNIBR (2022)

#### Nebrioporus hostilis

(Sharp, 1884)

6175237D-B980-5427-BF8C-51599162D10E

##### Notes

ME (1993), Kwon et al. (1996), Lee and Jung (2001), HNIBR (2022)

#### Platambus ussuriensis

(Nilsson, 1997)

F62BF3CB-D947-547C-A053-2C436FC373FB

##### Notes

Lim and Lee (2012), HNIBR (2022)

#### Rhantus suturalis

(Macleay, 1825)

61DD2897-F46D-5855-88F8-E059CFCBD2C7

##### Notes

ME (1993), Kwon et al. (1996), Lee and Jung (2001), HNIBR (2022)

#### Elateridae

E5DB7753-6117-59BE-A52B-6258D6DBA1BA

#### Actenicerus pruinosus

Motschulsky, 1861

C233C5F4-066A-5B49-8100-96A38E0A548A

##### Notes

Lee and Jung (2001), HNIBR (2022)

#### Agrypnus binodulus coreanus

Kishii, 1961

BEDF7F75-6922-53C4-851C-8C4C892E878B

##### Notes

Lee and Jung (2001), Lim and Lee (2012), HNIBR (2022)

#### Agrypnus depressus

(Candèze, 1874)

7B901C2E-70F9-5207-AF49-8C043582B442

##### Notes

Kwon et al. (1996), Lee and Jung (2001), HNIBR (2022)

#### Agrypnus miyamotoi miyamotoi

(Nakane & Kishii, 1955)

01FCC06F-2D12-5710-BC35-F8E8343CF725

##### Notes

Lee and Jung (2001), Oh et al. (2013), HNIBR (2022)

#### Drasterius agnatus

(Candèze, 1873)

8E622D03-36E2-51B2-A232-9EA69BD4A38D

##### Notes

Kwon et al. (1996), Lee and Jung (2001), Lim and Lee (2012), HNIBR (2022), Won et al. (2023)

#### Melanotus legatus legatus

Candèze, 1860

F7334AC1-FFF8-5BC2-A2EA-F4D173282AFD

##### Notes

Kwon et al. (1996), Lee and Jung (2001), Nam et al. (2018), HNIBR (2022)

#### Pectocera fortunei

Candèze, 1873

B0A9A322-394A-5D11-AFA0-E2526722169D

##### Notes

Lim and Lee (2012), Lim et al. (2013), HNIBR (2022), Won et al. (2023)

#### Endomychidae

3960B702-EE5D-5B03-8CEC-3792B3FE5A75

#### Ancylopus melanocephalus

Olivier, 1808

642E69D3-4C52-5B5D-89E2-C15F17BD4266

##### Notes

Lee and Kwon (1981), ME (1993), Kwon et al. (1996), Lee and Jung (2001)

#### Ancylopus pictus asiaticus

(Strohecker, 1972)

57F00E5B-29E0-5FB1-B047-89A6A6EF9503

##### Notes

Kwon et al. (1996), Lee and Jung (2001), Lim et al. (2013), Nam et al. (2018), HNIBR (2022), Won et al. (2023)

#### Erotylidae

830861AA-727A-5367-BC3A-58E3914E46CC

#### Anadastus atriceps

(Crotch, 1873)

6809EFA4-60A3-51F0-92DA-F0BB42C1CC37

##### Notes

Lim et al. (2013), Nam et al. (2018)

#### Anadastus filiformis

(Fabricius, 1801)

BA4E6CB6-80C2-5E2E-A868-280866CDE573

##### Notes

Lim et al. (2013)

#### Anadastus menetriesii

(Motschulsky, 1860)

76C4D369-5D4A-5654-AA21-5B5CECD52393

##### Notes

Cho (1955), Lee and Kwon (1981), ME (1993), Kwon et al. (1996), Lee and Jung (2001), HNIBR (2022)

#### Anadastus praeustus

(Crotch, 1873)

B127510B-7AFD-5721-9C7F-7044E049FA63

##### Notes

Kwon et al. (1996), Lee and Jung (2001), Lim et al. (2013), HNIBR (2022)

#### Histeridae

72673FF4-EE3A-548B-A866-4167E619280A

#### Merohister jekeli

(Marseul, 1857)

F26D4B77-3FCC-5813-BBDB-299815B3F258

##### Notes

Lim et al. (2013)

#### Hydrophilidae

EF00792C-4C93-5685-B0A3-D2A0C1951A7C

#### Cercyon dux

Sharp, 1873

51340C20-61D6-511F-82E2-F861EA1203C1

##### Notes

ME (1993), Kwon et al. (1996), HNIBR (2022)

#### Cercyon laminatus

Sharp, 1873

BCBD650D-1B5F-5488-96ED-2AAA42DFBAC5

##### Notes

ME (1993), Kwon et al. (1996), Lee and Jung (2001), HNIBR (2022)

#### Cercyon olibrus

Sharp, 1874

2D4DE7B7-9339-5967-B25D-FC1987BF059F

##### Notes

ME (1993), Kwon et al. (1996), Lee and Jung (2001), HNIBR (2022)

#### Cercyon quisquilius

(Linnaeus, 1760)

9C845E22-1AFF-5F1B-B548-5D17DFA66CF9

##### Notes

ME (1993), Kwon et al. (1996), Lee and Jung (2001), HNIBR (2022)

#### Laccobius bedeli

Sharp, 1884

B01FDE0A-C07A-58F6-8FD6-F497C73620CE

##### Notes

ME (1993), Kwon et al. (1996), Lee and Jung (2001), HNIBR (2022)

#### Laccobius binotatus

Orchymont, 1934

B54C006F-E5FD-5F25-8E0F-28DDC2B4D408

##### Notes

Lee and Ahn (2019)

#### Laccobius oscillans

Sharp, 1884

DD6CC92E-186F-5EE5-8EFE-F3B1FB0682DC

##### Notes

ME (1993), Kwon et al. (1996), Lee and Jung (2001), HNIBR (2022)

#### Kateretidae

A79469E1-DC19-567B-9301-BF6F4DAEB86B

#### Brachypterus urticae

(Fabricius, 1792)

D872FD35-0D4E-5C6D-96F3-6007A211D19A

##### Notes

Kim (1971), Lee and Kwon (1981), ME (1993), Kwon et al. (1996), Lee and Jung (2001), HNIBR (2022)

#### Latridiidae

6E5DCD99-D45C-5BDA-87DF-BCE14A8ECC5B

#### Corticarina truncatella

(Mannerheim, 1844)

20D7F69A-E556-5DF5-8948-CEAA517F8A7C

##### Notes

Nam et al. (2018)

#### Cortinicara gibbosa

(Herbst, 1793)

BBE1CB0A-3E31-5B82-AFD4-05ABFCF28BC0

##### Notes

Lee and Jung (2001), HNIBR (2022)

#### Leiodidae

56AAE9CE-EA5E-52AE-A794-49C8ABC23091

#### Pseudcolenis hilleri

Reitter, 1884

A57C1562-4D80-5976-9921-12C9179CC6FC

##### Notes

Kwon (2022)

#### Sciodrepoides fumatus

(Spence, 1815)

161312D3-C6AE-55DE-B6A9-93FECC7D8DFE

##### Notes

Nam et al. (2018)

#### Lucanidae

62B778B2-96F6-5EB6-9EB4-7CAFC5F088BF

#### Dorcus hopei binodulosus

Waterhouse, 1874

AD168AB8-CE2B-5D3A-B7BC-E0BC0784CCAC

##### Notes

Kim and Kim (2014)

#### Dorcus rectus rectus

(Motschulsky, 1858)

98D526C3-E860-5C13-BE5F-1A010213261E

##### Notes

Cho (1955), Cho (1965), Lee and Kwon (1981), ME (1993), Kwon et al. (1996), Lee and Jung (2001), Lim and Lee (2012), Lim et al. (2013), Kim and Kim (2014), HNIBR (2022), Won et al. (2023)

#### Dorcus rubrofemoratus rubrofemoratus

(Snellen van Vollenhoven, 1865)

BEB9A365-F5A2-56B4-B411-F91C5999EA52

##### Notes

Cho (1955), Cho (1965), Lee and Kwon (1981), ME (1993), Kwon et al. (1996), Lee and Jung (2001), Lim et al. (2013), HNIBR (2022), Won et al. (2023)

#### Dorcus titanus castanicolor

(Motschulsky, 1861)

24B466EB-DDE4-547D-8DB5-F3FD54FE3137

##### Notes

Lee and Kwon (1981), ME (1993), Kwon et al. (1996), Lee and Jung (2001), HNIBR (2022)

#### Prosopocoilus inclinatus inclinatus

(Motschulsky, 1858)

7441F4AA-B23A-51D1-9D9E-E8F4847E7C2C

##### Notes

Cho (1955), Cho (1965), Lee and Kwon (1981), ME (1993), Kwon et al. (1996), Lee and Jung (2001), Lim et al. (2013), Kim and Kim (2014), HNIBR (2022)

#### Malachiidae

7A0870DB-F48B-57A5-BBE2-024FA7D73C4E

#### Attalus elongatulus

Lewis, 1895

09DE4194-7366-58AA-A7EA-A73BF8A2CA6C

##### Materials

- **Type status:** Other material. **Occurrence:** recordedBy: Lee, D. Y.; sex: 2 male; lifeStage: adult; occurrenceID: E5FC118C-0DDD-5226-8222-617E04594E32; **Location:** island: Ulleungdo Island; country: Republic of Korea; stateProvince: Gyeongsangbuk-do; county: Ulleung-gun; locality: Nari basin, Nari-ri, Bukmyeon; **Event:** samplingProtocol: Sweeping net; eventDate: 6/11/2021
- **Type status:** Other material. **Occurrence:** recordedBy: Lee, D. Y.; sex: 1male, 1 female; lifeStage: adult; occurrenceID: 2449CC06-DABE-5237-BB74-7DFB4F0CF753; **Location:** island: Ulleungdo Island; country: Republic of Korea; stateProvince: Gyeongsangbuk-do; county: Ulleung-gun; locality: Seonginbong, Dodong-ri, Ulleungeup; minimumElevationInMeters: 300; maximumElevationInMeters: 500; **Event:** samplingProtocol: Sweeping net; eventDate: 7/7/2020

##### Notes

It was previously misidentified as *Apioninae* sp. by Won et al. (2023). This is the first record of this species from Ulleungdo Island.

Distribution: Korea (National Institute of Biological Resources 2019), Japan (Asano and Kojima 2017a)

#### Megalopodidae

6B8C3484-0C32-5CDD-9B1E-992FCB52D902

#### Zeugophora bicolor

(Kraatz, 1879)

35C57CA5-E7AF-5FF3-B69B-1920C63757FD

##### Notes

Lim et al. (2013)

#### Meloidae

F499E688-63FB-55FD-A07A-2315FC631FA8

#### Epicauta flabellicornis

(Germar, 1817)

BF2D6638-2FC8-54EE-9222-46B605C0492E

##### Notes

Lee and Kwon (1981), ME (1993), Kwon et al. (1996), Lee and Jung (2001)

#### Epicauta gorhami

Lewis, 1879

66F3A866-49F8-543E-9F5A-A6A76A846A8E

##### Notes

Cho (1955), Cho (1965), Lee and Kwon (1981), ME (1993), Kwon et al. (1996), Lee and Jung (2001), HNIBR (2022)

#### Meloe auriculatus

Marseul, 1877

64AC051A-819F-5E10-8265-5B256F508986

##### Notes

Cho (1965), Lee and Kwon (1981), ME (1993), Kwon et al. (1996), Lee and Jung (2001), Lim and Lee (2012), HNIBR (2022)

#### Meloe corvinus

Marseul, 1877

A3FBD3F2-C494-5E03-81AB-EBB36E7A9E5D

##### Notes

HNIBR (2022)

#### Meloe lobatus

Gelber, 1832

A7F8288D-10EC-514D-94A3-84F227FAD648

##### Notes

Lim and Lee (2012), HNIBR (2022)

#### Meloe proscarabaeus proscarabaeus

Linnaeus, 1758

633A1585-091E-5F43-B1BC-12EE816F4213

##### Notes

Lim et al. (2013), HNIBR (2022), Won et al. (2023)

#### Zonitoschema japonica

(Pic, 1910)

96BC07FD-54D1-5D3E-BF81-19A3A28EA62E

##### Notes

Lee and Jung (2001), HNIBR (2022)

#### Melyridae

464D3DD7-C92D-5DD5-89C7-46B8C4406EF8

#### Dasytes vulgaris

Nakane, 1963

64130DDF-7DBA-5F03-9BD3-EF13CDC8E5E0

##### Materials

- **Type status:** Other material. **Occurrence:** recordedBy: Lee, D. Y.; sex: 1 male; lifeStage: adult; occurrenceID: 1CF2066B-5B0F-5D50-80B9-8F5287A45D87; **Location:** island: Ulleungdo Island; country: Republic of Korea; stateProvince: Gyeongsangbuk-do; county: Ulleung-gun; locality: Nari basin, Nari-ri, Bukmyeon; **Event:** samplingProtocol: Sweeping net; eventDate: 6/11/2021

##### Notes

This is the first record of this species from Ulleungdo Island.

Distribution: Korea (National Institute of Biological Resources 2019), Japan (Nakane 1963)

#### Mordellidae

96FEEEE8-C30E-5A1D-92C7-DC512A0F6A86

#### Falsomordellistena auromaculata

(Kôno, 1928)

8A983321-14D7-542F-BD9C-233917B76915

##### Notes

Kim (1971), Lee and Kwon (1981), ME (1993), Kwon et al. (1996), Lee and Jung (2001), HNIBR (2022)

#### Falsomordellistena hananoi

(Nomura, 1951)

7C09AE91-8CC2-5BCD-8915-BFE48B90FEC2

##### Notes

Kim (1971), Lee and Kwon (1981), ME (1993), Kwon et al. (1996), Lee and Jung (2001), HNIBR (2022)

#### Tomoxia nipponica

Kôno, 1928

67CFB9AB-5670-5E1E-A9AA-0646F49219EC

##### Materials

- **Type status:** Other material. **Occurrence:** recordedBy: Lee, D. Y.; sex: 1 female; lifeStage: adult; occurrenceID: 250C99DC-0C81-5ACF-9657-3E06773A7B2D; **Location:** island: Ulleungdo Island; country: Republic of Korea; stateProvince: Gyeongsangbuk-do; county: Ulleung-gun; locality: Seonginbong, Dodong-ri, Ulleungeup; minimumElevationInMeters: 500; maximumElevationInMeters: 700; **Event:** samplingProtocol: Sweeping net; eventDate: 7/28/2020
- **Type status:** Other material. **Occurrence:** recordedBy: Lee, D. Y.; sex: 1 female; lifeStage: adult; occurrenceID: 57D8206B-A0FA-558D-AAA2-99ECC9276EF5; **Location:** island: Ulleungdo Island; country: Republic of Korea; stateProvince: Gyeongsangbuk-do; county: Ulleung-gun; locality: Nari basin, Nari-ri, Bukmyeon; **Event:** samplingProtocol: Sweeping net; eventDate: 8/20/2021

##### Notes

This is the first record of this species from Ulleungdo Island.

Distribution: Korea (National Institute of Biological Resources 2013), Japan (Asano and Kojima 2017b)

#### Nitidulidae

C8E6FA25-8D9D-5191-9973-B2C4257E7E2D

#### Carpophilus chalybaeus

Murray, 1864

BBBC809B-FA66-5ECF-978B-19DE4B06EA13

##### Notes

Kim (1971), Lee and Kwon (1981), ME (1993), Kwon et al. (1996), Lee and Jung (2001), HNIBR (2022)

#### Epuraea oblonga

(Herbst, 1793)

1DEC4289-E9FD-5DED-8114-F8BA42050740

##### Notes

Won et al. (2023)

#### Epuraea ocularis

Fairmaire, 1849

4185882B-352D-5368-A667-5A06DECCA379

##### Notes

Nam et al. (2018), Kwon (2022)

#### Glischrochilus japonius

(Motschulsky, 1858)

EE29F6A6-E733-5D28-8D39-CE4E18241B7B

##### Notes

Cho (1955), Cho (1965), Lee and Kwon (1981), ME (1993), Kwon et al. (1996), Lee and Jung (2001), HNIBR (2022)

#### Glischrochilus pantherinus

(Reitter, 1879)

272F40C3-BF48-5AE5-B185-0CD2C671B0E6

##### Notes

Lee and Jung (2001)

#### Glischrochilus rufiventris

(Reitter, 1879)

E51CA70F-0C76-5689-907F-B472CD05A4DB

##### Notes

Won et al. (2023)

#### Ipidia variolosa variolosa

Reitter, 1879

0F0D62CF-58E4-56CA-AC54-66DC60356874

##### Notes

Won et al. (2023)

#### Librodor rufiventris

(Reitter, 1879)

3F2626E1-FB32-511D-9E93-4AA66958DBE1

##### Notes

Kwon et al. (1996), Lee and Jung (2001)

#### Meligethes flavicollis

Reitter, 1873

78604F0C-6B0A-5FD8-B8BE-4FA76401E05A

##### Notes

Won et al. (2023)

#### Neopallodes omogonis

Hisamatsu, 1953

235C0568-C8A3-54E4-89AA-2EED0793A9D1

##### Notes

Won et al. (2023)

#### Omosita discoidea

(Fabricius, 1775)

E45C02E0-0673-5F25-8616-6145482C4245

##### Notes

HNIBR (2022), Won et al. (2023)

#### Omosita japonica

Reitter, 1874

A61AB8A8-1F49-5829-A35D-751006D317D6

##### Notes

HNIBR (2022)

#### Noteridae

005A90A0-BFC0-5F6B-93B2-DE31C4E2B4DF

#### Noterus japonicus

Sharp, 1873

3F999EE2-9B59-58EC-9703-EAF757393E03

##### Notes

Lee and Ahn (2019)

#### Oedemeridae

01B498F1-D346-5854-B34E-9F7B201617F4

#### Eobia chinensis

(Hope, 1843)

8F0AFE3C-C4E4-5FB7-B3CC-FBE5CBC7918E

##### Notes

Cho (1965), Lee and Kwon (1981), ME (1993), Kwon et al. (1996), Lee and Jung (2001), HNIBR (2022)

#### Nacerdes hilleri hilleri

(Harold, 1878)

CBE4D7DF-FBDF-54AB-A837-2915C9620464

##### Notes

Lee and Jung (2001), HNIBR (2022)

#### Nacerdes luteipennis

(Marseul, 1877)

E174D423-AF1D-5FF9-B942-714007C4C198

##### Notes

Kim (1971), Lee and Kwon (1981), ME (1993), Kwon et al. (1996), Lee and Jung (2001), HNIBR (2022)

#### Nacerdes melanura

(Linnaeus, 1758)

0FF1E5BB-4DAC-561F-AB88-C2D962DA81BC

##### Notes

Oh et al. (2013), HNIBR (2022)

#### Pyrochroidae

F7EA3042-F8FA-509D-A692-5D728A6018AF

#### Pseudopyrochroa laticollis

(Lewis, 1887)

8BA16CED-7AF5-5164-854E-D4B36D4325FC

##### Notes

Byun et al. (1996), Kwon et al. (1996), Lee and Jung (2001), HNIBR (2022)

#### Schizotus fuscicollis

(Mannerheim, 1852)

B1C61812-61DE-5912-AD4D-C81A9AFB18AD

##### Notes

Lee and Jung (2001), HNIBR (2022)

#### Salpingidae

5260E79D-9CD2-5374-8D0E-A874401CB07D

#### Elacatis kraatzi

Reitter, 1879

C1A2254E-792D-5611-BC4D-C0B05A8FDE8B

##### Materials

- **Type status:** Other material. **Occurrence:** recordedBy: Lee, D. Y.; sex: 1 male; lifeStage: adult; occurrenceID: 47C7E282-F04F-5F91-990C-FCEA7A2586CA; **Location:** island: Ulleungdo Island; country: Republic of Korea; stateProvince: Gyeongsangbuk-do; county: Ulleung-gun; locality: Seonginbong, Dodong-ri, Ulleungeup; **Event:** samplingProtocol: Sweeping net; eventDate: 6/5/2020

##### Notes

It was previously misidentified as *Salpingus* sp. by Won et al. (2023). This is the first record of this species from Ulleungdo Island.

Distribution: Korea, Japan, Russia (Kim 2018)

#### Salpingus depressifrons

Nikitsky and Belov, 1983

0986972E-9FF2-579D-A18C-0529172FFDF5

##### Notes

Won et al. (2023)

#### Scarabaeidae

278A1195-D486-5341-A1D1-4C4D28376464

#### Adoretus tenuimaculatus

Waterhouse, 1875

76D2EA6A-0ACA-5A3A-BF14-84263FC5AE86

##### Notes

Lim et al. (2013)

#### Agrilinus uniformis

(Waterhouse, 1875)

864729EA-BB4F-5171-83D1-F5DA07E8C933

##### Notes

Kim (2012)

#### Anomala albopilosa

(Hope, 1839)

04B4E234-3774-52F3-A0C1-AA69C078C4D6

##### Notes

Cho (1955), Cho (1965), Lee and Kwon (1981), ME (1993), Kwon et al. (1996), Lee and Jung (2001), Kim (2011), Lim et al. (2013), HNIBR (2022)

#### Anomala aulax

(Wiedemann, 1823)

2DE0A6A2-3C73-58D3-B4C3-3F67369D8480

##### Notes

Kim (2011), HNIBR (2022)

#### Anomala cuprea

(Hope, 1839)

329D47A1-DFD0-5B7F-8757-DD902B6B4EA9

##### Notes

Lee and Kwon (1981), ME (1993), Kwon et al. (1996), Lee and Jung (2001), HNIBR (2022)

#### Anomala luculenta

Erichson, 1847

07508AF3-AA79-5E8B-961B-418B3F99F2DE

##### Notes

Kim (2011)

#### Anomala mongolica

(Faldermann, 1835)

7B64279C-869F-5F9C-A3F4-AAD115877F98

##### Notes

Kim (2011)

#### Anomala orientalis

(Waterhouse, 1875)

F9C48864-9932-5C41-8125-6DBE3F9621A0

##### Notes

Won et al. (2023)

#### Anomala viridana

(Kolbe, 1886)

50106594-8E31-5A35-BAF1-C9BBD01326CC

##### Notes

Lee and Jung (2001), Kim (2011), HNIBR (2022)

#### Anthracophora rusticola

Burmister, 1842

D356C4C0-D1AE-5D80-B071-FD852F7CF178

##### Notes

Lee and Kwon (1981), ME (1993), Kwon et al. (1996), Lee and Jung (2001), HNIBR (2022)

#### Aphodius sublimbatus

(Motschulsky, 1860)

24997F7B-ABD7-5215-8F45-C467779F8C77

##### Notes

Kim (2012)

#### Bodilopsis sordidus

(Fabricius, 1775)

694DE962-FF1C-54A4-A4B1-02B31FBA5260

##### Notes

Kim (2012)

#### Caccobius sordidus

Harold, 1886

21AD5A3D-EE9F-5FE4-829D-3C6D7EA8B952

##### Notes

Kim (2012)

#### Colobopterus quadratus

(Reiche, 1850)

43393D41-40FA-5D55-9A94-6350E725620E

##### Notes

Kim (2012)

#### Gametis jucunda

(Faldermann, 1835)

44C7E789-986C-5ACF-8639-9E63373146E9

##### Notes

Lim et al. (2013)

#### Heptophylla picea

Motschulsky, 1857

BFA18FC0-8B54-5439-8E63-4B6C33B22802

##### Notes

Kwon (2022)

#### Holotrichia diomphalia

(Bates, 1888)

5A5E1C35-6493-5115-9115-7D40B3588870

##### Notes

Kwon (2022)

#### Holotrichia parallela

(Motschulsky, 1854)

AFCF6FF2-FD3A-5D8D-AA40-EB51219E7382

##### Notes

Nam et al. (2018), Info (2023)

#### Holotrichia reticulata

Murayama, 1941

559C863D-D94D-5DE3-9895-77E6B5022423

##### Notes

Kim (2011), HNIBR (2022)

#### Lasiotrichius succinctus

(Pallas, 1781)

226904CB-F634-55FB-BFD1-6DA1E1F28748

##### Notes

Kwon (2022)

#### Maladera formosae

(Brenske, 1898)

C26CB2A4-1D9C-5A49-86C0-FC5A2DF86097

##### Notes

Nam et al. (2018)

#### Maladera verticalis

(Fairmaire, 1888)

52D8F3A1-B1C3-5060-82AE-08CFACE64ACA

##### Notes

Kwon (2022)

#### Mimela splendens

(Gyllenhal, 1817)

415FD27D-DBCE-5C40-A3AD-CD14E403D11F

##### Notes

Kwon (2022)

#### Mimela testaceipes

(Motschulsky, 1860)

C34DE98A-0E42-5A79-982D-9FF94C3B4B09

##### Notes

Kim (2011)

#### Onthophagus japonicus

Harold, 1875

E35DD5B2-FA92-5AE8-A482-73B25D656193

##### Notes

Kim (2012)

#### Onthophagus viduus

Harold, 1875

3873221D-25A1-549C-8FC8-C91D0942E7D3

##### Notes

Kim (2012)

#### Phaeaphodius rectus

(Motschulsky, 1866)

270928E7-3E9A-594B-9840-D36E2A9E6A98

##### Notes

Kim (2012)

#### Planolinoides borealis

(Gyllenhal, 1827)

FCEADCA5-A278-5B19-9470-039C254D769C

##### Notes

Kim (2012)

#### Popillia mutans

Newman, 1838

937D7DDE-BB8A-5BCD-B5DA-F12ED377E5A8

##### Notes

Lee and Kwon (1981), ME (1993), Kwon et al. (1996), Lee and Jung (2001), Lim et al. (2013), HNIBR (2022)

#### Protaetia brevitarsis

seulensis (Kolbe, 1886)

931E8317-13EB-51B9-9E37-8880DF2BA10D

##### Notes

Cho (1965), Lee and Kwon (1981), ME (1993), Kwon et al. (1996), Lee and Jung (2001), Lim and Lee (2012), HNIBR (2022)

#### Protaetia lugubris

(Herbst, 1786)

1B8EEC22-F673-5891-9DBC-16C063F87AB0

##### Notes

Kim (2011), HNIBR (2022), Won et al. (2023)

#### Protaetia orientalis submarmorea

(Burmeister, 1842)

2CA8CE7F-8391-52B5-B129-5DD32F95EE1A

##### Notes

Kim (1971), Lee and Kwon (1981), ME (1993), Kwon et al. (1996), Lee and Jung (2001), HNIBR (2022)

#### Serica fulvopubens

(Reitter, 1896)

AF0118F3-1F1E-59B1-903A-D43A8A475C65

##### Notes

Kwon (2022)

#### Sericania fuscolineata

Motschulsky, 1860

144C2E6F-24AE-5841-B542-B59CDDB714F8

##### Notes

HNIBR (2022), Kwon (2022), Won et al. (2023)

#### Sericania latisulcata

Murayama, 1941

63971EC5-4A57-5FF3-8AE9-2B7266C71A77

##### Notes

Kwon et al. (1996), Lee and Jung (2001), Kim (2011), HNIBR (2022)

#### Sophrops striatus

(Brenske, 1892)

C1F7E19C-E0E9-52CA-A0B3-943EEE4F01DB

##### Notes

Won et al. (2023)

#### Silvanidae

D7229D37-B139-53D3-A84C-3D8D734FDDF9

#### Psammoecus triguttatus

Reitter, 1874

C4C27A26-F71F-5F06-8D1F-CCFB31B3A7E8

##### Notes

Lee and Jung (2001), HNIBR (2022)

#### Uleiota arboreus

(Reitter, 1889)

8C946F11-5937-5262-8A93-96ADE4B51F63

##### Notes

Won et al. (2023)

#### Staphylinidae

C4AFDFAA-8E2E-5D45-B3F7-38188839E268

#### Agelosus weisei

(Harold, 1877)

AEB46852-7C13-5414-BE57-537A464B8BDD

##### Notes

Lim and Lee (2012), HNIBR (2022)

#### Aleochara coreana

Bernhauer, 1926

C4809052-8167-590F-9729-D3D8403F01B0

##### Notes

HNIBR (2022)

#### Aleochara curtula

(Goeze, 1777)

9CBFA2FA-3ADB-5B96-95EB-82453818A751

##### Notes

Lee and Jung (2001), Oh et al. (2013), HNIBR (2022), Won et al. (2023)

#### Aleochara lata

Gravenhorst, 1802

2CEA35F9-948F-5C2C-AD18-0517245A574C

##### Notes

HNIBR (2022)

#### Aleochara parens

Sharp, 1874

790FA92A-2081-5296-A18F-FC477E10732C

##### Notes

HNIBR (2022)

#### Algon sphaericollis

Schillhammer, 2006

8FA8CC2B-9DD2-5FA5-AD45-A68A5010C48B

##### Notes

Lim et al. (2013), Oh et al. (2013), Cho (2019), HNIBR (2022)

#### Anotylus lewisius

(Sharp, 1874)

8830FC4E-5EFE-5DF1-98D9-3090A7F1E776

##### Notes

HNIBR (2022)

#### Astenus porosus

(Sharp, 1889)

E08D7E72-672C-5E5D-B0CB-C14E06DC4F4B

##### Notes

Oh et al. (2013), HNIBR (2022)

#### Astenus suffusus

(Sharp, 1874)

A44E4A82-0C3B-5EC8-9449-7B033D16CADF

##### Notes

Oh et al. (2013), Nam et al. (2018), HNIBR (2022)

#### Aulacocypus parvulus

(Sharp, 1874)

6A7F0B7F-C6D8-5602-B278-AE42A00E4711

##### Notes

Nam et al. (2018)

#### Baeocera freyi

Löbl, 1966

19BED22C-CB8A-55F0-B31D-86BBE44416FC

##### Notes

Info and Park (2023)

#### Bisnius parcus

(Sharp, 1874)

A3154912-1520-57F0-8CA9-D7CC2004469D

##### Notes

Cho (2014), HNIBR (2022)

#### Bolitobius parasetiger

Schülke, 1993

DF64637D-5638-5E6F-88A7-9DC5DA6764A4

##### Notes

Nam et al. (2018)

#### Creophilus maxillosus maxillosus

(Linnaeus, 1758)

E2FF2934-CAC1-52B2-B6B5-12D79EEA0713

##### Notes

Cho (1955), Cho (1965), Lee and Kwon (1981), ME (1993), Kwon et al. (1996), Lee and Jung (2001), HNIBR (2022)

#### Diochus japonicus

Cameron, 1930

26745795-D4ED-5982-8A56-2DF6B696A1FD

##### Notes

Oh et al. (2013), HNIBR (2022)

#### Domene chenpengi

Li, 1990

30DFA050-A729-5B3F-BA9B-EB7A6FC7B07C

##### Notes

Oh et al. (2013), HNIBR (2022)

#### Falagria caesa

Erichson, 1837

2DEA73FF-B707-5474-968F-435F5CBA9BC8

##### Notes

HNIBR (2022)

#### Gabrius ophion

Smetana, 1984

2A44A9A5-B8FE-5131-A5B5-170AD96CED26

##### Notes

Cho (2014), HNIBR (2022)

#### Gabrius praesignis

Schillhammer, 2001

8EF6DB3B-BD63-5C99-9BF6-E3AF5E00F2C8

##### Notes

Cho (2014), HNIBR (2022)

#### Indoquedius praeditus

(Sharp, 1889)

0A249605-6404-52C2-8B01-334477C677DD

##### Notes

Nam et al. (2018)

#### Isocheilus staphylinoides

(Kraatz, 1859)

BF910879-1A0E-56A1-B84C-1199F9F1C2B4

##### Notes

HNIBR (2022)

#### Lithocharis nigriceps

Kraatz, 1859

37E98DDB-AA26-58CB-B1F0-9ADBEEA64213

##### Notes

Nam et al. (2018), HNIBR (2022)

#### Lithocharis uvida

Kraatz, 1859

0A9B678C-AA18-5CC6-A3A0-7C4439192CC0

##### Notes

HNIBR (2022)

#### Megarthrus aino

Cuccodoro, 1996

4405BBFA-5188-548B-B182-AA91BA5E7F53

##### Notes

HNIBR (2022)

#### Micropeplus fulvus japonicus

Sharp, 1874

6795D545-DC4A-5B88-9A37-84A0F69577AB

##### Notes

HNIBR (2022)

#### Myrmecocephalus sapidus

(Sharp, 1874)

0FB0AFBD-B2F9-5FD2-A2D6-6E9DE16EE3EF

##### Notes

HNIBR (2022)

#### Necrodes littoralis

(Linnaeus, 1758)

25BCC22A-FC3F-5B6B-958F-3DE91FD1463B

##### Notes

Lim and Lee (2012), Lim et al. (2013), HNIBR (2022)

#### Necrophila brunneicollis brunneicollis

(Kraatz, 1877)

60B49D20-17F9-51E0-A853-6AA6D8359A39

##### Notes

Lee and Kwon (1981), ME (1993), Kwon et al. (1996), Lee and Jung (2001), HNIBR (2022)

#### Necrophila jakowlewi jakowlewi

(Semenov, 1891)

D36250B7-B31E-5B9A-8C3A-1880290BA68E

##### Notes

Cho (1955), Cho (1965), Kim (1971), Lee and Kwon (1981), ME (1993), Kwon et al. (1996), Lee and Jung (2001), Lim and Lee (2012), Lim et al. (2013), Oh et al. (2013), HNIBR (2022), Won et al. (2023)

#### Nehemitropia lividipennis

(Mannerheim, 1830)

AC0067AC-9D53-5528-8888-4BAB7EA33B80

##### Notes

HNIBR (2022)

#### Nicrophorus maculifrons

(Kraatz, 1877)

2B38A574-51DF-588C-ACD8-7EC403324E77

##### Notes

Kwon et al. (1996), Lee and Jung (2001), Kim and Kim (2013), HNIBR (2022)

#### Nicrophorus quadripunctatus

(Kraatz, 1877)

5493C634-10A3-5F25-B16C-3E681A9319FA

##### Notes

Lim et al. (2013)

#### Nudobius pleuralis

(Sharp, 1874)

AE4891FD-7BA6-5E64-881F-6CF700ACED28

##### Notes

Cho (2019)

#### Ochthephilum densipenne

(Sharp, 1889)

2966B31F-A27D-5C78-BE7E-38C8ADA77A3E

##### Notes

Lee and Jung (2001), Oh et al. (2013), Nam et al. (2018), HNIBR (2022)

#### Osorius taurus taurus

Sharp, 1889

16092428-7579-5D36-82B4-2CF9C8FEE7BC

##### Notes

HNIBR (2022)

#### Othius medius

Sharp, 1874

2E9BD094-07D7-51CF-AB26-86ED4D047486

##### Notes

Cho (2019)

#### Paederus fuscipes fuscipes

Curtis, 1826

38A83969-47A2-5228-8BF9-8734D648820B

##### Notes

Lee and Kwon (1981), ME (1993), Kwon et al. (1996), Lee and Jung (2001), HNIBR (2022)

#### Pelioptera opaca

Kraatz, 1857

495C0C46-9BAF-5AD3-BE51-C6F99D1B2B43

##### Notes

HNIBR (2022)

#### Pella coreana

Maruyama, 2006

CE95127C-2E18-52B8-86BA-77D858AB0EB8

##### Notes

Nam et al. (2018)

#### Phacophallus japonicus

(Cameron, 1933)

C1926F83-7D33-5FE3-93ED-3D1BC9DE6A3B

##### Notes

HNIBR (2022)

#### Philonthus addendus

Sharp, 1867

0B10A179-5199-5511-A49A-6E4264FD2F68

##### Notes

Nam et al. (2018)

#### Philonthus japonicus

Sharp, 1874

0F5742F0-51BA-5B99-B096-5A9BDDDD4BA4

##### Notes

Cho (2014), HNIBR (2022)

#### Philonthus lewisius

Sharp, 1874

C38D866D-1D1B-5E34-B605-A6F2552AA417

##### Notes

HNIBR (2022), NSMK (2023a)

#### Philonthus longicornis

Stephens, 1832

E1E29FD6-B93B-5620-BCC0-9387EA759BD5

##### Notes

Cho (2014), HNIBR (2022), Kwon (2022)

#### Philonthus minutus

Boheman, 1848

20FDBE5A-C392-5BFF-82C2-57507A58DBC8

##### Notes

HNIBR (2022)

#### Philonthus ohizumi

R. Dvořák, 1958

B77E6EE1-09D1-54A0-8431-D5D1F547315F

##### Notes

Cho (2014), HNIBR (2022)

#### Philonthus rectangulus

Sharp, 1874

A0B5B68B-C9C8-5C6D-8F81-E71AC7FF9529

##### Notes

HNIBR (2022)

#### Philonthus spinipes spinipes

Sharp, 1874

660B8F91-547C-5BF0-AF20-081CDF911B32

##### Notes

HNIBR (2022)

#### Philonthus sublucanus

Herman, 2001

D67560B9-E50B-5478-B89E-DC9106C2F684

##### Notes

Cho (2014), HNIBR (2022)

#### Philonthus tardus

Kraatz, 1859

20302036-A4AB-568B-AD32-490A2CA96E4D

##### Notes

Lee and Jung (2001), HNIBR (2022)

#### Philonthus wuesthoffi

Bernhauer, 1939

9384C915-E27A-5084-8ADD-151E49C52C15

##### Notes

HNIBR (2022)

#### Rugilus longipennis

(Sharp, 1889)

CF128FC6-BFAC-500F-B039-9EC311C50037

##### Notes

HNIBR (2022)

#### Rhomphocallus coreanus

Assing, 2011

E8712FC6-50C8-5614-A185-B78658476FA2

##### Notes

Assing (2011)

#### Scopaeus currax

Sharp, 1889

86AF0774-0F5E-5AE2-9296-1BFFE1636C19

##### Notes

Oh et al. (2013), HNIBR (2022)

#### Stenus distans

Sharp, 1889

A4C495D7-34D0-53F8-8ED2-8F34BCA70F45

##### Notes

Oh et al. (2013), HNIBR (2022)

#### Stenus rugipennis

Sharp, 1874

94C07655-C74B-5D78-8D0C-74D6DC6CE46A

##### Notes

Oh et al. (2013), HNIBR (2022)

#### Thanatophilus rugosus

(Linnaeus, 1758)

BBF4E90F-3C9B-5315-9B0B-4D5CFB85D1A6

##### Notes

Info (2023)

#### Thanatophilus sinuatus

(Fabricius, 1775)

742AA112-2541-5BEB-BD18-29A6E200DDF4

##### Notes

Lee and Kwon (1981), ME (1993), Kwon et al. (1996), Lee and Jung (2001)

#### Tenebrionidae

9EE9785B-58C8-5605-B147-D7DD918A65DC

#### Allecula ussuriensis

Borchmann, 1937

BD1E0110-9B84-5F4E-9873-9E7A07F8135A

##### Notes

Jung (2012b)

#### Diaperis lewisi lewisi

Bates, 1873

6341D210-40E9-5F08-8AA6-D8481CFBC08D

##### Notes

Kwon (2022)

#### Gonocephalum coenosum

Kaszab, 1952

B8571D73-CB27-597B-8FE8-CB5EEDE30A7D

##### Notes

Lim and Lee 2012, Jung 2012a, HNIBR 2022

#### Gonocephalum japanum

(Motschulsky, 1861)

B33E7949-1C01-53EB-B2D4-E2E38C298FF6

##### Notes

Cho (1955), Cho (1965), Lee and Kwon (1981), ME (1993), Kwon et al. (1996), Lee and Jung (2001), HNIBR (2022)

#### Gonocephalum persimile

(Lewis, 1894)

E7F0154A-AF3C-59A4-84AE-DAABCCD4AC12

##### Notes

Nam et al. (2018)

#### Gonocephalum pubens

(Marseul, 1876)

F98EE9C5-591D-52DF-99A3-1016FAC228D9

##### Notes

Lee and Jung (2001), HNIBR (2022), Won et al. (2023)

#### Lagria nigricollis

Hope, 1843

2689EDA6-5A52-599F-B387-B37B660721ED

##### Notes

Cho (1955), Cho (1965), Lee and Kwon (1981), ME (1993), Kwon et al. (1996), Lee and Jung (2001), Lim et al. (2013), Won et al. (2023)

#### Lagria rufipennis

Marseul, 1876

E5A80AA0-D48E-5C20-AC7D-A47F8BFC2A0E

##### Notes

Oh et al. (2013), Won et al. (2023)

#### Luprops orientalis

(Motschulsky, 1868)

2C6E2859-8EFE-581D-805E-24A2CC425DC6

##### Notes

Oh et al. (2013), HNIBR (2022), Won et al. (2023)

#### Mycetochara orientalis

Dubrovin, 1992

9ECAE4B6-7547-5202-B16C-09BBB092B657

##### Notes

Won et al. (2023)

#### Opatrum subaratum

(Faldermann, 1835)

B05552E6-BE78-5420-909E-47B58FC22E00

##### Notes

Lim and Lee (2012)

#### Uloma latimanus

Kolbe, 1886

354AEC8C-8A52-5D59-8EAD-7F5677D90416

##### Notes

Lim and Lee (2012), HNIBR (2022)

#### Upinella hirokii

(Akita & Masumoto, 2012)

6A0FEEA9-0145-54B7-897D-A6A2B161E5EC

##### Notes

Won et al. (2023)

#### Trogossitidae

25B5D4B2-692C-5A25-A734-B64A7BC03BD0

#### Leperina squamulosa

(Gebler, 1830)

577BE267-5DD0-5495-80E6-8748BAEA54A2

##### Notes

Nam et al. (2018)

#### Tenebroides mauritanicus

(Linnaeus, 1758)

097506D7-885C-5B1B-8093-49099D27E735

##### Notes

Nam et al. (2018)

#### Thymalus parviceps

Lewis, 1894

6334E66F-DC41-5843-80DA-4D31A87DD68F

##### Notes

Kwon et al. (1996), Lee and Jung (2001)

#### Zopheridae

8FBF3992-8FB1-5182-A792-7CF0D3C55CDB

#### Phellopsis suberea

Lewis, 1887

A1BD3593-9CE6-5FD2-9A4C-C420BFE95F52

##### Notes

Cho (1955), Cho (1965), Lee and Kwon (1981), ME (1993), Kwon et al. (1996), Lee and Jung (2001), HNIBR (2022)

#### Dermaptera

B33AB84C-0177-50B5-AC06-51F7AF3CF56B

#### Anisolabididae

106B3546-84A7-5859-9799-4ACFBE6AC251

#### Anisolabella marginalis

(Dohrn, 1864)

51312198-1C30-5161-9C21-0BDD81C34F66

##### Notes

Lee and Jung (2001), Nam et al. (2018), Won et al. (2023)

#### Anisolabis maritima

(Bonelli, 1832)

7622780C-B0E2-5442-9E43-9CD8FA99A36E

##### Notes

Cho (1955), Cho (1965), Lee and Kwon (1981), ME (1993), Kwon et al. (1996), Lee and Jung (2001), Lim et al. (2013)

#### Euborellia annulata

(Fabricius, 1793)

65A10F7E-1CFB-5204-87D8-BC19153E5D52

##### Notes

Won et al. (2023)

#### Euborellia annulipes

(Lucas, 1847)

0E13E681-1307-5D2B-AD29-310E582EBBF4

##### Notes

Nam et al. (2018)

#### Forficulidae

C996EFBF-345B-5896-9F0B-47E237AE02CB

#### Anechura harmandi

(Burr, 1904)

0C41497B-7222-5244-8095-47EA59C11772

##### Notes

Lee and Jung (2001)

#### Anechura japonica

(De Bormans, 1880)

A2760776-74DC-50ED-AAD4-09AF7933F9A5

##### Notes

Cho (1955), Cho (1965), Lee and Kwon (1981), ME (1993), Kwon et al. (1996), Lee and Jung (2001), Lim and Lee (2012), Lim et al. (2013), Nam et al. (2018), HNIBR (2022), Won et al. (2023)

#### Forficula scudderii

De Bormans, 1880

935043AC-41A3-52CF-9F6B-4A9EC7F65588

##### Notes

Lee and Jung (2001)

#### Labiduridae

50675551-1B70-5BC5-AB92-1B2611309B1B

#### Labidura riparia japonica

(De Haan, 1842)

BCA97B1C-50FC-572C-9324-CFDCB8E6CDCE

##### Notes

Lee and Jung (2001), Lim and Lee (2012), HNIBR (2022)

#### Diptera

968C1AF2-CC67-5CAA-B12D-295FD3B8989E

#### Anisopodidae

90FA1F93-60C2-538A-8EBB-7C445F2D5D9A

#### Sylvicola japonicus

(Matsumura, 1915)

F86D42AD-32D8-514D-899C-35B30AAC2744

##### Notes

Won et al. (2023)

#### Anthomyiidae

6C7434EA-89A6-517F-A3BD-9BF26B354BC0

#### Anthomyia illocata

Walker, 1856

E349911D-8B26-53CA-A6FE-80D39A84F1F1

##### Notes

Lee and Kwon (1981), ME (1993), Kwon et al. (1996), Lee and Jung (2001), Lim et al. (2013), Nam et al. (2018), Suh (2019), HNIBR (2022)

#### Anthomyia oculifera

Bigot, 1885

AE70E602-56AD-5916-B59C-23067385EEE3

##### Notes

Kwon et al. (1996), Lee and Jung (2001), HNIBR (2022)

#### Anthomyia plumiseta

Stein, 1918

E943109A-EFF8-5D13-80D4-A446EA8843DD

##### Notes

Suh (2019)

#### Delia antiqua

(Meigen, 1826)

F0A1A4B6-4B75-509D-AC0D-2A1DEAD01BB2

##### Notes

Kwon et al. (1996), Lee and Jung (2001)

#### Delia platura

(Meigen, 1826)

DE44AC89-5CC7-5628-BD8B-4943C28B337F

##### Notes

Kwon et al. (1996), Lee and Jung (2001), Suh (2019), HNIBR (2022), Won et al. (2023)

#### Emmesomyia hasegawai

Suwa, 1979

EF782CDE-44C2-5E3D-9CA5-3B7F24A74CCC

##### Notes

Kwon (2022)

#### Fucellia apicalis

Kertestz, 1908

91887D3C-AE6E-5B57-947B-927AF428A3DA

##### Notes

Kwon et al. (1996), Lee and Jung (2001), Suh (2019), Won et al. (2023)

#### Leucophora sponsa

(Meigen, 1826)

A7C4B19F-5728-5F0D-8FB4-97EDBF1F6542

##### Notes

Suh (2019), HNIBR (2022)

#### Pegomya cunicularia

(Rondani, 1866)

F6FADC0C-FEEA-5AA7-AE2A-D4F44193BB7A

##### Notes

Suh (2019)

#### Asilidae

28683E11-E6C4-54E7-BC0A-1F075DBBB78A

#### Choerades japonicus

(Matsumura, 1931)

6F2A22DC-F629-5479-92F9-90B3CD724011

##### Notes

Kim (1971), Lee and Kwon (1981), ME (1993), Kwon et al. (1996), Lee and Jung (2001)

#### Cophinopoda chinensis

(Fabricius, 1794)

917AAFA9-E3B0-509D-A227-CA2AD8AFCCD6

##### Notes

Lim et al. (2013)

#### Neoitamus angusticornis

(Loew, 1858)

24150FDA-4324-5936-BE5C-455343201B6D

##### Notes

Kwon (2022)

#### Neoitamus cothurnatus univittatus

(Loew, 1871)

220A7FEF-478F-5CCA-A916-798D33908477

##### Materials

- **Type status:** Other material. **Occurrence:** recordedBy: Lee, D. Y.; sex: 1 male; lifeStage: adult; occurrenceID: 281D2747-D5D1-5CCA-90A0-DCC5DCB082BD; **Location:** island: Ulleungdo Island; country: Republic of Korea; stateProvince: Gyeongsangbuk-do; county: Ulleung-gun; locality: Nari basin, Nari-ri, Bukmyeon; **Event:** samplingProtocol: Sweeping net; eventDate: 8/16/2021

##### Notes

This is the first record of this species from Ulleungdo Island.

Distribution: Austria, Denmark, England, Finland, France, Italy, Netherlands, Romania, Russia, Sweden (Lehr 1998), Japan, Kazakhstan, Mongolia (Astakhov et al. 2019), Korea (National Institute of Biological Resources 2019)

#### Trichomachimus scutellaris

(Coquillett, 1898)

911C826D-3F9B-5A51-8705-AF1799E44A0C

##### Notes

Kwon (2022)

#### Bibionidae

77F237DB-CCA0-5479-A46D-A3561DBB3091

#### Bibio holomaurus

Hardy & Takahashi, 1960

7F250430-FDC1-5E05-A4B6-A79838F6D754

##### Notes

Kwon (2022)

#### Bibio tenebrosus

Coquillett, 1898

FF3BF09B-9F99-53D8-B491-19A4A415C526

##### Notes

Won et al. (2023)

#### Bombyliidae

783854FE-E0FF-50FB-9BBE-C6BD9D70835F

#### Bombylius major

Linnaeus, 1758

3E5BFDFC-4E77-5556-9639-9E0E7FCC68E9

##### Notes

Lim and Lee (2012)

#### Villa limbata

(Coquillett, 1898)

A4BA575B-FD4B-578D-8357-330D691CF5C6

##### Notes

Cho (1955), Cho (1965), Lee and Kwon (1981), ME (1993), Kwon et al. (1996), Lee and Jung (2001)

#### Calliphoridae

FA2C1D02-A83C-58B3-8C0B-57A5C5687973

#### Calliphora calliphoroides

(Rohdendorf, 1931)

51A255EC-45CA-58B9-BE5D-A4B885159D79

##### Notes

Lee and Kwon (1981), ME (1993), Kwon et al. (1996), Lee and Jung (2001)

#### Calliphora grahami

Aldrich, 1930

9FD42480-709A-55B0-B27D-E842F3564A90

##### Notes

Park and Kano (1963), Lee and Kwon (1981), ME (1993), Kwon et al. (1996), Lee and Jung (2001), Nam et al. (2018), HNIBR (2022)

#### Calliphora lata

Coquillett, 1898

04828175-93D6-5C40-A835-6478A3AFCE48

##### Notes

Cho (1955), Cho (1965), Lee and Kwon (1981), ME (1993), Kwon et al. (1996), Lee and Jung (2001), HNIBR (2022)

#### Calliphora vicina

Robineau-Desvoidy, 1830

ED8561E2-CB3F-521C-BFD4-A89E9B0B9630

##### Notes

Kwon (2022)

#### Calliphora vomitoria

(Linnaeus, 1758)

D837C556-93E9-5B2D-B4FB-FD8C11299C49

##### Notes

Lim et al. (2013)

#### Chrysomya megacephala

(Fabricius, 1794)

4F6CFB0D-F279-5E4A-9473-38AB2F07C010

##### Notes

Lee and Kwon (1981), ME (1993), Kwon et al. (1996), Lee and Jung (2001), HNIBR (2022)

#### Chrysomya pinguis

(Walker, 1858)

37FD8DAE-758B-5D14-B63A-A30C36DCE1FD

##### Notes

Park and Kano (1963), Lee and Kwon (1981), ME (1993), Kwon et al. (1996), Lee and Jung (2001)

#### Hemipyrellia ligurriens

(Wiedemann, 1830)

9835ED6C-2CFF-5853-B453-E7786182100D

##### Notes

Park and Kano (1963), Lee and Kwon (1981), ME (1993), Kwon et al. (1996), Lee and Jung (2001)

#### Lucilia ampullacea

Villeneuve, 1922

DE2EF740-4B26-59F9-9E11-9B31C051C5E1

##### Notes

Lee and Kwon (1981), ME (1993), Kwon et al. (1996), Lee and Jung (2001)

#### Lucilia caesar

(Linnaeus, 1758)

1EE4B9A0-F2AE-5A30-8E43-25D0D167DB86

##### Notes

Cho (1955), Cho (1965), Lee and Kwon (1981), ME (1993), Kwon et al. (1996), Lee and Jung (2001), Lim et al. (2013), Nam et al. (2018), HNIBR (2022)

#### Lucilia cuprina

(Wiedemann, 1830)

9D2E568B-55E0-589B-B174-2802A990850A

##### Notes

Lee and Kwon (1981), ME (1993), Kwon et al. (1996), Lee and Jung (2001), HNIBR (2022)

#### Lucilia illustris

(Meigen, 1826)

EB982735-9D5D-56CB-87B9-5248FBBEAC5C

##### Notes

Park and Kano (1963), Lee and Kwon (1981), ME (1993), Kwon et al. (1996), Lee and Jung (2001), HNIBR (2022)

#### Lucilia papuensis

Macquart, 1843

277ADF7F-CE1F-596B-994B-240C2263C27E

##### Notes

Lee and Kwon (1981), ME (1993), Kwon et al. (1996), Lee and Jung (2001), HNIBR (2022)

#### Lucilia porphyrina

(Walker, 1856)

E666736D-6B95-5DB9-8D85-0E1EC077AB8A

##### Notes

Park and Kano (1963), Lee and Kwon (1981), ME (1993), Kwon et al. (1996), Lee and Jung (2001)

#### Lucilia sericata

(Meigen, 1826)

73D07FC7-7A43-56C2-A997-ACC572055F9C

##### Notes

Park and Kano (1963), Lee and Kwon (1981), ME (1993), Kwon et al. (1996), Lee and Jung (2001), HNIBR (2022)

#### Stomorhina obsoleta

(Wiedemann, 1830)

A72807C8-1431-5566-84B9-C89A8CFFEB9A

##### Notes

Lee and Kwon (1981), ME (1993), Kwon et al. (1996), Lee and Jung (2001), Lim et al. (2013)

#### Cecidomyiidae

6485D6E1-8C6F-58EE-832A-8941025CF49E

#### Thecodiplosis japonensis

Uchida & Inouye, 1955

3D7894FD-204F-5099-AEAF-0B465B6E22B3

##### Notes

Nam et al. (2018)

#### Chironomidae

E8CB9FFF-9CFD-50A6-8380-1A0B70AAB6C2

#### Chironomus circumdatus

(Kieffer, 1916)

3B5A5AF3-87C6-5575-8E66-2294732A17B0

##### Notes

Nam et al. (2018)

#### Chloropidae

D64107D8-2C5E-54D4-B85A-3493C6F3F154

#### Elachiptera insignis

(Thomson, 1869)

F992CEDA-8C3A-5013-A8C0-FAF6E132BA35

##### Materials

- **Type status:** Other material. **Occurrence:** recordedBy: Lee, D. Y.; sex: 1 male, 2 female; lifeStage: adult; occurrenceID: 1E40F52B-D910-5A21-A44E-2FD5B3FEEAA2; **Location:** island: Ulleungdo Island; country: Republic of Korea; stateProvince: Gyeongsangbuk-do; county: Ulleung-gun; locality: Nari basin, Nari-ri, Bukmyeon; **Event:** samplingProtocol: Sweeping net; eventDate: 8/18/2021
- **Type status:** Other material. **Occurrence:** recordedBy: Lee, D. Y.; sex: 1 female; lifeStage: adult; occurrenceID: 353DEFDE-3E2F-52DF-B91F-AED98B0B7295; **Location:** island: Ulleungdo Island; country: Republic of Korea; stateProvince: Gyeongsangbuk-do; county: Ulleung-gun; locality: Taeharyeong, Namseo-ri, Seomyeon; **Event:** samplingProtocol: Sweeping net; eventDate: 8/17/2021

##### Notes

This is the first record of this species from Ulleungdo Island.

Distribution: China, Japan, Korea, Russia, Taiwan (Liu et al. 2017)

#### Elachiptera sibirica

(Loew, 1858)

851DB823-3854-5B6C-848F-2809E6144DFD

##### Materials

- **Type status:** Other material. **Occurrence:** recordedBy: Lee, D. Y.; sex: 1 female; lifeStage: adult; occurrenceID: 1A829F8B-D867-53E6-9791-A30ED0C85570; **Location:** island: Ulleungdo Island; country: Republic of Korea; stateProvince: Gyeongsangbuk-do; county: Ulleung-gun; locality: Nari basin, Nari-ri, Bukmyeon; **Event:** samplingProtocol: Sweeping net; eventDate: 8/18/2021

##### Notes

This is the first record of this species from Ulleungdo Island.

Distribution: Austria, Bulgaria, China, Czech Republic, France, Hungary, Italy, Japan, Kazakhstan, Korea, Mongolia, Romania, Russia, Saudi Arabia, Slovakia, Switzerland, Ukraine (Liu et al. 2017)

#### Coelopidae

58E32804-B0EF-5D45-9EB4-A239ED88051B

#### Coelopa frigida

(Fabricius, 1805)

0E8F529F-768C-5FB8-8F4E-831958FEA1E8

##### Notes

Won et al. (2023)

#### Culicidae

7BAAD7AE-F97A-5A24-8B72-A5ED691930C1

#### Aedes albopictus

(Skuse, 1894)

F293CA42-40D5-5B9D-91AD-20E974986B07

##### Materials

- **Type status:** Other material. **Occurrence:** recordedBy: Lee, D. Y.; sex: 1 male, 1 female; lifeStage: adult; occurrenceID: 1D6C3A54-E089-57E0-BEE1-7EAB0A290065; **Location:** island: Ulleungdo Island; country: Republic of Korea; stateProvince: Gyeongsangbuk-do; county: Ulleung-gun; locality: Nari basin, Nari-ri, Bukmyeon; **Event:** samplingProtocol: Sweeping net; eventDate: 6/19/2021

##### Notes

This is the first record of this species from Ulleungdo Island.

Distribution: Albania, Algeria, Argentina, Australia, Austria, Bangladesh, Barbados, Belgium, Belize, Bermuda, Bhutan, Bolivia, Bosnia and Herzegovina, Brazil, Bulgaria, Cambodia, Cameroon, Cayman Islands, Central African Republic, China, Colombia, Comoros, Congo, Costa Rica, Croatia, Cuba, Czechia, Dominican Republic, El Salvador, Equatorial Guinea, Fiji, France, French Polynesia, Gabon, Georgia, Germany, Greece, Guam, Guatemala, Haiti, Honduras, Hong Kong, India, Indonesia, Israel, Italy, Japan, Korea, Laos, Lebanon, Macau, Madagascar, Malaysia, Maldives, Malta, Marshall Islands, Mauritius, Mexico, Montenegro, Myanmar, Nepal, Netherlands, New Caledonia, New Zealand, Nicaragua, Nigeria, Pakistan, Palau, Panama, Papua New Guinea, Paraguay, Philippines, Puerto Rico, Reunion, Romania, Russia, Samoa, San Marino, Seychelles, Singapore, Slovakia, Slovenia, Solomon Islands, South Africa, Spain, Sri Lanka, Switzerland, Syria, Taiwan, Thailand, Timor, Tonga, Trinidad and Tobago, Turkiye, Tuvalu, USA, Uruguay, Vanuatu, Venezuela, Vietnam (Rattanarithikul et al. 2010)

#### Aedes galloisi

Yamada, 1921

F795C8E9-2D53-52BE-9186-E49F5685AE8F

##### Materials

- **Type status:** Other material. **Occurrence:** recordedBy: Lee, D. Y.; sex: 1 female; lifeStage: adult; occurrenceID: ADEEC2E8-ADBE-5B42-9A54-70F760CA896B; **Location:** island: Ulleungdo Island; country: Republic of Korea; stateProvince: Gyeongsangbuk-do; county: Ulleung-gun; locality: Nari basin, Nari-ri, Bukmyeon; **Event:** samplingProtocol: black light traps (BioTrap, Seoul, ROK); eventDate: 8/15/2023

##### Notes

This is the first record of this species from Ulleungdo Island.

Distribution: China, Japan, Russia, Korea (Mogi et al. 1981)

#### Aedes koreicus

(Edwards, 1917)

BDA4D222-62D3-5E6B-8DA1-2B2CE7C7EEBF

##### Materials

- **Type status:** Other material. **Occurrence:** recordedBy: Lee, D. Y.; sex: 1 male; lifeStage: adult; occurrenceID: 9774B865-019E-50EA-9CD5-45E479646775; **Location:** island: Ulleungdo Island; country: Republic of Korea; stateProvince: Gyeongsangbuk-do; county: Ulleung-gun; locality: 196-1, Dodong-ri, Ulleungeup; **Event:** samplingProtocol: BG-sentinel trap (Biogents, Regensburg, Germany); eventDate: 8/18/2019

##### Notes

This is the first record of this species from Ulleungdo Island.

Distribution: Belgium, China, Germany, Hungary, Italy, Japan, Korea, Russia (Rattanarithikul et al. 2010)

#### Aedes togoi

(Theobald, 1907)

7890C02F-4900-5CD5-A877-8CD30FDA3E9A

##### Materials

- **Type status:** Other material. **Occurrence:** recordedBy: Lee, D. Y.; sex: 1 male; lifeStage: adult; occurrenceID: D64250A3-1077-5410-997E-6DB66271DAEF; **Location:** island: Ulleungdo Island; country: Republic of Korea; stateProvince: Gyeongsangbuk-do; county: Ulleung-gun; locality: 196-1, Dodong-ri, Ulleungeup; **Event:** samplingProtocol: BG-sentinel trap (Biogents, Regensburg, Germany); eventDate: 9/30/2019

##### Notes

This is the first record of this species from Ulleungdo Island.

Distribution: Cambodia, Canada, China, Japan, Korea, Malaysia, Russia, Taiwan, Thailand, USA, Vietnam (Rattanarithikul et al. 2010)

#### Anopheles sinensis

Wiedemann, 1828

3FE2A273-D851-583D-B0D9-1434A4752C4D

##### Notes

Cho (1965), Lee and Kwon (1981), ME (1993), Kwon et al. (1996), Lee and Jung (2001)

#### Armigeres subalbatus

(Coquillett, 1898)

D583507E-F96D-5845-982F-A096F7CE3643

##### Materials

- **Type status:** Other material. **Occurrence:** recordedBy: Lee, D. Y.; sex: 1 male; lifeStage: adult; occurrenceID: 8BABFA3C-DB2D-5DA7-B385-02E093D68AE7; **Location:** island: Ulleungdo Island; country: Republic of Korea; stateProvince: Gyeongsangbuk-do; county: Ulleung-gun; locality: 196-1, Dodong-ri, Ulleungeup; **Event:** samplingProtocol: BG-sentinel trap (Biogents, Regensburg, Germany); eventDate: 9/30/2019

##### Notes

This is the first record of this species from Ulleungdo Island.

Distribution: Bangladesh, Cambodia, China, India, Indonesia, Japan, Korea, Laos, Malaysia, Myanmar, Nepal, Pakistan, Philippines, Sri Lanka, Taiwan, Thailand, Vietnam (Rattanarithikul et al. 2010)

#### Culex orientalis

Edwards, 1921

788EA898-C520-59C7-9C10-6C25DF8DD848

##### Notes

Lee and Jung (2001)

#### Culex pipiens f. molestus

Forskal, 1775

9A479B6F-FDB8-5F15-BDFA-AFA73D5042FA

##### Notes

Ryu and Choi (2022)

#### Culex whitmorei

(Giles, 1904)

A872B582-7869-535D-9347-478811DB1FBF

##### Notes

Cho (1965), Lee and Kwon (1981), ME (1993), Kwon et al. (1996), Lee and Jung (2001)

#### Dolichopodidae

C48C7011-87E6-5DE8-B079-19BBC90EF25B

#### Condylostylus nebulosus

(Matsumura, 1916)

3D8DC807-CFEB-5F91-9030-6FA07AEA03E5

##### Notes

HNIBR (2022), Won et al. (2023)

#### Dolichopus nitidus

Fallén, 1823

7FDA87DA-A264-5E74-9252-1BEE274D9A3B

##### Notes

Nam et al. (2018)

#### Drosophilidae

20AE87DA-C0C8-5FED-86DE-AC24DA9E7AA0

#### Drosophila angularis

Okada, 1956

12AD61DB-D5FB-5203-9491-D291562B6D34

##### Notes

Lee and Kwon (1981), ME (1993), Kwon et al. (1996), Lee and Jung (2001), HNIBR (2022)

#### Drosophila auraria

Peng, 1937

5E6B8A0B-02B1-5EAF-8547-C71092DDAE00

##### Notes

Lee and Kwon (1981), ME (1993), Kwon et al. (1996), Lee and Jung (2001), HNIBR (2022)

#### Drosophila bifasciata

Pomini, 1940

D663C471-5080-5E70-9996-F5594BB574EA

##### Notes

Lee and Kwon (1981), ME (1993), Kwon et al. (1996), Lee and Jung (2001), HNIBR (2022)

#### Drosophila bizonata

Kikkawa & Peng, 1938

1E0F4486-0539-5EB5-8511-C93176DDE38A

##### Notes

Lee and Kwon (1981), ME (1993), Kwon et al. (1996), Lee and Jung (2001), HNIBR (2022)

#### Drosophila brachynephros

Okada, 1956

95F6E6C8-52FB-5A4E-A2C9-2A38CEE2F70B

##### Notes

Lee and Kwon (1981), ME (1993), Kwon et al. (1996), Lee and Jung (2001)

#### Drosophila histrio

Meigen, 1830

2482B971-4C01-5020-94D5-E149E384186B

##### Notes

Lee and Kwon (1981), ME (1993), Kwon et al. (1996), Lee and Jung (2001), HNIBR (2022)

#### Drosophila lacertosa

Okada, 1956

C34B8F35-0131-5031-90A8-0A90C2E88AB6

##### Notes

Lee and Kwon (1981), ME (1993), Kwon et al. (1996), Lee and Jung (2001), HNIBR (2022)

#### Drosophila melanogaster

Meigen, 1830

55BC25C6-6385-5913-9CC6-FEAAD25F8D0A

##### Notes

Lee and Kwon (1981), ME (1993), Kwon et al. (1996), Lee and Jung (2001)

#### Drosophila nigromaculata

Kikkawa & Peng, 1938

DA140FE1-D5C8-508E-A3FE-F63D3AD3842A

##### Notes

Lee and Kwon (1981), ME (1993), Kwon et al. (1996), Lee and Jung (2001), HNIBR (2022)

#### Drosophila simulans

Sturtevant, 1919

03A2F1B8-BA86-54BB-853D-0903B2303BC0

##### Notes

Nam et al. (2018)

#### Drosophila sordidula

Kikkawa & Peng, 1938

FC5AA854-1401-5222-9763-11F52DE931C6

##### Notes

Lee and Kwon (1981), ME (1993), Kwon et al. (1996), Lee and Jung (2001), HNIBR (2022)

#### Drosophila suzukii

(Matsumura, 1931)

354A12E9-50A8-5F8E-874F-693E7CA358FE

##### Notes

Lee and Kwon (1981), ME (1993), Kwon et al. (1996), Lee and Jung (2001), HNIBR (2022)

#### Drosophila unispina

Okada, 1956

88B4A72A-76E3-5506-BA2B-757FD7AC79B4

##### Notes

Lee and Kwon (1981), ME (1993), Kwon et al. (1996), Lee and Jung (2001)

#### Drosophila virilis

Sturtevant, 1916

3CBACC6D-B253-556A-AB70-6782A61F4C2D

##### Notes

Lee and Kwon (1981), ME (1993), Kwon et al. (1996), Lee and Jung (2001), HNIBR (2022)

#### Hirtodrosophila histrioides

(Okada & Kurokawa, 1957)

2DF5C934-657A-5977-A2B9-BE7FF8E480D4

##### Notes

Lee and Kwon (1981), ME (1993), Kwon et al. (1996), Lee and Jung (2001)

#### Hirtodrosophila sexvittata

(Okada, 1956)

79C25F84-2A38-5728-866B-6812E8CC6FDD

##### Notes

Lee and Kwon (1981), ME (1993), Kwon et al. (1996), Lee and Jung (2001)

#### Hirtodrosophila trilineata

(Chung, 1960)

326144E8-5DC9-587A-B5DB-BD5FEFF33837

##### Notes

Lee and Kwon (1981), ME (1993), Kwon et al. (1996), Lee and Jung (2001)

#### Mycodrosophila gratiosa

(Meijere, 1911)

7182E103-4A3C-5F1B-A5BD-0FDBD5A14B2C

##### Notes

Lee and Kwon (1981), ME (1993), Kwon et al. (1996), Lee and Jung (2001)

#### Scaptodrosophila coracina

(Kikkawa & Peng, 1938)

9CC300A5-EF0C-526A-AB26-B781A25F1CAF

##### Notes

Lee and Kwon (1981), ME (1993), Kwon et al. (1996), Lee and Jung (2001)

#### Scaptodrosophila puncticeps

(Okada, 1956)

23FD71E3-BB37-53C1-9F00-845FF284552C

##### Notes

Lee and Kwon (1981), ME (1993), Kwon et al. (1996), Lee and Jung (2001)

#### Scaptomyza graminum

(Fallén, 1823)

7ED0C82C-BEA5-564F-A082-1A427AA3292A

##### Notes

Lee and Kwon (1981), ME (1993), Kwon et al. (1996), Lee and Jung (2001), HNIBR (2022)

#### Scaptomyza pallida

(Zetterstedt, 1847)

C9B1FCD8-C0AA-570F-8A68-48DF10341224

##### Notes

Lee and Kwon (1981), ME (1993), Kwon et al. (1996), Lee and Jung (2001)

#### Empididae

47FA1EF1-2193-50CE-8155-F05ADC3BAC83

#### Empis latro

Frey, 1953

14785090-F9A5-56E5-B854-6E4C81B088D7

##### Notes

Nam et al. (2018)

#### Empis stercorea

Linnaeus, 1761

8FA39EC3-6640-5FDD-B59D-EDA2EEB63F50

##### Notes

Nam et al. (2018)

#### Fanniidae

15F5A7DF-D853-56A7-BE12-7891545406B0

#### Fannia canicularis

(Linnaeus, 1761)

C3E4012F-4944-5EAB-9F7B-F0841CE0A81A

##### Notes

Lee and Kwon (1981), ME (1993), Kwon et al. (1996), Lee and Jung (2001)

#### Fannia prisca

Stein, 1918

58078752-6F09-5B40-98AF-3572AA4B6B5A

##### Notes

Lee and Kwon (1981), ME (1993), Kwon et al. (1996), Lee and Jung (2001)

#### Fannia scalaris

(Fabricius, 1794)

51D8A3A0-EF69-58A9-B56E-7B9199F3B9C2

##### Notes

Lee and Kwon (1981), ME (1993), Kwon et al. (1996), Lee and Jung (2001)

#### Heleomyzidae

8732B8AB-69BD-5377-B004-8F3AFEAAE4AE

#### Suillia brunneipennis

Czerny, 1932

21AD7424-B60D-5A89-9B19-2C3C660B601A

##### Notes

Won et al. (2023)

#### Suillia lineitergum

(Pandellé, 1901)

AD4A6595-589C-5A0A-96D3-3A4DE31E4C72

##### Notes

Won et al. (2023)

#### Suillia nartshukella

Gorodkov, 1965

7275C074-F1C8-5369-A9E5-C3190C4F2D11

##### Notes

Won et al. (2023)

#### Lauxaniidae

BF82ABEE-5B23-5F8A-B018-4661A36E10B7

#### Homoneura filiola

Czerny, 1932

DEE68522-6A87-588D-A4A6-94E533EA4A7B

##### Notes

Won et al. (2023)

#### Homoneura haejuana

Sasakawa & Kozánek, 1995

7ABC0161-3669-5D6F-BF2A-DB28E29F14D5

##### Notes

Won et al. (2023)

#### Minettia longipennis

(Fabricius, 1794)

5B910B4C-B9C6-5077-ACBE-25D945D5D7F1

##### Notes

Lim et al. (2013)

#### Sciasmomyia supraorientalis

(Papp, 1984)

D13B1105-182B-5CA0-9E93-E598A2E2BC15

##### Notes

Won et al. (2023)

#### Limoniidae

A67A442F-7A50-59D7-8B61-F9CF6C9C33E1

#### Antocha bifida

Alexander, 1924

1742FFA4-593B-5179-A244-5915A5C0E5DD

##### Notes

Info and Park (2023)

#### Geranomyia semjina

Podenas, 2016

4EE115AB-8D93-5C06-BF9D-BFB6093E0495

##### Notes

Info and Park (2023)

#### Muscidae

DADCA812-58C7-55F4-86BD-DD02A3049545

#### Dichaetomyia bibax

(Wiedemann, 1830)

3FF2C6BE-BE77-5273-B58E-4EA8398F457A

##### Notes

Lee and Kwon (1981), ME (1993), Kwon et al. (1996), Lee and Jung (2001), Won et al. (2023)

#### Graphomyia maculata

(Scopoli, 1763)

DE480ABF-A881-58D2-8460-F489A00252EA

##### Notes

Kim (1971), Lee and Kwon (1981), ME (1993), Kwon et al. (1996), Lee and Jung (2001), Lim et al. (2013)

#### Graphomyia rufitibia

Stein, 1918

95048B06-3EE6-5D1D-A4A7-27AEE89135B5

##### Notes

Kim (1971), Lee and Kwon (1981), ME (1993), Kwon et al. (1996), Lee and Jung (2001)

#### Helina evecta

(Harris, 1780)

CEF4F714-8F84-55AA-B0BA-8FA9B794394A

##### Notes

Kim et al. (2023)

#### Hydrotaea dentipes

(Fabricius, 1805)

F37CE962-775D-558A-972D-AF71B57B6C29

##### Notes

Lee and Kwon (1981), ME (1993), Kwon et al. (1996), Lee and Jung (2001)

#### Lispe orientalis

Wiedemann, 1824

535AD661-CECF-5E08-ACF0-1D1748672D00

##### Notes

Lee and Kwon (1981), ME (1993), Kwon et al. (1996), Lee and Jung (2001)

#### Musca bezzii

Patton & Cragg, 1913

D564DDB5-A881-58A5-A7C6-29BE205A9ACE

##### Notes

Lee and Kwon (1981), ME (1993), Kwon et al. (1996), Lee and Jung (2001)

#### Musca domestica

Linnaeus, 1758

22A5BD5B-89B4-58DF-8C29-17BD2294B3F5

##### Notes

Cho (1955), Cho (1965), Lee and Kwon (1981), ME (1993), Kwon et al. (1996), Lee and Jung (2001), HNIBR (2022)

#### Musca hervei

Villeneuve, 1922

30F4DA3E-2532-512E-B5DD-21F309B68818

##### Notes

Kim (1971), Lee and Kwon (1981), ME (1993), Kwon et al. (1996), Lee and Jung (2001)

#### Musca sorbens

Wiedemann, 1830

C1755C14-D900-5F63-8DED-92105B4017AC

##### Notes

Lee and Kwon (1981), ME (1993), Kwon et al. (1996), Lee and Jung (2001)

#### Musca tempestiva

Fallén, 1817

ACB5B128-01AD-5784-8BFE-84D72621A69D

##### Notes

Lee and Kwon (1981), ME (1993), Kwon et al. (1996), Lee and Jung (2001)

#### Muscina angustifrons

(Loew, 1858)

84C61969-2BF0-50B6-8EC3-7049389A2BFF

##### Notes

Lee and Kwon (1981), ME (1993), Kwon et al. (1996), Lee and Jung (2001), HNIBR (2022)

#### Muscina levida

(Harris, 1780)

32E6EC55-4691-5BFF-943A-12EA71AC0E2B

##### Materials

- **Type status:** Other material. **Occurrence:** recordedBy: Lee, D. Y.; sex: 2 female; lifeStage: adult; occurrenceID: 6738E170-FF68-5D68-B437-F28673FEEE73; **Location:** island: Ulleungdo Island; country: Republic of Korea; stateProvince: Gyeongsangbuk-do; county: Ulleung-gun; locality: Taeharyeong, Namseo-ri, Seomyeon; **Event:** samplingProtocol: Pitfall trap (octopus); eventDate: 8/19/2021

##### Notes

This is the first record of this species from Ulleungdo Island.

Distribution: Holarctic (Vikhrev and Esin 2023)

#### Muscina stabulans

(Fallén, 1817)

F8FD8093-74B5-5F47-8C83-4087ACB8A9C1

##### Notes

Lee and Kwon (1981), ME (1993), Kwon et al. (1996), Lee and Jung (2001), HNIBR (2022)

#### Ophyra chalcogaster

(Wiedemann, 1824)

EB4D84BD-C56F-5E52-9DFC-25792D0B2543

##### Notes

Lee and Kwon (1981), ME (1993), Kwon et al. (1996), Lee and Jung (2001)

#### Ophyra leucostoma

(Wiedemann)

BDD0A978-3661-5781-9EE5-E555EF9CE2E0

##### Notes

Kim (1971), Lee and Kwon (1981), ME (1993), Kwon et al. (1996), Lee and Jung (2001)

#### Orchisia costata

(Meigen, 1826)

045C1883-E81F-5313-9E4E-EF8E1312A1E4

##### Materials

- **Type status:** Other material. **Occurrence:** recordedBy: Lee, D. Y.; sex: 1 male; lifeStage: adult; occurrenceID: FD644753-4803-5C51-B692-C96EF404F4F6; **Location:** island: Ulleungdo Island; country: Republic of Korea; stateProvince: Gyeongsangbuk-do; county: Ulleung-gun; locality: Taeharyeong, Namseo-ri, Seomyeon; **Event:** samplingProtocol: Sweeping net; eventDate: 8/19/2021

##### Notes

This is the first record of this species from Ulleungdo Island.

Distribution: Afrotropical, Australia, China, Europe, Japan, Korea, Oriental (Suh and Kwon 2017), Saudi Arabia (El-Hawagry et al. 2023)

#### Phaonia angustifrons

Shinonaga & Kano, 1971

AA31FECC-789A-53D3-BA04-C5220FB82E7B

##### Notes

Lee and Kwon (1981), ME (1993), Kwon et al. (1996), Lee and Jung (2001)

#### Phaonia fuscata

(Fallén, 1825)

26C45003-C58E-549E-B2D6-3F0AE6AF9AF7

##### Notes

Lee and Kwon (1981), ME (1993), Kwon et al. (1996), Lee and Jung (2001)

#### Phaonia gobertii

(Mik, 1881)

43B3FC91-62B8-5498-BA5D-BC374FA61B66

##### Notes

Lee and Kwon (1981), ME (1993), Kwon et al. (1996), Lee and Jung (2001)

#### Stomoxys calcitrans

(Linnaeus, 1758)

EE3B5181-4BE1-5AFB-A2D4-F2D579A0AC8E

##### Notes

Lee and Kwon (1981), ME (1993), Kwon et al. (1996), Lee and Jung (2001)

#### Ziminella asetosa

(Baranov, 1925)

9D5476F2-1743-5EE0-BCDF-B752EA413F18

##### Notes

Lee and Kwon (1981), ME (1993), Kwon et al. (1996), Lee and Jung (2001), HNIBR (2022)

#### Mycetophilidae

D8E1D3D8-9A86-5DEB-8B47-7B2838E3E7CB

#### Mycomya occultans

(Winnertz, 1863)

DA2C329E-212B-5F55-B4E7-7158E020FEC1

##### Notes

Kwon (2022)

#### Pediciidae

87D76A7F-839C-5236-B238-2C1EAE346155

#### Pedicia daimio

(Matsumura, 1916)

8E28664D-BA49-532C-8030-CD1370DB7735

##### Notes

Info (2023)

#### Platystomatidae

562A7CC0-3E37-5EBF-B4EA-CA87DC2E56C1

#### Euprosopia grahami

Malloch, 1931

09340CB9-EA7D-5ABE-857D-BDCDA6410FB0

##### Notes

Won et al. (2023)

#### Prosthiochaeta bifasciata

Hara, 1987

F54659B0-22E4-5745-8DC5-48B29D559C70

##### Notes

Kwon (2022)

#### Rivellia alini

Enderlein, 1937

74ADBE35-C9D1-5D5C-BB25-1077A4DB56DF

##### Notes

Lee and Jung (2001), Lim et al. (2013), Won et al. (2023)

#### Rivellia apicalis

Hendel, 1934

16242FAF-F53F-5651-A029-D89FEBF0ED54

##### Notes

Lee and Jung (2001)

#### Rivellia flaviventris

Hendel, 1914

EEA61699-F7C7-5617-A3EA-5FA088FF7E48

##### Notes

Kwon (2022)

#### Rivellia nigroapicalis

Byun & Suh, 2001

BC73AFC6-BE41-548A-94BF-14CE40F4E9E6

##### Notes

Lim et al. (2013), HNIBR (2022), Won et al. (2023)

#### Psychodidae

C346590F-2CA7-5396-A992-2FD61851AE5C

#### Psychoda alternata

Say, 1824

B3FCEFB5-C878-5E56-A35F-DEF8AA90B755

##### Notes

Nam et al. (2018)

#### Pyrgotidae

444172DF-D7F1-5CA7-B158-E8B111D0241B

#### Porpomastix fasciolata

Enderlein, 1942

4774ECBE-428E-5311-832D-F1AE2462444E

##### Notes

Lim and Lee (2012), HNIBR (2022)

#### Sarcophagidae

FA7CF3D7-7673-50F9-8749-3D77F24AC0F9

#### Parasarcophaga similis

(Meade, 1876)

4FEEC709-7430-5680-BCDA-AB7D61BF36A6

##### Notes

Park and Kano (1963), Lee and Kwon (1981), ME (1993), Kwon et al. (1996), Lee and Jung (2001), HNIBR (2022)

#### Phallosphaera gravelyi

(Senior-White, 1924)

70637B1B-2F01-5B4B-AC6A-2B4BCE12250B

##### Notes

Park and Kano (1963), Lee and Kwon (1981), ME (1993), Kwon et al. (1996), Lee and Jung (2001), HNIBR (2022)

#### Ravinia pernix

(Harris, 1780)

2A7A9903-5E50-576C-BB4C-94EA2CAA2639

##### Notes

Park and Kano (1963), Lee and Kwon (1981), ME (1993), Kwon et al. (1996), Lee and Jung (2001), HNIBR (2022)

#### Sarcophaga albiceps

Meigen, 1826

C0FC5C82-20CB-571B-B692-8162CBE8C999

##### Notes

Park and Kano (1963), Lee and Kwon (1981), ME (1993), Kwon et al. (1996), Lee and Jung (2001), HNIBR (2022)

#### Sarcophaga brevicornis

Ho, 1934

D8C169DE-1CE4-53B7-8A71-EC8096F02551

##### Notes

Park and Kano (1963), Lee and Kwon (1981), ME (1993), Kwon et al. (1996), Lee and Jung (2001)

#### Sarcophaga carnaria

(Linnaeus, 1758)

810EDD4F-2446-5B62-91BB-49B8B51CB07C

##### Notes

Cho (1955), Cho (1965), Lee and Jung (2001)

#### Sarcophaga crassipalpis

Macquart, 1839

CCCAF5A1-85EF-5A4C-BE4A-27E4D0673149

##### Notes

Park and Kano (1963), Lee and Kwon (1981), ME (1993), Kwon et al. (1996), Lee and Jung (2001), HNIBR (2022)

#### Sarcophaga harpax

Pandellé, 1896

CC9D98DF-DBAB-5CA7-8D47-C1114927B5FA

##### Notes

Park and Kano (1963), Lee and Kwon (1981), ME (1993), Kwon et al. (1996), Lee and Jung (2001), HNIBR (2022)

#### Sarcophaga josephi

Böttcher, 1912

D541B59D-5BFA-578B-83AE-D52577584210

##### Notes

Park and Kano (1963), Lee and Kwon (1981), ME (1993), Kwon et al. (1996), Lee and Jung (2001), HNIBR (2022)

#### Sarcophaga kanoi

Park, 1962

F69E1255-FA01-5BD4-978D-C21150515538

##### Notes

Park and Kano (1963), Lee and Kwon (1981), ME (1993), Kwon et al. (1996), Lee and Jung (2001)

#### Sarcophaga macroauriculata

Ho, 1932

73238F80-7E60-51C7-B5C7-5FB70FB12172

##### Notes

Park and Kano (1963), Lee and Kwon (1981), ME (1993), Kwon et al. (1996), Lee and Jung (2001)

#### Sarcophaga melanura

Meigen, 1826

B1B96601-02F7-59C9-9A45-A0D1E7CA3F6E

##### Notes

Park and Kano (1963), Lee and Kwon (1981), ME (1993), Kwon et al. (1996), Lee and Jung (2001), Lim et al. (2013), Nam et al. (2018), HNIBR (2022)

#### Sarcophaga misera

Walker, 1849

6E0DC7F1-53A4-5AFB-90F9-E4CADED4CF45

##### Notes

Park and Kano (1963), Lee and Kwon (1981), ME (1993), Kwon et al. (1996), Lee and Jung (2001)

#### Sarcophaga peregrina

(Robineau-Desvoidy, 1830)

DBF77B4D-BEDF-5C2B-8F9E-E13B63A69162

##### Notes

Park and Kano (1963), Lee and Kwon (1981), ME (1993), Kwon et al. (1996), Lee and Jung (2001), Nam et al. (2018)

#### Sarcophaga pingi

Ho, 1934

C09189AD-D2BF-53FC-94BF-FEEB11EA9209

##### Notes

HNIBR (2022)

#### Sarcophaga schuetzei

Kramer, 1909

521475A0-F279-5826-8FE4-EBB09D390377

##### Notes

Park and Kano (1963), Lee and Kwon (1981), ME (1993), Kwon et al. (1996), Lee and Jung (2001), HNIBR (2022)

#### Sarcophaga seniorwhitei

Bezzi, 1938

94E0C18C-5AF1-5A75-9053-FA5169033977

##### Notes

Lee and Kwon (1981), ME (1993), Kwon et al. (1996), Lee and Jung (2001), HNIBR (2022)

#### Sarcophaga shiritakaensis

Hori, 1954

B80ED130-D4F9-51EB-A0A9-461B6B73101B

##### Notes

Park and Kano (1963)

#### Sarcophaga tuberosa

Pandellé, 1896

40DA3D2D-4A53-5573-AA8F-48098DE1D9D1

##### Notes

Park and Kano (1963), Lee and Kwon (1981), ME (1993), Kwon et al. (1996), Lee and Jung (2001), HNIBR (2022)

#### Spirobolomyia basalis

(Walker, 1853)

9678A800-A8B3-5755-8D49-1854397E1ECF

##### Notes

Park and Kano (1963)

#### Scathophagidae

5FC81E0C-B25A-5405-9261-9CF852BA8838

#### Scathophaga mellipes

Coquillett, 1898

4ACAD5AE-E229-5464-BCF7-B71973BDBDB2

##### Notes

Won et al. (2023)

#### Scathophaga stercoraria

(Linnaeus, 1758)

FB4390D5-058A-5502-B21A-1678718B27A9

##### Notes

Lee and Kwon (1981), ME (1993), Kwon et al. (1996), Lee and Jung (2001), Nam et al. (2018), HNIBR (2022), Won et al. (2023)

#### Scatopsidae

131795AB-1BCD-5CEE-800E-19A7C2CEABDE

#### Coboldia fuscipes

(Meigen, 1830)

BBE02345-840D-5AE2-840C-3B88DAD7FCC1

##### Notes

Nam et al. (2018)

#### Sciaridae

84E2CBBC-DDCD-5FC9-A994-13C25DDB2564

#### Bradysia impatiens

(Johannsen, 1912)

3B3EF166-474A-5579-BA8B-E0E8BF8080B5

##### Notes

Nam et al. (2018)

#### Dolichosciara flavipes

(Meigen, 1804)

81DBC8B6-EE73-535A-8B5A-10E6C88EA220

##### Notes

Nam et al. (2018)

#### Phytosciara flavipes

(Meigen, 1804)

732818A0-98F1-56F1-9820-AAEA32F56D48

##### Notes

Kwon (2022)

#### Sciomyzidae

C34CF324-24B2-5501-9235-BE7B24C82908

#### Sepedon aenescens

Wiedemann, 1830

87607188-CB9B-57F3-BED0-63DB35FF49B7

##### Notes

Kwon (2022)

#### Sepsidae

D3F559D2-E320-5F80-BBA6-FF4BD937E6D2

#### Sepsis monostigma

Thomson, 1869

3390E93E-B715-5219-928D-02DC72610192

##### Notes

Lee and Kwon (1981), ME (1993), Kwon et al. (1996), Lee and Jung (2001)

#### Stratiomyidae

813AD58A-3077-5D5A-8743-0046B27AB7A2

#### Actina jezoensis

(Matsumura, 1916)

A0D7ACBF-882A-579B-99D9-77265DBF2C86

##### Notes

Nam et al. (2018)

#### Allognosta vagans

(Loew, 1873)

8FD81CBF-C466-5E17-8EF9-8BBB3D36F816

##### Notes

Won et al. (2023)

#### Craspedometopon frontale

Kertész, 1909

C425E545-151E-5CDE-943B-2463E54BB79C

##### Notes

Nam et al. (2018)

#### Hermetia illucens

(Linnaeus, 1758)

09B54E59-82E4-53FB-94A0-2E268A2BE172

##### Notes

Info (2023)

#### Microchrysa flaviventris

(Wiedemann, 1824)

ABE13FBF-661E-5FD6-B851-2A14C9767B37

##### Notes

Lee and Kwon (1981), ME (1993), Kwon et al. (1996), Lee and Jung (2001)

#### Ptecticus mitsuminensis

Ôuchi, 1940

0A549C70-2CB9-5E09-A5CF-FDA6E1793820

##### Notes

Lee and Jung (2001)

#### Ptecticus tenebrifer

(Walker, 1849)

1B43F081-A897-5D72-9DF9-C70754544CAD

##### Notes

Cho (1955), Cho (1965), Lee and Kwon (1981), ME (1993), Kwon et al. (1996), Lee and Jung (2001)

#### Syrphidae

933C41D9-40B6-5299-8431-654951D0429A

#### Allobaccha apicalis

(Loew, 1858)

9BDD60B8-C9AF-5CD6-ADE4-58C47B196B65

##### Notes

Lee and Jung (2001), Lim et al. (2013)

#### Allograpta javana

(Wiedemann, 1824)

12DE3ECF-9834-5D7C-8E72-EB537B1DAAAA

##### Notes

Lee and Jung (2001), Lim et al. (2013)

#### Asarkina porcina

(Coquillett, 1898)

3ADFB45E-DC23-590F-A93C-3982952CE808

##### Notes

Lee and Jung (2001)

#### Baccha maculata

Walker, 1852

C212404B-48A8-5386-8CBF-05EC3DE293C8

##### Notes

Info (2023)

#### Betasyrphus serarius

(Wiedemann, 1830)

D2DDFE2A-DCE3-5D7D-BC91-BF85C7E48A51

##### Notes

Lim et al. (2013)

#### Chalcosyrphus laterimaculatus

Jeong, Jung & Han, 2017

52E096BA-D550-5AA6-8F5E-355A29073563

##### Notes

Lim and Lee (2012), Lim et al. (2013), HNIBR (2022)

#### Chrysotoxum coreanum

Shiraki, 1930

4D8E3B99-1C12-5F6A-9554-C908A7C8E1D3

##### Notes

Lee and Jung (2001)

#### Chrysotoxum festivum

(Linnaeus, 1758)

F7FF4D32-DFFF-5868-9571-D7AAAD05AFCB

##### Notes

Kim (1971), Lee and Kwon (1981), ME (1993), Kwon et al. (1996), Lee and Jung (2001), HNIBR (2022)

#### Chrysotoxum shirakii

Matsumura, 1931

1AD1B098-15A8-5905-8E0F-09B371C804CC

##### Notes

Lim and Lee (2012), Lim et al. (2013)

#### Dasysyrphus albostriatus

(Fallén, 1817)

C0797EA6-2300-5311-8D15-68A839C9A122

##### Notes

Lim et al. (2013)

#### Dasysyrphus bilineatus

(Matsumura, 1917)

B03153D9-C1C2-5028-919D-15FB253110AB

##### Notes

Lim et al. (2013)

#### Dideoides latus

(Coquillett, 1898)

70BB2312-CB8E-51EE-A107-9FAD0B4A176B

##### Notes

Kim (1971), Lee and Kwon (1981), ME (1993), Kwon et al. (1996), Lee and Jung (2001), HNIBR (2022)

#### Episyrphus balteatus

(De Geer, 1776)

E2B8585D-712F-5516-BA45-F2869480D015

##### Notes

Kim (1971), Lee and Kwon (1981), ME (1993), Kwon et al. (1996), Lee and Jung (2001), Lim and Lee (2012), Lim et al. (2013), HNIBR (2022)

#### Eristalis arbustorum

(Linnaeus, 1758)

BE80D70C-0C0B-5D3A-AA86-48FC2C0578A7

##### Notes

Kim (1971), Lee and Kwon (1981), ME (1993), Kwon et al. (1996), Lee and Jung (2001), HNIBR (2022)

#### Eristalis cerealis

Fabricius, 1805

57E479F7-835A-5BF0-B7D2-4CC574D5D9EF

##### Notes

Cho (1955), Cho (1965), Lee and Kwon (1981), ME (1993), Kwon et al. (1996), Lee and Jung (2001), Lim and Lee (2012), Lim et al. (2013), HNIBR (2022)

#### Eristalis tenax

(Linnaeus, 1758)

F56D8CBA-CFE3-5F36-87B9-8750E6FDCE19

##### Notes

Kim (1971), Lee and Kwon (1981), ME (1993), Kwon et al. (1996), Lee and Jung (2001), Lim and Lee (2012), Lim et al. (2013), HNIBR (2022)

#### Eumerus japonicus

Matsumura, 1916

E020C7C3-80B6-5583-91F3-0E825D207ADA

##### Notes

Lee and Jung (2001), Lim and Lee (2012), Lim et al. (2013), HNIBR (2022)

#### Eupeodes corollae

(Fabricius, 1794)

FAB0E980-4C9F-573A-BD1E-39EF4B31D1C6

##### Notes

Lim et al. (2013), HNIBR (2022), Kwon (2022)

#### Eupeodes luniger

(Meigen, 1822)

3A53A672-F08A-5506-8F81-FDDBFCA9CCC9

##### Notes

Lee and Jung (2001)

#### Eupeodes nitens

(Zetterstedt, 1843)

64D28DD7-B4C2-5CFF-A9AE-9DC6EBA9C7B3

##### Notes

Lim et al. (2013)

#### Helophilus virgatus

Coquillett, 1898

19569592-83D4-5CD2-9E41-C28751F03F72

##### Notes

Lee and Jung (2001), Lim and Lee (2012), Lim et al. (2013), HNIBR (2022)

#### Melanostoma mellinum

(Linnaeus, 1758)

6593943F-8C4F-50D1-A067-525399402123

##### Notes

Lee and Jung (2001), Lim and Lee (2012), Lim et al. (2013), HNIBR (2022)

#### Melanostoma scalare

(Fabricius, 1794)

0E89AFE0-FB45-511D-AEF8-48D283CBF469

##### Notes

Lee and Kwon (1981), ME (1993), Kwon et al. (1996), Lee and Jung (2001), Lim et al. (2013)

#### Paragus fasciatus

Coquillett, 1898

DA2038C3-57CB-502D-9B93-ECA7B59D70BB

##### Notes

Lee and Kwon (1981), ME (1993), Kwon et al. (1996), Lee and Jung (2001)

#### Paragus haemorrhous

Meigen, 1822

A9E7210F-2AD3-5AF7-93F3-AD0DC1281DFA

##### Notes

Lee and Jung (2001), Lim et al. (2013)

#### Paragus jozanus

Matsumura, 1916

75467ECE-06C6-5F86-962C-762B40EC3009

##### Notes

Lee and Kwon (1981), ME (1993), Kwon et al. (1996), Lee and Jung (2001)

#### Paragus politus

Wiedemann, 1830

25F01496-0F01-578D-A06B-8D6B1084E85A

##### Notes

Lee and Kwon (1981), ME (1993), Kwon et al. (1996), Lee and Jung (2001)

#### Paragus quadrifasciatus

Meigen, 1822

DC59A014-3434-5172-8859-0A468CC628F3

##### Notes

Kim (1971), Lee and Kwon (1981), ME (1993), Kwon et al. (1996), Lee and Jung (2001)

#### Paragus tibialis

(Fallén, 1817)

FD9F6469-356C-506A-B2B3-F3592711686E

##### Notes

Kim (1971), Lee and Kwon (1981), ME (1993), Kwon et al. (1996), Lee and Jung (2001), HNIBR (2022)

#### Phytomia zonata

(Fabricius, 1787)

33874D48-0E4F-5A06-93F1-E582AFE1BF53

##### Notes

Lee and Kwon (1981), ME (1993), Kwon et al. (1996), Lee and Jung (2001)

#### Pterallastes unicolor

(Shiraki, 1930)

CD753D94-6806-5A1E-BE34-9AE68D1FCB66

##### Notes

Lim et al. (2013)

#### Sphaerophoria menthastri

(Linnaeus, 1758)

4B2F7666-1200-54E6-92DF-85D9B1E924AD

##### Notes

Kim (1971), Lee and Kwon (1981), ME (1993), Kwon et al. (1996), Lee and Jung (2001), Lim and Lee (2012), Lim et al. (2013), HNIBR (2022)

#### Sphegina clunipes

(Fallén, 1816)

E823B493-C31B-50AF-AFF7-312872F7834A

##### Notes

Kwon (2022)

#### Syritta pipiens

(Linnaeus, 1758)

D4A6C00B-3B23-5D28-BD8A-5F1B34CBC865

##### Notes

Lee and Jung (2001), Lim et al. (2013)

#### Syrphus ribesii

(Linnaeus, 1758)

1E4DB64B-FD0F-5561-8578-C341DA8437B7

##### Notes

Kim (1971), Lee and Kwon (1981), ME (1993), Kwon et al. (1996), Lee and Jung (2001), HNIBR (2022)

#### Syrphus torvus

Osten Sacken, 1875

EE9052AA-8A02-5A50-B76D-911B7CA49B7C

##### Notes

Lim et al. (2013)

#### Syrphus vitripennis

Meigen, 1822

490719F5-8C27-5966-BFF3-1823FF5A47E6

##### Notes

Lim et al. (2013)

#### Volucella nigricans

Coquillett, 1898

0EEEFF93-8885-575E-B9EF-F8381A235AC0

##### Notes

Lee and Kwon (1981), ME (1993), Kwon et al. (1996), Lee and Jung (2001), Lim et al. (2013)

#### Volucella pellucens tabanoides

Motschulsky, 1859

EA23FAC6-E82B-5D8E-89F7-2BC230290B07

##### Notes

Lee and Jung (2001), Lim et al. (2013)

#### Xanthandrus comtus

(Harris, 1780)

ECC3242D-6349-5E01-BBE4-B79C306460E4

##### Notes

Lee and Jung (2001), Lim et al. (2013)

#### Xylota coquilletti

Hervé-Bazin, 1914

4248904C-A383-5F8D-ACC0-E460D340ECBD

##### Notes

Kim (1971), Lee and Kwon (1981), ME (1993), Kwon et al. (1996), Lee and Jung (2001), Lim et al. (2013)

#### Tachinidae

59C84ABE-CF0D-5F88-81C1-4A17BADABCE1

#### Allophorocera rutila

(Meigen, 1824)

9F2CA14B-CDD8-5422-879E-4FA43F56B8BE

##### Notes

Kim (1971), Lee and Kwon (1981), ME (1993), Kwon et al. (1996), Lee and Jung (2001)

#### Aplomyia confinis

(Fallén, 1820)

40CBDC50-9AA8-51F6-95ED-8CE4442FD446

##### Notes

Kim (1971), Lee and Kwon (1981), ME (1993), Kwon et al. (1996), Lee and Jung (2001)

#### Blepharipa zebina

(Walker, 1849)

1B50FA4C-E2F0-5A2B-9D5A-12D47F8D61AE

##### Notes

Kim (1971), Lee and Kwon (1981), ME (1993), Kwon et al. (1996), Lee and Jung (2001), HNIBR (2022)

#### Cylindromyia brassicaria

(Fabricius, 1775)

EE88EA02-59B8-57CF-A8F4-2B9B2003C925

##### Notes

Lim et al. (2013)

#### Ectophasia crassipennis

(Fabricius, 1794)

44583DDA-72E2-516D-931E-979BEF424FC4

##### Notes

Lim et al. (2013)

#### Ectophasia rotundiventris

(Loew, 1858)

768DC894-9B16-5031-92A6-53E9DB10BBBF

##### Notes

Kim (1971), Lee and Kwon (1981), ME (1993), Kwon et al. (1996), Lee and Jung (2001), Lim and Lee (2012), Lim et al. (2013), Nam et al. (2018), HNIBR (2022)

#### Gonia chinensis

Wiedemann, 1824

CA93CC6C-22DE-514D-AE44-7AEBC20457AA

##### Notes

Lim et al. (2013)

#### Gymnosoma rotundatum

(Linnaeus, 1758)

D7ACCA6E-6D9A-5C90-9601-E9A0C43DDF62

##### Notes

Kim (1971), Lee and Kwon (1981), ME (1993), Kwon et al. (1996), Lee and Jung (2001), Lim et al. (2013)

#### Linnaemya atriventris

(Malloch, 1935)

0457612B-DB28-5022-9454-97D0A9E79805

##### Notes

Lim et al. (2013)

#### Prosena siberita

(Fabricius, 1775)

78491FCC-DCF4-5852-AFE5-856D6E847F9E

##### Notes

Lim et al. (2013), HNIBR (2022)

#### Senometopia excisa

(Fallén, 1820)

3BD88EEB-1C3B-583B-AA51-B8D6B03E5B65

##### Notes

Kim (1971), Lee and Kwon (1981), ME (1993), Kwon et al. (1996), Lee and Jung (2001)

#### Succingulum transvittata

Pandellé, 1896

14818F51-4EFD-5FB9-8267-53EE2496203F

##### Notes

Kim (1971), Lee and Kwon (1981), ME (1993), Lee and Jung (2001)

#### Tachina nupta

(Rondani, 1859)

CD410BB9-618A-5C5F-A9F3-698FDE22E344

##### Notes

Lee and Jung (2001), Lim and Lee (2012), Lim et al. (2013), Nam et al. (2018), HNIBR (2022)

#### Trigonospila ludio

(Zetterstedt, 1849)

B56604F2-128B-55B9-B8E6-76DDBF978B02

##### Notes

Lee and Jung (2001)

#### Trigonospila transvittata

(Pandellé, 1896)

443CF6B5-99C0-56C6-A0F3-B400B9DC7F78

##### Notes

Lee and Kwon (1981), ME (1993), Kwon et al. (1996), Lee and Jung (2001)

#### Tephritidae

6DC81D7B-0932-5895-80B4-544679D07818

#### Acanthonevra trigona

(Matsumura, 1905)

5F2FB024-8809-53BC-973C-6282757B1B8F

##### Notes

Won et al. (2023)

#### Acrotaeniostola scutellaris

(Matsumura, 1916)

CB83648A-6152-530B-9896-4E8A8C88ADF3

##### Notes

Kwon et al. (1996), Lee and Jung (2001)

#### Anomoia purmunda

(Harris, 1780)

31AC71AF-D809-55EE-A556-B7D3BFC233F0

##### Notes

Lim et al. (2013), Won et al. (2023)

#### Bactrocera depressa

(Shiraki, 1933)

783BAB57-717B-59B0-B136-BD79A8798555

##### Notes

Lim et al. (2013)

#### Bactrocera scutellata

(Hendel, 1912)

9D235D61-23A3-594A-8E8D-DAA1A59DBB35

##### Notes

Lee and Jung (2001)

#### Campiglossa deserta

(Hering, 1939)

8C7B1C45-AE18-580A-99E2-9E937E396A01

##### Notes

Kwon (2022)

#### Campiglossa hirayamae

(Matsumura, 1916)

E3E102AF-45FE-52A5-82C8-96AE8E3271A4

##### Notes

Lee and Kwon (1981), ME (1993), Kwon et al. (1996), Lee and Jung (2001)

#### Ensina sonchi

(Linnaeus, 1767)

5FB599CE-C709-5854-AAA5-E6E291C8F318

##### Materials

- **Type status:** Other material. **Occurrence:** recordedBy: Lee, D. Y.; sex: 4 male, 1 female; lifeStage: adult; occurrenceID: 0DCE82C7-BB81-58DB-AE6C-5FC61B53CD08; **Location:** island: Ulleungdo Island; country: Republic of Korea; stateProvince: Gyeongsangbuk-do; county: Ulleung-gun; locality: Taeharyeong, Namseo-ri, Seomyeon; **Event:** samplingProtocol: Sweeping net; eventDate: 6/19/2021

##### Notes

This is the first record of this species from Ulleungdo Island.

Distribution: Palaearctic (GBIF.org 2023a)

#### Hemilea infuscata

Hering, 1937

DCDBC994-EC36-5FE5-9C29-B7ECBAC82AFA

##### Notes

Lim and Lee (2012), Lim et al. (2013)

#### Trupanea amoena

(Frauenfeld, 1857)

DF4446C2-879B-5F6F-98C8-5D1EF2870494

##### Notes

Kwon et al. (1996), Lee and Jung (2001)

#### Trupanea convergens

(Hering, 1936)

27464F7A-F40D-5322-9166-EBFD8AA677D9

##### Notes

Lee and Jung (2001)

#### Tipulidae

145E010D-590F-5418-A323-9ECD8B05679A

#### Nephrotoma cornicina cornicina

(Linnaeus, 1758)

B9A75E66-A125-54E3-A9C3-0E7499CD3B6E

##### Notes

Lim and Lee (2012), HNIBR (2022)

#### Tipula aino

Alexander, 1914

526F326B-A796-585A-856B-C1F1F7637BED

##### Notes

Cho (1955), Cho (1965), Lee and Kwon (1981), ME (1993), Kwon et al. (1996), Lee and Jung (2001), Lim and Lee (2012), HNIBR (2022)

#### Tipula coquilletti

Enderlein, 1912

86E3D557-0401-5725-AE61-8A2608F41D76

##### Notes

Nam et al. (2018)

#### Tipula latemarginata latemarginata

Alexander, 1921

1EEA1F7C-22CC-5501-9731-C63773F00D9A

##### Notes

NIBR (2021)

#### Tipula nipponensis

Alexander, 1914

DE021765-AF5E-5BCC-B01E-3314540910C2

##### Notes

Lee and Jung (2001)

#### Tipula taikun

Alexander, 1921

7F290AC2-4E43-5491-85F6-4A70797F0C0F

##### Notes

HNIBR (2022), Kwon (2022)

#### Ephemeroptera

638CD07B-6833-5AD3-96BF-5DC46FD89CAC

#### Baetidae

D0017C94-261C-5B40-BC01-95CCA69FC5D3

#### Acentrella sibirica

(Kazlauskas, 1963)

2B00CDDD-A1C0-5D3B-A2EA-FAC1CEFA3CE9

##### Notes

NIBR (2021)

#### Baetiella tuberculata

(Kazlauskas, 1963)

93DA4007-A9DF-5E2E-9AA0-3D86D1BD9BBE

##### Notes

Bae (2010)

#### Baetis fuscatus

(Linnaeus, 1761)

F84CF311-51F0-53B1-9F70-843F1300F643

##### Notes

Kwon (2021)

#### Baetis silvaticus

Kluge, 1983

281BBF7C-6EF8-5AC8-A473-FB03607AD61A

##### Notes

Kwon (2021)

#### Cloeon dipterum

(Linnaeus, 1761)

908CF241-9657-5607-9A4C-2415E9DBD322

##### Notes

Kwon (2021)

#### Labiobaetis atrebatinus

(Eaton, 1870)

240AF3C5-5616-547E-B304-8A0B7F34F82C

##### Notes

Kwon (2021)

#### Nigrobaetis bacillus

(Kluge, 1983)

0C2008E7-866A-5D3E-99FE-844C8BBCE604

##### Notes

Lim and Lee (2012), HNIBR (2022)

#### Procloeon halla

Bae & Park, 1997

31E219E9-932F-5806-8EF4-4F01AA58388B

##### Notes

Kwon (2021)

#### Ephemerellidae

258C7FA0-4687-5738-B9CF-EA17D5705905

#### Cincticostella levanidovae

(Tshernova, 1952)

9298BFDE-BAF2-544C-B0B9-0D4F03FE5858

##### Notes

Kwon (2021)

#### Drunella aculea

(Allen, 1971)

144FED9B-58A3-566C-85CF-E5BEF108221A

##### Notes

Kwon (2021)

#### Drunella ishiyamana

Matsumura, 1931

932ABCB3-6DAB-522F-B3C4-F36E2B79795C

##### Notes

Kwon (2021)

#### Serratella setigera

(Bajkova, 1967)

03F2A13B-B24F-5ACC-92FF-A59126C0FC05

##### Notes

Kwon (2021)

#### Teloganopsis punctisetae

(Matsumura, 1931)

9BE74B27-DEEC-59D7-BC7C-3C624A88E6C3

##### Notes

Kwon (2021)

#### Ephemeridae

A74DB479-D658-56B0-92BC-C9D073617681

#### Ephemera orientalis

McLachlan, 1875

49DE15AB-3ADD-5901-AD20-312DA72B5210

##### Notes

Kwon (2021)

#### Ephemera strigata

Eaton, 1892

3E670936-0449-58B9-8E31-A5D1A0829842

##### Notes

Kwon (2021)

#### Heptageniidae

8AF0F8F9-0977-5266-B51A-2D3C43E88AD5

#### Ecdyonurus bajkovae

Kluge, 1986

617D6441-07B3-5720-AADF-DCCB7FD86787

##### Notes

Kwon (2021)

#### Ecdyonurus kibunensis

Imanishi, 1936

B0DF48EE-8846-5B4D-B423-258EF86718E6

##### Notes

Kwon (2021)

#### Ecdyonurus levis

(Navás, 1912)

5198A4EC-B38D-5986-BF54-A23CFD932354

##### Notes

Kwon (2021)

#### Epeorus nipponicus

(Uéno, 1931)

7F86E04C-BA7F-59CB-9FAD-9DA110EC47F0

##### Notes

Lim and Lee (2012), Kwon (2021), HNIBR (2022)

#### Epeorus pellucidus

(Brodsky, 1930)

69A62892-B991-5CFF-8569-A5E9186454C1

##### Notes

Kwon (2021)

#### Leptophlebiidae

7ED79302-504C-5A32-8C74-9D64B11762BE

#### Choroterpes altioculus

Kluge, 1984

67B2CB11-47D3-55E0-8738-734C2796129E

##### Notes

Kwon (2021)

#### Paraleptophlebia japonica

(Matsumura, 1931)

F43EA1F3-F247-51DB-9AF5-0BD202FE979D

##### Notes

Kwon (2021)

#### Micronectidae

F4463097-916E-5890-91A9-7688A29FC630

#### Micronecta guttata

Matsumura, 1905

EB9F8CD1-7BC1-5F70-9EBB-8DE365037B28

##### Notes

Lim and Lee (2012), HNIBR (2022)

#### Hemiptera

99E7DA18-6A3D-50FC-9481-C27F02CCF24A

#### Acanthosomatidae

28F39B89-AB65-5698-9D5C-33C3EF4B6C5C

#### Acanthosoma crassicaudum

Jakovlev, 1880

D4A3A2A2-E8AB-532E-BED4-1F78AA74AF91

##### Notes

Lim et al. (2013), Won et al. (2023)

#### Acanthosoma denticaudum

Jakovlev, 1880

78694BB3-A5F7-571B-9C85-6D2F6C37005F

##### Notes

Lee and Kwon (1981), ME (1993), Kwon et al. (1996), Lee and Jung (2001), Won et al. (2023)

#### Acanthosoma firmatum

(Walker, 1868)

F893A93B-B501-5C5C-9728-A5FB8845E45C

##### Notes

Lee and Jung (2001)

#### Acanthosoma forficula

Jakovlev, 1880

30393B08-7E27-5A57-B7C1-8B0B69EA0435

##### Notes

ME (1993), Kwon et al. (1996), Lee and Jung (2001), Lim and Lee (2012), HNIBR (2022), Won et al. (2023)

#### Acanthosoma labiduroides

Jakovlev, 1880

7E75D196-6A56-5CD5-9F80-B53D1B7ACD55

##### Notes

Cho (1955), Cho (1965), Lee and Kwon (1981), ME (1993), Kwon et al. (1996), Lee and Jung (2001), HNIBR (2022)

#### Acanthosoma spinicolle

Jakovlev, 1880

6ABBB9CD-6B09-595F-8C3B-DF1B154850BB

##### Notes

ME (1993), Kwon et al. (1996), Lee and Jung (2001)

#### Elasmostethus humeralis

Jakovlev, 1883

671C8EBA-DED2-5CEF-81ED-515840D850D6

##### Notes

Kim (1971), Lee and Kwon (1981), ME (1993), Kwon et al. (1996), Lee and Jung (2001), HNIBR (2022)

#### Elasmostethus nubilus

(Dallas, 1851)

00D218ED-6336-5873-A9DD-5C658831506C

##### Notes

HNIBR (2022), Kwon (2022), Won et al. (2023)

#### Elasmucha putoni

Scott, 1874

0EF34852-760D-5D4E-8075-5C83D8F6C040

##### Notes

Lee and Kwon (1981), ME (1993), Kwon et al. (1996), Lee and Jung (2001), Lim and Lee (2012), Lim et al. (2013), HNIBR (2022)

#### Sastragala esakii

Hasegawa, 1959

AD99C37F-9480-5BBC-A631-CA7E045B34F1

##### Notes

Lee and Kwon (1981), ME (1993), Kwon et al. (1996), Lee and Jung (2001), Lim et al. (2013)

#### Sastragala scutellata

(Scott, 1874)

01E30EE7-6765-58F0-AF4A-CD2F9E103DFC

##### Notes

Kim (1971), Lee and Kwon (1981), ME (1993), Kwon et al. (1996), Lee and Jung (2001), HNIBR (2022), Won et al. (2023)

#### Achilidae

2B7C99D1-7E0E-582D-8263-36113D2B4128

#### Errada nawae

(Matsumura, 1914)

491BBD72-3393-5BF9-8603-AAC590F75C2F

##### Notes

Lee and Kwon (1981), ME (1993), Kwon et al. (1996), Lee and Jung (2001), Won et al. (2023)

#### Aleyrodidae

51AD6827-3F14-569D-9874-DD7EB3B74F4E

#### Trialeurodes vaporariorum

(Westwood, 1856)

A0980710-2BEE-5A17-8F26-7552D8CC71A3

##### Notes

Nam et al. (2018)

#### Alydidae

C1B621D0-DD36-5935-B044-5262B704685C

#### Alydus calcaratus

(Linnaeus, 1758)

7589E57E-F692-5F89-AF4A-0930C6885E33

##### Notes

Won et al. (2023)

#### Leptocorisa chinensis

(Dallas, 1852)

F6A45A33-8C52-57F6-A213-38EAC45C08D8

##### Notes

Lim et al. (2013)

#### Paraplesius unicolor

Scott, 1874

9ECFB35F-FDE5-5BA5-8E68-EE53D7606FB0

##### Notes

Lee and Jung (2001), Lim et al. (2013), Won et al. (2023)

#### Riptortus pedestris

(Fabricius, 1775)

947E25F4-D8DF-5E31-9613-AA9E881612C5

##### Notes

Cho (1955), Cho (1965), Lee and Kwon (1981), ME (1993), Kwon et al. (1996), Lee and Jung (2001), Lim et al. (2013), Won et al. (2023)

#### Anthocoridae

604763B3-AAB1-530D-856C-83AF2453B3AC

#### Anthocoris miyamotoi

Hiura, 1959

DA5C43E3-156A-53E0-9188-EE83510436F2

##### Notes

ME (1993), Kwon et al. (1996), Lee and Jung (2001)

#### Orius sauteri

(Poppius, 1909)

D1B392C9-211E-5CB4-B498-9CFF70269EBC

##### Notes

Lee and Kwon (1981), ME (1993), Kwon et al. (1996), Lee and Jung (2001)

#### Aphalaridae

B040CB2C-675A-59D0-ACF3-E4EA41F8028B

#### Aphalara itadori

(Shinji, 1938)

52552C76-36D7-5AF0-BFD2-CA9B9BAB8289

##### Notes

Lee and Kwon (1981), ME (1993), Kwon et al. (1996), Lee and Jung (2001)

#### Celtisaspis japonica

(Miyatake, 1968)

D9526760-E4E6-5DC9-A6E3-4E4261CC0E6B

##### Notes

Lee and Kwon (1981), ME (1993), Kwon et al. (1996), Lee and Jung (2001)

#### Aphididae

1636F7B7-63C6-5E5D-BE3F-C3852B3ED006

#### Aleurodaphis asteris

Takahashi & Sorin, 1958

DDBA098B-2CA6-5AC1-A84A-42D5CCD76F59

##### Notes

Lee and Kwon (1981), ME (1993), Kwon et al. (1996), Lee and Jung (2001)

#### Aleurodaphis blumeae

Van der Goot, 1917

C28BDBCF-0610-5308-BAAB-1EBE3F7FDCD5

##### Notes

Lee and Kwon (1981), ME (1993), Kwon et al. (1996), Lee and Jung (2001)

#### Aphis aurantii

Boyer de Fonscolombe, 1841

D0A9F5CF-BD68-5258-BF55-5D4CA49FE12F

##### Notes

Lee and Kwon (1981), ME (1993), Kwon et al. (1996), Lee and Jung (2001)

#### Aphis fabae

Scopoli, 1763

89B1968D-748F-5F5B-B4CD-DC3A89B26BED

##### Notes

HNIBR (2022)

#### Aphis gossypii

Glover, 1877

B1F2AA8B-3716-5802-90DA-5DE4C2F57173

##### Notes

Nam et al. (2018)

#### Aphis horii

Takahashi, 1923

C56769FD-9CA7-5302-BE5C-D81600CBA5C7

##### Notes

Lee and Kwon (1981), ME (1993), Kwon et al. (1996), Lee and Jung (2001)

#### Aphis veroniciphaga

Kim & Lee, 2006

BFD7C9F5-9C8F-5795-8D79-3DD66D88F9DF

##### Notes

Kim et al. (2006)

#### Aulacorthum ibotum

(Essig & Kuwana, 1918)

C70B26BD-ACCF-5604-8445-E0D31C8A7A39

##### Notes

Lee and Kwon (1981), ME (1993), Kwon et al. (1996), Lee and Jung (2001)

#### Cinara formosanum

(Takahashi, 1924)

49121485-B8F9-51CF-9395-9E3A207A1FB8

##### Notes

Lee and Kwon (1981), ME (1993), Kwon et al. (1996), Lee and Jung (2001)

#### Cryptosiphum artemisiae

Buckton, 1879

810655FB-2C88-5F50-B4E8-7B2ACCA231AE

##### Notes

Lee and Kwon (1981), ME (1993), Kwon et al. (1996), Lee and Jung (2001)

#### Hayhurstia atriplicis

(Linnaeus, 1761)

552F33E1-A7CF-57F4-90FF-96C74CD3410C

##### Notes

Lee and Kwon (1981), ME (1993), Kwon et al. (1996), Lee and Jung (2001)

#### Macrosiphoniella formosartemisiae

Takahashi, 1921

A3F637D3-B035-5EF8-A637-D8776F4AE8D1

##### Notes

Lee and Kwon (1981), ME (1993), Kwon et al. (1996), Lee and Jung (2001)

#### Macrosiphoniella oblonga

(Mordvilko, 1901)

ECD1D7D8-9E06-5A03-89C7-4190100A2A35

##### Notes

Nam et al. (2018)

#### Macrosiphoniella yomogifoliae

(Shinji, 1922)

6648230C-C298-546F-8D3F-E34010590F51

##### Notes

Lee and Kwon (1981), ME (1993), Kwon et al. (1996), Lee and Jung (2001)

#### Matsumuraja rubi

(Matsumura, 1918)

CD5E2464-1035-598E-973F-3833F0DDBB9E

##### Notes

Lee and Kwon (1981), ME (1993), Kwon et al. (1996), Lee and Jung (2001), HNIBR (2022)

#### Myzus asteriae

Shinji, 1941

0B3F83B7-C7AB-5091-A633-86F85387051C

##### Notes

Lee and Kwon (1981), ME (1993), Kwon et al. (1996), Lee and Jung (2001)

#### Myzus siegesbeckiae

Takahashi, 1965

DF9DA8D8-D3B9-54EE-BA90-4BE2225FF790

##### Notes

Lee and Kwon (1981), ME (1993), Kwon et al. (1996), Lee and Jung (2001)

#### Periphyllus kuwanaii

(Takahashi, 1919)

24B667FB-497D-5271-87C3-392DD2FE902C

##### Notes

Kwon et al. (1996), Lee and Jung (2001)

#### Phorodon humuli japonensis

Takahashi, 1965

C4C17502-AD6C-53AF-A361-C0B645520781

##### Notes

Lee and Kwon (1981), ME (1993), Kwon et al. (1996), Lee and Jung (2001)

#### Rhopalosiphum padi

(Linnaeus, 1758)

01C0B93A-06F9-5CD8-BBBE-738513EFEE55

##### Notes

Nam et al. (2018)

#### Semiaphis heraclei

(Takahashi, 1921)

38214849-D510-5FD0-8FB0-D33EBB0AD6DD

##### Notes

Lee and Kwon (1981), ME (1993), Kwon et al. (1996), Lee and Jung (2001)

#### Sitobion ibarae

(Matsumura, 1917)

187924B6-1CDB-57F7-8F4F-F081B985A4D9

##### Notes

Lee and Kwon (1981), ME (1993), Kwon et al. (1996), Lee and Jung (2001)

#### Uroleucon formosanum

(Takahashi, 1921)

26BDC776-85B6-5B78-AE0E-BE20B799E619

##### Notes

Lee and Kwon (1981), ME (1993), Kwon et al. (1996), Lee and Jung (2001)

#### Aphrophoridae

C761F3EA-B8F8-53E3-903E-7CB37A9DEEAE

#### Aphrophora alni

(Fallén, 1805)

757D275E-B6CD-5508-8A9F-A9E7DC4E2F09

##### Notes

Lee and Kwon (1981), ME (1993), Kwon et al. (1996), Lee and Jung (2001), Lim et al. (2013)

#### Obiphora intermedia

(Uhler, 1896)

22D759F5-8055-56C1-BC8E-5CC3E4418528

##### Notes

Lee and Kwon (1981), ME (1993), Kwon et al. (1996), Lee and Jung (2001), Won et al. (2023)

#### Petaphora maritima

(Matsumura, 1903)

5928EEC0-5D27-5E36-9A19-793066DFAFC4

##### Notes

Lee and Kwon (1981), ME (1993), Kwon et al. (1996), Lee and Jung (2001)

#### Tilophora flavipes

(Uhler, 1896)

F0137951-67A4-50E4-BB55-BE3E3E112E01

##### Notes

Lee and Kwon (1981), ME (1993), Kwon et al. (1996), Lee and Jung (2001)

#### Berytidae

38ED639D-A92E-58B5-9C61-E5EC75C7B6A0

#### Yemma exilis

(Horváth, 1905)

34CE046B-5372-5536-B77B-2E0D33E81D5E

##### Notes

Lee and Kwon (1981), ME (1993), Kwon et al. (1996), Lee and Jung (2001)

#### Blissidae

F8E530FE-2234-5060-BC9C-B20C98A676DD

#### Dimorphopterus pallipes

(Distant, 1883)

4BD1A330-E937-5446-9C7D-828B426A1AD3

##### Notes

Lim and Lee (2012)

#### Macropes obnubilus

(Distant, 1883)

372934CA-9297-5ED6-8177-C749638DC551

##### Notes

Lee and Kwon (1981), ME (1993), Kwon et al. (1996), Lee and Jung (2001)

#### Calophyidae

9385CD0B-D867-5618-A1A1-E8CCEF4DEA74

#### Calophya nigra

Kuwayama, 1908

009FC42D-96B9-5616-B2D4-B318111C0E19

##### Notes

Lee and Kwon (1981), ME (1993), Lee and Jung (2001)

#### Calophya shinjii

Sasaki, 1954

070DEE1E-AA7E-5F7F-93B4-3399EDC28797

##### Notes

Kwon et al. (1996), Lee and Jung (2001)

#### Cicadellidae

8A8ECC4E-B534-5AEF-B491-1F27426825B6

#### Austroasca vittata

(Lethierry, 1884)

90217AC3-D3AE-544B-8DF0-AD379FBD55B2

##### Notes

Lee and Kwon (1981), ME (1993), Kwon et al. (1996), Lee and Jung (2001)

#### Balclutha rubrinervis

(Matsumura, 1902)

1FF69ECC-4789-5C60-92FA-6EA1187E0AE1

##### Notes

Lee and Kwon (1981), ME (1993), Kwon et al. (1996), Lee and Jung (2001)

#### Bhatia koreana

(Kwon & Lee, 1979)

046A6CD9-869F-577E-AF7B-07615AE44223

##### Notes

Lee and Kwon (1981), ME (1993), Kwon et al. (1996), Lee and Jung (2001)

#### Bhatia satsumensis

(Matsumura, 1914)

E0D98CF6-781A-514C-B5F2-EEF0D57C356E

##### Notes

Kwon et al. (1996), Lee and Jung (2001)

#### Bothrogonia ferruginea

(Fabricius, 1787)

0D390845-BC48-56B8-860B-B766D03F4195

##### Notes

Kwon (2022)

#### Cicadella viridis

(Linnaeus, 1758)

AAEA50CC-3F28-5DBA-8705-1BBF9D6D46D4

##### Notes

Kim (1971), Lee and Kwon (1981), ME (1993), Kwon et al. (1996), Lee and Jung (2001), Lim et al. (2013), HNIBR (2022), Won et al. (2023)

#### Drabescus limbaticeps

(Stål, 1858)

B4230017-3E82-5A2B-87C4-A1180D4670D3

##### Notes

Lee and Jung (2001)

#### Drabescus nigrifemoratus

(Matsumura, 1905)

4B864A57-AA92-5C7C-BC15-F03A2CE5635D

##### Notes

Kwon et al. (1996), Lee and Jung (2001), Won et al. (2023)

#### Drabescus nitobei

Matsumura, 1912

4843E933-2D75-5017-BB1C-BED2B2CCCF7C

##### Notes

Won et al. (2023)

#### Drabescus ogumae

Matsumura, 1912

62C48D70-ED9D-5DCD-8E15-D9B50B2D74B5

##### Notes

Lee and Kwon (1981), ME (1993), Kwon et al. (1996), Lee and Jung (2001)

#### Dryodurgades lamellaris

Vilbaste, 1968

6CDE9A22-49D9-54C4-8866-BC15D1A0D452

##### Notes

Kwon (2022)

#### Futasujinus candidus

(Matsumura, 1914)

FB5D0A65-BEC5-56FC-BFA5-681F502E6248

##### Notes

Lee and Kwon (1981), ME (1993), Kwon et al. (1996), Lee and Jung (2001)

#### Handianus limbifer

(Matsumura, 1902)

68C6CC08-DB3E-54E1-8F0B-D460B7202648

##### Notes

Kwon (2022)

#### Hishimonus sellatus

(Uhler, 1896)

CB43C277-5A29-5D1B-BC3D-F97B82F4EC45

##### Notes

Lee and Kwon (1981), ME (1993), Kwon et al. (1996), Lee and Jung (2001)

#### Idiocerus ishiyamae

Matsumura, 1905

C9E8CD04-6E60-53F0-8DBA-44F18634163A

##### Notes

Lee and Kwon (1981), ME (1993), Kwon et al. (1996), Lee and Jung (2001), Won et al. (2023)

#### Japananus aceri

(Matsumura, 1914)

E16F293D-2BBF-5E74-987D-A9A55E4BFEC7

##### Notes

Lee and Kwon (1981), ME (1993), Kwon et al. (1996), Lee and Jung (2001)

#### Laburrus impictifrons

(Boheman, 1852)

807DB750-594D-5279-8025-5FBEF0F8DDBB

##### Notes

Lee and Kwon (1981), ME (1993), Kwon et al. (1996), Lee and Jung (2001), Lim et al. (2013)

#### Macrosteles quadrimaculatus

(Matsumura, 1900)

CF1FCFF9-421B-5DCB-A8B3-C8C03527241F

##### Notes

Lee and Kwon (1981), ME (1993), Kwon et al. (1996), Lee and Jung (2001)

#### Maiestas oryzae

(Matsumura, 1902)

532B0D30-A391-5956-984B-733D8F20A2DD

##### Notes

Lee and Kwon (1981), ME (1993), Kwon et al. (1996), Lee and Jung (2001)

#### Matsumurella curticauda

Anufriev, 1971

2A192938-42DF-5FB0-A655-DADC9B8916E5

##### Notes

Kwon (2022)

#### Metidiocerus rutilans

(Kirschbaum, 1868)

DC12422F-6603-5727-9EED-251F0A0571A6

##### Notes

Lim et al. (2013)

#### Neotituria kongosana

(Matsumura, 1915)

F52300D2-865A-5676-ADEB-15A8806A1DAF

##### Notes

Lim et al. (2013), Won et al. (2023)

#### Nephotettix cincticeps

(Uhler, 1896)

5FB38FF2-D7D7-5E3B-8F06-75D79741E809

##### Notes

Kim (1971), Lee and Kwon (1981), ME (1993), Kwon et al. (1996), Lee and Jung (2001)

#### Pagaronia albescens

(Jacobi, 1943)

287A5C68-3A5B-5049-86F9-F690E259BDF2

##### Notes

Lee and Kwon (1981), ME (1993), Kwon et al. (1996), Lee and Jung (2001)

#### Paracyba akashiensis

(Takahashi, 1928)

17E6A15D-767C-5A56-9DFF-6155180301C0

##### Notes

Lee and Kwon (1981), ME (1993), Kwon et al. (1996), Lee and Jung (2001)

#### Paralaevicephalus nigrifemoratus

(Matsumura, 1902)

30E1337D-B1B3-556F-B09E-4799FBA4B4F8

##### Notes

Lee and Kwon (1981), ME (1993), Kwon et al. (1996), Lee and Jung (2001)

#### Paralimnoidella elegans

Kwon & Lee, 1979

18164D73-667D-5AAB-A5BB-88DFD733FB3F

##### Notes

Nam et al. (2018)

#### Petalocephala manchurica

Kato, 1932

39E763DB-0337-5A9E-AD63-B081A6EE1435

##### Notes

Lim et al. (2013)

#### Phlogotettix cyclops

(Mulsant & Rey, 1855)

10804D31-E3C8-58A7-A55D-BB8DC51A1927

##### Notes

Kwon et al. (1996), Lee and Jung (2001), Won et al. (2023)

#### Planaphrodes sahlbergii

(Signoret, 1879)

7AA7CADE-A9E9-5595-AEFE-87CCB1FDE447

##### Notes

Lee and Kwon (1981), ME (1993), Kwon et al. (1996), Lee and Jung (2001)

#### Psammotettix striata

(Linnaeus, 1758)

0FC04420-73B5-5725-8ED8-0E5C8FFA2C86

##### Notes

Kwon et al. (1996), Lee and Jung (2001)

#### Recilia coronifer

(Marshall, 1866)

978B52E9-E0C6-5CE5-A38D-508F1FB994A1

##### Notes

Lee and Kwon (1981), ME (1993), Kwon et al. (1996), Lee and Jung (2001)

#### Scaphoideus festivus

Matsumura, 1902

02CEFD3C-4CE5-5C4C-8D77-E0815FF003B8

##### Notes

Lee and Kwon (1981), ME (1993), Kwon et al. (1996), Lee and Jung (2001)

#### Scleroracus flavopictus

(Ishihara, 1953)

3A3B07C8-EA56-51EA-B76C-60979DC74C8A

##### Notes

Kwon et al. (1996), Lee and Jung (2001)

#### Stirellus grandis

(Matsumura, 1914)

3B8816F1-2DF1-5FA8-911C-1D1C52AF9DB5

##### Notes

Lee and Kwon (1981), ME (1993), Kwon et al. (1996), Lee and Jung (2001)

#### Stirellus productus

(Matsumura, 1902)

4B515AA4-0396-55A5-A0D6-24761CACE277

##### Notes

Lee and Kwon (1981), ME (1993), Kwon et al. (1996), Lee and Jung (2001)

#### Takagiella tezuyae

(Matsumura, 1902)

DDD3BC1B-324B-596D-9BC2-2F1AEBA852B7

##### Notes

Nam et al. (2018)

#### Thagria satsumensis

(Matsumura, 1914)

1667D451-5558-51B3-AA9F-8A8EA2C46DEA

##### Notes

Lee and Kwon (1981), ME (1993), Kwon et al. (1996), Lee and Jung (2001)

#### Xestocephalus guttulatus

(Motschulsky, 1859)

8E1293AD-DF8A-5169-B88C-B8BD5C7CE7A2

##### Notes

Lee and Kwon (1981), ME (1993), Kwon et al. (1996), Lee and Jung (2001)

#### Cicadidae

C8605658-0186-5CBD-8D09-116E85F82011

#### Meimuna mongolica

(Distant, 1881)

47AEC643-58EA-53F3-9077-6ED40D9C3FD1

##### Notes

Cho (1955), Cho (1965), Lee and Kwon (1981), ME (1993), Kwon et al. (1996), Lee and Jung (2001)

#### Meimuna opalifera

(Walker, 1850)

5EE1B986-5B2B-5CB4-B512-A2AEDB0D2D80

##### Notes

Cho (1955), Cho (1965), Lee and Kwon (1981), ME (1993), Kwon et al. (1996), Lee and Jung (2001), Lim et al. (2013), HNIBR (2022), Won et al. (2023)

#### Platypleura kaempferi

(Fabricius, 1794)

CEA3375A-0C1F-5FE1-BBDB-50A6D4B51B38

##### Notes

Cho (1955), Cho (1965), Lee and Kwon (1981), ME (1993), Kwon et al. (1996), Lee and Jung (2001)

#### Cimicidae

C60F0BCD-8EE2-5949-97E1-E87D5C5C980D

#### Cimex lectularius

Linnaeus, 1758

F99BAA54-95D3-5BDC-BC5B-C80AA4082ACC

##### Notes

Cho (1965), Lee and Kwon (1981), ME (1993), Kwon et al. (1996), Lee and Jung (2001)

#### Coccidae

F3E0FF89-3917-5836-9DC0-19A2B514C361

#### Ceroplastes ceriferus

(Fabricius, 1798)

2B966BE7-3334-58B9-8451-5A9792A9C9C5

##### Notes

Lee and Choi (2019), HNIBR (2022)

#### Metaceronema japonica

(Maskell, 1897)

AD56D45D-A53F-5057-94C4-C37E323DB911

##### Notes

HNIBR (2022)

#### Coreidae

C09BE007-E359-5E42-AD47-E3E095D7113B

#### Cletus punctiger

(Dallas, 1852)

BF8C6663-A9B5-5DFE-9105-1958F6A1782D

##### Notes

Info (2023)

#### Cletus schmidti

Kiritshenko, 1916

3F52CB17-0CFC-5F26-B186-EBAAE2A4F4F4

##### Notes

Kwon et al. (1996), Lee and Jung (2001), Lim and Lee (2012), HNIBR (2022)

#### Homoeocerus dilatatus

Horváth, 1879

0A3365F7-4FCE-54E4-8B86-26F3B717B1CD

##### Notes

Cho (1955), Cho (1965), Lee and Kwon (1981), ME (1993), Kwon et al. (1996), Lee and Jung (2001), Lim and Lee (2012), Lim et al. (2013), HNIBR (2022), Won et al. (2023)

#### Homoeocerus striicornis

Scott, 1874

343D77C7-D726-5AA3-AC78-C61CA05843D3

##### Notes

Lee and Kwon (1981), ME (1993), Kwon et al. (1996), Lee and Jung (2001)

#### Homoeocerus unipunctatus

(Thunberg, 1783)

CE12A132-F994-5C6D-AD7A-520C94B6F9BD

##### Notes

Kwon et al. (1996), Lee and Jung (2001), Lim and Lee (2012), HNIBR (2022), Won et al. (2023)

#### Hygia lativentris

(Motschulsky, 1866)

084C1FB1-846B-5551-A864-D197F954DAE4

##### Notes

Kim (1971), Lee and Kwon (1981), ME (1993), Kwon et al. (1996), Lee and Jung (2001), Lim and Lee (2012), Lim et al. (2013), HNIBR (2022)

#### Hygia opaca

(Uhler, 1860)

1499B635-0E51-53C6-8025-C45DE7B8902A

##### Notes

Cho (1955), Cho (1965), Lee and Kwon (1981), ME (1993), Kwon et al. (1996), Lee and Jung (2001)

#### Cydnidae

D56427ED-A193-5F3E-94A2-1BB17B396902

#### Adomerus triguttulus

(Motschulsky, 1866)

793D66BF-CEC0-5330-BF68-70D6873A6AD9

##### Notes

Lee and Kwon (1981), ME (1993), Kwon et al. (1996), Lee and Jung (2001), Lim et al. (2013)

#### Canthophorus niveimarginatus

Scott, 1874

73955E65-625D-514C-9727-C85009EC4F91

##### Notes

ME (1993), Kwon et al. (1996), Lee and Jung (2001)

#### Fromundus pygmaeus

(Dallas, 1851)

986CE7A0-B82F-522E-9783-4B5A55A6B63D

##### Notes

ME (1993), Kwon et al. (1996), Lee and Jung (2001), Lim and Lee (2012), HNIBR (2022), Won et al. (2023)

#### Macroscytus japonensis

Scott, 1874

CEDAA265-E929-5269-A7CC-E473B04DBE11

##### Notes

Lee and Kwon (1981), ME (1993), Kwon et al. (1996), Lee and Jung (2001), Lim and Lee (2012), Lim et al. (2013), Nam et al. (2018), HNIBR (2022), Won et al. (2023)

#### Microporus nigrita

(Fabricius, 1794)

99857131-1FDD-5BA4-A13E-141DA9B2C85A

##### Notes

Lee and Kwon (1981), ME (1993), Kwon et al. (1996), Lee and Jung (2001)

#### Delphacidae

F6C45015-3B4E-5E41-AB3F-922044C917F1

#### Epeurysa nawaii

Matsumura, 1900

531D8A4B-38DA-540C-B29A-CE340AE0B7AC

##### Notes

Kwon et al. (1996), Lee and Jung (2001)

#### Garaga nagaragawana

(Matsumura, 1900)

58B5CC03-D999-5BC4-8E42-0D15BE135CE5

##### Notes

Lee and Kwon (1981), ME (1993), Kwon et al. (1996), Lee and Jung (2001)

#### Laodelphax striatellus

(Fallén, 1826)

FA3181CC-E1F2-5C42-8370-716E62AA6C67

##### Notes

Lee and Kwon (1981), ME (1993), Kwon et al. (1996), Lee and Jung (2001), Lim et al. (2013)

#### Nilaparvata lugens

(Stål, 1854)

FC7E79A5-EBCF-586D-9246-B0544675B397

##### Notes

Lee and Kwon (1981), ME (1993), Kwon et al. (1996), Lee and Jung (2001)

#### Sogatella furcifera

(Horváth, 1899)

591D70CD-1D71-5563-93D5-8AAB99901088

##### Notes

Lee and Kwon (1981), ME (1993), Kwon et al. (1996), Lee and Jung (2001), Won et al. (2023)

#### Sogatella kolophon

(Kirkaldy, 1907)

D963E3FE-06AC-5072-886F-516D4CDA522A

##### Notes

Lee and Kwon (1981), ME (1993), Kwon et al. (1996), Lee and Jung (2001)

#### Stenocranus yasumatsui

Ishihara, 1952

E50546B8-C588-531F-967E-0D0814C112EA

##### Notes

Lee and Kwon (1981), ME (1993), Kwon et al. (1996), Lee and Jung (2001)

#### Terthron albovittatum

(Matsumura, 1900)

5F765CB4-2387-5239-8E0B-4BED2B6FE050

##### Notes

Lee and Kwon (1981), ME (1993), Kwon et al. (1996), Lee and Jung (2001)

#### Tropidocephala brunnipennis

Signoret, 1860

46476CDF-708E-5DF9-9485-E3BED86D4E20

##### Notes

Lee and Kwon (1981), ME (1993), Kwon et al. (1996), Lee and Jung (2001)

#### Tropidocephala nigra

(Matsumura, 1900)

1602B7F2-6346-56D1-B37A-DFB17085D869

##### Notes

Lee and Kwon (1981), ME (1993), Kwon et al. (1996), Lee and Jung (2001)

#### Unkanodes sapporonus

(Matsumura, 1935)

950D74DE-1C95-523F-A7E2-63213AA47F10

##### Notes

Lee and Kwon (1981), ME (1993), Kwon et al. (1996), Lee and Jung (2001)

#### Derbidae

C76B9A94-687D-52F6-8318-7692B3D8B041

#### Diostrombus politus

Uhler, 1896

833ACC63-30E5-5D1B-B0B3-BC02AD3EAF4E

##### Notes

Kwon (2022)

#### Diaspididae

C29F0435-D3AD-52A2-97F2-D0BC641CAEF9

#### Chrysomphalus bifasciculatus

Ferris, 1938

49CB76FF-77D0-544B-89AC-AC2907B087EC

##### Notes

Lee and Kwon (1981), ME (1993), Kwon et al. (1996), Lee and Jung (2001), HNIBR (2022)

#### Odonaspis secreta

(Cockerell, 1896)

655EBDDC-5F0F-5E6A-B9C6-3E1BA9454C5E

##### Notes

Lee and Kwon (1981), ME (1993), Kwon et al. (1996), Lee and Jung (2001)

#### Geocoridae

25A7CBD6-AFAA-5FD7-A111-E82585098C64

#### Geocoris proteus

Distant, 1883

F9933B25-16FE-5411-BF38-2F8210F4B94A

##### Notes

Kwon et al. (1996), Lee and Jung (2001)

#### Gerridae

11A5444C-7AF4-553B-9699-2BB1C3E7246B

#### Aquarius paludum paludum

(Fabricius, 1794)

2080F7B3-01BE-5398-9AFC-8EF744112105

##### Notes

Lim and Lee (2012), HNIBR (2022)

#### Gerris latiabdominis

Miyamoto, 1958

44C0D5E6-67A0-56BA-93A8-D1ABD70FBC2D

##### Notes

Kwon (2021)

#### Issidae

248EB0DD-FF7F-5210-95BB-5E9DB7E06126

#### Sarima amagisana

Melichar, 1906

7C4DB9B1-9FDB-553A-A30A-8A181A37A41C

##### Notes

Lee and Kwon (1981), ME (1993), Kwon et al. (1996), Lee and Jung (2001)

#### Liviidae

F2F45BAF-8735-5389-BC5E-578D98AF59AC

#### Livia jesoensis

Matsumura, 1908

1F2E5BC6-DA8D-58C9-9254-9A4299980246

##### Notes

Lee and Kwon (1981), ME (1993), Kwon et al. (1996), Lee and Jung (2001)

#### Lygaeidae

D74EC9C9-CCB0-5BA3-9AF0-D1710F5CC7EA

#### Lygaeus equestris

(Linnaeus, 1758)

BE902B2C-5BE0-5E8B-A55F-6720127EDD2B

##### Notes

Cho (1955), Cho (1965), Lee and Kwon (1981), ME (1993), Kwon et al. (1996), Lee and Jung (2001)

#### Lygaeus hanseni

(Jakovlev, 1883)

5A59AE8E-BF3E-556B-8A12-615C0C5E12B0

##### Notes

Lee and Kwon (1981), ME (1993), Kwon et al. (1996), Lee and Jung (2001)

#### Nysius plebeius

Distant, 1883

39CCCB53-5531-5930-9477-F937F092CEBF

##### Notes

Kim (1971), Lee and Kwon (1981), ME (1993), Kwon et al. (1996), Lee and Jung (2001), Lim and Lee (2012), HNIBR (2022), Won et al. (2023)

#### Pylorgus colon

(Thunberg, 1784)

B36133A2-B55C-5430-9E40-93E7F4B45B7D

##### Notes

ME (1993), Kwon et al. (1996), Lee and Jung (2001)

#### Pylorgus ishiharai

Hidaka & Izzard, 1960

AD8CF489-024C-5847-9213-469020044C41

##### Notes

Lee and Kwon (1981), ME (1993), Kwon et al. (1996), Lee and Jung (2001)

#### Tropidothorax cruciger

(Motschulsky, 1859)

66A24958-DF9D-5C53-99E9-85EE0718B809

##### Notes

Cho (1955), Cho (1965), Lee and Kwon (1981), ME (1993), Kwon et al. (1996), Lee and Jung (2001), Lim et al. (2013)

#### Malcidae

46FF2DDE-44F2-52F6-824B-F7DEEFC723AF

#### Chauliops fallax

Scott, 1874

AAEB931F-9475-5887-B3E5-32A13DC41651

##### Notes

ME (1993), Kwon et al. (1996), Lee and Jung (2001)

#### Miridae

6C1F04A3-59A4-526A-BABC-A9344B321434

#### Adelphocoris demissus

Horváth, 1905

6F42F29F-5846-540A-9A22-5A3711B802D9

##### Materials

- **Type status:** Other material. **Occurrence:** recordedBy: Lee, D. Y.; sex: 1 male; lifeStage: adult; occurrenceID: 84BB96EC-B0CE-551A-BAA8-73F7BDB6A43A; **Location:** island: Ulleungdo Island; country: Republic of Korea; stateProvince: Gyeongsangbuk-do; county: Ulleung-gun; locality: Taeharyeong, Namseo-ri, Seomyeon; **Event:** samplingProtocol: Sweeping net; eventDate: 8/19/2021

##### Notes

This is the first record of this species from Ulleungdo Island.

Distribution: Japan, Korea (Jung and Kim 2015)

#### Adelphocoris lineolatus

(Goeze, 1778)

8B9DFC3F-FFD7-5794-ABBD-F4DD92D9DDDC

##### Notes

Lee and Kwon (1981), ME (1993), Kwon et al. (1996), Lee and Jung (2001)

#### Adelphocoris suturalis

(Jakovlev, 1882)

60D07668-F25F-5E11-87FF-C0DA8F20CF1B

##### Notes

Kim (1971), Lee and Kwon (1981), ME (1993), Kwon et al. (1996), Lee and Jung (2001), Lim et al. (2013), HNIBR (2022)

#### Adelphocoris triannulatus

(Stål, 1858)

96CD473F-8D7A-5A8B-83A5-8C652218F23A

##### Notes

Lee and Kwon (1981), ME (1993), Kwon et al. (1996), Lee and Jung (2001)

#### Adelphocoris variabilis

(Uhler, 1897)

DCF00130-63D6-52A9-A544-AE26BECE54FC

##### Notes

ME (1993), Kwon et al. (1996), Lee and Jung (2001)

#### Alloeotomus chinensis

Reuter, 1903

3D43989E-D0B7-59FC-97D7-9390CA98B735

##### Notes

Lee and Kwon (1981), ME (1993), Kwon et al. (1996), Lee and Jung (2001)

#### Apolygus limbatus

(Fallén, 1807)

8410184C-A619-55DC-9C38-B20D14B88048

##### Notes

Kwon (2021)

#### Apolygus nigrovirens

(Kerzhner, 1988)

CE5CD12B-54E2-5A5C-B156-2E8111E91AF1

##### Notes

ME (1993), Kwon et al. (1996), Lee and Jung (2001)

#### Bryocoris montanus

Kerzhner, 1972

9919D7B8-EA9F-5272-A33F-9F994B5B3254

##### Notes

Won et al. (2023)

#### Campylomma lividicornis

Reuter, 1912

B7F41CBD-BE88-5E55-A6E1-E0B7E9C3091D

##### Notes

ME (1993), Kwon et al. (1996), Lee and Jung (2001)

#### Castanopsides potanini

(Reuter, 1906)

3D1C2907-BC5B-56C2-8DCA-08E016FF362D

##### Notes

Lim and Lee (2012), HNIBR (2022)

#### Charagochilus angusticollis

Linnavuori, 1961

E285803D-4CDE-5043-A032-76E0F34E36CF

##### Notes

Won et al. (2023)

#### Cimicicapsus koreanus

(Linnavuori, 1963)

C93F9336-CDBB-579E-A9AC-AB3ECDF98A8B

##### Notes

ME (1993), Kwon et al. (1996), Lee and Jung (2001)

#### Creontiades pacificus

(Stal, 1859)

09E336EB-7874-5FD0-82EA-4132285DD90F

##### Notes

Lee and Kwon (1981), ME (1993), Kwon et al. (1996), Lee and Jung (2001)

#### Cyrtorhinus lividipennis

Reuter, 1885

B58FBB7F-A05C-5AC8-B296-EFD09ED64C85

##### Notes

Kwon et al. (1996), Lee and Jung (2001)

#### Deraeocoris pulchellus

(Reuter, 1906)

D4799A54-227D-5AA7-A688-319789C3E360

##### Notes

Lim and Lee (2012), HNIBR (2022)

#### Ectmetopterus micantulus

(Horváth, 1905)

2122D170-DB4A-5CA9-891F-038743CCD493

##### Notes

Lee and Kwon (1981), ME (1993), Kwon et al. (1996), Lee and Jung (2001)

#### Europiella artemisiae

(Becker, 1864)

5EFAFC1C-93D9-520D-9384-7B736CACB41C

##### Notes

ME (1993), Kwon et al. (1996), Lee and Jung (2001)

#### Eurystylus coelestialium

(Kirkaldy, 1902)

48B6BE64-85F9-5979-A452-0D80954D6924

##### Notes

Lee and Kwon (1981), ME (1993), Kwon et al. (1996), Lee and Jung (2001)

#### Kasumiphylus kyushuensis

(Linnavuori, 1961)

B1B8A3D7-F07A-51A7-81C1-49148A1351E6

##### Notes

ME (1993), Lee and Jung (2001)

#### Lygocoris idoneus

(Linnavuori, 1963)

1597DF38-8BD4-54CD-BCDF-28092EC04D63

##### Notes

Lee and Kwon (1981), ME (1993), Kwon et al. (1996), Lee and Jung (2001)

#### Lygus rugulipennis

Poppius, 1911

AD4BEE6C-E49E-5ADC-9715-E749264AF4DC

##### Notes

Kim (1971), Lee and Kwon (1981), ME (1993), Kwon et al. (1996), Lee and Jung (2001), HNIBR (2022)

#### Moissonia befui

Yasunaga, 1999

5896795F-63F7-58A6-8F84-7CCDAABA9F20

##### Notes

Lee et al. (2018)

#### Monalocoris filicis

(Linnaeus, 1758)

AFC27C38-2AA6-5D2D-BCB5-B7ED584A5769

##### Notes

Kwon (2021), Won et al. (2023)

#### Neolygus hakusanensis

(Yasunaga, 1991)

74EB093B-68A5-574C-8FF9-BD5F0C237B66

##### Notes

HNIBR (2022)

#### Nesidiocoris tenuis

(Reuter, 1895)

948CE015-6DF1-5958-B856-B4A2B613CF42

##### Notes

Lee and Kwon (1981), ME (1993), Kwon et al. (1996), Lee and Jung (2001)

#### Orthocephalus funestus

Jakovlev, 1881

29C23558-70C2-5859-9C62-9C4E49720E46

##### Notes

Lee and Kwon (1981), ME (1993), Kwon et al. (1996), Lee and Jung (2001)

#### Orthops scutellatus

Uhler, 1877

1B30ADFE-2246-5580-98BC-46E76D5E5A40

##### Notes

ME (1993), Kwon et al. (1996), Lee and Jung (2001)

#### Orthotylus flavosparsus

(Sahlberg, 1841)

1C9F19A9-5CA7-5DC0-BE52-5E0E51FB7ECE

##### Notes

Lee and Kwon (1981), ME (1993), Kwon et al. (1996), Lee and Jung (2001)

#### Pilophorus miyamotoi

Linnavuori, 1961

EBEB4285-A019-5410-B113-0A9D5ECAEF6B

##### Notes

ME (1993), Kwon et al. (1996), Lee and Jung (2001)

#### Pilophorus typicus

(Distant, 1909)

960CE178-1ECB-5271-8E1C-94B3A0FBFC1A

##### Notes

ME (1993), Kwon et al. (1996), Lee and Jung (2001)

#### Proboscidocoris malayus

Reuter, 1908

F1C201B3-863E-5FA8-B6BE-374D83F1E81A

##### Notes

Lee and Kwon (1981)

#### Proboscidocoris varicornis

(Jakovlev, 1904)

F3C9F0E4-2630-5292-8DC9-DD34DC8317DA

##### Notes

ME (1993), Kwon et al. (1996), Lee and Jung (2001)

#### Stenodema calcarata

(Fallén, 1807)

90264ACD-5CEE-55D3-9DAF-BCA8076EDCEF

##### Notes

Lee and Kwon (1981), ME (1993), Kwon et al. (1996), Lee and Jung (2001)

#### Stenodema rubrinerve

Horváth, 1905

C5B0D6EA-002C-51FB-862B-E5EC6B98DE8F

##### Notes

ME (1993), Kwon et al. (1996), Lee and Jung (2001)

#### Trigonotylus caelestialium

(Kirkaldy, 1902)

B178739C-6A12-500A-8680-CB00D983C890

##### Notes

Lee and Kwon (1981), ME (1993), Kwon et al. (1996), Lee and Jung (2001)

#### Tytthus chinensis

(Stål, 1859)

8695CE72-0814-5E1D-A932-BB4AACC2D58C

##### Notes

Lee et al. (2018)

#### Nabidae

C2B4ED29-25BB-58A3-96F3-53CE710A90FA

#### Gorpis brevilineatus

(Scott, 1874)

7F84FA4B-34A0-542E-8571-B8C408DF6700

##### Notes

Lee and Kwon (1981), ME (1993), Kwon et al. (1996), Lee and Jung (2001), Lim and Lee (2012), Lim et al. (2013), HNIBR (2022)

#### Himacerus apterus

(Fabricius, 1798)

14F0060C-FCF0-5EB5-AC58-3DF29D7DF726

##### Notes

Lee and Kwon (1981), ME (1993), Kwon et al. (1996), Lee and Jung (2001)

#### Nabis apicalis

Matsumura, 1913

EF67A075-E9F5-5743-AB5A-62B5FA25D73F

##### Notes

Lee and Kwon (1981), ME (1993), Kwon et al. (1996), Lee and Jung (2001), Lim and Lee (2012), HNIBR (2022), Won et al. (2023)

#### Nabis reuteri

Jakovlev, 1876

32C7AD8F-92CD-51A8-ABDE-9CCCCEEB8248

##### Notes

ME (1993), Kwon et al. (1996), Lee and Jung (2001)

#### Nabis stenoferus

Hsiao, 1964

D316D1A0-4420-5492-96D6-A946C6D76749

##### Notes

Lee and Kwon (1981), ME (1993), Kwon et al. (1996), Lee and Jung (2001), Won et al. (2023)

#### Prostemma hilgendorfii

Stein, 1878

18C169CD-34C9-5CCB-9686-22D38F58F41C

##### Notes

ME (1993), Kwon et al. (1996), Lee and Jung (2001)

#### Stenonabis yasumatsui

Miyamoto and Lee, 1966

5B60ADD8-607D-5D35-9976-F0D26600686C

##### Notes

Lee and Kwon (1981), ME (1993), Kwon et al. (1996), Lee and Jung (2001)

#### Nepidae

5D8ED47F-8BF2-51AC-8CDD-74D618D04922

#### Laccotrephes japonensis

Scott, 1874

C0397AEC-B23F-5598-AC7B-C77167568CC9

##### Notes

Choi (2023)

#### Pachygronthidae

79A13411-D392-5A8F-863A-84F579E1B277

#### Pachygrontha antennata

(Uhler, 1860)

9877048C-6C6E-56B6-B32B-74E8EC1E5158

##### Notes

Lim and Lee (2012), Lim et al. (2013), HNIBR (2022)

#### Pentatomidae

DA1A6DEA-E35E-5EBA-B134-E3B12548DDE2

#### Aelia fieberi

Scott, 1874

4BFC269A-CD83-5CF9-A663-69B6E4E876B5

##### Notes

Lee and Kwon (1981), ME (1993), Kwon et al. (1996), Lee and Jung (2001), Lim et al. (2013), Won et al. (2023)

#### Aelia klugii

Hahn, 1833

02C51C77-B9B1-54F6-BF0D-6AF7D0831732

##### Notes

Won et al. (2023)

#### Arma custos

(Fabricius, 1794)

018DD1A5-0D32-5AC8-B90D-7898FD2FECD8

##### Notes

Kwon (2021), HNIBR (2022)

#### Carbula putoni

(Jakovlev, 1876)

A8E5FEE7-E0BA-5AC8-A03A-CC47BA86BD18

##### Notes

Lim et al. (2013)

#### Carpocoris purpureipennis

(DeGeer, 1773)

D0792727-EF71-5ED0-82A5-0E6A826678F2

##### Notes

ME (1993), Kwon et al. (1996), Lee and Jung (2001)

#### Dolycoris baccarum

(Linnaeus, 1758)

957084EE-2638-5083-B9DA-0252CBE3E040

##### Notes

Cho (1955), Cho (1965), Lee and Kwon (1981), ME (1993), Kwon et al. (1996), Lee and Jung (2001), Lim and Lee (2012), Lim et al. (2013), HNIBR (2022)

#### Dybowskyia reticulata

(Dallas, 1851)

D41B216F-D154-52DA-892F-3B88CDF1DB39

##### Notes

Lee and Kwon (1981), ME (1993), Kwon et al. (1996), Lee and Jung (2001), Lim and Lee (2012), HNIBR (2022)

#### Eurydema gebleri gebleri

Kolenati, 1846

9A1017C3-5CC7-5AD0-810E-3FE16D302BEB

##### Notes

Lee and Jung (2001), Lim and Lee (2012), Lim et al. (2013), HNIBR (2022)

#### Eurydema rugulosa

(Dohrn, 1860)

B579194F-0DE4-5AB4-867A-24F5B46A1002

##### Notes

Cho (1955), Cho (1965), Lee and Kwon (1981), ME (1993), Kwon et al. (1996), Lee and Jung (2001)

#### Eysarcoris aeneus

(Scopoli, 1763)

95B06C7A-B12D-5775-9CFD-E455AE5A5CD4

##### Notes

Kwon (2021)

#### Eysarcoris annamita

Breddin, 1909

71502416-8EA4-52F4-9294-FF460788A170

##### Notes

Lim and Lee (2012), HNIBR (2022)

#### Eysarcoris guttigerus

(Thunberg, 1783)

AB683B8E-0449-5F2D-9B7D-E692000C9ECE

##### Notes

Lee and Jung (2001), Lim and Lee (2012), HNIBR (2022)

#### Eysarcoris ventralis

(Westwood, 1837)

0DFBDF20-11F4-5B5E-B337-E313CD39252A

##### Notes

ME (1993), Kwon et al. (1996), Lee and Jung (2001)

#### Glaucias subpunctatus

(Walker, 1867)

F03E8852-3128-5414-A74D-8791CB7F843F

##### Notes

Won et al. (2023)

#### Gonopsis affinis

(Uhler, 1860)

064E52A0-9AD8-50D4-9CFE-8EA5CD878CCE

##### Notes

Kwon (2022)

#### Halyomorpha halys

(Stål, 1855)

59FDB148-CAFC-5BDA-96D9-4A92AC0D890A

##### Notes

Kim (1971), Lee and Kwon (1981), ME (1993), Kwon et al. (1996), Lee and Jung (2001), Lim and Lee (2012), Lim et al. (2013), HNIBR (2022)

#### Homalogonia obtusa obtusa

(Walker, 1868)

2190B194-32F8-51F4-B08E-59CBD9B8EAC4

##### Notes

Lee and Kwon (1981), ME (1993), Kwon et al. (1996), Lee and Jung (2001), Lim and Lee (2012), HNIBR (2022)

#### Lelia decempunctata

(Motschulsky, 1860)

9AB97981-4F4B-542F-A5B6-77D8DC571F1F

##### Notes

Lee and Kwon (1981), ME (1993), Kwon et al. (1996), Lee and Jung (2001), Lim et al. (2013), Roca-Cusachs and Jung (2020), Won et al. (2023)

#### Menida disjecta

(Uhler, 1860)

3CA857DC-ABF0-5324-8A2F-7F4CCF77CD95

##### Notes

Lee and Kwon (1981), ME (1993), Kwon et al. (1996), Lee and Jung (2001), Lim and Lee (2012), Lim et al. (2013), HNIBR (2022), Won et al. (2023)

#### Menida musiva

(Jakovlev, 1876)

71F95A5A-A077-5DDA-9104-9865BFF04B4B

##### Notes

Lee and Jung (2001), Lim et al. (2013)

#### Menida violacea

Motschulsky, 1861

9BD5E72B-BAFB-5274-A413-2865B035744A

##### Notes

Cho (1955), Cho (1965), Lee and Kwon (1981), ME (1993), Kwon et al. (1996), Lee and Jung (2001), Lim and Lee (2012), Lim et al. (2013), HNIBR (2022)

#### Nezara antennata

Scott, 1874

7F564EA7-7051-5A75-9A56-46EC2E52254F

##### Notes

ME (1993), Kwon et al. (1996), Lee and Jung (2001), Lim et al. (2013), HNIBR (2022)

#### Nezara viridula

(Linnaeus, 1758)

DA890D25-5AE6-5041-8395-D437C0C8D394

##### Notes

ME (1993), Kwon et al. (1996), Lee and Jung (2001)

#### Pentatoma japonica

(Distant, 1882)

58DA16D2-1818-541E-AC64-292FE1708BD9

##### Notes

Lim et al. (2013), HNIBR (2022)

#### Picromerus lewisi

Scott, 1874

B916A4A9-CE57-545E-BCFA-C0E65825F142

##### Notes

Lee and Kwon (1981), ME (1993), Kwon et al. (1996), Lee and Jung (2001)

#### Piezodorus hybneri

(Gmelin, 1790)

E189EC76-63F4-5DF7-BC98-71D6D4CA069D

##### Notes

Lee and Kwon (1981), ME (1993), Kwon et al. (1996), Lee and Jung (2001)

#### Pinthaeus sanguinipes

(Fabricius, 1781)

6BB73DFD-2EA0-54D6-A259-BCA375923D43

##### Notes

Kwon (2021)

#### Plautia splendens

(Distant, 1900)

7B498DA6-A590-5D5A-A6DC-D7E4FBFD28AE

##### Notes

Lee and Kwon (1981), ME (1993), Kwon et al. (1996), Lee and Jung (2001)

#### Plautia stali

Scott, 1874

DD41FE86-0E7E-5603-8F0D-B9E94BAE9C00

##### Notes

Cho (1955), Cho (1965), Kim (1971), Lee and Kwon (1981), ME (1993), Kwon et al. (1996), Lee and Jung (2001), Lim and Lee (2012), Lim et al. (2013), HNIBR (2022), Won et al. (2023)

#### Zicrona caerulea

(Linnaeus, 1758)

FFB3969F-DC72-59A1-A222-7B009AEE04A9

##### Notes

Kim (1971), Lee and Kwon (1981), ME (1993), Kwon et al. (1996), Lee and Jung (2001), Lim and Lee (2012), Lim et al. (2013), HNIBR (2022), Won et al. (2023)

#### Piesmatidae

7D73807F-8B93-5C20-8D26-767BF3DB3F58

#### Piesma maculata

(Laporte, 1833)

2C2A1042-0139-59B0-8B4E-2D51E032E10E

##### Notes

Lee and Kwon (1981), ME (1993), Kwon et al. (1996), Lee and Jung (2001)

#### Plataspididae

48D7929E-1331-5D28-84E5-D04778721CB5

#### Coptosoma biguttulumm

Motschulsky, 1860

CA450C27-F90B-5BD3-B6DC-79CE3430F3C7

##### Notes

Cho (1955), Cho (1965), Lee and Kwon (1981), ME (1993), Kwon et al. (1996), Lee and Jung (2001)

#### Coptosoma japonicum

Matsumura, 1913

1583F2AC-894D-59F5-902A-6685402E86AD

##### Notes

Lee and Kwon (1981), ME (1993), Kwon et al. (1996), Lee and Jung (2001)

#### Megacopta punctatissima

(Montandon, 1894)

D62015E5-0415-58B2-A9CA-BAA2DEE89ED1

##### Notes

Lee and Kwon (1981), ME (1993), Kwon et al. (1996), Lee and Jung (2001), Lim and Lee (2012), Lim et al. (2013), HNIBR (2022)

#### Psyllidae

612BCAFF-B0E4-5684-88B1-24C1E034E27A

#### Acizzia jamatonica

(Kuwayama, 1908)

538D9171-AA54-5B5B-B95E-F495FA3DA63A

##### Notes

Lee and Kwon (1981), ME (1993), Kwon et al. (1996), Lee and Jung (2001)

#### Anomoneura mori

Schwarz, 1896

68F9C16B-8AC3-5320-82FE-3CDBD57F43FA

##### Notes

Lee and Kwon (1981), ME (1993), Kwon et al. (1996), Lee and Jung (2001)

#### Cacopsylla albopontis

(Kuwayama, 1908)

BD4D6AA8-A957-5EF7-8DCD-814ACF585E1A

##### Notes

Lee and Kwon (1981), ME (1993), Kwon et al. (1996), Lee and Jung (2001)

#### Cacopsylla fulguralis

(Kuwayama, 1908)

E33667A0-E2C3-56D9-AD39-82D715D16DF2

##### Notes

Lee and Kwon (1981), ME (1993), Kwon et al. (1996), Lee and Jung (2001)

#### Cacopsylla hederae

(Miyatake, 1964)

55B1B2FA-A572-5A56-89D1-DBE24F40968E

##### Notes

Kwon et al. (1996), Lee and Jung (2001)

#### Cacopsylla pseudosieboldiani

(Konovalova & Loginova, 1985)

4412DDB4-4259-5A0E-B41F-EEE870C8FA32

##### Notes

Kwon and Kwon (2020)

#### Cacopsylla ulleungensis

(Kwon, 1983)

1B95C3F2-B00D-5094-B466-AFE2C6D0972A

##### Notes

Kwon et al. (1996), Lee and Jung (2001)

#### Pyrrhocoridae

DBC13BE0-4FEE-527C-BA0B-F051E30DB694

#### Pyrrhocoris sibiricus

Kuschakewitsch, 1866

B14D4A0D-C36A-5562-9E77-85284C4532FB

##### Notes

Cho (1955), Cho (1965), Lee and Kwon (1981), ME (1993), Kwon et al. (1996), Lee and Jung (2001), Lim and Lee (2012), Lim et al. (2013), HNIBR (2022)

#### Reduviidae

FE21CFD5-289B-5A85-9169-74EBB40743F2

#### Gardena brevicollis

Stål, 1871

F449D136-82B5-511A-BB17-8E20986873DD

##### Notes

Won et al. (2023)

#### Haematoloecha nigrorufa

(Stål, 1867)

07D46B7D-DF10-5B75-A904-27D6D9072772

##### Notes

NSMK (2023b)

#### Haematoloecha rufithorax

(Breddin, 1903)

3CDD85E8-A896-5FA8-917D-F6F995CE6028

##### Notes

Lim and Lee (2012), HNIBR (2022)

#### Sphedanolestes impressicollis

(Stål, 1861)

A2B70F74-8852-5691-9975-57A37F4EFF1B

##### Notes

Lee and Jung (2001)

#### Velinus nodipes

(Uhler, 1860)

897064CA-EA67-56B0-87EA-E2DF977E11C0

##### Notes

Lim and Lee (2012), HNIBR (2022)

#### Rhopalidae

7611143F-110D-5C71-83BB-42367647E82D

#### Liorhyssus hyalinus

(Fabricius, 1794)

10210641-2625-5228-A085-56C5B5D9E0EB

##### Notes

Lee and Kwon (1981), ME (1993), Kwon et al. (1996), Lee and Jung (2001), Lim et al. (2013)

#### Rhopalus maculatus

(Fieber, 1837)

813B71C9-0CC8-5FF2-942D-DED2D500FF9F

##### Notes

ME (1993), Kwon et al. (1996), Lee and Jung (2001), Lim and Lee (2012), HNIBR (2022)

#### Rhopalus sapporensis

(Matsumura, 1905)

715E3F39-5743-528E-9121-796E29E69B29

##### Notes

Lee and Kwon (1981), ME (1993), Kwon et al. (1996), Lee and Jung (2001), Lim and Lee (2012), Lim et al. (2013), HNIBR (2022)

#### Stictopleurus crassicornis

(Linnaeus, 1758)

66AA401C-1AB3-5B1F-84EA-86064D244118

##### Notes

Lee and Kwon (1981), ME (1993), Kwon et al. (1996), Lee and Jung (2001), Lim et al. (2013)

#### Stictopleurus minutus

Blöte, 1934

6AD08D4C-2958-53C2-A8F8-934B7CE2D352

##### Notes

Lim and Lee (2012), HNIBR (2022)

#### Rhyparochromidae

948760CD-740B-5C04-BBC1-8E140DD50623

#### Horridipamera lateralis

(Scott, 1874)

8421F023-5813-56F7-AAF2-08166A357AD3

##### Notes

ME (1993), Kwon et al. (1996), Lee and Jung (2001), Lim et al. (2013)

#### Horridipamera nietneri

(Dohrn, 1860)

B64AF346-ABCC-5E88-B600-C24A713BBA10

##### Notes

Cho (1955)

#### Iodinus ferrugineus

Lindberg, 1927

D8CE400D-B9B9-5645-B374-BEDC665FA73F

##### Notes

Lim and Lee (2012), HNIBR (2022)

#### Metochus abbreviatus

(Scott, 1874)

A957FB58-C1A3-527F-9F95-BC0F091896CC

##### Notes

Lee and Kwon (1981), ME (1993), Kwon et al. (1996), Lee and Jung (2001), Lim et al. (2013)

#### Neolethaeus assamensis

(Distant, 1901)

6CE410F2-EAD1-58EF-9B93-93FA5C162CC9

##### Notes

ME (1993), Kwon et al. (1996), Lee and Jung (2001)

#### Neolethaeus dallasi

(Scott, 1874)

A2AB98A2-ABA9-5E18-9E2E-3A4621104A0A

##### Notes

Lee and Kwon (1981), ME (1993), Kwon et al. (1996), Lee and Jung (2001), Lim et al. (2013), Won et al. (2023)

#### Pamerarma picta

(Scott, 1880)

BEBFF7C6-FA10-5D98-A26A-006F7E55F2AF

##### Notes

ME (1993), Kwon et al. (1996), Lee and Jung (2001)

#### Panaorus albomaculatus

(Scott, 1874)

A0928E42-EB6D-5ABB-9B3D-968F8B6E7322

##### Notes

Cho (1955), Cho (1965), Kim (1971), Lee and Kwon (1981), ME (1993), Kwon et al. (1996), Lee and Jung (2001), HNIBR (2022)

#### Panaorus japonicus

(Stål, 1874)

7B604281-2EBA-5DB0-93DC-B0B96032D597

##### Notes

Lee and Kwon (1981), ME (1993), Kwon et al. (1996), Lee and Jung (2001), Lim and Lee (2012), HNIBR (2022)

#### Paradieuches dissimilis

(Distant, 1883)

4B7B0D16-EC98-51EC-BF9D-F0E5C9DB46E7

##### Notes

Lee and Kwon (1981), ME (1993), Kwon et al. (1996), Lee and Jung (2001), Lim and Lee (2012), HNIBR (2022)

#### Scudderocoris albomarginatus

(Scott, 1874)

66EC2E94-9B56-5C99-B55D-46EE455100DA

##### Notes

Lee and Jung (2001), Lim and Lee (2012), HNIBR (2022)

#### Stigmatonotum rufipes

(Motschulsky, 1866)

57C804BD-68B9-5B91-BD3C-10FCCA405440

##### Notes

Lee and Kwon (1981), ME (1993), Kwon et al. (1996), Lee and Jung (2001)

#### Ricaniidae

29910AC2-EBE5-510D-A7B0-DF102196A3E7

#### Pochazia shantungensis

(Chou & Lu, 1977)

A0490EC9-4D38-54AC-BBF5-E0373AD061D4

##### Notes

Kwon (2022)

#### Ricania simulans

(Walker, 1851)

BD31F411-E023-5491-A4CA-FCC89BEB5002

##### Notes

Kim (1971), Lee and Kwon (1981), ME (1993), Kwon et al. (1996), Lee and Jung (2001), Lim et al. (2013), HNIBR (2022), Won et al. (2023)

#### Ricania taeniata

Stål, 1870

086412B5-3705-56D9-8E5D-76EE16BC9EAB

##### Notes

Kwon (2022)

#### Saldidae

03CB54EC-B338-53D2-A25D-74E184BD6514

#### Saldula saltatoria

(Linnaeus, 1758)

06341215-E1A1-589F-AC3F-8A5DF9BEA5BA

##### Notes

Lee and Kwon (1981), ME (1993), Kwon et al. (1996), Lee and Jung (2001)

#### Tingidae

48769F78-5736-59C1-AE27-41F1558F956B

#### Cantacader lethierryi

Scott, 1874

65501904-3D55-5994-BD9B-BD05236A1D41

##### Notes

ME (1993), Kwon et al. (1996), Lee and Jung (2001)

#### Corythucha marmorata

(Uhler, 1878)

20B79A34-9567-5A2A-99A4-E425BC945DC5

##### Materials

- **Type status:** Other material. **Occurrence:** recordedBy: Lee, D. Y.; sex: 1 male; lifeStage: adult; occurrenceID: 5E841167-E3D2-5B73-A258-4B5808F9918D; **Location:** island: Ulleungdo Island; country: Republic of Korea; stateProvince: Gyeongsangbuk-do; county: Ulleung-gun; locality: Taeharyeong, Namseo-ri, Seomyeon; **Event:** samplingProtocol: Sweeping net; eventDate: 8/19/2021

##### Notes

This is the first record of this species from Ulleungdo Island.

Distribution: Canada, China, Italy, Jamaica, Japan, Korea, Mexico, USA (Li et al. 2024)

#### Galeatus spinifrons

(Fallén, 1807)

787FC9D7-3C6C-5979-AC94-59CA6F45DD84

##### Notes

Lee and Kwon (1981), ME (1993), Kwon et al. (1996), Lee and Jung (2001)

#### Physatocheila fieberi

(Scott, 1874)

E6B214BB-EAD0-5413-83C0-73791771BC08

##### Notes

Won et al. (2023)

#### Stephanitis fasciicarina

Takeya, 1931

328AE620-83A7-5EFF-872C-30A79154A7D7

##### Notes

Lee and Kwon (1981), ME (1993), Kwon et al. (1996), Lee and Jung (2001)

#### Stephanitis pyrioides

(Scott, 1874)

37D8E620-54D5-5B8F-A474-09A600073640

##### Notes

Lee and Kwon (1981), ME (1993), Kwon et al. (1996), Lee and Jung (2001)

#### Triozidae

9B439B67-CF67-5912-86CB-19141A17F7E5

#### Heterotrioza chenopodii

(Reuter, 1876)

F313F948-74C5-51D7-A6F5-53BDDCC3FF6D

##### Notes

Lee and Kwon (1981), ME (1993), Kwon et al. (1996), Lee and Jung (2001)

#### Trichochermes rhamnisugus

(Li, 1994)

3BF6DB13-EA46-54CA-A90F-10354700B4B8

##### Notes

Kwon and Lee (1979), Kwon et al. (1996), Lee and Jung (2001)

#### Veliidae

24C1132E-6D98-55B7-8760-B8F53313986D

#### Pseudovelia tibialis tibialis

Esaki & Miyamoto, 1955

6A9FADB0-3405-5945-AD11-E9AB13F7ABB7

##### Notes

ME (1993), Kwon et al. (1996), Lee and Jung (2001)

#### Hymenoptera

0717912E-BB9C-5B49-A1D9-EF1337BEC45C

#### Andrenidae

B3904104-E016-536A-A5F1-3CA310AD9CA1

#### Andrena dentata

Smith, 1879

1E27A9AB-2CC9-569D-A84F-ED704F2CE0DF

##### Notes

Kim (1971), Lee and Kwon (1981), ME (1993), Kwon et al. (1996), Lee and Jung (2001), HNIBR (2022)

#### Andrena kaguya

Hirashima, 1965

7671BE00-C0B1-52D2-96E9-A868C1DE1A03

##### Notes

Kwon (2022)

#### Andrena thoracica

(Fabricius, 1775)

D33BD328-1D07-542C-BEDD-763C8586ACA9

##### Notes

Kim (1971), Lee and Kwon (1981), ME (1993), Kwon et al. (1996), Lee and Jung (2001), Nam et al. (2018), HNIBR (2022)

#### Andrena tsukubana

Hirashima, 1957

E30D34E4-9BA0-5411-9023-A5A30E313BC0

##### Notes

Nam et al. (2018)

#### Panurginus crawfordi

Cockerell, 1914

B935EDA7-535E-53C0-8AC5-E5D7AC7321E2

##### Notes

Lee and Kwon (1981), ME (1993), Kwon et al. (1996), Lee and Jung (2001)

#### Apidae

39A259A7-B627-51EE-946A-7B891C04E016

#### Apis cerana

Fabricius, 1793

A5D547FE-136B-51C8-816D-8C6000B82AFD

##### Notes

Info (2023)

#### Apis mellifera

Linnaeus, 1758

D400D1D8-27F3-5236-B69F-51C859C77068

##### Notes

Kim (1971), Lee and Kwon (1981), ME (1993), Kwon et al. (1996), Lee and Jung (2001), Lim and Lee (2012), Lim et al. (2013), HNIBR (2022), Won et al. (2023)

#### Bombus ardens ardens

(Smith, 1879)

DDA2A9B4-61C3-5EA0-9D4B-B23C6150141F

##### Notes

Cho (1955), Cho (1965), Lee and Kwon (1981), ME (1993), Kwon et al. (1996), Lim and Lee (2012), Lim et al. (2013), HNIBR (2022), Won et al. (2023)

#### Bombus ardens ullungensis

Kim & Kim 1993

A14D67F1-013E-582A-9EAA-9FF581A92C8E

##### Notes

Kwon et al. (1996), Lee and Jung (2001)

#### Bombus diversus

Smith, 1869

6E1D2293-6850-5AD7-B33C-569C364F77F6

##### Notes

Lee and Jung (2001)

#### Bombus hypocrita

Pérez, 1905

B6C9F1FB-BECD-5553-A7A7-7B6A0E0D33C9

##### Notes

Lee and Kwon (1981), ME (1993), Kwon et al. (1996), Lee and Jung (2001)

#### Bombus ignitus

(Smith, 1869)

C4FFD945-36DD-5542-9E97-F18A3D9E89B0

##### Notes

Cho (1955), Cho (1965), Kim (1971), Lee and Kwon (1981), ME (1993), Kwon et al. (1996), Lee and Jung (2001), Lim and Lee (2012), Lim et al. (2013), HNIBR (2022), Won et al. (2023)

#### Bombus koreanus

(Skorikov, 1933)

CEB7AC2A-9829-5845-8477-10480B5C8CF8

##### Notes

Kim (1971), Lee and Kwon (1981), ME (1993), Kwon et al. (1996), Lee and Jung (2001), HNIBR (2022)

#### Ceratina flavipes

Smith, 1879

48835B00-41CF-5870-8D6A-721E12AFF509

##### Notes

Kim (1971), Lee and Kwon (1981), ME (1993), Kwon et al. (1996), Lee and Jung (2001), Lim et al. (2013), HNIBR (2022)

#### Ceratina japonica

Cockerell, 1911

80B64ED6-E8DF-589A-B53F-5C442B6A6018

##### Notes

Lee and Kwon (1981), ME (1993), Kwon et al. (1996), Lee and Jung (2001), Lim et al. (2013)

#### Eucera spurcatipes

(Perez, 1911)

3A339FAE-942B-517F-A771-A92B2C11F16B

##### Notes

Cho (1955), Cho (1965), Lee and Kwon (1981), ME (1993), Kwon et al. (1996), Lee and Jung (2001)

#### Xylocopa appendiculata circumvolans

Smith, 1852

973EEED7-7563-5F61-B83E-4F495B029213

##### Notes

Cho (1955), Cho (1965), Lee and Kwon (1981), ME (1993), Kwon et al. (1996), Lee and Jung (2001), Lim and Lee (2012), Lim et al. (2013), HNIBR (2022)

#### Argidae

EE456520-FE97-569E-A07E-DCA4D95DDA3A

#### Arge jonasi

(Kirby, 1882)

15D19C84-16B9-5AD3-AC92-FA63DF8A168B

##### Notes

Lee and Jung (2001)

#### Arge mali

(Uchiyama, 1906)

261EB214-A34D-5E3D-B73E-E2D87E55BCC5

##### Notes

Lee and Kwon (1981), ME (1993), Kwon et al. (1996), Lee and Jung (2001)

#### Arge pagana pagana

(Panzer, 1797)

8C6EC66F-C66E-5AC3-A493-30462D0CF236

##### Notes

HNIBR (2022)

#### Arge similis

(Vollenhoven, 1860)

B38B2E1D-8BF4-58EF-91E3-C3509577D87D

##### Notes

Kim (1971), Lee and Kwon (1981), ME (1993), Kwon et al. (1996), Lee and Jung (2001), HNIBR (2022)

#### Spinarge fulvicornis

(Mocsáry, 1909)

B4F8571A-DD12-5884-A740-272C58D9D7A4

##### Notes

Lee and Kwon (1981), ME (1993), Kwon et al. (1996), Lee and Jung (2001)

#### Braconidae

FAB43CCF-FF7D-5CD3-957B-F62698943432

#### Apanteles molestae

Muesebeck, 1933

B363F628-624D-5D68-A643-6B4141B20D2E

##### Notes

Kim (1971), Lee and Kwon (1981), ME (1993), Kwon et al. (1996), Lee and Jung (2001), HNIBR (2022)

#### Aphidius matricariae

Haliday, 1834

71375F8F-080E-5A49-9675-9D79EC61D0B0

##### Notes

Nam et al. (2018)

#### Aphidius salicis

Haliday, 1834

DF218641-DB7E-5277-9DEA-CFA0A14BDF7B

##### Notes

Nam et al. (2018)

#### Ascogaster hei

Tang & Marsh, 1994

66D69810-73EB-5DCD-8C07-681EA6621977

##### Notes

HNIBR (2022)

#### Chelonus munakatae

Matsumura, 1912

AF6ABA4B-FA29-5D59-84E1-A0EAD6EE9A6E

##### Notes

Lee and Kwon (1981), ME (1993), Kwon et al. (1996), Lee and Jung (2001)

#### Cotesia glomerata

(Linnaeus, 1758)

84FD43B5-97A7-5505-9B6C-98C7EF1147E7

##### Notes

Kim (1971), Lee and Kwon (1981), ME (1993), Lee and Jung (2001), HNIBR (2022)

#### Cotesia scabricula

(Reinhard, 1880)

E456F52F-1988-5F7D-9529-5348806B5593

##### Notes

Kim (1971), Lee and Kwon (1981), ME (1993), Kwon et al. (1996), Lee and Jung (2001), HNIBR (2022)

#### Habrobracon hebetor

(Say, 1836)

AAA6CE62-07F8-586A-BD32-688D3F537CCF

##### Notes

Lee and Jung (2001)

#### Macrocentrus linearis

(Nees, 1811)

102F7EBE-F4AB-5DF9-8433-338A2A457A6B

##### Notes

Lee and Kwon (1981), ME (1993), Kwon et al. (1996), Lee and Jung (2001)

#### Proterops basalis

Walker, 1874

C973BEDD-BAB7-5CCF-987F-31896CCAB592

##### Notes

Lee and Kwon (1981), ME (1993), Kwon et al. (1996), Lee and Jung (2001)

#### Xiphozele compressiventris

Cameron, 1906

B8F15652-4BF5-5108-8CF9-7CBED28AF5F8

##### Notes

Lee and Kwon (1981), ME (1993), Kwon et al. (1996), Lee and Jung (2001), Nam et al. (2018)

#### Chalcididae

8452A108-47A8-5D63-BFB2-80CF2CF7D08B

#### Brachymeria fiskei

(Crawford, 1910)

C451FD9E-D1FD-5357-B9E6-948DADB4D53D

##### Notes

Kim (1971), Lee and Kwon (1981), ME (1993), Kwon et al. (1996), Lee and Jung (2001), HNIBR (2022)

#### Brachymeria lasus

(Walker, 1841)

A4FEE1EA-7803-56B3-ADF9-B2D04E8E26A3

##### Notes

Lee and Kwon (1981), ME (1993), Kwon et al. (1996), Lee and Jung (2001)

#### Brachymeria minuta

(Linnaeus, 1767)

DD176E51-FAB2-5117-95C5-F53DE7B0E6A1

##### Notes

Lee and Kwon (1981), ME (1993), Kwon et al. (1996), Lee and Jung (2001)

#### Chrysididae

62A9A6A0-E14B-50A0-AC9B-8BEE8F7F9B64

#### Chrysis ignita

(Linnaeus, 1758)

A258CE84-40FF-5FBA-9487-45F44130F367

##### Notes

Kim (1971), Lee and Kwon (1981), ME (1993), Kwon et al. (1996), Lee and Jung (2001), HNIBR (2022)

#### Colletidae

C850D032-06F9-5F92-B27F-2D7FA7788C83

#### Colletes collaris

Dours, 1872

2D43FA38-BAA3-5591-8854-04BAB0A589F6

##### Notes

Lee and Kwon (1981), ME (1993), Kwon et al. (1996), Lee and Jung (2001)

#### Hylaeus perforatus

(Smith, 1873)

08FDD0B2-3C95-525D-A35F-D27149516607

##### Notes

Lee and Kwon (1981), ME (1993), Kwon et al. (1996), Lee and Jung (2001)

#### Crabronidae

7055A089-4191-5A58-BA60-41AEAE352566

#### Astata boops

(Schrank, 1781)

02035A65-2465-5A74-9F6C-E255464D9AAA

##### Notes

Lee and Kwon (1981), ME (1993), Kwon et al. (1996), Lee and Jung (2001)

#### Cerceris arenaria

(Linnaeus, 1758)

D6315B23-2D50-50A1-ACA1-D7952EB8ABAB

##### Notes

Lee and Kwon (1981), ME (1993), Kwon et al. (1996), Lee and Jung (2001)

#### Cerceris coreensis

Tsuneki, 1961

22454DCB-FC77-5D96-86CF-8736C8FB7176

##### Notes

Kim (1971), Lee and Kwon (1981), ME (1993), Kwon et al. (1996), Lee and Jung (2001), HNIBR (2022)

#### Cerceris hortivaga

Kohl, 1880

D5F358CD-9465-5ED4-94E2-C30DF34E1517

##### Notes

Kim (1971), Lee and Kwon (1981), ME (1993), Kwon et al. (1996), Lee and Jung (2001), HNIBR (2022)

#### Cerceris sobo

Yasumatsu & Okabe, 1936

71146CCE-CCFC-5D0C-BB68-A1BB2E3109B2

##### Notes

Lee and Kwon (1981), ME (1993), Kwon et al. (1996), Lee and Jung (2001)

#### Crossocerus vagabundus koreanus

Tsuneki, 1957

D5B9A7A7-5B05-5AA7-80F6-3D08D5F9959F

##### Notes

Cho (1955), Lee and Kwon (1981), ME (1993), Kwon et al. (1996)

#### Ectemnius continuus

(Fabricius, 1804)

9CE0C9F5-EA6E-5C25-A087-E8EDE4B2A06A

##### Notes

Lee and Kwon (1981), ME (1993), Kwon et al. (1996), Lee and Jung (2001)

#### Pemphredon diervillae

Iwata, 1933

466B3543-B7A6-5DF1-AE01-6734509D9C92

##### Notes

Nam et al. (2018)

#### Psenulus fuscipennis japonicus

Tsuneki, 1959

335D5F4A-FF8C-58BB-A089-3B710D6C8D75

##### Notes

Lim et al. (2013)

#### Tachysphex bengalensis

Cameron, 1889

C5357137-F6D9-5A8F-9AB7-DCCED34579C7

##### Notes

Lee and Kwon (1981), ME (1993), Kwon et al. (1996), Lee and Jung (2001)

#### Trypoxylon malaisei

Gussakovskij, 1932

E9C03E5B-1FFC-586E-BBF3-7C80E07C68C4

##### Notes

Lee and Jung (2001)

#### Eulophidae

21C81E04-2014-5BF1-872E-B5C5E1A0852E

#### Pediobius iwatai

Kamijo, 1983

10887C64-8A3F-54FA-9750-47A7DC87D4AB

##### Notes

Nam et al. (2018)

#### Eumenidae

D69798CE-A239-5D02-9089-51C24F082D24

#### Anterhynchium flavomarginatum flavomarginatum

(Smith, 1852)

292EA546-1F50-5156-A461-F583A600DDA5

##### Notes

Lee and Kwon (1981), ME (1993), Kwon et al. (1996), Lee and Jung (2001), Lim et al. (2013)

#### Anterhynchium flavopunctatum flavopunctatum

(Smith, 1852)

2ED54B0E-CCC0-5C25-87DA-40EEADF61963

##### Notes

Cho (1955), Cho (1965), Lee and Kwon (1981), ME (1993), Kwon et al. (1996), Lee and Jung (2001)

#### Euodynerus dantici violaceipennis

Giordani Soika, 1973

0DF83CCC-9D9D-551C-94AF-5691FF402E79

##### Notes

Lim et al. (2013)

#### Euodynerus quadrifasciatus atripes

Giordani Soika, 1976

90588B43-0D36-53D1-89D4-9E4EAE77A749

##### Notes

Lee and Kwon (1981), ME (1993), Kwon et al. (1996), Lee and Jung (2001)

#### Euodynerus seulii

(Radoszkowski, 1890)

403FED26-0EED-5D9B-B94F-B0F112CF4965

##### Notes

Kim (1971), Lee and Kwon (1981), ME (1993), Kwon et al. (1996), Lee and Jung (2001)

#### Orancistrocerus drewseni drewseni

(Saussure, 1857)

C6F4A7DF-5E5B-5D4D-B4EA-306F5A96CAD7

##### Notes

Lee and Kwon (1981), ME (1993), Kwon et al. (1996), Lee and Jung (2001)

#### Oreumenes decoratus

(Smith, 1852)

D867A5C9-918C-5A98-8146-BE813863426E

##### Notes

Cho (1955), Cho (1965), Lee and Kwon (1981), ME (1993), Kwon et al. (1996), Lee and Jung (2001)

#### Pararrhynchium paradoxum paradoxum

(Gussakovskij, 1932)

57B7BA65-0BC6-5958-B084-708B1462AFCC

##### Notes

Lee and Kwon (1981), ME (1993), Kwon et al. (1996), Lee and Jung (2001)

#### Polistes japonicus japonicus

Saussure, 1858

ED0AF6ED-4BE6-529F-B721-C46D643013FF

##### Notes

Cho (1955), Cho (1965), Lee and Kwon (1981), ME (1993), Kwon et al. (1996), Lee and Jung (2001)

#### Polistes jokahamae

Rodoszkowski, 1887

10B03C94-E0EC-59F3-8A1A-66E2B2ACB9D4

##### Notes

Kim (1971), Lee and Kwon (1981), ME (1993), Kwon et al. (1996), Lee and Jung (2001), HNIBR (2022)

#### Polistes mandarinus

Saussure, 1853

F3AA85D6-D2C7-5EF7-945F-6CBF02227F63

##### Notes

Cho (1955), Lee and Kwon (1981), ME (1993), Kwon et al. (1996), Lee and Jung (2001), Lim and Lee (2012), HNIBR (2022)

#### Polistes olivaceus

(Degeer, 1773)

F382153D-5E2B-54BC-A14F-8BAA7A56A2DA

##### Notes

Cho (1955), Cho (1965), Lee and Kwon (1981), ME (1993), Kwon et al. (1996), Lee and Jung (2001)

#### Polistes snelleni

De Saussure, 1862

B5E4D8D6-6274-5F68-96A3-0A3C2E488F97

##### Notes

Kim (1971), Lee and Kwon (1981), ME (1993), Kwon et al. (1996), Lee and Jung (2001), Lim et al. (2013), HNIBR (2022)

#### Rhynchium quinquecintum fukaii

Cameron, 1911

BF93F22E-12C3-5FFF-82CB-F60F52D728D9

##### Notes

Kim (1971), Lee and Kwon (1981), ME (1993), Kwon et al. (1996), Lee and Jung (2001)

#### Eupelmidae

63185B98-763C-5B71-A8EF-CD65F8035F42

#### Anastatus japonicus

Ashmead, 1904

DD762BAA-8CB6-513C-B283-8BD25D57FA61

##### Notes

Lee and Kwon (1981), ME (1993), Kwon et al. (1996), Lee and Jung (2001)

#### Mesocomys albitarsis

(Ashmead, 1904)

891C78DB-3B5F-52E4-858E-54445671AA1E

##### Notes

Kim (1971), Lee and Kwon (1981), ME (1993), Kwon et al. (1996), Lee and Jung (2001)

#### Formicidae

A2610229-F8C4-53EE-8996-EB214B93F88C

#### Brachyponera chinensis

(Emery, 1895)

7462496F-9673-540A-BC7B-AE6AB4F9323D

##### Notes

Choi (1996)

#### Camponotus itoi

Forel, 1912

DA6DD6A7-3AD0-5D0D-94A2-EA3C8626B272

##### Notes

Choi (1996), Won et al. (2023)

#### Camponotus japonicus

Mayr, 1866

BB8C4FC4-5D7F-5ABC-A2B6-511F9A365F2B

##### Notes

Cho (1965), Lee and Kwon (1981), ME (1993), Kwon et al. (1996), Lee and Jung (2001), Lim and Lee (2012), Lim et al. (2013), HNIBR (2022), Won et al. (2023)

#### Camponotus kiusiuensis

Santschi, 1937

AAC04EF4-0A4E-5538-9AF1-5BD17EF39ADF

##### Notes

HNIBR (2022), Won et al. (2023)

#### Camponotus ligniperda

(Latreille, 1802)

9D075848-8877-5AD0-B73C-191B4A142A26

##### Notes

Cho (1955), Lee and Kwon (1981), ME (1993), Kwon et al. (1996), Lee and Jung (2001)

#### Camponotus nipponicus

Wheeler, 1928

7EB8669F-B442-59C1-ACC7-5E6D2C014E11

##### Notes

Choi (1996)

#### Camponotus quadrinotatus

Forel, 1886

9FDA4A74-FDD2-5D3C-BCB8-0ED16D9F3843

##### Notes

Choi (1996), HNIBR (2022)

#### Crematogaster matsumurai

Forel, 1901

D9871173-96D7-54A4-8DEE-0C8CD771946A

##### Notes

Choi (1996)

#### Crematogaster osakensis

(Forel, 1901)

EE885D8F-E7AF-567C-807E-499F36BDF5BA

##### Notes

Lee and Kwon (1981), ME (1993), Kwon et al. (1996), Lee and Jung (2001), Lim et al. (2013)

#### Crematogaster teranishii

Santschi, 1930

AC6DBC7A-DCED-568E-B7C7-C84E1558A66D

##### Notes

Choi (1996)

#### Cryptopone sauteri

(Wheeler, 1906)

CC7253A1-9D37-5F34-A64B-ABCA32E58579

##### Notes

Choi (1996), HNIBR (2022), Won et al. (2023)

#### Dolichoderus sibiricus

Emery, 1889

4D3B7CB7-A35B-5B00-A715-01D8305F0AFC

##### Notes

Nam et al. (2018)

#### Formica japonica

(Motschulsky, 1866)

CF5F22BA-F10F-5778-BF28-5905E751CFD6

##### Notes

Kwon (2022)

#### Formica lemani

Bondroit, 1917

B7328B3B-38A7-5430-9681-9FBA8FA134D9

##### Notes

Won et al. (2023)

#### Formica rufa

Linnaeus, 1761

8B0B7F68-3BCF-5C19-BB41-72B34D621CC0

##### Notes

Lee and Kwon (1981), ME (1993), Kwon et al. (1996), Lee and Jung (2001)

#### Formica sanguinea

Latreille, 1798

BB706FDA-043D-5D2C-BC5D-4613863074FC

##### Notes

Nam et al. (2018)

#### Formica yessensis

Wheeler, 1913

C7C8DDEF-4C4C-558E-A4E9-804AFF67E9DB

##### Notes

Nam et al. (2018)

#### Hypoponera sauteri

Onoyama, 1989

38C2ADB9-8DB7-5E5D-A804-63D2F325BD3C

##### Notes

Choi (1996), HNIBR (2022)

#### Lasius alienus

(Förster, 1850)

843FB4EC-127F-5A01-A8DB-27F1E85478CD

##### Notes

Choi (1996), Lee and Jung (2001), Lim and Lee (2012), HNIBR (2022), Won et al. (2023)

#### Lasius brunneus

(Latreille, 1798)

E1D2E14E-01C5-5676-95F9-418C6AFF8225

##### Notes

Choi (1996)

#### Lasius flavus

(Fabricius, 1781)

2DDC769A-C0ED-58DC-98C8-2312DC2836D6

##### Notes

Choi (1996)

#### Lasius fuliginosus

(Latreille, 1798)

FA16EC8E-8A03-577F-A739-BDFA679D04FA

##### Notes

Choi (1996)

#### Lasius hayashi

Yamauchi & Hayashida, 1970

B7088809-677B-5ED9-B4D5-B814856F00E5

##### Notes

Won et al. (2023)

#### Lasius japonicus

Santschi, 1941

3BC9B11C-BEF1-5769-927B-49B54B23026E

##### Notes

Lee and Jung (2001)

#### Lasius meridionalis

(Bondroit, 1920)

C56D4BD6-4C94-5F3E-8A39-C944F71BDBEC

##### Notes

Choi (1996)

#### Lasius niger

(Linnaeus, 1758)

E75B9160-6531-5774-A5F8-AA8CAEA4D820

##### Notes

Choi (1996)

#### Lasius nipponensis

Forel, 1912

74AA70CC-B9D5-5D94-9892-CA4A4DECCF45

##### Notes

Choi (1996)

#### Lasius spathepus

Wheeler, 1910

3AECCE38-A4D6-5560-AC63-BB14EEE482BC

##### Notes

Choi (1996), Lee and Jung (2001), Lim and Lee (2012), HNIBR (2022), Won et al. (2023)

#### Lasius talpa

Wilson, 1955

37B23CAB-CB9C-5E13-B52F-30BA04543A70

##### Notes

Choi (1996)

#### Lasius umbratus

(Nylander, 1846)

1BFDF4EF-EC5A-5155-AC9B-948510D9E76D

##### Notes

Lee and Kwon (1981), ME (1993), Kwon et al. (1996), Lee and Jung (2001)

#### Leptothorax acervorum

(Fabricius, 1793)

DF09E259-4566-5A53-997E-39542FF16E69

##### Notes

Nam et al. (2018)

#### Messor aciculatus

(Smith, 1874)

718924C6-0D9D-5AD6-8A71-16B8D89ABD2E

##### Notes

Nam et al. (2018)

#### Monomorium chinense

(Santschi, 1925)

FD3307A3-0AF8-5047-9B9C-808F96172A4A

##### Notes

Choi (1996)

#### Myrmecina nipponica

Wheeler, 1906

12D2DBD7-744C-5C46-BA5C-43A4F2C27CB8

##### Notes

Kim (1971), Lee and Kwon (1981), ME (1993), Kwon et al. (1996), Lee and Jung (2001), HNIBR (2022)

#### Myrmica lobicornis

Nylander, 1846

464A0215-63F6-57E0-ABA9-3EE2130AEAB9

##### Notes

Nam et al. (2018)

#### Myrmica rubra

(Linnaeus, 1758)

0E3C6FE8-CC1C-586D-9D34-9EC08D842215

##### Notes

Nam et al. (2018)

#### Myrmica sulcinodis

Nylander, 1846

49A85F18-0235-5BDD-8FDF-4E6D201A9E65

##### Notes

Nam et al. (2018)

#### Nylanderia flavipes

(Smith, 1874)

72CA5E2A-B1EE-5363-BEC0-7D26AB81FAF5

##### Notes

Choi (1996), Lee and Jung (2001), Lim and Lee (2012), HNIBR (2022), Won et al. (2023)

#### Nylanderia sakurae

(Ito, 1914)

F11EABAE-BE7F-5D55-ABA2-0932BF312FB2

##### Notes

Choi (1996)

#### Pheidole fervida

Smith, 1874

7E4632EB-5E40-5F60-ACFB-8B16375285EF

##### Notes

Kim (1971), Lee and Kwon (1981), ME (1993), Kwon et al. (1996), Lee and Jung (2001), Lim and Lee (2012), HNIBR (2022), Won et al. (2023)

#### Pheidole indica

Mayr, 1879

0BB98B77-BD05-540D-B456-94D0465FDA31

##### Notes

Choi (1996)

#### Pheidole noda

Smith, 1874

8BA87D1C-4E4C-578C-A574-5E7A5512F98B

##### Notes

Choi (1996)

#### Plagiolepis manczshurica

Ruzsky, 1905

007174B8-F188-5149-83FE-DA6500081C88

##### Notes

Choi (1996)

#### Ponera japonica

(Wheeler, 1906)

305A3050-A989-5D79-9B21-BD47846F247B

##### Notes

Choi (1996), HNIBR (2022)

#### Ponera scabra

Wheeler, 1928

C202C749-55D5-56F9-8C78-BD33CE7A817D

##### Notes

Info (2023)

#### Pristomyrmex punctatus

(Smith, 1860)

07916D06-B237-5EAF-8689-6E0C29FD5ACE

##### Notes

Choi (1996), Lee and Jung (2001), Lim and Lee (2012), HNIBR (2022), Won et al. (2023)

#### Proceratium itoi

(Forel, 1918)

B52289C4-BF0C-5D37-8C5A-0470244780B9

##### Notes

Choi (1996), HNIBR (2022)

#### Solenopsis japonica

Wheeler, 1928

85760252-C104-5302-9811-77F39134AA72

##### Notes

Choi (1996), Lee and Jung (2001)

#### Stenamma owstoni

Wheeler, 1906

5EA62C24-2C63-5C85-BBAE-649302A9FAFB

##### Notes

Nam et al. (2018), Won et al. (2023)

#### Stigmatomma silvestrii

Wheeler, 1928

E1E64A37-8374-5897-9335-92B4D9088FBF

##### Notes

Won et al. (2023)

#### Strumigenys incerta

(Brown, 1949)

57A0E05E-DAD6-5C86-B989-0C47A894C8E8

##### Notes

Choi (1996)

#### Strumigenys lewisi

Cameron, 1886

A0FC3711-F3D3-53DB-B179-07EECEEB11A8

##### Notes

Choi (1996)

#### Technomyrmex gibbosus

Wheeler, 1906

71FD4AF0-6D9A-55AB-A3CB-528924F0BBF7

##### Notes

Choi (1996), Won et al. (2023)

#### Temnothorax congruus

(Smith, 1874)

CE8A7BD7-9EC9-5F1F-9DB7-2E409B799B29

##### Notes

Choi (1996)

#### Temnothorax spinosior

(Forel, 1901)

23254D84-022C-5B4E-AB4C-3ED470B9897C

##### Notes

Won et al. (2023)

#### Tetramorium tsushimae

Emery, 1925

29E78ECD-26A0-5347-8544-E7FD28B5E2B1

##### Notes

Choi (1996), Lee and Jung (2001), Lim and Lee (2012), HNIBR (2022), Won et al. (2023)

#### Vollenhovia emeryi

Wheeler, 1906

D32EED7A-EB00-56D0-9A4C-5E825E60E4D9

##### Notes

Choi (1996)

#### Gasteruptiidae

A1628562-4F5B-515A-B089-B09D0C411DCB

#### Gasteruption japonicum

Cameron, 1888

5253FBE7-A97A-5113-B287-08A4BBB469CA

##### Notes

Lee and Kwon (1981), ME (1993), Kwon et al. (1996), Lee and Jung (2001)

#### Halictidae

05026018-6033-5056-ACE6-E3071A1EA285

#### Lasioglossum duplex

(Dalla Torre, 1896)

D245A4F7-3B11-53D3-968D-46C894F45CC9

##### Notes

Lee and Jung (2001)

#### Lasioglossum mutilum

(Vachal, 1903)

98A247FA-C59D-5583-B218-D327BBF97E38

##### Notes

Kim (1971), Lee and Kwon (1981), ME (1993), Kwon et al. (1996), Lee and Jung (2001)

#### Seladonia aeraria

(Smith, 1873)

A95065FA-A335-59ED-9037-B08D058A99A5

##### Notes

Lee and Kwon (1981), ME (1993), Kwon et al. (1996), Lee and Jung (2001)

#### Sphecodes simillimus

Smith, 1873

944F30F2-2990-5DF3-91CA-7033C3958141

##### Notes

Lee and Kwon (1981), ME (1993), Kwon et al. (1996), Lee and Jung (2001)

#### Ichneumonidae

393F3EDE-0C5E-51EC-9B69-4718F1F5AAD3

#### Achaius oratorius albizonellus

(Matsumura, 1912)

481F70C3-401D-5FBD-84CF-1C8FAF7007D2

##### Notes

Lee and Kwon (1981), ME (1993), Kwon et al. (1996), Lee and Jung (2001), Lee (2014)

#### Acroricnus ambulator

(Smith, 1874)

97BFD4DD-CF44-5A12-9205-CB0AE1193FCA

##### Notes

Cho (1955), Cho (1965), Lee and Kwon (1981), ME (1993), Kwon et al. (1996), Lee and Jung (2001)

#### Aphanistes jozankeanus

(Matsumura, 1912)

D9353C04-9DFE-5108-AEC7-6CA02AB77429

##### Notes

HNIBR (2022)

#### Aphanistes ruficornis

(Gravenhorst, 1829)

E58057B6-EE29-5B58-A633-867A591432BF

##### Notes

Lee and Kwon (1981), ME (1993), Kwon et al. (1996), Lee and Jung (2001)

#### Clistopyga sziladyi

Kiss, 1959

D7458BED-EBDC-560C-9E2E-F2A35D75F287

##### Notes

HNIBR (2022)

#### Coelichneumon cyaniventris

(Wesmael, 1859)

DFB7D8C4-5F34-53AC-8D28-D46F2EABC1A3

##### Notes

Won et al. (2023)

#### Cratichneumon argemus

Townes, Momoi & Townes, 1965

16CA0DBE-89DC-5E26-8C3C-91F051265F11

##### Notes

Lee and Kwon (1981), ME (1993), Kwon et al. (1996), Lee and Jung (2001)

#### Dicamptus nigropictus

(Matsumura, 1912)

6BA32795-9A6C-518D-B20F-54D92D6A129D

##### Notes

Lee and Jung (2001)

#### Dolichomitus tuberculatus tuberculatus

(Geoffroy, 1785)

416CDD8F-5C93-5FA2-9F62-F155B557051C

##### Notes

Cho (1955), Kim (1971), Lee and Kwon (1981), ME (1993), Kwon et al. (1996), HNIBR (2022)

#### Exochus semilividus

Vollenhoven, 1875

6E2A2CDE-E84B-5B29-A31A-03BEC1963F41

##### Notes

Lee et al. (2017)

#### Habronyx sonani

(Uchida, 1958)

A8468582-A494-5DBE-8670-7A4C7552D3B1

##### Notes

Lee and Jung (2001)

#### Hyposoter didymator

(Thunberg, 1822)

9DE91F54-909B-5DCD-8D7E-6243D1803CD7

##### Notes

HNIBR (2022)

#### Hyposoter takagii

(Matsumura, 1926)

4A8B82EF-9885-53D6-BD8A-3452D9D3A250

##### Notes

Kim (1971), Lee and Kwon (1981), ME (1993), Kwon et al. (1996), Lee and Jung (2001), HNIBR (2022)

#### Javra coreensis

(Uchida, 1930)

043B9271-35C8-5BE4-B62F-7B5DA3466ED5

##### Notes

Lee and Kwon (1981), ME (1993), Kwon et al. (1996), Lee and Jung (2001)

#### Mesochorus minowai

Uchida, 1929

CBD6EA3B-6080-5A4B-BF03-EC572AE59044

##### Notes

Lim and Lee (2012), HNIBR (2022)

#### Netelia cristata

(Thomson, 1888)

1F7A6001-DA4B-54EA-9974-D3B6371F245A

##### Notes

Lim and Lee (2012)

#### Netelia kiuhabona

(Uchida, 1928)

767D6C46-2D4D-5160-BB0F-3BACC8A1A698

##### Notes

Lee and Jung (2001)

#### Netelia ocellaris

(Thomson, 1888)

2A23C395-BAE7-5886-B7B9-F07073818A5D

##### Notes

Lee and Jung (2001)

#### Netelia testacea

(Gravenhorst, 1829)

A2F8CCAC-6E1C-5538-A649-3377D275556E

##### Notes

Lim and Lee (2012)

#### Ophion ainoicus

Uchida, 1928

8EA4E63E-FFFE-5D57-B53F-A2393F26A4A9

##### Notes

Lim and Lee (2012), HNIBR (2022)

#### Pimpla aethiops

Curtis, 1828

EF299AFE-292E-5497-9E6A-5AF05DA7A29F

##### Notes

Kim (1971), Lee and Kwon (1981), ME (1993), Kwon et al. (1996), Lee and Jung (2001), HNIBR (2022)

#### Pimpla luctuosa

Smith, 1874

8546CF80-8778-572B-B8C9-30883C255D25

##### Notes

Lee and Kwon (1981), ME (1993), Kwon et al. (1996), Lee and Jung (2001)

#### Pimpla nipponica

Uchida, 1928

C3F4AF41-CD80-5F88-B86A-41A5CC3B322C

##### Notes

HNIBR (2022)

#### Pimpla rufipes

Brulle, 1846

0C0C6EE8-8E11-5A92-9970-6A87B8FE6C96

##### Notes

Lee and Kwon (1981), ME (1993), Kwon et al. (1996), Lee and Jung (2001)

#### Quandrus pepsoides

(Smith, 1852)

DAB97CA8-0CA5-5177-8C7C-7337FB1420CC

##### Notes

Lee and Kwon (1981), ME (1993), Kwon et al. (1996), Lee and Jung (2001), Lee et al. (2012)

#### Zatypota albicoxa

(Walker, 1874)

0C09AFCD-09DD-5868-8996-434627B360F4

##### Notes

HNIBR (2022)

#### Megachilidae

3295E281-5ED2-53D1-B6BE-721158C5A8E8

#### Coelioxys fenestratus

Smith, 1873

5F36837C-2C4E-5058-B994-C20E1394F5AA

##### Notes

Lee and Kwon (1981), ME (1993), Kwon et al. (1996), Lee and Jung (2001)

#### Megachile lapponica

Thomson, 1872

0E551112-B582-5758-93FF-B4B9AD2BE055

##### Notes

Lee and Jung (2001)

#### Megachile nipponica

Cockerell, 1914

764096D1-AC8C-54A7-AA71-2A43E6D3C8A7

##### Notes

Lim et al. (2013)

#### Megachile sculpturalis

Smith, 1853

32A836B4-0FB1-507E-810F-1161D3CC3737

##### Notes

Lee and Kwon (1981), ME (1993), Kwon et al. (1996), Lee and Jung (2001), Lim et al. (2013), HNIBR (2022)

#### Mymaridae

C4233E51-9D73-5D2F-A00F-F6176EC6F339

#### Gonatocerus cincticipitis

Sahad, 1982

149AB17C-8710-5FEA-B1E2-A2FC56BEF5EA

##### Notes

Nam et al. (2018)

#### Himopolynema hishimonus

Taguchi, 1977

C7DD41F1-69CC-5E5E-B291-2FD67426CE18

##### Notes

Nam et al. (2018)

#### Pomphilidae

F8AC9EDF-5584-5B4B-BA7F-1C78D97099FB

#### Cyphononyx fulvognathus

(Rohwer, 1911)

0F0F3A15-B0D8-515E-B767-B7F2D38DFECF

##### Notes

Cho (1955), Cho (1965), Lee and Kwon (1981), ME (1993), Kwon et al. (1996), Lee and Jung (2001)

#### Eopompilus internalis

(Matsumura, 1911)

0B875A07-B8D9-5E89-AADE-4FECBC895765

##### Notes

Lee and Jung (2001)

#### Priocnemis irritabilis

Smith, 1873

FE4B4BBF-BE93-5504-8D47-D3A624D8E6E8

##### Notes

Kim (1971), Lee and Kwon (1981), ME (1993), Kwon et al. (1996), Lee and Jung (2001), HNIBR (2022)

#### Pteromalidae

90262D59-C938-5D06-B06C-557820D79F43

#### Nasonia vitripennis

(Walker, 1836)

DE427A46-BACD-59A8-AE9B-F4D62048850A

##### Notes

Nam et al. (2018)

#### Psilocera obscura

Walker, 1833

BC1C2492-C7B5-598F-A259-1B7B7131BEE8

##### Notes

Lee et al. (2019)

#### Pteromalide

BF9C2E44-3408-5D8C-9DF7-96A72635B2D2

#### Pteromalus puparum

(Linnaeus, 1758)

9DE721B8-F65C-5912-8382-B1E01ADD5EDB

##### Notes

Kim (1971), Lee and Kwon (1981), ME (1993), Kwon et al. (1996), Lee and Jung (2001), Nam et al. (2018)

#### Sphecidae

AA1CA4C6-31DC-5B92-B47F-5408F93B07C5

#### Ammophila infesta

Smith, 1873

8A14CF26-525C-5933-917C-B7E3A0B215EC

##### Notes

Cho (1955), Cho (1965), Lee and Kwon (1981), ME (1993), Kwon et al. (1996), Lee and Jung (2001), Lim et al. (2013)

#### Ammophila vagabunda

Smith, 1856

9C5C75EE-A731-54A5-B93F-44AA2585D0C7

##### Notes

Kim (1971)

#### Sceliphron deforme atripes

(Morawitz, 1888)

2B075218-E876-5FBB-AE71-F5972F002D8C

##### Notes

Lee and Kwon (1981), ME (1993), Kwon et al. (1996), Lee and Jung (2001)

#### Tenthredinidae

CA5B6BA7-D452-5713-9886-27BD45C3C70F

#### Athalia kashmirensis

Benson, 1932

C793F4B0-CE77-55D0-8F1B-98367830A91B

##### Notes

Lim et al. (2013)

#### Athalia proxima

(Klug, 1815)

81D7C13D-7914-519C-8629-27E6907B7B2A

##### Notes

Lee and Kwon (1981), ME (1993), Kwon et al. (1996), Lee and Jung (2001)

#### Athalia rosae ruficornis

JakovLev, 1888

5E1E0A9E-2848-526D-BE9B-BA0E7A75AF80

##### Notes

Kim (1971), Lee and Kwon (1981), ME (1993), Kwon et al. (1996), Lee and Jung (2001), Lim and Lee (2012), Lim et al. (2013), HNIBR (2022)

#### Macrophya fascipennis

Takeuchi, 1933

CA0A73B8-EA96-5C7D-B27F-D7485BDF183C

##### Notes

Lee and Kwon (1981), ME (1993), Kwon et al. (1996), Lee and Jung (2001)

#### Torymidae

8D6780DE-7040-589D-96CB-3881C52250D1

#### Podagrion nipponicum

Habu, 1962

781D218B-B5C6-5853-8F1B-52BFF0A8636C

##### Notes

Lee and Kwon (1981), ME (1993), Kwon et al. (1996), Lee and Jung (2001)

#### Vespidae

C0B3CB03-1E19-5DEF-92A6-01C661574034

#### Discoelius japonicus

Peréz, 1905

7C6ED199-A4C8-5689-B000-01ACF51F747A

##### Notes

Kim (1971), Lee and Kwon (1981), ME (1993), Kwon et al. (1996), Lee and Jung (2001), Lim et al. (2013)

#### Eumenes pomiformis

(Fabricius, 1781)

6B6E5916-6CCF-50B8-95D1-8F9E9BCA991A

##### Notes

Lee and Kwon (1981), ME (1993), Kwon et al. (1996), Lee and Jung (2001)

#### Eumenes punctatus

de Saussure, 1852

9C58F355-652B-504F-B5E0-C7371C21CA08

##### Notes

Lee and Jung (2001)

#### Eumenes rubronotatus

Pérez, 1905

D0B1AD27-F9FC-5703-8A9C-9618F70F9F16

##### Notes

Kim (1971), Lee and Kwon (1981), ME (1993), Kwon et al. (1996), Lee and Jung (2001), Lim et al. (2013), HNIBR (2022)

#### Vespa binghami

Buysson, 1905

61F39BD6-2ABF-5697-AAF3-6DB21A51086E

##### Notes

Kwon (2022)

#### Vespa crabro flavofasciata

Cameron, 1903

E26E1BB2-C0A5-5DAC-82E9-FE7A28CD3B94

##### Notes

Kwon (2022)

#### Vespa simillima simillima

Smith, 1868

800D5286-5272-5BBD-AB14-175FF7BC7430

##### Notes

Lim et al. (2013), Won et al. (2023)

#### Vespula flaviceps flaviceps

Smith, 1870

E6C88375-9335-5F76-A3D7-351CF2DCA376

##### Notes

Lee and Jung (2001), Won et al. (2023)

#### Vespula koreensis koreensis

Radoszkowski, 1887

8F6EF682-939D-50F9-94F4-5200225B4DC6

##### Notes

Lim and Lee (2012), HNIBR (2022)

#### Vespula rufa schrenckii

(Radoszkowski, 1861)

D1322D2C-5FC4-5793-BEF3-A47F6ABA93AB

##### Notes

Lim et al. (2013)

#### Lepidoptera

C3B39A24-D83E-5204-BBDE-DE6FDEB32ACB

#### Argyresthiidae

BAB70B5B-0F7F-53F8-B21F-2035F24A94D5

#### Argyresthia subrimosa

Meyrick, 1932

D36A0018-DEB1-5285-80C3-2D0583CDEEEC

##### Notes

Info and Park (2023)

#### Autostichidae

B1C176A0-E10B-59C5-844A-8B96E93BB2FD

#### Meleonoma torophanes

(Meyrick, 1935)

57B58B71-9A70-5DAB-9D88-B95EEF2CF2E5

##### Notes

Kwon (2022)

#### Bombycidae

5052F7B7-6DF3-54F4-95C4-D9E941EB7F3E

#### Bombyx mandarina

(Moore, 1872)

E5B006AD-195F-5877-9EFF-48A99D40840C

##### Notes

Lee and Jung (2001), Lim et al. (2013)

#### Rondotia menciana

Moore, 1885

124B6729-16C3-5DB6-A1C5-37460BA0ECB9

##### Notes

Kwon (2022)

#### Callidulidae

DBFC3CB0-F82C-5936-A46B-09BD4975CBF3

#### Pterodecta felderi

(Bremer, 1864)

B417FD42-2CD6-5D4A-89D8-47BABA854A92

##### Notes

Cho (1929), Cho (1955), Cho (1965), Lee and Kwon (1981), ME (1993), Byun et al. (1996), Kwon et al. (1996), Lee and Jung (2001), Lim and Lee (2012), Lim et al. (2013), HNIBR (2022), Won et al. (2023)

#### Choreutidae

5EEAD010-1215-5F8B-9E6E-5C94D5ADA369

#### Choreutis hyligenes

(Butler, 1879)

2BCEF765-7E61-5DAB-8F6C-0717DC33C0E6

##### Notes

Kwon (2022)

#### Crambidae

2E5EB60A-ABB4-599D-B2F7-A493A9E3D26C

#### Anania lancealis

(Denis & Schiffermüller, 1775)

3DF9C150-5E68-53D5-94F9-BB39196B7FCF

##### Notes

Lim et al. (2013)

#### Anania verbascalis

(Denis et Schiffermüller, 1775)

17F8A39C-1ACE-5B20-9985-A6764AEFEF00

##### Notes

Lim et al. (2013)

#### Anania vicinalis

(South, 1901)

720E40B6-2D92-58FC-BDD9-D4F59EAC898F

##### Notes

Bae (2014)

#### Ancylolomia japonica

Zeller, 1887

B0A2F7B2-F142-54C1-9DEA-D57BF9608C85

##### Notes

Lim et al. (2013)

#### Botyodes principalis

Leech, 1889

0CE05772-2DD4-5BF1-8D0D-5DC557EDAC05

##### Notes

Lim et al. (2013)

#### Bradina geminalis

Caradja, 1927

80B91C99-0A72-5AAB-8512-49E7B769700B

##### Notes

Kim (1971), Lee and Kwon (1981), ME (1993), Byun et al. (1996), Kwon et al. (1996), Lee and Jung (2001), HNIBR (2022)

#### Chrysoteuchia diplogramma

Zeller, 1863

78794874-27EB-5BA1-BDE4-DE0C55D68BC8

##### Notes

Lim et al. (2013)

#### Cnaphalocrocis medinalis

(Guenée, 1854)

EAE82BD2-4A9E-5F8E-8306-9603B856D23A

##### Notes

Kim (1971), Lee and Kwon (1981), ME (1993), Byun et al. (1996), Kwon et al. (1996), Lee and Jung (2001)

#### Conogethes punctiferalis

Guenée, 1854

0A82B8EF-2BE6-5E9E-948F-6981B1CA060F

##### Notes

Lim et al. (2013)

#### Cotachena alysoni

Whalley, 1961

838CD68E-898D-5530-98F6-C810257985CA

##### Notes

Kim (1999)

#### Crambus perlellus

Scopoli, 1763

E764C0F6-64DF-5652-AE76-A96899438022

##### Notes

Lim et al. (2013)

#### Diaphania indica

(Saunders, 1851)

D84EAE38-8BDC-5044-9F79-D4A3509B6CBE

##### Notes

Lee and Jung (2001)

#### Ecpyrrhorrhoe minnehaha

(Pryer, 1877)

D2E88623-4968-5F65-8F1D-63083993CF76

##### Notes

Lee and Kwon (1981), ME (1993), Byun et al. (1996), Kwon et al. (1996), Lee and Jung (2001), Lim et al. (2013), Won et al. (2023)

#### Eurrhyparodes accessalis

Walker, 1859

D21CBDEB-9ABB-5562-A08F-61706421F00B

##### Notes

Lim et al. (2013)

#### Glaucocharis exsectella

(Christoph, 1881)

D14DB14E-13BF-5F31-B741-1F34F8131277

##### Notes

Lim et al. (2013)

#### Glaucocharis vermeeri

(Bleszynski, 1965)

5A3F2B5B-4E6A-58A7-A6AD-3E8A2CD9227D

##### Notes

HNIBR (2022)

#### Glyphodes pryeri

Butler, 1879

DB09F0FF-1A7E-58A7-993A-E58B51691709

##### Notes

Lim and Lee (2012), Lim et al. (2013), HNIBR (2022), Won et al. (2023)

#### Glyphodes quadrimaculalis

Bremer & Grey, 1853

840F63BD-EE7B-55A5-9906-0BD4E4F2E396

##### Notes

Kim (1971), Lee and Kwon (1981), ME (1993), Byun et al. (1996), Kwon et al. (1996), Lee and Jung (2001), HNIBR (2022), Won et al. (2023)

#### Goniorhynchus clausalis

Christoph, 1881

F0A3DFDA-1A7B-5CE0-9119-69CE10B19BE4

##### Notes

Lim et al. (2013)

#### Goniorhynchus exemplaris

Hampson, 1898

8DD72F8D-2144-53E4-93AA-92824B6E1705

##### Notes

Lee and Kwon (1981), ME (1993), Byun et al. (1996), Kwon et al. (1996), Lee and Jung (2001)

#### Haritalodes derogata

(Fabricius, 1775)

C61E0F21-369E-5A3F-88C4-08A9CC083A9A

##### Notes

Kim (1971), Lee and Kwon (1981), ME (1993), Byun et al. (1996), Kwon et al. (1996), Lee and Jung (2001), Lim et al. (2013), Won et al. (2023)

#### Herpetogramma fuscescens

(Warren, 1892)

FA3B1E2E-BE43-5E67-B964-334352810D55

##### Notes

Lim et al. (2013)

#### Herpetogramma luctuosalis

(Guenée, 1854)

F2633BD9-B881-54F1-9411-60556881FB05

##### Notes

Cho (1965), Lee and Kwon (1981), ME (1993), Byun et al. (1996), Kwon et al. (1996), Lee and Jung (2001), Lim et al. (2013), HNIBR (2022), Won et al. (2023)

#### Herpetogramma magna

(Butler, 1879)

BA86CF58-532B-561D-A521-31311381A45E

##### Notes

Kim (1971), Byun et al. (1996), HNIBR (2022)

#### Herpetogramma ochrimaculalis

(South, 1901)

096F2223-159D-5A25-AB70-D6AB2C4F5834

##### Notes

HNIBR (2022)

#### Herpetogramma phaeopteralis

(Guenée, 1854)

1EE4DAFF-ADD2-5595-A7E3-2AA757226D98

##### Notes

Lim et al. (2013)

#### Herpetogramma rudis

(Warren, 1892)

35DD58AF-A0C7-5267-A060-EB98E4E0431B

##### Notes

Lim et al. (2013)

#### Maruca vitrata

(Fabricius, 1787)

7BC37BC3-A6CC-583E-82D3-6E7066754A3E

##### Notes

Lim et al. (2013)

#### Mimudea tritalis

Christoph, 1881

F13F2B44-D950-55A6-993E-F48BFBD15D50

##### Notes

Info and Park (2023)

#### Nacoleia commixta

(Butler, 1879)

C3EDD3EC-99D3-5E43-88C1-D4676A012906

##### Notes

Lim et al. (2013)

#### Nacoleia sibirialis

(Milliere, 1879)

F6F65360-15FC-552E-8718-95C1826C92E2

##### Notes

NSMK (2023a)

#### Neopediasia mixtalis

Walker, 1863

0CB64565-2EA8-54E4-8086-88249AAF4327

##### Notes

Lim et al. (2013)

#### Nomophila noctuella

(Denis & Schiffermüller, 1775)

37B6549E-034D-5E76-AE1C-B3A2754E8C45

##### Notes

Kwon (2022)

#### Omiodes diemenalis

(Guenée, 1854)

456D4464-3317-515D-87A0-548AF8CF0D3E

##### Notes

HNIBR (2022)

#### Omiodes noctescens

Moore, 1888

283E8147-6977-596A-8554-7E5D5D06A34F

##### Notes

Lee and Jung (2001), Lim et al. (2013)

#### Omiodes poeonalis

(Walker, 1859)

1701AD74-76F9-5C2E-9B40-BE8A0A06A0D0

##### Notes

Kim (1971), Lee and Kwon (1981), ME (1993), Byun et al. (1996), Kwon et al. (1996), Lee and Jung (2001), Lim and Lee (2012), HNIBR (2022)

#### Ostrinia furnacalis

(Guenée, 1854)

46198FC4-2B79-5D58-B253-F4F229D0FCFF

##### Notes

Lee and Kwon (1981), ME (1993), Byun et al. (1996), Kwon et al. (1996), Lee and Jung (2001), Lim and Lee (2012), HNIBR (2022)

#### Ostrinia palustralis

(Hübner, 1796)

9313D3E9-BFC2-50E1-8385-0C1CD5F78DFE

##### Notes

Kim (1971), Lee and Kwon (1981), ME (1993), Byun et al. (1996), Kwon et al. (1996), Lee and Jung (2001), Lim and Lee (2012), HNIBR (2022)

#### Ostrinia scapulalis

(Walker, 1859)

3AB1E3C4-C315-54DC-A1B9-D0C8B69BE0B1

##### Notes

Kim (1971)

#### Ostrinia zealis

(Guenée, 1854)

2A49F463-4A45-577C-A597-46BA42659C0A

##### Notes

Lee and Kwon (1981), ME (1993), Byun et al. (1996), Kwon et al. (1996), Lee and Jung (2001)

#### Pagyda quadrilineata

Butler, 1881

7860A9C6-67CC-5FE7-9B03-4BADC2D07D23

##### Notes

Lim et al. (2013)

#### Pagyda quinquelineata

Hering, 1903

2F6C1C71-80A0-5F58-9654-F89670A008B4

##### Notes

Kwon (2022)

#### Pagyda salvalis

Walker, 1859

90C2F7C1-AA6F-5D31-8F67-8EE2A4EE6368

##### Notes

Bae (2014)

#### Palpita nigropunctalis

Bremer, 1864

EA092698-FE0F-53FA-93CC-58C255486073

##### Notes

Kim (1971), Lee and Kwon (1981), ME (1993), Byun et al. (1996), Kwon et al. (1996), Lee and Jung (2001), Lim et al. (2013), HNIBR (2022), Won et al. (2023)

#### Patania balteata

(Fabricius, 1798)

92AE5D4E-D2BE-5BC9-99DB-FF7DFE6BDDD7

##### Notes

Kim (1971), Lee and Kwon (1981), ME (1993), Byun et al. (1996), Kwon et al. (1996), Lee and Jung (2001), Lim et al. (2013)

#### Patania chlorophanta

(Butler, 1878)

181C1C40-EC1E-52F1-B4D0-DC3B5EA484D0

##### Notes

Won et al. (2023)

#### Patania ruralis

(Scopoli, 1763)

B91BCB63-1ADD-5C47-9DA8-DF18C348163C

##### Notes

Kwon (2022)

#### Polythlipta liquidalis

Leech, 1889

B532242E-CED6-580A-B675-17EC02B2B280

##### Notes

Kwon (2022)

#### Prodasycnemis inornata

(Butler, 1879)

46AA16BA-DC3B-5AAF-888C-45693C6ECA45

##### Notes

Kim (1971), HNIBR (2022)

#### Pseudebulea fentoni

Butler, 1881

EEFDAA6B-1AAC-5916-96A3-81B488E4FF93

##### Notes

Kwon (2022)

#### Pseudocatharylla simplex

Zeller, 1877

53306E7E-A925-587A-A034-AF5F5A281599

##### Notes

Lim et al. (2013)

#### Sitochroa palealis

(Denis et Schiffermüller, 1775)

7644BAC5-4A2A-575C-9004-8EC0F65C3341

##### Notes

Kim (1971), Lee and Kwon (1981), ME (1993), Byun et al. (1996), Kwon et al. (1996), Lee and Jung (2001), HNIBR (2022)

#### Spoladea recurvalis

(Fabricius, 1775)

96CF10F5-E626-584F-B965-FA69178AD3C6

##### Notes

Cho (1965), Lee and Kwon (1981), ME (1993), Byun et al. (1996), Kwon et al. (1996), Lee and Jung (2001), Lim et al. (2013)

#### Stenia bruguieralis

(Duponchel, 1833)

341D3384-1D62-5515-BA9B-9EC99FB5EDE2

##### Notes

HNIBR (2022), NSMK (2023a)

#### Syllepte pallidinotalis

Hampson, 1912

3C33F1E6-F935-5F29-8969-9797D888786B

##### Notes

Lim et al. (2013)

#### Syllepte taiwanalis

Shibuya, 1928

DFD02CE4-B72D-527E-BFD1-D81267DE8D72

##### Notes

Byun et al. (1996), Lee and Jung (2001), Lim et al. (2013)

#### Tabidia strigiferalis

Hampson, 1900

1C96B83D-5018-5E11-AA99-5259F1C1EE37

##### Notes

Lim et al. (2013)

#### Trichophysetis cretacea

Butler, 1879

0720A495-AB99-56F6-8086-F0B728B89A13

##### Notes

Byun et al. (1996), Lim et al. (2013)

#### Udea exigualis

(Wileman, 1911)

A588F3EB-306E-55B5-B23A-7053D019E926

##### Notes

Kwon (2022)

#### Depressariidae

907E2ED6-E5B1-57E8-A6F4-000D9A929F2A

#### Acria ceramitis

Meyrick, 1908

BDB6B273-CBED-5F69-B4E0-57028C4EE8CD

##### Notes

Lim et al. (2013)

#### Drepanidae

F4D79695-EED2-5070-B132-39DDE1994DE6

#### Auzata superba

(Butler, 1878)

8DCEBB0C-79A0-5ACC-815D-9D5608419D76

##### Notes

Lim et al. (2013)

#### Callidrepana patrana palleolus

(Motschulsky, 1866)

D2304515-1BFC-5481-A098-F05DC5FE32DA

##### Notes

Lim et al. (2013)

#### Cyclidia substigmaria nigralbata

Warren, 1914

4F818881-EDEE-5E42-9AAF-1C2169BD2647

##### Notes

Lim and Lee (2012)

#### Ditrigona conflexaria microniodes

(Strand, 1916)

3C1DBD5F-6A59-52E5-957A-DD15E63DFFC0

##### Notes

Kim (1971), Lee and Kwon (1981), ME (1993), Kwon et al. (1996), Lee and Jung (2001), Lim and Lee (2012), HNIBR (2022)

#### Ditrigona komarovi

(Kurentzov, 1935)

139AF415-095B-5D08-ABA0-BC4212ABFD4D

##### Notes

Kwon (2022)

#### Nordstromia grisearia

(Staüdinger, 1892)

D43F027E-2679-56B2-AD25-83FD89957FB0

##### Notes

Lim et al. (2013)

#### Nordstromia japonica

(Moore, 1877)

EC55B69F-6ED4-5682-9158-A5415E10C166

##### Notes

Cho (1965), Lee and Kwon (1981), ME (1993), Kwon et al. (1996), Lee and Jung (2001), Lim and Lee (2012), Lim et al. (2013), HNIBR (2022), Won et al. (2023)

#### Tethea albicostata

(Bremer, 1861)

0907DFBC-C24C-5EA3-B5C2-8F97E1E43B77

##### Notes

Lim et al. (2013)

#### Tethea consimilis consimilis

(Warren, 1912)

66B01F9B-7B59-5863-9A6C-09828E840DBD

##### Notes

NSMK (2023a)

#### Thyatira batis batis

(Linnaeus, 1758)

1B0CB288-53E0-538A-A044-7D434EA1711C

##### Notes

Byun et al. (1996), Lee and Jung (2001), Lim and Lee (2012), Lim et al. (2013), HNIBR (2022), Won et al. (2023)

#### Erebidae

1C3DFAEB-BD17-54E9-817B-EECBB4F5762B

#### Amata fortunei

(Orza, 1869)

8A72B10E-5C08-5CBB-8C7C-3AFF8166F58B

##### Notes

Kwon et al. (1996), Lee and Jung (2001)

#### Amata germana

Felder, 1862

B5D755D2-AF9D-583B-BCE9-96B28F0EB02A

##### Notes

Kwon et al. (1996), Lee and Jung (2001)

#### Artaxa subflava

(Bremer, 1864)

09F6EA86-F1AD-5F1C-BB1D-41F146546EE2

##### Notes

Cho (1955), Cho (1965), Lee and Kwon (1981), ME (1993), Byun et al. (1996), Kwon et al. (1996), Lee and Jung (2001), HNIBR (2022)

#### Artena dotata

(Fabricius, 1794)

D4D4D523-25D2-5D75-9447-E309799EC7F0

##### Notes

Lim et al. (2013)

#### Aventiola pusilla

(Butler, 1879)

AAE9643E-1538-5E4C-A5D7-A40AAE20289C

##### Notes

Kim (1971), Lee and Kwon (1981), ME (1993), Kwon et al. (1996), Lee and Jung (2001), Lim et al. (2013)

#### Barsine striata

(Bremer & Grey, 1852)

B1EE3457-C359-5B5B-9391-C149A3D9BB97

##### Notes

Kim (1971), Lee and Kwon (1981), ME (1993), Kwon et al. (1996), Lee and Jung (2001), Lim and Lee (2012), Lim et al. (2013), HNIBR (2022), Won et al. (2023)

#### Blasticorhinus ussuriensis

(Bremer, 1861)

0952DA43-87F6-557C-A560-A56B0F61C09E

##### Notes

Kim (1971), Lee and Kwon (1981), ME (1993), Kwon et al. (1996), Lee and Jung (2001)

#### Calyptra thalictri

(Borkhausen, 1790)

EF6F0DB0-A14C-5967-87EE-7059043E804F

##### Notes

Lee and Kwon (1981), ME (1993), Kwon et al. (1996), Lee and Jung (2001), Lim et al. (2013)

#### Catocala dula

Bremer, 1861

F038CE03-C9F5-56FE-93D7-EEBBA17BCC39

##### Notes

Lee and Jung (2001)

#### Catocala jonasii

Butler, 1877

2025495F-C26D-5861-A946-7E6D095A68E2

##### Notes

Lim et al. (2013)

#### Catocala lara

Bremer, 1861

F4A2AD88-E7E1-5497-AA8F-BC3798A248F7

##### Notes

Won et al. (2023)

#### Catocala nagioides

Wileman, 1924

0782F178-AECE-510E-BACE-E169BE52CCB2

##### Notes

Lee and Jung (2001)

#### Catocala nubila

Butler, 1881

071A10C6-C272-568E-880C-B595EEFA9223

##### Notes

Cho (1965), Lee and Kwon (1981), ME (1993), Kwon et al. (1996), Lee and Jung (2001), Won et al. (2023)

#### Chionarctia nivea

(Ménétriés, 1859)

BD7EC08D-DE8B-55B9-B46D-06F47226D019

##### Notes

Cho (1955), Cho (1965), Lee and Kwon (1981), ME (1993), Kwon et al. (1996), Lee and Jung (2001), Won et al. (2023)

#### Cifuna locuples locuples

Walker, 1855

0DC97734-875F-5CF8-A193-366D28FACE7F

##### Notes

Lim et al. (2013)

#### Collita griseola

(Hübner, [1803])

859933FF-14F2-5103-8B1B-4619E771968D

##### Notes

Kim (1971), Lee and Kwon (1981), ME (1993), Byun et al. (1996), Kwon et al. (1996), Lee and Jung (2001), Lim et al. (2013), HNIBR (2022)

#### Corgatha gifuensis

Nagano, 1918

5E19FDA4-0F86-50B0-B538-FEFE8262C545

##### Notes

Lim et al. (2013)

#### Cyana hamata

(Walker, 1854)

098DFDC2-457C-5387-98A2-176CFF6E6E59

##### Notes

Lee and Jung (2001), Lim et al. (2013)

#### Diomea discisigna

Sugi, 1963

8D2DC2D7-198E-538F-8A72-4F785E50C680

##### Notes

Lim et al. (2013)

#### Edessena hamada

(Felder & Rogenhofer, 1874)

D60EDC79-39F7-5491-B4BB-46D61A354E3C

##### Notes

Lim et al. (2013)

#### Ercheia umbrosa

Butler, 1881

1850D3CD-570D-566F-9CC7-B32E96C3F2A9

##### Notes

Lee and Jung (2001)

#### Euwilemania angulata

(Wileman, 1911)

99F8124D-2295-5E79-A4C1-6C216CF342A8

##### Notes

Kwon (2022)

#### Ghoria gigantea

(Oberthür, 1879)

3CF5ADDF-EC8E-57E0-BF09-7EA022385C04

##### Notes

Lim et al. (2013)

#### Gonitis mesogona

(Walker, 1858)

3603E122-D02E-5324-82FE-EC4F99D7D719

##### Notes

Kim (1971), Lee and Kwon (1981), ME (1993), Kwon et al. (1996), Lee and Jung (2001), Lim et al. (2013)

#### Hadennia incongruens

(Butler, 1879)

D01BE583-DA3D-52A7-A9BA-6FCA40C093AF

##### Notes

Lim et al. (2013)

#### Herminia arenosa

Butler, 1878

9E4EF959-CA76-503D-A248-73D7730055CC

##### Notes

Kwon (2022)

#### Herminia grisealis

(Denis & Schiffermüller, 1775)

6688EE40-52AB-5F74-8216-DE9BDDD8AB17

##### Notes

Byun et al. (1996), Kwon (2022)

#### Herminia innocens

Butler, 1879

151DB358-6877-5FE3-9E31-EFD03A658901

##### Notes

Kwon (2022)

#### Herminia tarsicrinalis

(Knoch, 1782)

8EF329B7-D0DB-526E-9576-7E029789A45A

##### Notes

Kwon (2022)

#### Hydrillodes morosa

(Butler, 1879)

EC3F3665-89F7-5482-8E7D-A9A6FCE03F9A

##### Notes

Byun et al. (1996), Lim et al. (2013), Nam et al. (2018), HNIBR (2022)

#### Hypena amica

(Butler, 1878)

65F81923-7A2C-5F27-8D96-9C1DF7038F3A

##### Notes

Kim (1971), Lee and Kwon (1981), ME (1993), Kwon et al. (1996), Lee and Jung (2001), Lim et al. (2013), HNIBR (2022), Won et al. (2023)

#### Hypena proboscidalis

(Linnaeus, 1758)

F1448084-6340-5CFB-8339-0256C8DA7FCB

##### Notes

Lim et al. (2013)

#### Hypena trigonalis

(Guenée, 1854)

29E6F266-35B6-5078-8954-4B20C54D50B7

##### Notes

Lim and Lee (2012), HNIBR (2022)

#### Hypersypnoides astrigera

(Butler, 1885)

39FFCBBB-F127-5E25-8CA9-E9030D6FE92D

##### Notes

Lim and Lee (2012)

#### Hypocala subsatura

Guenée, 1852

E03F9EC7-4201-55B7-9E85-81ED718088B7

##### Notes

Won et al. (2023)

#### Hypopyra vespertilio

(Fabricius, 1787)

375B20CE-C876-55AB-818B-27CF2653405D

##### Materials

- **Type status:** Other material. **Occurrence:** recordedBy: Lee, D. Y.; sex: 1 male; lifeStage: adult; occurrenceID: DAF39A97-3029-5E47-81AC-E5EFED5FB97B; **Location:** island: Ulleungdo Island; country: Republic of Korea; stateProvince: Gyeongsangbuk-do; county: Ulleung-gun; locality: Taeharyeong, Namseo-ri, Seomyeon; **Event:** samplingProtocol: Light trap; eventDate: 8/17/2021

##### Notes

This is the first record of this species from Ulleungdo Island.

Distribution: Bangladesh, China, India, Indonesia, Japan, Korea, Singapore, Sri Lanka, Taiwan, Thailand (GBIF.org 2023b)

#### Ivela auripes

(Butler, 1877)

86FB88B3-9380-532A-81D2-5C31C26FF838

##### Notes

Info (2023)

#### Katha deplana

(Esper, 1787)

CE843136-1819-5149-998D-BC583742ADDC

##### Notes

Kim (1971), Lee and Kwon (1981), ME (1993), Kwon et al. (1996), Lee and Jung (2001), Lim et al. (2013), HNIBR (2022)

#### Lemyra boghaika

Tshistjakov & Kishida, 1994

2F911F74-4D13-515C-BDBB-2CC586862E8B

##### Notes

Bae (2013)

#### Lemyra inaequalis

(Butler, 1879)

BE5D8125-4E52-5E03-B0F1-22CFF0A294BA

##### Notes

Lee and Kwon (1981), ME (1993), Kwon et al. (1996), Lee and Jung (2001), Lim et al. (2013)

#### Lymantria dispar

(Linnaeus, 1758)

158F8576-A431-5897-AD05-71F957961B08

##### Notes

Cho (1929), Cho (1955), Cho (1965), Lee and Kwon (1981), ME (1993), Byun et al. (1996), Kwon et al. (1996), Lee and Jung (2001), HNIBR (2022)

#### Lymantria monacha

(Linnaeus, 1758)

9B64CC28-F70A-592A-A113-955BA17CE46A

##### Notes

Kwon (2022)

#### Manulea degenerella

(Walker, 1863)

6AAF033D-7F1E-5E88-A17A-42E1FE98F96B

##### Notes

NSMK (2023b)

#### Manulea japonica

(Leech, [1889])

543AE65E-3017-54F0-A778-FD27DEA36C16

##### Notes

Won et al. (2023)

#### Miltochrista miniata

(Forster, 1771)

85636D0D-EECA-50F5-8C88-F256E5E15AF3

##### Notes

Won et al. (2023)

#### Miltochrista ziczac

(Walker, 1856)

B5EC5C38-23B2-5003-8EB6-6E769E040DAC

##### Notes

Bae (2013), Lim et al. (2013)

#### Mocis ancilla

(Warren, 1913)

199427FD-31A1-52A0-BB5B-C8381E1AE8A6

##### Notes

Kim (1971), Lee and Kwon (1981), ME (1993), Kwon et al. (1996), Lee and Jung (2001)

#### Mocis annetta

(Butler, 1878)

F3D0A5F6-B827-5BC0-A01F-75E47DC6C252

##### Notes

Lee and Jung (2001), Lim and Lee (2012)

#### Nudina artaxidia

(Butler, 1881)

DB650B97-8339-54E9-B4F6-8945A6C99B51

##### Notes

Bae (2013), Lim et al. (2013)

#### Nyctemera adversata

(Schaller, 1788)

E80C22F2-6DE0-5B09-BC82-32725B239E4D

##### Notes

Nam et al. (2018)

#### Ophiusa tirhaca

(Cramer, 1777)

DB6708F4-AA03-5ADB-99DD-1A7B44D8A64F

##### Notes

Lim et al. (2013)

#### Pangrapta curtalis

(Walker, 1865)

2009ECD4-FCF4-5D2C-89A3-094694FA9676

##### Notes

Kwon (2022)

#### Paragona inchoata

(Wileman, 1911)

5C067C72-456C-5498-8BA9-F232295B9875

##### Notes

Kwon (2022)

#### Pelosia noctis

(Butler, 1881)

6797C160-5D8C-5206-ADB9-ADB1B3E41C35

##### Notes

Info (2023)

#### Perinaenia accipiter

(Felder & Rogenhofer, 1874)

6E75F668-B1BD-515E-8E66-54F75A52A6E3

##### Notes

Lim et al. (2013)

#### Rivula sericealis

(Scopoli, 1763)

4C461D3C-7AAF-55BD-9D53-8581BB25E243

##### Notes

Lim et al. (2013)

#### Rusicada leucolopha

(Prout, 1928)

B419D0A4-F7DD-535B-90DC-A6C8ACECBA97

##### Notes

Lim et al. (2013)

#### Simplicia rectalis

(Eversmann, 1842)

DFCC61B0-C523-5FC5-BD19-D81B45541924

##### Materials

- **Type status:** Other material. **Occurrence:** recordedBy: Lee, D. Y.; sex: 1 male; lifeStage: adult; occurrenceID: 1B8A2C65-613A-5F2B-8CD2-183D83EB01D5; **Location:** island: Ulleungdo Island; country: Republic of Korea; stateProvince: Gyeongsangbuk-do; county: Ulleung-gun; locality: Taeharyeong, Namseo-ri, Seomyeon; **Event:** samplingProtocol: Light trap; eventDate: 6/20/2021

##### Notes

This is the first record of this species from Ulleungdo Island.

Distribution: Palaearctic (GBIF.org 2023c)

#### Spilarctia alba

(Bremer & Grey, 1853)

1CD0B717-4574-5DDB-9F1A-1F239A0A1974

##### Notes

Lee and Kwon (1981), ME (1993), Kwon et al. (1996), Lee and Jung (2001), Lim et al. (2013)

#### Spilarctia lutea

(Hüfnagel, 1766)

A8E490BB-6994-5AB2-AA5A-F378C9AD8C3E

##### Notes

Lim and Lee (2012), Lim et al. (2013), HNIBR (2022)

#### Spilarctia seriatopunctata suzukii

(Motschulsky, 1860)

73D2C578-A738-588B-BC74-AF0DCE5848D1

##### Notes

Kim (1971), Lee and Kwon (1981), ME (1993), Kwon et al. (1996), Lee and Jung (2001), Lim and Lee (2012), Lim et al. (2013), HNIBR (2022), Won et al. (2023)

#### Spilarctia seriatopunctata suzukii

Inoue & Maenami, 1963

F3081D54-BE1C-5C9C-ABDB-BA1675D5955C

##### Materials

- **Type status:** Other material. **Occurrence:** recordedBy: Lee, D. Y.; sex: 1 male; lifeStage: adult; occurrenceID: 3C6848D2-997E-5667-994E-1DD90ED4B5D8; **Location:** island: Ulleungdo Island; country: Republic of Korea; stateProvince: Gyeongsangbuk-do; county: Ulleung-gun; locality: Taeharyeong, Namseo-ri, Seomyeon; **Event:** samplingProtocol: Light trap; eventDate: 8/16/2021

##### Notes

This is the first record of this species from Ulleungdo Island.

Distribution: Japan (Inoue and Maenami 1963), Korea (National Institute of Biological Resources 2019)

#### Spilosoma lubricipeda

(Linnaeus, 1758)

924CFBBC-91A0-5A3F-BCFB-065B648404AA

##### Notes

Byun et al. (1996), Lee and Jung (2001), Lim et al. (2013)

#### Spilosoma punctaria

(Stoll, 1782)

0D17414A-CD1A-5508-90FD-79EC42829CF2

##### Notes

Lim and Lee (2012), HNIBR (2022)

#### Spirama helicina

(Hübner, 1831)

F5E088C5-E2CB-5D8A-8E35-922EACEB070E

##### Notes

Byun et al. (1996), Lim et al. (2013)

#### Telochurus recens

(Hübner, 1819)

C3C4471E-22B4-5DBD-909B-F43EE5F134D2

##### Notes

Kwon (2022)

#### Thyas juno

(Dalman, 1823)

4542F2D8-CA93-5003-8AEB-1AE26E798C23

##### Notes

Won et al. (2023)

#### Zanclognatha lunalis

(Scopoli, 1763)

E8CE96D3-9EA4-513B-9861-958992DE5D68

##### Notes

Kwon (2022)

#### Zanclognatha tarsipennalis

(Treitschke, 1835)

5A231969-88E9-5376-8692-5860B01FE684

##### Notes

Kwon (2022)

#### Gelechiidae

16425EFB-210F-5D4A-8B62-85EF30EC39F4

#### Agnippe syrictis

(Meyrick, 1936)

2A519AE7-62FE-5455-9646-873A94456A43

##### Notes

HNIBR (2022)

#### Altenia inscriptella

(Christoph, 1882)

A78D45CF-1CDB-534B-8B9F-ACA4F7AD100A

##### Notes

Park et al. (2014), HNIBR (2022)

#### Anarsia bipinnata

(Meyrick, 1932)

F6172552-6C3C-522A-AC52-6FDD007F2E74

##### Notes

Lim et al. (2013)

#### Anarsia ulneongensis

Park & Ponomarenko, 1996

E1DCB384-8E1A-5627-A6CE-CCCEAEC55863

##### Notes

Park (2013), Park et al. (2014)

#### Angustialata gemmellaformis

Omelko, 1988

A15F0527-96E5-5FA6-A56A-882E67332EF4

##### Notes

Park et al. (2014), HNIBR (2022)

#### Argolamprotes micella

(Denis & Schiffermüller, 1775)

3B0FC6DE-AD8C-515A-AC9C-0D6C6A4093DD

##### Notes

HNIBR (2022)

#### Bagdadia gnomia

(Ponomarenko, 1996)

1A240ED0-D802-5E60-991A-80F90DA36C2B

##### Notes

Park et al. (2014), HNIBR (2022)

#### Dichomeris anisacuminata

Li & Zheng, 1996

C7795A55-9BAB-5AF4-8473-CF35720697AE

##### Notes

Park et al. (2014), HNIBR (2022)

#### Dichomeris chinganella

(Christoph, 1882)

F848C885-04C7-5AB3-8766-97E5FF898D3F

##### Notes

Lim et al. (2013), HNIBR (2022)

#### Dichomeris rasilella

(Herrich-Schäffer, 1854)

F400643B-F9CE-5343-B34C-6CBD142D5C4C

##### Notes

Park et al. (2014), HNIBR (2022)

#### Faristenia geminisignella

Ponomarenko, 1991

E4A5A32B-2FDA-58B4-B23F-B062AA80D6A6

##### Notes

Nam et al. (2018)

#### Mesophleps albilinella

(Park, 1990)

D547540B-E316-5A76-A346-BFB9CDFA4142

##### Notes

Lim et al. (2013), HNIBR (2022)

#### Polyhymno pontifera

(Meyrick, 1932)

9472E853-79C6-5806-84E1-EC3510C9FEAD

##### Notes

Lim et al. (2013)

#### Thiotricha pontifera

(Meyrick, 1932)

7A02CAC5-7BA1-5F4C-8CA7-8CCA77C5C59A

##### Notes

Park et al. (2014), HNIBR (2022)

#### Geometridae

DFE914D6-B56C-514D-AC8B-80FE37EC51E6

#### Abraxas fulvobasalis

Warren, 1894

3EEC46C9-9146-52C2-91B9-3927587BF0B9

##### Notes

Lim and Lee (2012), Lim et al. (2013), HNIBR (2022), Won et al. (2023)

#### Abraxas miranda

Butler, 1878

96824552-1880-5E3A-8C45-D3070B2888C0

##### Notes

Kim (1971), Lee and Kwon (1981), ME (1993), Byun et al. (1996), Kwon et al. (1996), Lee and Jung (2001)

#### Abraxas niphonibia

Wehrli, 1935

81CDA70A-29D0-554A-A8CA-CE364D962E67

##### Notes

Kwon (2022)

#### Aethalura ignobilis

(Butler, 1878)

F4568835-8AEA-5F7A-B48D-54419AE681CC

##### Notes

Kwon (2022)

#### Alcis angulifera

(Butler, 1878)

6858C344-A224-52DE-BED2-5E1BC0C1D780

##### Notes

HNIBR (2022), Kwon (2022)

#### Ascotis selenaria

(Denis & Schiffermüller, 1775)

7B8F0010-6FB4-5A06-AB17-E59921913DA5

##### Notes

Kim (1971), Lee and Kwon (1981), ME (1993), Byun et al. (1996), Kwon et al. (1996), Lee and Jung (2001), Lim et al. (2013), HNIBR (2022)

#### Astygisa morosa

(Butler, 1881)

84FD6742-A4F2-5478-B565-D52625C5E129

##### Notes

HNIBR (2022), Kwon (2022)

#### Biston robustum

Butler, 1879

AA084DB1-BA51-59A7-85D4-75D5CEAD477E

##### Notes

Won et al. (2023)

#### Brabira artemidora

(Oberthür, 1884)

6CB95462-5BAF-5AA8-BEE1-7DD9B580ADFA

##### Notes

HNIBR (2022), Kwon (2022)

#### Cabera griseolimbata

(Oberthür, 1879)

46634F8A-37D7-5BCA-854E-AB673B286C73

##### Notes

Kim (1971), Lee and Kwon (1981), ME (1993), Kwon et al. (1996), Lee and Jung (2001), Lim et al. (2013), HNIBR (2022), Won et al. (2023)

#### Chiasmia defixaria

(Walker, 1861)

35365CF4-63E5-52DF-9906-52AA8F07ADD7

##### Notes

Kim (1971), Lee and Kwon (1981), ME (1993), Kwon et al. (1996), Lee and Jung (2001), HNIBR (2022)

#### Chlorissa anadema

(Prout, 1930)

71C9B76A-2D78-54FF-A0EE-E4EF74EB17E9

##### Notes

Lim and Lee (2012), HNIBR (2022)

#### Chlorissa obliterata

(Walker, 1863)

5C17A0E8-DF5B-5667-B640-602982727F1A

##### Notes

Info (2023)

#### Cleora leucophaea

(Butler, 1878)

88AC1E07-31DA-5501-99BB-076E83B60F46

##### Notes

Kwon (2022)

#### Cleora venustaria

(Leech, 1891)

8605D17E-78C5-59F3-B7F4-42BA0DA59846

##### Notes

Nam et al. (2018), Kwon (2022)

#### Comostola subtiliaria

(Bremer, 1864)

AAA56480-0F07-573D-B1B0-96F8583E1B56

##### Notes

Kwon (2022)

#### Corymica pryeri

(Butler, 1878)

BD46F06A-80F5-55A1-9E48-70803DA32D77

##### Notes

Lim et al. (2013)

#### Deileptenia ribeata

(Clerck, 1759)

DF4FA105-9A81-5A7D-AAB4-23C1D6182105

##### Notes

HNIBR (2022), Won et al. (2023)

#### Descoreba simplex

Butler, 1878

60320FF1-E38C-5275-B2AC-56841F425029

##### Notes

HNIBR (2022), Kwon (2022)

#### Dysstroma cinereata japonica

(Heydemann, 1929)

9BBDE82B-CD4C-59F6-95CE-6DEF651A20EF

##### Notes

HNIBR (2022), Kwon (2022), Won et al. (2023)

#### Ecliptopera umbrosaria

(Motschulsky, 1861)

0AD339A7-30B9-5E4C-AA66-27870F7BDBAD

##### Notes

Kim (1971), Lee and Kwon (1981), ME (1993), Byun et al. (1996), Kwon et al. (1996), Lee and Jung (2001), Lim and Lee (2012), Lim et al. (2013), HNIBR (2022)

#### Ecpetelia albifrontaria

(Leech, 1891)

0B6E009A-934E-5458-AFFC-A2CC5A067E11

##### Notes

Byun et al. (1996), NSMK (2023a)

#### Ectropis aigneri

Prout, 1930

602E6956-9106-56BA-BB82-C9F7206F03B5

##### Notes

Info (2023)

#### Ectropis crepuscularia

(Denis & Schiffermüller, 1775)

B0454EA1-203B-5FAA-9E10-7D1582D712A8

##### Notes

Kwon (2021)

#### Ectropis excellens

(Butler, 1884)

6F875144-42B5-54A1-98E3-677E4475F944

##### Notes

HNIBR (2022), Kwon (2022)

#### Ectropis obliqua

(Prout, 1915)

DD988691-F725-509C-839A-D9790870E4DF

##### Notes

Lim and Lee (2012), HNIBR (2022)

#### Electrophaes corylata

(Thunberg, 1792)

8BB4E269-ADAC-517A-A800-43EDA2FA2357

##### Notes

Lim et al. (2013)

#### Epirrhoe supergressa

(Butler, 1878)

430E60DC-5092-5630-8D47-FB9924BAEBAE

##### Notes

Byun et al. (1996), HNIBR (2022), Won et al. (2023)

#### Eulithis ledereri

(Bremer, 1864)

4478F96F-3641-5412-89AB-3BAE364467B4

##### Notes

Lim and Lee (2012), Lim et al. (2013)

#### Euphyia cineraria

(Butler, 1878)

40C1B42B-AB35-5C6F-9F96-DFEE2AF6A879

##### Notes

Lim et al. (2013)

#### Eupithecia clavifera

Inoue, 1955

B4A0856C-EA9C-5081-A026-C2C565B03286

##### Notes

Kwon (2022)

#### Eupithecia kobayashii

Inoue, 1958

DFFEB03B-3F96-5362-80AC-A592D074C00C

##### Notes

Kwon (2022)

#### Eupithecia spadix

Inoue, 1955

ECD41B0B-4CAF-5AC0-8210-BFF282BFD93F

##### Notes

Choi (2014)

#### Eupithecia subtacincta

Hampson, 1895

47EB125A-65E4-50A1-99A8-856F33E9A985

##### Notes

Kwon (2022)

#### Eustroma melancholica

(Butler, 1878)

366E621C-8F88-52D3-A9BA-1D2690487660

##### Notes

HNIBR (2022)

#### Gandaritis agnes

(Butler, 1878)

1C660E6C-0884-541E-8481-5CDFA348D999

##### Notes

Lee and Jung (2001)

#### Gandaritis fixseni

(Bremer, 1864)

1F211C71-9251-5270-B0E0-A3B7E973F5AD

##### Notes

Kwon et al. (1996), Lee and Jung (2001), Lim et al. (2013), Won et al. (2023)

#### Gandaritis whitelyi

(Butler, 1878)

15A0FFE2-48EA-5E73-9F51-395AB6315389

##### Notes

Lim et al. (2013)

#### Geometra glaucaria

Ménétriès, 1859

538DD84E-EBA5-5660-AFEB-8FBBD5D2971F

##### Notes

Kwon (2022)

#### Gymnoscelis esakii

Inoue, 1955

830A4D93-C98B-5115-BB53-EA239D45514C

##### Notes

Kwon (2022)

#### Hemistola veneta

(Butler, 1879)

5F07B0AF-1FF9-531C-8201-EAA98BF118E3

##### Notes

Lim et al. (2013)

#### Heterothera postalbida

(Wileman, 1911)

87620559-8A1E-564A-84FC-072099F88444

##### Notes

Info (2023)

#### Hypomecis punctinalis

(Scopoli, 1763)

5E32DC02-4914-5972-B95A-4291134D966B

##### Notes

Kim (1971), Lee and Kwon (1981), ME (1993), Kwon et al. (1996), Lee and Jung (2001), Lim and Lee (2012), Lim et al. (2013), HNIBR (2022)

#### Idaea auricruda

(Butler, 1879)

2B6D1DDB-4616-535D-A4DF-CDDA782E7289

##### Notes

NSMK (2023a)

#### Idaea biselata

(Hüfnagel, 1767)

D187F661-A96E-5EE1-BFB1-F19DB22CD7A0

##### Notes

Kwon (2022)

#### Idaea imbecilla

(Inoue, 1955)

BB2DD344-A435-5A25-AB54-02BE0F0E48D4

##### Notes

HNIBR (2022)

#### Idiochlora ussuriaria

(Bremer, 1864)

DC52B99D-FDE4-5E44-BDA6-F275FB3C3088

##### Notes

Lim et al. (2013)

#### Jankowskia fuscaria

(Leech, 1891)

CCAC3025-B6FA-5527-83AF-F0EC389D70A5

##### Notes

Kwon (2022)

#### Jodis lactearia

(Linnaeus, 1758)

691EB640-8807-5A2C-B017-DFB4E85D7081

##### Notes

Kwon (2022)

#### Lassaba nikkonis

(Butler, 1881)

ABD621C3-6A28-5EBC-9095-C672CE0ED81D

##### Notes

Kwon (2022)

#### Lobogonodes erectaria

(Leech, 1897)

692F351B-CCD4-5923-BE32-B0D2C47CEC9C

##### Notes

Choi (2012), Lim and Lee (2012), Lim et al. (2013), HNIBR (2022), Won et al. (2023)

#### Lomographa bimaculata

(Fabricius, 1775)

497ECCDA-6372-584A-9ADB-FC75472E7D7B

##### Notes

Byun et al. (1996), Lim and Lee (2012), Lim et al. (2013), HNIBR (2022), Won et al. (2023)

#### Lomographa subspersata

(Wehrli, 1939)

4B39DB1D-8457-5694-AB39-AD5411A05E31

##### Notes

HNIBR (2022), Info (2023)

#### Lomographa temerata

(Denis & Schiffermüller, 1775)

8CA524E6-BE22-5964-9798-48D5011ACC89

##### Notes

Lim et al. (2013), Won et al. (2023)

#### Maxates ambigua

(Butler, 1878)

1C479E61-2551-53F9-9BA3-E03063FA5933

##### Notes

Lim et al. (2013)

#### Menophra senilis

(Butler, 1878)

B868A9B3-9BE0-5CAC-93C0-C7D6BA473790

##### Notes

Lee and Jung (2001), Lim and Lee (2012), Lim et al. (2013), HNIBR (2022)

#### Ninodes splendens

(Butler, 1878)

AA48A243-4186-54FE-B180-9F71470FFCA3

##### Notes

Info (2023)

#### Odontopera arida

(Butler, 1878)

13EBFAB8-8B51-5C06-869A-6794F1785226

##### Notes

Lee and Kwon (1981), ME (1993), Kwon et al. (1996), Lee and Jung (2001), Lim and Lee (2012), Lim et al. (2013), HNIBR (2022), Won et al. (2023)

#### Ophthalmitis irrorataria

(Bremer & Grey, 1853)

65A9D295-C7D3-5091-9D4D-A5658D75BAFB

##### Notes

Lim and Lee (2012), HNIBR (2022)

#### Orthocabera sericea

Butler, 1879

1B891A8C-D44B-5368-AFDF-AEC71E8DAC0E

##### Notes

Byun et al. (1996), Lim and Lee (2012), HNIBR (2022)

#### Orthocabera tinagmaria

(Guenée, 1858)

32695BB8-51DD-5A83-8D7F-173990485F92

##### Notes

Lim et al. (2013), Won et al. (2023)

#### Ourapteryx koreana

Inoue, 1993

D4EF8033-7D54-5D20-9829-25EE6B304018

##### Notes

Kwon (2021), Won et al. (2023)

#### Ourapteryx maculicaudaria

(Motschulsky, 1866)

FF9395A3-42DA-5B89-90E4-9DDDC61B309D

##### Notes

Lim et al. (2013), Kwon (2022)

#### Ourapteryx nivea

(Butler, 1883)

9B538DC1-067B-5466-87CC-7021A0DCAE0E

##### Notes

Lee and Jung (2001), Lim et al. (2013)

#### Oxymacaria temeraria

(Swinhoe, 1891)

691EFED0-3109-520C-B04B-EB57A9180A4A

##### Notes

Lim et al. (2013)

#### Pachyligia dolosa

Butler, 1878

0B3C38D7-EE70-52A7-9039-4A3A14CD8F84

##### Notes

Won et al. (2023)

#### Parabapta clarissa

(Butler, 1878)

55DB2312-80EA-57F5-96B0-ABA716EF85AC

##### Notes

Lim and Lee (2012), HNIBR (2022)

#### Paradarisa consonaria

(Hübner, 1799)

07F2B2D4-26B5-5AE4-919C-8DBC0DF66F39

##### Notes

Lim and Lee (2012), HNIBR (2022)

#### Parectropis nigrosparsa

(Wileman & South, 1917)

E9E3FCBC-FE8C-50D3-B14D-95C7DBDDFC34

##### Notes

Lim et al. (2013)

#### Pasiphila rectangulata

(Linnaeus, 1758)

D8344413-FD2E-506F-8DDA-8E5FDC1DE568

##### Notes

Kwon (2022)

#### Peratophyga hyalinata

(Kollar, 1844)

9F5DE219-58DF-5AC8-BD4F-3758A6A3ECFA

##### Notes

Kim (1971), Lee and Kwon (1981), ME (1993), Byun et al. (1996), Kwon et al. (1996), Lee and Jung (2001), HNIBR (2022)

#### Petelia rivulosa

(Butler, 1881)

ACCE4683-47EF-59E0-9E1F-BF65B1094BCB

##### Notes

Lim et al. (2013)

#### Phthonandria atrilineata

(Butler, 1881)

D306AA45-67AA-502A-BF00-C5B68D5E8E10

##### Notes

Kwon (2022)

#### Phthonosema tendinosaria

(Bremer, 1864)

BD62A96F-8CB5-5A2F-A391-9FE2B9AA0ED1

##### Notes

Lee and Jung (2001), Lim et al. (2013), Won et al. (2023)

#### Problepsis discophora

Fixsen, 1887

9A2DDE63-7B61-585B-89F6-F47C198DE36A

##### Notes

Won et al. (2023)

#### Problepsis eucircota

Prout, 1913

F77C6206-3E55-5BA3-9450-7BA59A5BE97F

##### Notes

Lim et al. (2013)

#### Pseuderannis lomozemia

(Prout, 1930)

516C72F9-574B-5BDE-BD9B-C923C6DD666A

##### Notes

Kwon (2022)

#### Rikiosatoa grisea

(Butler, 1878)

9D567DA2-6160-5ED3-A296-AF50A8949426

##### Notes

Lim et al. (2013)

#### Scionomia mendica

(Butler, 1879)

CBE873ED-53F1-5743-A317-E728B2E56D7D

##### Notes

Lim et al. (2013)

#### Scopula floslactata

(Haworth, 1809)

F84A112E-840F-527B-AF19-ABF877E24F9A

##### Notes

Kwon (2022)

#### Scopula ignobilis

(Warren, 1901)

EF0CA8EE-BB67-5BFE-A209-C267E5ACBA60

##### Notes

HNIBR (2022), Info (2023)

#### Scopula nigropunctata

(Hüfnagel, 1767)

CAE2FF9A-A310-55AF-B030-6FDA079960CB

##### Notes

Lim et al. (2013)

#### Taeniophila unio

(Oberthür, 1880)

258FC75F-8EEB-556B-924A-641E345DDADD

##### Notes

NSMK (2023a)

#### Thinopteryx crocoptera

(Kollar, 1844)

64E4F00E-E455-55A3-B518-FDBD96931703

##### Notes

Lim et al. (2013)

#### Timandra comptaria

Walker, 1863

DE804C53-A80D-5791-9B56-5878D123841E

##### Notes

Lim et al. (2013)

#### Tyloptera bella

(Butler, 1878)

358C3A94-EE9C-565A-A6B7-00E4300FD64F

##### Notes

Lim et al. (2013)

#### Wilemania nitobei

(Nitobe, 1907)

DD3F53EB-0456-5B69-9678-35F79BD6A225

##### Notes

Kwon (2022)

#### Xandrames dholaria

Moore, 1868

8DAEA13D-3A7B-5E1D-A62B-7C711BD6D9F3

##### Notes

Lim et al. (2013)

#### Xanthorhoe hortensiaria

(Graeser, 1890)

1840CA12-8689-56C2-A663-B8DDAC19D945

##### Notes

Byun et al. (1996), Kwon (2022)

#### Xanthorhoe saturata

(Guenée, 1858)

4A39CEC0-3EBB-5D66-A3CA-3BD9FC80AFEE

##### Notes

Lim et al. (2013)

#### Xerodes albonotaria

(Bremer, 1864)

2E5B0B99-4F76-57C1-B92A-CD4DBF32D670

##### Notes

HNIBR (2022), Info (2023)

#### Xerodes rufescentaria

(Motschulsky, 1861)

01B7EE38-E4FF-5A69-A57E-D426BBF8245B

##### Notes

Byun et al. (1996), Kwon (2022)

#### Gracillariidae

6A37B601-40A2-5496-8D64-704091C98106

#### Caloptilia aceris

Kumata, 1966

E9C353CD-2571-5698-8DD0-8A5679159D9B

##### Notes

Lim et al. (2013)

#### Hesperiidae

A9D518E7-26E3-5D34-BBC1-8E8F74F9102E

#### Parnara guttata

(Bremer et Grey, 1852)

ADFEC102-707F-549A-AFEF-58817D84C22A

##### Notes

Seok (1938), Cho (1955), Cho (1965), Lee and Kwon (1981), ME (1993), Kwon et al. (1996), Lee and Jung (2001), Lim et al. (2013), HNIBR (2022)

#### Lasiocampidae

AD31969A-8DB2-5CCE-8420-5FD349027F3A

#### Dendrolimus spectabilis

(Butler, 1877)

6CDA2977-B719-5F39-8623-ACC3DFC2AB97

##### Notes

Kwon et al. (1996), Lee and Jung (2001)

#### Limacodidae

DC8761A2-52F7-58F4-8241-13D209CA00AB

#### Ceratonema christophi

(Graeser, 1888)

E1E3264A-B8C6-5239-BBAF-AE4095BF573F

##### Notes

Lim et al. (2013)

#### Microleon longipalpis

Butler, 1885

FC0B1D3E-432A-5FA0-A69C-DB379BF3B9A3

##### Notes

Kwon (2022)

#### Monema flavescens

Walker, 1855

BEA49D69-655C-53C0-A6C8-534335681C46

##### Notes

Cho (1965), Lee and Kwon (1981), ME (1993), Kwon et al. (1996), Lee and Jung (2001)

#### Phrixolepia sericea

Butler, 1877

0D547C5C-718D-5870-A9C1-1819EE7C09CF

##### Notes

Kwon (2022)

#### Lycaenidae

52EC3BAD-55FD-5CCC-B84B-815F58F2FB21

#### Celastrina argiolus

(Linnaeus, 1758)

4DE7FD2C-8778-5759-8E8E-F5C7F6FECA25

##### Notes

Cho (1929), Seok (1938), Cho (1955), Cho (1965), Lee and Kwon (1981), ME (1993), Kwon et al. (1996), Lee and Jung (2001), Lim and Lee (2012), Lim et al. (2013), HNIBR (2022)

#### Cupido argiades

(Pallas, 1771)

97B16C57-936B-5AD3-9307-77E2F556D2E6

##### Notes

Cho (1929), Masaki (1934), Masaki (1936), Seok (1938), Cho (1955), Cho (1965), Lee and Kwon (1981), ME (1993), Kwon et al. (1996), Lee and Jung (2001), Lim and Lee (2012), HNIBR (2022), Won et al. (2023)

#### Favonius orientalis

(Murray, 1875)

4BBF633A-059B-5135-ABBD-E89B6D0960DB

##### Notes

HNIBR (2022)

#### Rapala arata

(Bremer, 1861)

8692A4C2-5F96-5169-8359-997AFDA8E578

##### Notes

Seok (1938), Cho (1965), Lee and Kwon (1981), ME (1993), Byun et al. (1996), Kwon et al. (1996), Lee and Jung (2001), Lim and Lee (2012), HNIBR (2022)

#### Rapala caerulea

(Bremer & Grey, [1852])

68B0240A-82FC-5544-86F4-FC7DBF6811D0

##### Notes

Cho (1955), Lee and Kwon (1981), ME (1993), Kwon et al. (1996), Lee and Jung (2001), Lim et al. (2013)

#### Scolitantides orion

(Pallas, 1771)

11228E59-3698-5140-A0B1-56B07D07E957

##### Notes

Seok (1938), Cho (1955), Cho (1965), Lee and Kwon (1981), ME (1993), Kwon et al. (1996), Lee and Jung (2001), Byun et al. (1996), Lim and Lee (2012), HNIBR (2022)

#### Taraka hamada

(Druce, 1875)

FB5594FF-46D4-5BBC-BBE0-3182BA61B69C

##### Notes

Cho (1955), Cho (1965), Lee and Kwon (1981), ME (1993), Kwon et al. (1996), Lee and Jung (2001), HNIBR (2022)

#### Zizeeria maha

(Kollar, 1844)

0445A978-00AA-5DF3-8878-3B7C8C772CCE

##### Notes

Seok (1938), Cho (1955), Cho (1965), Lee and Kwon (1981), ME (1993), Byun et al. (1996), Kwon et al. (1996), Lee and Jung (2001), Lim and Lee (2012), Lim et al. (2013), HNIBR (2022), Won et al. (2023)

#### Lymantriidae

B3EE958F-2580-5451-BC5E-8334B33C9816

#### Somena pulverea

(Leech, 1889)

20947D62-8704-5195-847C-8CA9006C81F2

##### Notes

Byun et al. (1996), Lim and Lee (2012), Lim et al. (2013), HNIBR (2022)

#### Lyonetiidae

CBFB7F7F-BA4A-5474-86CF-B8478E27B5FB

#### Lyonetia clerkella

(Linnaeus, 1758)

F42E52B2-6311-5A3E-A345-F4252DFD1011

##### Notes

Lim et al. (2013)

#### Noctuidae

F5DD9F6A-B5F1-5800-98BE-9D84C5073D84

#### Abrostola ussuriensis

Dufay, 1958

3E8FAC6A-C3CF-5E0C-9AB2-C6902093D13E

##### Notes

Lee and Jung (2001), Lim et al. (2013)

#### Acosmetia chinensis

(Wallengren, 1860)

D1B9D4FE-C1CA-58CE-93A3-3EC1CE079EDD

##### Notes

Kwon (2022)

#### Acronicta concrepta

Draudt, 1937

FADF6069-619E-59B3-BB6C-B00EBB7B9389

##### Notes

Lim et al. (2013)

#### Acronicta cuspis

(Hübner, 1813)

0A3FCAA4-E940-5743-A0DF-4B0F505EEA66

##### Notes

Lim et al. (2013)

#### Acronicta hercules

(Felder & Rogenhofer, 1874)

C0AD6746-D99F-5C9C-B895-08176EC1A675

##### Materials

- **Type status:** Other material. **Occurrence:** recordedBy: Lee, D. Y.; sex: 1 male; lifeStage: adult; occurrenceID: 1292E45B-7026-517C-A5AF-0F5E70EA4C55; **Location:** island: Ulleungdo Island; country: Republic of Korea; stateProvince: Gyeongsangbuk-do; county: Ulleung-gun; locality: Taeharyeong, Namseo-ri, Seomyeon; **Event:** samplingProtocol: Light trap; eventDate: 6/20/2021

##### Notes

This is the first record of this species from Ulleungdo Island.

Distribution: China, Japan, Korea, Russia (Han and Kononenko 2010)

#### Acronicta leucocuspis

(Butler, 1878)

53E8A775-F999-5574-BD7E-A9B1E1DE3F88

##### Notes

HNIBR (2022)

#### Acronicta major

(Bremer, 1861)

A9B6BE6C-C0B6-5C37-B8A5-F6ED71B8A2D1

##### Notes

Lee and Jung (2001), Lim et al. (2013)

#### Acronicta rumicis

(Linnaeus, 1758)

58D7A799-691E-5DD8-B552-F58F1EFAA6F8

##### Notes

Kwon (2022)

#### Agrotis ipsilon

(Hufnagel, 1766)

6B690E7B-853B-5DEF-9AF5-D0909FF01C70

##### Notes

Lee and Jung (2001), Lim et al. (2013)

#### Agrotis segetum

(Denis & Schiffermuller, 1775)

063EDDEB-DF67-58F6-981A-F62DEBDD4F4F

##### Notes

Lim and Lee (2012), HNIBR (2022)

#### Amphipyra acheron

Draudt, 1950

5C7EAC87-86FD-5F35-B7E2-6BBE9089D09B

##### Notes

Byun et al. (1996), Lim et al. (2013)

#### Amphipyra livida

(Denis & Schiffermüller) 1775

6E7469B4-22CD-5F27-BF5E-CBECA9BD6160

##### Notes

Lim et al. (2013), Won et al. (2023)

#### Amphipyra pyramidea

(Linnaeus, 1758)

1DF328B0-F031-5BF0-B900-BF5DCC6B3285

##### Notes

Lee and Jung (2001)

#### Amphipyra schrenckii

Ménétriés, 1859

705FC703-394D-5000-B925-39F98DF3FB65

##### Notes

Kwon (2022)

#### Analetia postica

(Hampson, 1905)

786966E0-4ADD-5840-9BE2-6A0EA7A263E1

##### Materials

- **Type status:** Other material. **Occurrence:** recordedBy: Lee, D. Y.; sex: 1 male; lifeStage: adult; occurrenceID: BAC1694B-90E0-559B-A1BA-5F2D7C8127EE; **Location:** island: Ulleungdo Island; country: Republic of Korea; stateProvince: Gyeongsangbuk-do; county: Ulleung-gun; locality: Taeharyeong, Namseo-ri, Seomyeon; **Event:** samplingProtocol: Light trap; eventDate: 8/17/2021

##### Notes

This is the first record of this species from Ulleungdo Island.

Distribution: China, Japan, Korea, Russia (Bálint et al. 2014)

#### Anaplectoides virens

(Butler, 1878)

332D799B-7C53-5C13-87A4-BCF940214363

##### Notes

Kwon (2022)

#### Antivaleria viridimacula

(Graeser, 1889)

8823679A-193D-5648-A9AD-1C842369FCE7

##### Notes

Kwon (2022)

#### Antoculeora locuples

(Oberthür, 1880)

2360DA2C-C6C1-5750-85A6-77819C76A0AE

##### Notes

Lee and Jung (2001), Lim et al. (2013), Won et al. (2023)

#### Apamea striata

Haruta, 1958

57BC04B8-7CCF-5F33-BCAE-D003FF94781C

##### Notes

Lee and Jung (2001)

#### Athetis albisignata

(Oberthür, 1879)

FA2B594D-DFE7-56BD-A0B0-032E32860525

##### Notes

Kwon (2022)

#### Athetis lineosa

(Moore, 1881)

9BC25385-6C5D-5FCA-B9E6-E34C07439562

##### Notes

Lee and Jung (2001), Won et al. (2023)

#### Athetis stellata

(Moore, 1882)

8B36DBF6-828E-50BE-93D6-64ED77E691A2

##### Notes

Cho (1965), Lee and Kwon (1981), ME (1993), Kwon et al. (1996), Lee and Jung (2001), HNIBR (2022)

#### Atrachea nitens

(Butler, 1878)

63C6AB1F-DC9A-5039-8CF5-E2504F47197A

##### Notes

Kwon (2022)

#### Auchmis saga

(Butler, 1878)

E095C8B7-3581-5925-A8C5-C5DC1D8B5228

##### Notes

Lim et al. (2013)

#### Bryophila granitalis

(Butler, 1881)

CB5DF9D8-BFAA-527F-A073-3D2785C0CDCD

##### Notes

Lim et al. (2013)

#### Callopistria aethiops

Butler, 1878

074C7441-09E9-55F1-9FDE-D6629D8CCF9A

##### Notes

Lim et al. (2013)

#### Callopistria albolineola

(Graeser, 1889)

A41408B8-0E85-5633-A005-0C9B97030528

##### Notes

Lim et al. (2013)

#### Callopistria duplicans

Walker, 1858

05F4D4DE-EE3D-5D3D-8061-5D625B06D189

##### Notes

Lim et al. (2013)

#### Callopistria juventina

(Stoll, 1782)

2030B000-201E-5332-80B6-449EDAC9880F

##### Notes

Lim et al. (2013)

#### Callopistria repleta

Walker, 1858

CA8A90C1-CF1F-51D0-A105-35A668AE06F6

##### Notes

Lim et al. (2013), Won et al. (2023)

#### Chasminodes albonitens

(Bremer, 1861)

967A615D-29C5-57E2-B461-DA41800E267A

##### Notes

Lim et al. (2013), HNIBR (2022), Won et al. (2023)

#### Chrysodeixis eriosoma

(Doubleday, 1843)

62A7CE76-7223-54E9-9E50-CAF480B2B2E7

##### Notes

Lim et al. (2013), Won et al. (2023)

#### Chytonix albonotata

(Staudinger, 1892)

F30C0B9F-CC22-5D0A-AAA1-7A2796AC1943

##### Notes

Lim et al. (2013)

#### Clavipalpula aurariae

(Oberthür, 1880)

E6B85466-1A27-5B37-8C06-2C5E076145F2

##### Notes

Byun et al. (1996), HNIBR (2022), Kwon (2022)

#### Cosmia achatina

Butler, 1879

0C4DBCA1-8AD9-5BCC-80D4-FC6B309B57CE

##### Notes

Lim et al. (2013)

#### Cosmia affinis

(Linnaeus, 1767)

6FB4CB74-2AD5-5272-834B-2175C3A00D33

##### Notes

Lim et al. (2013)

#### Cosmia trapezina

(Linnaeus, 1758)

D14972CB-78DF-5B6E-8C2A-0D986155349A

##### Notes

Lee and Jung (2001), Kwon (2022)

#### Cosmia unicolor

(Staüdinger, 1892)

0BC4D8EA-72D0-5C20-AFCB-A95207D1C1C8

##### Notes

Lee and Jung (2001)

#### Cryphia bryophasma

(Boursin, 1951)

D4994E9A-982E-5EAC-BB3C-69C739731E22

##### Notes

Kwon (2022)

#### Cryphia mitsuhashi

(Marumo, 1917)

543800BC-6D48-56B3-A941-F9BC1F68ABA6

##### Notes

Lim et al. (2013)

#### Ctenoplusia agnata

(Staüdinger, 1892)

85BBFC8C-7CBE-5E0A-B3A2-4E08AAEA3663

##### Notes

Lim et al. (2013)

#### Ctenoplusia albostriata

(Bremer & Grey, 1853)

6EC225CD-52CC-5706-863D-1B09C692B5CB

##### Notes

Lim et al. (2013), Won et al. (2023)

#### Cucullia perforata

Bremer, 1861

2A1229CE-5E77-538D-AA65-44DEEFC1221C

##### Notes

Lim et al. (2013)

#### Daddala lucilla

(Butler, 1881)

BC115020-B0E9-51D1-9F1B-DD5E176C1634

##### Notes

Lim et al. (2013)

#### Diarsia canescens

(Butler, 1878)

8392CFA6-4F8E-51C5-AB86-F943E812845E

##### Notes

HNIBR (2022), Kwon (2022), Won et al. (2023)

#### Diarsia deparca

(Butler, 1879)

9CEA3809-0CEB-5B1B-8ABB-C434916A84D8

##### Notes

Won et al. (2023)

#### Dichagyris triangularis

(Moore, 1867)

486F2845-C69C-52DD-8FE3-77A4FC28A8B2

##### Notes

Kwon (2022)

#### Dictyestra dissectus

(Walker, 1865)

812D3BC9-65EE-5B84-956C-AD2006686380

##### Notes

Won et al. (2023)

#### Dimorphicosmia variegata

(Oberthür, 1879)

CCA52FDD-7A02-57E6-931D-E6E467DA1F34

##### Notes

Won et al. (2023)

#### Dypterygia caliginosa

(Walker, 1858)

E00B5A1A-0B0D-5B71-9424-DBB8FF841A89

##### Notes

Lim et al. (2013), Won et al. (2023)

#### Eucarta fasciata

(Butler, 1878)

451BC993-E305-53EC-9E94-3060D3A531A2

##### Notes

Info (2023)

#### Euplexia lucipara

(Linnaeus, 1758)

1EF7FEB8-FBB3-54D0-8E6E-B0EAD0DE83BF

##### Notes

Won et al. (2023)

#### Hadena variolata

(Smith, 1888)

F2A5F39D-EC59-50CC-84ED-4E65BDF50531

##### Notes

Info (2023)

#### Hadena illoba

(Butler, 1878)

15FBB06D-F075-5684-99B9-E0034B4F5683

##### Notes

Kim (1971), Lee and Kwon (1981), ME (1993), Kwon et al. (1996), Lee and Jung (2001), Lim et al. (2013)

#### Hermonassa cecilia

Butler, 1878

36D86DF9-F89D-5D2C-8359-5AE908BA5378

##### Notes

Lim et al. (2013)

#### Hydrillodes lentalis

Guenée, 1854

C0178315-A2DC-59A8-9339-B3DF8E25F6C2

##### Notes

Cho (1965), Lee and Kwon (1981), ME (1993), Kwon et al. (1996), Lee and Jung (2001)

#### Iambia japonica

Sugi, 1958

C17EDAAD-3565-5998-B62C-F3DC62E8ADF1

##### Notes

Lim et al. (2013)

#### Lacanobia splendens

(Hübner, 1808)

2D6F2EBB-81BB-5EC3-A344-A3AFDAD898C9

##### Notes

Info (2023)

#### Macdunnoughia purissima

(Butler, 1878)

FB101744-F2FD-51F7-AA71-61458CAA628D

##### Notes

Kwon (2022)

#### Maliattha signifera

(Walker, 1858)

89834455-7F2B-5455-B986-0B9F4BEF0539

##### Notes

Lim et al. (2013)

#### Mamestra brassicae

(Linnaeus, 1758)

AAC1B454-9928-53F5-A5D1-249482F1E4AE

##### Notes

Lim et al. (2013)

#### Mesoligia fodinae

(Oberthür, 1880)

46E6FB72-7C43-5C58-8234-4FA283E32EBF

##### Notes

Lim et al. (2013)

#### Mythimna divergens

Butler, 1878

66C4FA35-EAC7-5606-A2B1-11825FAE148A

##### Notes

Lee and Jung (2001)

#### Mythimna monticola

Sugi, 1958

03B0655E-8B6A-57CF-ABA8-DAEF9CCD7ACC

##### Notes

Kwon (2022)

#### Mythimna separata

(Walker, 1865)

C16F398E-D578-5735-A5B3-57B856C7F729

##### Notes

Kim (1971), Lee and Kwon (1981), ME (1993), Kwon et al. (1996), Lee and Jung (2001), Lim et al. (2013), HNIBR (2022)

#### Mythimna stolida

(Leech, 1889)

C4642EED-1E67-576D-925F-35DCBB046A3B

##### Notes

Lee and Jung (2001)

#### Mythimna striata

Leech, 1900

81736396-D40A-5416-BF76-88B54E9C67C1

##### Notes

Lee and Jung (2001)

#### Mythimna turca

(Linnaeus, 1761)

A988B885-71EF-533C-865A-7E94FABA87EB

##### Notes

Kwon (2022)

#### Niphonyx segregata

(Butler, 1878)

14207E81-9E05-5FE2-B2EF-4D494BC67A17

##### Notes

Lim et al. (2013)

#### Olivenebula oberthueri

(Staüdinger, 1892)

A19DB60A-272F-5976-84D5-E9641DAA9AA9

##### Notes

Lim et al. (2013)

#### Orthogonia sera

Felder, 1862

220A161B-04DC-5CC0-80AA-1F4D07CA9B59

##### Notes

Kwon (2022)

#### Orthosia askoldensis

(Staüdinger, 1892)

405C41D1-CD07-5F1A-9AB3-BF0E69DDF455

##### Notes

Won et al. (2023)

#### Orthosia carnipennis

(Butler, 1878)

D1989A83-3E27-515E-97A8-E6FFFD673494

##### Notes

HNIBR (2022), Won et al. (2023)

#### Orthosia cedermarki

(Bryk, 1948)

2E348EB7-601F-5CC2-99FA-4DB808CFB08F

##### Notes

Kwon (2022)

#### Orthosia limbata

(Butler, 1879)

8CBB09D4-5623-5D84-9CBB-15901388B3CF

##### Notes

Info (2023)

#### Panolis japonica

Draudt, 1935

F9216933-582A-57CF-A427-0C34C275E53D

##### Notes

Kwon (2022)

#### Perigrapha hoenei

Püngeler, 1914

DA5F5A8F-0EA6-5BC9-92A3-E69C23882B2D

##### Notes

Kwon (2022)

#### Phlogophora illustrata

(Graeser, 1889)

F23E244C-CC62-5229-9723-A4772F8B5D16

##### Notes

Lim et al. (2013)

#### Polia hepatica

(Clerck, 1759)

E9EAF25B-7612-5F8F-A9E8-FF5CE68E224A

##### Notes

Lim et al. (2013)

#### Polychrysia splendida

(Butler, 1878)

A8658365-ECD2-5A0C-A56C-1DA246AD2927

##### Notes

Kwon (2022)

#### Sarbanissa venusta

(Leech, 1889)

4845BF70-7623-509F-B9A9-5DEFA7F615B8

##### Notes

Kim (1971), Lee and Kwon (1981), ME (1993), Kwon et al. (1996), Lee and Jung (2001), HNIBR (2022)

#### Sineugraphe exusta

(Butler, 1878)

82BAE813-5396-5148-AB36-069012028784

##### Notes

Kwon (2022)

#### Sineugraphe oceanica

(Kardakoff, 1928)

EC6F8CA0-9AB7-553D-99F4-EAAEF0718C52

##### Notes

Lim and Lee (2012), Lim et al. (2013), HNIBR (2022), Won et al. (2023)

#### Spaelotis lucens

Butler, 1881

C2E4A97D-0773-5F47-97F3-D83663571FBB

##### Notes

Cho (1955), Cho (1965), Lee and Kwon (1981), ME (1993), Kwon et al. (1996), Lee and Jung (2001)

#### Spodoptera depravata

(Butler, 1879)

822D9049-2F12-54FB-AA53-6B9F7C0DB7E2

##### Notes

Info (2023)

#### Stenoloba jankowskii

(Oberthür, 1884)

CC563370-804B-5DC9-ABA2-9618B1B12766

##### Notes

Lee and Jung (2001)

#### Triphaenopsis jezoensis

Sugi, 1962

8B5E3A73-503E-56D9-93AE-59F781838E21

##### Notes

Kwon (2022)

#### Triphaenopsis lucilla

Butler, 1878

61DD3E5A-052F-5A0A-9BDD-9488BBC257C6

##### Notes

Lee and Jung (2001)

#### Xenapamea pacifica

Sugi, 1970

53A319D8-961F-5680-AEBE-0E7C2AA782B1

##### Notes

Info (2023)

#### Xestia c-nigrum

(Linnaeus, 1758)

EF065F7B-09A1-5189-9FCE-B9B9F17D512B

##### Notes

Kim (1971), Lee and Kwon (1981), ME (1993), Kwon et al. (1996), Lee and Jung (2001), Lim et al. (2013), Won et al. (2023)

#### Xestia efflorescens

(Butler, 1879)

031E989F-0727-5735-8F68-BA3D0F6635DC

##### Notes

Lim et al. (2013), Won et al. (2023)

#### Nolidae

04CE35BD-DB0C-56B2-AF15-16A632587DFD

#### Blenina senex

(Butler, 1878)

695B2DB7-8239-5883-AF27-6B3B8D203BC4

##### Notes

Lim et al. (2013)

#### Earias roseifera

Butler, 1881

7724F7F3-D807-5C90-82F6-C11D27A42273

##### Notes

Kwon (2022)

#### Evonima mandschuriana

(Oberthür, 1880)

86FACDE0-2B57-5564-A218-04191698D2C3

##### Notes

Lim et al. (2013)

#### Pseudoips prasinana

(Linnaeus, 1758)

A69ED058-23AD-51E8-937A-8A117CD760A8

##### Notes

Byun et al. (1996), Lee and Jung (2001), Kwon (2021)

#### Notodontidae

334244F4-2364-5A39-9E68-64BB7BC895B5

#### Cerura menciana

Moore, 1887

9B352781-84D2-5A86-8EC2-24589D38CEF6

##### Notes

Byun et al. (1996), Park (2011)

#### Clostera anachoreta

(Denis & Schiffermüller, 1775)

DB1712C4-1C89-522A-9B09-D9BAC1209C32

##### Notes

Lim and Lee (2012), HNIBR (2022)

#### Epodonta lineata

(Oberthür, 1880)

DD77CB13-2F94-5737-A9FF-E91668F721AB

##### Notes

Lim et al. (2013), HNIBR (2022), Won et al. (2023)

#### Euhampsonia cristata

(Butler, 1877)

8EA400D3-16F8-527C-8B1D-8BEA9225140D

##### Notes

Kim (1971), Lee and Kwon (1981), ME (1993), Kwon et al. (1996), Lee and Jung (2001), Lim et al. (2013), Won et al. (2023)

#### Fentonia ocypete

(Bremer, 1861)

866791A9-0EA9-5E9B-8CC9-E7544100A1B0

##### Notes

Kwon (2022)

#### Phalera flavescens

(Bremer & Grey, 1853)

A0A3338F-751D-5C3A-9BAF-281B7B8175AC

##### Notes

Lim et al. (2013)

#### Ptilodon hoegei

(Graeser, 1888)

D1973086-A89A-5830-9620-87908E0D463A

##### Notes

HNIBR (2022), Kwon (2022)

#### Shaka atrovittatus

(Bremer, 1861)

306794E9-2421-5864-8FC1-3863C173C2F1

##### Notes

Byun et al. (1996), Park (2011), Lim and Lee (2012), HNIBR (2022)

#### Spatalia plusiotis

(Oberthür, 1880)

F6A88263-9E89-5835-9F82-695E68008C7A

##### Notes

Lee and Jung (2001), Lim et al. (2013), Won et al. (2023)

#### Stauropus fagi

(Linnaeus, 1758)

F91AEE69-1CED-5B68-9882-D37719275170

##### Notes

Kwon (2022)

#### Syntypistis cyanea

(Leech, 1889)

6C151AFD-9C7E-592A-8325-4337DBD03B65

##### Notes

Lee and Jung (2001), Lim and Lee (2012), Lim et al. (2013), HNIBR (2022)

#### Syntypistis pryeri

(Leech, 1889)

8CA37EAC-E02D-554A-95F9-719773483299

##### Notes

Lim and Lee (2012), HNIBR (2022)

#### Syntypistis subgeneris

(Strand, 1915)

2DE83B0D-0FAE-545A-A689-29A0F9C063F0

##### Notes

Kwon (2022)

#### Zaranga tukuringra

Streltzov & Yakovlev, 2007

8CCE4FC1-C971-5A30-9B2F-A5616DD0E284

##### Notes

Lee and Jung (2001)

#### Nymphalidae

267EC673-6A53-5A3C-A16A-6D6BC784D428

#### Argynnis paphia

(Linnaeus, 1758)

EC4F83B5-48B8-5FDD-841B-8906FBEDF2DE

##### Notes

Cho (1929), Seok (1938), Cho (1955), Cho (1965), Lee and Kwon (1981), ME (1993), Kwon et al. (1996), Lee and Jung (2001), Lim et al. (2013), HNIBR (2022)

#### Argyreus hyperbius

(Linnaeus, 1763)

9A45ACA3-3A19-5C32-B834-DD5AD28A60EE

##### Notes

Seok (1938), Cho (1955), Cho (1965), Lee and Kwon (1981), ME (1993), Kwon et al. (1996), Lee and Jung (2001), HNIBR (2022)

#### Argyronome laodice

(Pallas, 1771)

308F412F-C385-5760-9761-5BDE01CDE84A

##### Notes

Lee and Kwon (1981), ME (1993), Kwon et al. (1996), Lee and Jung (2001), HNIBR (2022)

#### Chitoria ulupi

(Doherty, 1889)

038DE2E8-66B3-5730-8CE9-19BDE28356A2

##### Notes

Lee and Kwon (1981), ME (1993), Kwon et al. (1996), Lee and Jung (2001), HNIBR (2022)

#### Kaniska canace

(Linnaeus, 1763)

76F49C77-69A6-5A58-9646-6FF3759E4C81

##### Notes

Seok (1938), Cho (1955), Cho (1965), Lee and Kwon (1981), ME (1993), Kwon et al. (1996), Lee and Jung (2001), Lim and Lee (2012), Lim et al. (2013), HNIBR (2022), Won et al. (2023)

#### Kirinia epimenides

(Ménétriès, 1859)

97198A97-8AE3-50D2-82B3-A7480CED7ECA

##### Notes

Lee and Kwon (1981), ME (1993), Byun et al. (1996), Kwon et al. (1996), Lee and Jung (2001), HNIBR (2022)

#### Limenitis helmanni

Lederer, 1853

AE59F834-1E0B-5EF4-9360-833CA6BBFB57

##### Notes

Cho (1955), Cho (1965), Lee and Kwon (1981), ME (1993), Kwon et al. (1996), Lee and Jung (2001), HNIBR (2022)

#### Melanitis leda

(Linnaeus, 1758)

0FC57649-8B90-5C19-A894-2B5B6591BF5F

##### Notes

HNIBR (2022)

#### Minois dryas

(Scopoli, 1763)

E0A6235B-9036-513D-BF45-8597B9BAD6BD

##### Notes

Cho (1929), Seok (1938), Cho (1955), Cho (1965), Lee and Kwon (1981), ME (1993), Byun et al. (1996), Kwon et al. (1996), Lee and Jung (2001), Lim et al. (2013), HNIBR (2022), Won et al. (2023)

#### Neptis alwina

(Bremer et Grey, 1852)

80B4272A-2D23-51D4-9D43-679D9515B3BC

##### Notes

Lee and Kwon (1981), ME (1993), Kwon et al. (1996), Lee and Jung (2001), HNIBR (2022)

#### Neptis sappho

(Pallas, 1771)

EE64F9D1-C1DF-5885-8D96-FA2A46C15469

##### Notes

Cho (1929), Seok (1938), Cho (1955), Cho (1965), Lee and Kwon (1981), ME (1993), Kwon et al. (1996), Lee and Jung (2001), Lim and Lee (2012), HNIBR (2022)

#### Parantica sita

(Kollar, [1844])

B6530BC2-B0AE-583C-B575-3FA12F4900BF

##### Notes

Kim (1971), Lee and Kwon (1981), ME (1993), Kwon et al. (1996), Lee and Jung (2001), HNIBR (2022)

#### Polygonia c-album

(Linnaeus, 1758)

B0B32850-C42A-5B06-922C-F0EFF1C42FDC

##### Notes

HNIBR (2022)

#### Polygonia c-aureum

(Linnaeus, 1758)

80EB4104-4CE5-5505-B465-DD5C2CCB4DFA

##### Notes

Cho (1929), Seok (1938), Cho (1955), Cho (1965), Lee and Kwon (1981), ME (1993), Kwon et al. (1996), Lee and Jung (2001), Lim et al. (2013), HNIBR (2022)

#### Vanessa cardui

(Linnaeus, 1758)

56609AB9-29B9-5C43-953F-2363C6A8EF8B

##### Notes

Cho (1929), Seok (1938), Cho (1955), Cho (1965), Lee and Kwon (1981), ME (1993), Kwon et al. (1996), Lee and Jung (2001), Lim and Lee (2012), Lim et al. (2013), HNIBR (2022)

#### Vanessa indica

(Herbst, 1794)

7CE8DB0B-648E-5E9E-B33A-0CEA0710E325

##### Notes

Cho (1929), Masaki (1934), Masaki (1936), Seok (1938), Cho (1955), Cho (1965), Lee and Kwon (1981), ME (1993), Kwon et al. (1996), Lee and Jung (2001), Lim and Lee (2012), Lim et al. (2013), HNIBR (2022)

#### Oecophoridae

D816BD5A-9717-54D2-A2DA-96444E9929A4

#### Pedioxestis isomorpha

Meyrick, 1932

34087587-5973-5040-B5AA-A4CADF1EC024

##### Notes

Info and Park (2023)

#### Papilionidae

7C025726-0D55-52EB-83BD-DF8FA5146F42

#### Graphium sarpedon

(Linnaeus, 1758)

21D0752C-C185-5020-A755-9A6897B8A47B

##### Notes

Cho (1929), Masaki (1936), Seok (1938), Cho (1955), Cho (1965), Lee and Kwon (1981), ME (1993), Kwon et al. (1996), Lee and Jung (2001), Lim and Lee (2012), HNIBR (2022)

#### Papilio bianor

Cramer, [1777]

41D694A2-2D19-56DA-8B7D-660C0DE86D05

##### Notes

Seok (1938), Cho (1955), Cho (1965), Lee and Kwon (1981), ME (1993), Kwon et al. (1996), Lee and Jung (2001), Lim and Lee (2012), Lim et al. (2013), HNIBR (2022)

#### Papilio maackii

Ménétriés, 1859

2DA2983A-3B43-5621-9ABE-2B046C456089

##### Notes

Cho (1929), Seok (1938), Cho (1955), Cho (1965), Lee and Kwon (1981), ME (1993), Byun et al. (1996), Kwon et al. (1996), Lee and Jung (2001), Lim and Lee (2012), Lim et al. (2013), HNIBR (2022)

#### Papilio xuthus

Linnaeus, 1767

9E367A07-2EC1-5C6C-BA95-AD5823EBC11C

##### Notes

Cho (1929), Seok (1938), Cho (1955), Cho (1965), Lee and Kwon (1981), ME (1993), Byun et al. (1996), Kwon et al. (1996), Lee and Jung (2001), Lim and Lee (2012), Lim et al. (2013), HNIBR (2022)

#### Pieridae

C0C3F2CF-8331-5D17-8740-07AAA195B849

#### Anthocharis scolymus

Butler, 1866

0CECF019-161A-55E0-91DB-E2A7BA907F75

##### Notes

Seok (1938), Cho (1955), Cho (1965), Lee and Kwon (1981), ME (1993), Kwon et al. (1996), Lee and Jung (2001), Lim and Lee (2012), HNIBR (2022), Won et al. (2023)

#### Colias erate

(Esper, [1805])

799D6ED1-4F80-500E-855E-4B0DD0A9C228

##### Notes

Cho (1929), Masaki (1934), Masaki (1936), Cho (1955), Cho (1965), Lee and Kwon (1981), ME (1993), Byun et al. (1996), Kwon et al. (1996), Lee and Jung (2001), Lim and Lee (2012), Lim et al. (2013), HNIBR (2022)

#### Eurema brigitta

(Stoll, [1780])

C80FEC27-6C3F-5E27-A050-E240BFBE5A25

##### Notes

HNIBR (2022)

#### Eurema mandarina

(De L’Orza, 1869)

477E0650-C169-53B2-813A-0684D0C260D8

##### Notes

Cho (1955), Cho (1965), Lee and Kwon (1981), ME (1993), Kwon et al. (1996), Lee and Jung (2001), HNIBR (2022)

#### Pieris canidia

(Sparrman, 1768)

F9E0300D-F978-57E8-B64B-AB2D473045B7

##### Notes

Cho (1929), Cho (1955), Cho (1965), Lee and Kwon (1981), ME (1993), Byun et al. (1996), Kwon et al. (1996), Lee and Jung (2001), Lim et al. (2013), HNIBR (2022)

#### Pieris melete

Ménétriès, 1857

A3AC715D-ABF9-5351-9A9E-EF49111B1A3D

##### Notes

Lee and Kwon (1981), ME (1993), Byun et al. (1996), Kwon et al. (1996), Lee and Jung (2001), Lim and Lee (2012), Lim et al. (2013), HNIBR (2022)

#### Pieris napi

(Linnaeus, 1758)

3D48458A-7024-5886-9127-769275CB90B5

##### Notes

Cho (1929), Cho (1955), Cho (1965), Lee and Kwon (1981), ME (1993), Byun et al. (1996), Kwon et al. (1996), Lee and Jung (2001), HNIBR (2022)

#### Pieris rapae

(Linnaeus, 1758)

CA7D3D2C-1FDE-5662-8369-5501D0A86022

##### Notes

Masaki (1934), Seok (1938), Cho (1955), Cho (1965), Lee and Kwon (1981), ME (1993), Byun et al. (1996), Kwon et al. (1996), Lee and Jung (2001), Lim and Lee (2012), Lim et al. (2013), HNIBR (2022), Won et al. (2023)

#### Plutellidae

B3759FF5-05D4-580F-9C3C-8C049E2D38B7

#### Plutella xylostella

(Linnaeus, 1767)

6B693AFE-D255-5107-84AE-5B38652ABE5E

##### Notes

Byun et al. (1996), Lim et al. (2013)

#### Pterophoridae

03BA1060-BAB0-530C-89C5-3BC0E140B1DB

#### Petrophora chlorosata

(Scopoli, 1763)

922FFF57-5C49-50A1-BD0A-15B03A220E23

##### Notes

Info (2023)

#### Pyralidae

15750709-6FC1-5890-B106-7316750A0C78

#### Aglossa dimidiata

(Haworth, 1809)

18AF862F-64EE-5104-8744-CE0167F3FBDB

##### Notes

Cho (1965), Lee and Kwon (1981), ME (1993), Byun et al. (1996), Kwon et al. (1996), Lee and Jung (2001)

#### Arippara indicator

Walker, 1864

0B18CF39-DFBF-5D75-BEB8-46278D450F9C

##### Notes

Kwon (2022)

#### Calguia defiguralis

Walker, 1863

7021E496-2815-53FE-BAE5-ECB61BB62B3C

##### Notes

Byun et al. (1996), Lim et al. (2013)

#### Cataprosopus monstrosus

Butler, 1881

94A55762-41B6-5C59-A381-21632B3558CB

##### Notes

Lim et al. (2013)

#### Dioryctria abietella

(Denis & Schiffermüller, 1775)

4DA8522B-F432-57AA-B046-3936B663AB4A

##### Notes

Lim et al. (2013)

#### Dioryctria sylvestrella

(Ratzeburg, 1840)

326A5365-ABCB-5AE4-B551-BB4B5066C079

##### Notes

Lim et al. (2013)

#### Endotricha olivacealis

Bremer, 1864

7ED8BF89-8A67-548D-A7CA-73F01F7114F5

##### Notes

Lim et al. (2013)

#### Hypsopygia glaucinalis

(Linnaeus, 1758)

7111F36E-BB9F-5206-88AB-5907B71B58BC

##### Notes

Kim (1971), Lee and Kwon (1981), ME (1993), Byun et al. (1996), Kwon et al. (1996), Lee and Jung (2001), Lim et al. (2013), HNIBR (2022)

#### Hypsopygia iwamotoi

Kirpichnikova & Yamanaka, 1995

81AE95A5-4393-57B4-B0E4-177EDE7810FB

##### Notes

Lim et al. (2013)

#### Lamoria glaucalis

Caradja, 1925

EF29A019-6B29-5996-A84C-ED1878C1051B

##### Notes

Kwon (2022)

#### Nyctegretis triangulella

Ragonot, 1901

0E628A9B-8C57-56D1-862E-28B9C285A175

##### Notes

Lim et al. (2013)

#### Oncocera semirubella

(Scopoli, 1763)

BF2CBED4-A4AF-5ED2-A447-FBBFA6DE445E

##### Notes

Kwon (2022)

#### Orthaga onerata

Butler, 1879

D4E17D4E-335C-5C87-8D40-F01489F9A3FA

##### Notes

Lim et al. (2013)

#### Phycitodes subcretacellus

(Ragonot, 1901)

BD19369D-C4D4-5F21-A92E-1F9354DDB25F

##### Notes

Lim et al. (2013)

#### Pyralis regalis

Denis & Schiffermüller, 1775

646A0FC9-B497-5029-97C6-BDB996DB05B9

##### Notes

Lim et al. (2013)

#### Stericta melanobasis

(Hampson, 1906)

560293D9-74C0-54F0-9C73-227AF1F7A04A

##### Notes

Lim and Lee (2012), HNIBR (2022)

#### Termioptycha nigrescens

(Warren, 1891)

F081D78F-CBAA-5158-A6D2-ECB07D61DFD4

##### Notes

Byun et al. (1996), Lim et al. (2013)

#### Saturniidae

88A9A03A-27D4-50D8-9E5B-80DC23DCA59C

#### Samia cynthia

(Drury, 1773)

D5EDB590-84C0-5334-9F78-8DF63FAEB55A

##### Notes

Kwon et al. (1996), Lee and Jung (2001), Lim et al. (2013), Won et al. (2023)

#### Scythrididae

EF551487-E409-56C3-8334-6EE00000C84B

#### Scythris sinensis

(Felder & Rogenhofer, 1875)

09DF252D-CFFD-52A7-A311-66BB70A93A8C

##### Notes

Lim et al. (2013)

#### Sesiidae

6DD0F080-4EC5-5515-9B35-A1E751FF2973

#### Synanthedon bicingulata

(Staudinger, 1887)

8A37C2F4-225D-5EB3-A5D0-C5C29C0BA83B

##### Notes

Kim (1971), Lee and Kwon (1981), ME (1993), Byun et al. (1996), Kwon et al. (1996), Lee and Jung (2001), Lim et al. (2013)

#### Sphingidae

79CB851A-FD4E-543F-B9BD-920E6A55E32A

#### Acosmeryx naga

(Moore, 1857)

485C4764-8443-53FB-83E9-FB3A9D7A1454

##### Notes

Byun et al. (1996), Kwon et al. (1996), Lee and Jung (2001), Lim and Lee (2012), Lim et al. (2013), HNIBR (2022), Won et al. (2023)

#### Ambulyx japonica koreana

Inoue, 1993

33DF5FB8-11E0-5B4D-B9DE-794531C9158A

##### Notes

Kwon et al. (1996), Lee and Jung (2001), Lim and Lee (2012), Lim et al. (2013), HNIBR (2022), Won et al. (2023)

#### Ambulyx ochracea

Butler, 1885

7E7C1D3D-5869-507B-8277-3E41C31D84C4

##### Notes

Kwon et al. (1996), Lee and Jung (2001)

#### Ampelophaga rubiginosa

Bremer & Grey, [1852]

F352152D-7F57-5508-A6EC-AFEDD1E36D9D

##### Notes

Kim (1971), Lee and Kwon (1981), ME (1993), Kwon et al. (1996), Lee and Jung (2001), HNIBR (2022)

#### Callambulyx tatarinovii

(Bremer & Grey, 1852)

F1B15436-5268-5E12-9EC8-F0A247A68ACC

##### Notes

Kim (1971), Lee and Kwon (1981), ME (1993), Kwon et al. (1996), Lee and Jung (2001), Lim and Lee (2012), Lim et al. (2013), HNIBR (2022), Won et al. (2023)

#### Clanis bilineata

(Walker, 1886)

C102D458-087B-55DC-8AA4-1636195B5B44

##### Notes

Kwon et al. (1996), Lee and Jung (2001)

#### Deilephila elpenor

(Linnaeus, 1758)

C0020B03-989F-5778-88FA-9716F26033D0

##### Notes

Kim (1971), Lee and Kwon (1981), ME (1993), Kwon et al. (1996), Lee and Jung (2001), HNIBR (2022)

#### Macroglossum bombylans

(Boisduval, [1875])

F3581D6A-DD75-5807-969A-E50B766DD107

##### Notes

Kwon (2022)

#### Macroglossum pyrrhosticta

Butler, 1875

13C29FC8-E19A-5CDB-BC0F-8442CED83A32

##### Notes

Lee and Kwon (1981), ME (1993), Kwon et al. (1996), Lee and Jung (2001), Lim et al. (2013)

#### Macroglossum saga

(Butler, 1878)

9EDA3F54-57CF-57CF-B6E8-32DC9E0E3650

##### Notes

Lim et al. (2013)

#### Parum colligata

(Walker, 1856)

B63367E9-B92D-54F5-AEBD-8CF39E87A46E

##### Notes

Lee and Jung (2001), Lim et al. (2013)

#### Psilogramma increta

(Walker, 1865)

87129BD0-7B35-5240-B40F-9E878B95A105

##### Notes

Kwon et al. (1996), Lee and Jung (2001), Lim et al. (2013)

#### Theretra japonica

(Boisduval, 1867)

9FD909E6-89F2-527F-AD44-247F7C8FA996

##### Notes

Kwon et al. (1996), Lee and Jung (2001)

#### Tineidae

1E901543-FD49-5A3F-A1DD-4B5C8290E809

#### Triaxomasia orientanus

(Ponomarenko & Park, 1996)

06F997E8-A484-5ACF-956C-AD0E38B809DE

##### Notes

Lim et al. (2013), HNIBR (2022)

#### Tortricidae

D13C9FF8-5B9E-554C-9B02-D44044962E68

#### Acleris amurensis

(Caradja, 1928)

4EB33D7B-9ED6-5C8C-ADD9-6D2177942DAB

##### Notes

Lim et al. (2013)

#### Acleris laterana

(Fabricius, 1794)

2570ED7A-71FC-5B2E-AA3C-040B2615915F

##### Notes

Lim et al. (2013)

#### Adoxophyes orana

(Fischer von Röslerstamm, 1834)

7835F80C-B614-583E-9A98-517C29DB778F

##### Notes

Byun et al. (1996), Lim et al. (2013)

#### Antichlidas holocnista

Meyrick, 1931

EC4EB415-BA64-5E0B-8219-7D547FE34237

##### Notes

Lim et al. (2013)

#### Choristoneura longicellanus

(Walsingham, 1900)

5D27B937-BD94-5ABA-858C-6FCEC98D7B5D

##### Notes

Kim (1971), Lee and Kwon (1981), ME (1993), Byun et al. (1996), Kwon et al. (1996), Lee and Jung (2001), HNIBR (2022)

#### Clepsis rurinana

(Linnaeus, 1758)

546F2F78-46A5-58ED-A4ED-26A8BA26A68F

##### Notes

Byun et al. (1996), Lim et al. (2013)

#### Cryptaspasma marginifasciata

(Walsingham, 1900)

8ACEF6EC-82AE-574F-ADDE-B49BEB651BE7

##### Notes

Lim et al. (2013)

#### Epiblema foenella

(Linnaeus, 1758)

B005DB1C-DF30-5E65-8075-6B5803B772D3

##### Notes

Byun et al. (1996), Lim et al. (2013)

#### Eucosma glebana

(Snellen, 1883)

2249E8E6-BA2B-58D8-880E-665FFA9957BB

##### Notes

Byun et al. (1996), Lim et al. (2013)

#### Eudemis brevisetosa

Oku, 2005

DC30A478-9451-556A-B784-C29CC383AA2A

##### Materials

- **Type status:** Other material. **Occurrence:** recordedBy: Lee, D. Y.; sex: 1 male; lifeStage: adult; occurrenceID: 6F8EB0D0-2B19-5E5C-A802-F5E311804978; **Location:** island: Ulleungdo Island; country: Republic of Korea; stateProvince: Gyeongsangbuk-do; county: Ulleung-gun; locality: Taeharyeong, Namseo-ri, Seomyeon; **Event:** samplingProtocol: Light trap; eventDate: 6/22/2021

##### Notes

This is the first record of this species from Ulleungdo Island. However, Sohn et al. (2015) stated that the species in Korea previously known as *Eudemisprofundana* can be a complex of *Eudemisbrevistosa* and *Eudemislucina*, meaning *E.profundana* previously recorded in Ulleungdo Island by Lim et al. (2013) is possibly a misidentified species of *E.brevistosa* or *E.lucina*.

Distribution: Japan, Korea (Sohn et al. 2015)

#### Eudemis profundana

(Denis & Schiffermüller) 1775

F7C38B7F-6907-5CBB-BC25-F3C58C4A5030

##### Notes

Lim et al. (2013)

#### Eupoecilia ambiguella

(Hübner, 1796)

38D94DBC-62FA-57BB-B189-143CD837FBB8

##### Notes

Lim et al. (2013)

#### Grapholita delineana

Walker, 1863

744E849F-182F-5C9C-AA5F-5940C90C02B8

##### Notes

Lim et al. (2013)

#### Gravitarmata margarotana

(Heinemann, 1863)

A26590E1-6ADE-5381-B91A-7A9F15F71452

##### Notes

Kwon (2022)

#### Homona magnanima

Diakonoff, 1948

06949002-4CFD-598E-A0EA-6988E668732F

##### Notes

Byun et al. (1996), Lim et al. (2013)

#### Lobesia yasudai

Bae & Komai, 1991

F66A1BF8-F4F3-5F2F-85FB-D5994EB7615B

##### Notes

Lim et al. (2013), HNIBR (2022)

#### Pandemis monticolana

Yasuda, 1975

DE736300-468A-5CBA-9F8E-C78D0BFF3C16

##### Notes

Lim et al. (2013)

#### Rhopobota naevana

Hübner, [1814-1817]

FCB1E8E0-2776-5B7B-882C-555A46F14C9F

##### Notes

Lim et al. (2013)

#### Rhyacionia dativa

Heinrich, 1928

F9CFE054-4B3E-510E-8A9E-FF71B4ADDD26

##### Notes

Lim et al. (2013)

#### Yponomeutidae

7E729554-4744-5E36-BF08-40FAA3DB15A4

#### Yponomeuta evonymella

(Linnaeus, 1758)

ADD6DCBA-E3BE-5916-89A5-85DF4A62BC2A

##### Notes

Lim et al. (2013)

#### Zygaenidae

1A7E483E-5214-5A1B-804E-433F38DE3DE4

#### Illiberis dirce

(Leech, 1888[1889])

24BD006B-A9C8-50CE-88DF-1463509F25BC

##### Notes

Lim and Lee (2012), HNIBR (2022)

#### Illiberis rotundata

Jordan, [1907]

891245A0-1F49-5CF3-967B-257BD0178B2F

##### Notes

Lim et al. (2013)

#### Illiberis tenuis

(Butler, 1877)

D21B9ED4-E66F-5F16-80B6-51BC45A9249B

##### Notes

HNIBR (2022)

#### Mantodea

B13CE58E-7843-5213-9877-A1C542D68A0E

#### Mantidae

D1AFA0A0-8731-5B8E-8057-42AD2973710E

#### Hierodula patellifera

(Serville, 1839)

349375D2-79FF-5E80-B9F1-32D6B6E4560A

##### Notes

NSMK (2023b)

#### Statilia maculata

Thunberg, 1784

C5937F69-91F2-51D4-9939-79ABA86175D1

##### Notes

Lee and Jung (2001)

#### Tenodera angustipennis

Saussure, 1869

96CF55A3-DF9C-569E-A7E2-E75578AC962D

##### Notes

Cho (1955), Cho (1965), Lee and Kwon (1981), ME (1993), Kwon et al. (1996), Lee and Jung (2001), Lim and Lee (2012), Lim et al. (2013), Nam et al. (2018), HNIBR (2022)

#### Tenodera sinensis

Saussure, 1871

F32D7054-B82A-5C83-AC17-56F17C2573A7

##### Notes

Lim et al. (2013), Won et al. (2023)

#### Megaloptera

F7DF805F-A058-503C-828D-5C85DCE8C637

#### Corydalidae

83DA3DFB-9FDD-53AC-9BF4-5ACEF3AD31F3

#### Parachauliodes continentalis

Van der Weele, 1909

C3CF9EA9-1857-575C-9C1D-BB30DD75E9CB

##### Notes

Kwon (2021)

#### Neuroptera

5127324F-F515-5017-9E40-BCDCED1394DE

#### Chrysopidae

EEBC1077-B6C4-516F-BF90-36CD0B942ECB

#### Chrysopa intima

McLachlan, 1893

3B2BB60F-D512-52F3-824C-488517DBCE02

##### Notes

Lim et al. (2013)

#### Chrysopa pallens

(Rambur, 1838)

98DB9E93-5239-59F4-9ADC-A0C6D46D3D7B

##### Notes

Lee and Kwon (1981), ME (1993), Kwon et al. (1996), Lee and Jung (2001), Lim et al. (2013)

#### Chrysoperla furcifera

(Okamoto, 1914)

0C0A9B27-E1DC-5EF3-81C9-C14AF65D2E86

##### Notes

Kwon et al. (1996), Lee and Jung (2001)

#### Mantispidae

BF7A6F43-9077-5E61-9CD6-0C0F79FFAA84

#### Mantispa japonica

MacLachlan, 1875

170DE5BA-0A5A-5776-9712-9191BB7A2126

##### Notes

Info (2023)

#### Myrmeleontidae

A5C4A795-978F-5962-83E3-C0BCE399BBB9

#### Distoleon nigricans

(Okamoto, 1910)

AC332EA2-2B0C-5020-947D-64363FD32F54

##### Notes

Cho (1965), Lee and Kwon (1981), ME (1993), Kwon et al. (1996), Lee and Jung (2001)

#### Hagenomyia micans

(MacLachlan, 1875)

A3DB095C-30D3-5957-ADC0-7605AE9D8A34

##### Notes

Lim et al. (2013)

#### Paraglenurus japonicus

(McLachlan, 1867)

7D770831-9BD2-5D32-95AC-C409A0724E74

##### Notes

Lim et al. (2013)

#### Synclisis japonica

(Hagen, 1866)

03BFF332-D11C-5B1D-A7DA-C57811666A7B

##### Notes

Cho (1965), Lee and Kwon (1981), ME (1993), Kwon et al. (1996), Lee and Jung (2001)

#### Osmylidae

1AF17C2E-9AA3-5551-8C42-D2FEA85CC1A3

#### Lysmus harmandinus

(Navás, 1910)

B6E940A2-CC4D-5EE4-A463-734E3869B83D

##### Notes

Kwon (2022)

#### Odonata

9114AC93-1B25-546A-8CD5-74C674218110

#### Aeshnidae

D25F5EEF-29D2-50FE-A175-01154D224B40

#### Anax parthenope julius

Brauer, 1865

169613DC-7931-57B7-8BEC-1CEA30AFDEE7

##### Notes

Kim (1971), Lee and Kwon (1981), ME (1993), Kwon et al. (1996), Lee and Jung (2001), Lim and Lee (2012), Lim et al. (2013), HNIBR (2022)

#### Coenagrionidae

4F9F7B52-F23A-5CE0-B201-B9DED2B7D073

#### Coenagrion lanceolatum

(Selys, 1872)

26B745E2-E4A3-52F9-AC57-297FFAB82751

##### Notes

Lee and Kwon (1981), ME (1993), Kwon et al. (1996), Lee and Jung (2001)

#### Ischnura asiatica

(Brauer, 1865)

F111677B-7BFE-543A-B5A7-DD5A633EF4A2

##### Notes

Lim and Lee (2012), Kwon (2021), HNIBR (2022)

#### Mortonagrion selenion

(Ris, 1916)

55DD42D7-BDC9-54D4-8925-56A6AAF2CAEF

##### Notes

Info (2023)

#### Paracercion calamorum

(Ris, 1916)

129D7D2B-668C-5578-83BE-FBC2C60E59F8

##### Notes

Bae (2011)

#### Paracercion hieroglyphicum

(Brauer, 1865)

D0252566-BA9F-5421-A946-E3EE808DCCCA

##### Notes

Lim et al. (2013)

#### Gomphidae

6AD68CC8-2726-5764-9EE1-BB95F264CC81

#### Anisogomphus maacki

(Selys, 1872)

79B70740-75DD-5CCC-8193-E42C467ADC27

##### Notes

Kwon (2021)

#### Davidius lunatus

(Bartenev, 1914)

B55D9DDE-66F8-5F4F-AC9B-0FE40A037AEA

##### Notes

Kwon (2021)

#### Sieboldius albardae

Selys, 1886

85223AE0-CDC3-5D5D-AFAA-D357420E1DC5

##### Notes

Kwon (2021)

#### Libellulidae

81DD3B3C-A0D9-561E-B132-18DB30D87E45

#### Crocothemis servilia mariannae

Kiauta, 1898

08FEA4D1-F3D4-5BBE-98FA-74F99D525DA7

##### Notes

Cho (1965), Lee and Kwon (1981), ME (1993), Kwon et al. (1996), Lee and Jung (2001)

#### Orthetrum albistylum

(Selys, 1848)

5B46B5AD-6BB4-5741-AB73-AEAAA9885A73

##### Notes

Cho (1955), Cho (1965), Lee and Kwon (1981), ME (1993), Kwon et al. (1996), Lee and Jung (2001), Lim and Lee (2012), HNIBR (2022)

#### Orthetrum triangulare

(Selys, 1878)

9EE7C363-ED15-5F1F-99EA-A58F54BCB1B4

##### Notes

Lee and Jung (2001)

#### Pantala flavescens

(Fabricius, 1798)

4B9AE8D6-CA5F-54FC-A91E-59EFC2BF7699

##### Notes

Kim (1971), Lee and Kwon (1981), ME (1993), Kwon et al. (1996), Lee and Jung (2001), Lim et al. (2013), HNIBR (2022)

#### Sympetrum darwinianum

(Selys, 1883)

74A7EC35-4DFD-5851-9E94-5157F8B42FAF

##### Notes

Kim (1971), Lee and Kwon (1981), ME (1993), Kwon et al. (1996), Lee and Jung (2001), HNIBR (2022)

#### Sympetrum frequens

(Selys, 1883)

691BA16C-EA9E-5727-A0D1-FF5AFFAA547A

##### Notes

Lim et al. (2013)

#### Sympetrum infuscatum

(Selys, 1883)

70EB6AE0-4491-5C49-8A91-EC4DCAE83E7E

##### Notes

Lim et al. (2013)

#### Orthoptera

3B21D670-32AA-5E23-82C0-4900DB242B5B

#### Acrididae

30C44A3A-A8D8-5AAF-A58F-0A888DE5F527

#### Acrida cinerea

(Thunberg, 1815)

579FCA45-C9E3-51DC-943F-A71D48C1B7B5

##### Notes

Cho (1955), Cho (1965), Lee and Kwon (1981), ME (1993), Kwon et al. (1996), Lee and Jung (2001), Lim et al. (2013), HNIBR (2022)

#### Chorthippus maritimus maritimus

Mistshenko, 1951

2EC644B7-CEC0-5DE5-98B3-B2A709860AFC

##### Notes

Info (2023)

#### Gastrimargus marmoratus

(Thunberg, 1815)

2842D0D5-CE43-596B-A4A1-60D7D1F17F32

##### Notes

Cho (1955), Cho (1965), Lee and Kwon (1981), ME (1993), Kwon et al. (1996), Lee and Jung (2001)

#### Gonista bicolor

(Haan, 1842)

8D9692BB-DE70-549B-BF10-8F05B98274FD

##### Notes

Lee and Kwon (1981), ME (1993), Kwon et al. (1996), Lee and Jung (2001)

#### Locusta migratoria migratoria

Linnaeus, 1758

46814505-1209-53E4-B4EE-EB9FD830BFC3

##### Notes

Cho (1955), Cho (1965), Lee and Kwon (1981), ME (1993), Kwon et al. (1996), Lee and Jung (2001), Lim et al. (2013), HNIBR (2022)

#### Oedaleus infernalis

Saussure, 1884

A63619E1-3B4A-5D34-B800-0FED8736A712

##### Notes

Cho (1955), Cho (1965), Lee and Kwon (1981), ME (1993), Kwon et al. (1996), Lee and Jung (2001), Lim et al. (2013)

#### Oxya sinuosa

Mishchenko, 1951

81EC95D7-02A0-55E8-B015-CB887733EE15

##### Notes

Lee and Kwon (1981), ME (1993), Kwon et al. (1996), Lee and Jung (2001)

#### Podismopsis genicularibus

(Shiraki, 1910)

C68DF7B9-6FB2-554A-B8F6-EE40618F1655

##### Notes

HNIBR (2022)

#### Shirakiacris shirakii

(Bolívar, 1914)

BDFB62A2-6817-57FB-BD7B-D9096E11B72F

##### Notes

Cho (1965), Lee and Kwon (1981), ME (1993), Kwon et al. (1996), Lee and Jung (2001), Lim et al. (2013), HNIBR (2022), Won et al. (2023)

#### Trilophidia annulata

(Thunberg, 1815)

A5F57D55-E36F-531B-94F8-BEE6AF11990A

##### Notes

Kim (1971), Lee and Kwon (1981), ME (1993), Kwon et al. (1996), Lee and Jung (2001), HNIBR (2022), Won et al. (2023)

#### Gryllidae

63AD202A-01F3-5AD2-9774-C977B4C7FE9E

#### Oecanthus euryelytra

Ichikawa, 2001

8809E4FE-94CF-59F0-ABB6-B616D76170CF

##### Notes

NSMK (2023b)

#### Oecanthus longicauda

Matsumura, 1904

C800013D-9E6B-57FE-B13C-D92E5E5557D8

##### Notes

Lim et al. (2013), HNIBR (2022), Won et al. (2023)

#### Teleogryllus emma

(Ohmachi & Matsuura, 1951)

EAFEF6E5-FA05-54B2-8936-55979898258E

##### Notes

Cho (1965), Won et al. (2023)

#### Velarifictorus aspersus

(Walker, 1869)

43D35FFE-0AC4-5FE3-A55A-B2C295DF23F9

##### Notes

Lee and Jung (2001)

#### Velarifictorus micado

(Saussure, 1877)

9790A116-1EBE-57C0-8AE7-EE3F1C779BAE

##### Notes

Lim et al. (2013)

#### Pyrgomorphidae

99FC5A7E-A00E-5437-8E1B-87A6286890F0

#### Atractomorpha lata

(Motschulsky, 1866)

7D656145-E1F3-5C37-A832-49E8D3D9DC4C

##### Notes

Lee and Kwon (1981), ME (1993), Kwon et al. (1996), Lee and Jung (2001)

#### Rhaphidophoridae

99B54C50-631F-5EB3-9B02-6D9D5C028675

#### Paratachycines ussuriensis

Storozhenko, 1990

ADCB2BC1-E80F-59D3-9BAB-830E300475B5

##### Notes

Lim and Lee (2012), HNIBR (2022), Won et al. (2023)

#### Tachycines asynamorus

Adelung, 1902

F68060D4-EB0A-5872-89B3-329F0F399752

##### Notes

Cho (1955), Cho (1965), Lee and Kwon (1981), ME (1993), Kwon et al. (1996), Lee and Jung (2001)

#### Tachycines coreanus

Yamasaki, 1969

BFBE4AF8-A3F4-5C9B-B387-EFA67D85DC45

##### Notes

Cho (1955), Cho (1965), Lee and Kwon (1981), ME (1993), Kwon et al. (1996), Lee and Jung (2001), Lim and Lee (2012), Lim et al. (2013), Nam et al. (2018), HNIBR (2022), Won et al. (2023)

#### Tetrigidae

04B8A508-04FE-5FA1-8F67-4C0BCE1BC57B

#### Euparatettix insularis

Bey-Bienko, 1951

4DDA465C-AA9A-537C-BDB6-46486BB0D010

##### Notes

Lee and Kwon (1981), ME (1993), Kwon et al. (1996), Lee and Jung (2001)

#### Tetrix japonica

(Bolívar, 1887)

F79CA9E0-BF6D-5B16-8553-7827156140C3

##### Notes

Lee and Kwon (1981), ME (1993), Lee and Jung (2001), Lim and Lee (2012), HNIBR (2022), Won et al. (2023)

#### Tettigoniidae

9A4D6AC1-94C4-5903-8186-3BCCF360941C

#### Chizuella bonneti

(Bolívar, 1890)

24E5859C-07E7-5099-9F10-4E954D442FB9

##### Notes

Info (2023)

#### Conocephalus japonicus japonicus

(Redtenbacher, 1891)

939C898A-3D52-5178-B938-02199EECC459

##### Notes

Kwon et al. (1996), Lee and Jung (2001)

#### Conocephalus maculatus

(Le Guillou, 1841)

0A982DF5-274B-5ABF-A921-B7D745389F31

##### Notes

Info (2023)

#### Cosmetura fenestrata

Yamasaki, 1983

CB49D2E7-FA53-5CF2-886F-E557527732D4

##### Notes

Kwon (2022)

#### Ducetia japonica

(Thunberg, 1815)

B29859B0-A632-5D06-B17D-91451C0F3186

##### Notes

Cho (1955), Cho (1965), Lee and Kwon (1981), ME (1993), Kwon et al. (1996), Lee and Jung (2001), Lim et al. (2013), Won et al. (2023)

#### Elimaea fallax

Bey-Bienko, 1951

8B4FD4AA-76D5-5BB0-A529-9F7BE26A664D

##### Notes

Lim et al. (2013)

#### Hexacentrus japonicus

Karny, 1907

6AF2873C-5F0C-5234-827D-CFA8AF1247F2

##### Notes

Lim et al. (2013), Won et al. (2023)

#### Phaneroptera falcata

(Poda, 1761)

0E16932C-21D8-5F21-8D31-228812758805

##### Notes

Lee and Kwon (1981), ME (1993), Kwon et al. (1996), Lee and Jung (2001), Lim et al. (2013), Won et al. (2023)

#### Phaneroptera nigroantennata

Brunner von Wattenwyl, 1878

F9BDF7F5-578A-528D-A7AA-FF77F4E06994

##### Notes

Kwon et al. (1996), Lee and Jung (2001), Lim et al. (2013), Won et al. (2023)

#### Ruspolia dubia

(Redtenbacher, 1891)

96E6CDC1-7CD7-521B-B679-777F02A3B1A3

##### Notes

Lee and Kwon (1981), ME (1993), Lee and Jung (2001)

#### Ruspolia lineosa

(Walker, 1869)

CA9BF93C-43B1-5CF5-A63F-949E2D3AE2F4

##### Notes

Kwon et al. (1996), Lee and Jung (2001), Lim et al. (2013)

#### Sinochlora longifissa

(Matsumura & Shiraki, 1908)

A0A61D79-6B29-5AA8-970A-5EF16D6910DD

##### Notes

Lim et al. (2013)

#### Tridactylidae

F27C3F6F-00D4-5095-9863-01A310BD5B55

#### Xya japonica

(De Haan, 1842)

C96A5571-8920-583B-A006-807FFDD831C2

##### Notes

Kwon (2021)

#### Trigonidiidae

2B93FA39-8BCC-503C-953D-F4BC7994082A

#### Metioche japonica

(Ichikawa, 2001)

3A8D072B-6DCA-5B82-8333-220072640A9C

##### Notes

Nam et al. (2018)

#### Polionemobius flavoantennalis

(Shiraki, 1911)

BC03EC11-C2F0-58C2-8397-2A01464E2147

##### Notes

Kwon et al. (1996), Lee and Jung (2001)

#### Svistella bifasciata

(Shiraki, 1911)

DB484B7B-D0E8-5B70-8559-AC057238C8CD

##### Notes

Choi (1996), Kwon et al. (1996), Lee and Jung (2001)

#### Phasmida

11293ADA-C10D-5090-B622-3B621710EF62

#### Lonchodidae

ED304A02-3F35-563A-85ED-D8FFC8139F2D

#### Phraortes elongatus

(Thunberg, 1815)

EDCF694B-0B42-5BD0-91A2-999E18F3FE42

##### Notes

Lim et al. (2013)

#### Plecoptera

FD87A0B2-63D5-57BD-A1B8-8FF6470B6306

#### Chloroperlidae

13420B0D-4278-5971-8CCE-33F40CB15F0F

#### Sweltsa nikkoensis

(Okamoto, 1912)

2A0012E0-2813-583A-B1E4-2279C3343112

##### Notes

Kwon (2021)

#### Nemouridae

B9B8128E-CC8E-55FE-9BB3-A009AC1232BD

#### Amphinemura coreana

Zwick, 1973

D5BC444C-13E5-5925-9B15-529C235F8036

##### Notes

Kwon (2021)

#### Nemoura tau

Zwick, 1973

36FBECCF-4C57-54E1-9A14-A7331A9F5B20

##### Notes

Kwon (2021)

#### Perlidae

E3992116-607F-5E5E-ABB6-EA311676B2DA

#### Neoperla coreensis

Ra, Kim, Kang & Ham, 1994

7A1509A2-23C8-5BC9-9827-3645B693D167

##### Notes

Kwon (2021)

#### Paragnetina flavotincta

(McLachlan, 1872)

FC1690D1-C8BD-51BF-86E5-CFDFDCC3AECF

##### Notes

Kwon (2021)

#### Pteronarcyidae

48C264AF-6BBC-56B7-AE76-141C16DF1F08

#### Pteronarcys macra

Ra, Baik & Cho, 1991

5287F361-88BD-5A34-8CF4-0C2B62AEE950

##### Notes

Kwon (2021)

#### Psocodea

AEA8CA96-CCA5-5E1F-9F62-AAF9BC8BB9BE

#### Pediculidae

B4C2F0E0-F9E7-5665-A10B-E31E39D4DBA3

#### Pediculus humanus

Linnaeus, 1758

7C09F1F4-A949-56A8-A5EB-DCE0F8F05353

##### Notes

Cho (1955), Cho (1965), Lee and Kwon (1981), ME (1993), Kwon et al. (1996), Lee and Jung (2001)

#### Siphonaptera

A4F99E38-4A35-5BE8-96C6-200A3DBF3AAF

#### Pulicidae

0EE40359-F898-562A-B697-451D553A7CDB

#### Pulex irritans

Linnaeus, 1758

04AD757B-22C6-538B-9A01-E900641328CE

##### Notes

Cho (1955), Cho (1965), Lee and Kwon (1981), ME (1993), Kwon et al. (1996), Lee and Jung (2001)

#### Thysanoptera

80856768-BEDF-52B3-A187-55EBB0659E3F

#### Phlaeothripidae

8FDB3604-0A9D-5748-84E4-D2FEEAF90BDC

#### Haplothrips aculeatus

(Fabricius, 1803)

5F6B1C08-7876-5530-8785-BC72CDE62E53

##### Notes

Lee and Kwon (1981), ME (1993), Kwon et al. (1996), Lee and Jung (2001)

#### Haplothrips chinensis

Priesner, 1933

28F394C9-ABBA-59DF-9B4C-A63CDEAF2F6C

##### Notes

Lee and Kwon (1981), ME (1993), Kwon et al. (1996), Lee and Jung (2001)

#### Haplothrips leucanthemi

(Schrank, 1781)

F00B6537-AA1F-5090-9354-BD189D4C722A

##### Notes

Lee and Kwon (1981), ME (1993), Kwon et al. (1996), Lee and Jung (2001)

#### Liothrips vaneeckei

Priesner, 1920

B9A73E00-923C-5CD3-B185-8D5EFBED9BDD

##### Notes

Nam et al. (2018)

#### Thripidae

9719C586-F4F7-5CAC-AC5D-609AC8E599A2

#### Chirothrips manicatus

(Haliday, 1836)

461AFF3A-D1CF-52AE-9F19-F52A6F613D38

##### Notes

Lee and Kwon (1981), ME (1993), Kwon et al. (1996), Lee and Jung (2001)

#### Frankliniella intonsa

(Trybom, 1895)

47E570FA-12D0-56CD-8545-E90C4401C3ED

##### Notes

Lee and Kwon (1981), ME (1993), Kwon et al. (1996), Lee and Jung (2001)

#### Megalurothrips distalis

(Karny, 1913)

4330EADC-EF4C-5AD5-811C-D748B32B19FC

##### Notes

Lee and Kwon (1981), ME (1993), Kwon et al. (1996), Lee and Jung (2001)

#### Mycterothrips glycines

(Okamoto, 1911)

738492DE-FD04-5C94-BC31-1244D913F8CA

##### Notes

Lee and Kwon (1981), ME (1993), Kwon et al. (1996), Lee and Jung (2001)

#### Scirtothrips dorsalis

Hood, 1919

DEB42AD0-B495-525C-9C93-5E3042515B96

##### Notes

Nam et al. (2018)

#### Taeniothrips oreophilus

Priesner, 1935

24641DCB-5BB4-5C00-B047-FE17987E21DE

##### Notes

Lee and Kwon (1981), ME (1993), Kwon et al. (1996), Lee and Jung (2001)

#### Taeniothrips pallipes

Bagnall

0E731C42-E5A6-5B4A-9FBB-E12AD4312F24

##### Notes

Lee and Kwon (1981), ME (1993), Kwon et al. (1996), Lee and Jung (2001)

#### Thrips flavidulus

(Bagnall, 1923)

4E9844F7-CF6D-5EB3-8086-D34B10108488

##### Notes

Lee and Kwon (1981), ME (1993), Kwon et al. (1996), Lee and Jung (2001)

#### Thrips hawaiiensis

(Morgan, 1913)

6F580560-9678-515B-A794-F9EB87B9D22A

##### Notes

Lee and Kwon (1981), ME (1993), Kwon et al. (1996), Lee and Jung (2001)

#### Trichromothrips xanthius

(Williams, 1917)

226131CF-70E6-5970-BCAB-E51DBA3020AC

##### Notes

Lee and Kwon (1981), ME (1993), Kwon et al. (1996), Lee and Jung (2001)

#### Trichoptera

7B1AA528-87B8-511B-9BB6-BB6E8BB11CCB

#### Apataniidae

A788C450-4C7F-50EB-9638-4ACE2F61D47F

#### Apatania maritima

Ivanov & Levanidova, 1993

8E68AE5C-23DD-5C8C-96CD-CF966E057CED

##### Notes

NIBR (2021)

#### Apatania sinensis

(Martynov, 1914)

941CF626-BE2B-5C7E-AB35-E20DBB3A24CC

##### Notes

Lim and Lee (2012), Info and Park (2023)

#### Goeridae

59C2B1E3-558B-5A51-8E34-FBF5213F924F

#### Goera japonica

Banks, 1906

C516EFDB-DB64-55D4-A040-EA843458693A

##### Notes

NIBR (2021)

#### Hydrobiosidae

75A30965-85F4-5CB1-A8B8-40D9E6003975

#### Apsilochorema sutshanum

Martynov, 1934

41E27E77-EA8E-5CA0-9C72-268D5F007EEE

##### Notes

NIBR (2021)

#### Hydropsychidae

F74B3288-D588-54B8-9A56-AF58A40B1FA7

#### Cheumatopsyche brevilineata

(Iwata, 1927)

89D6752A-16F7-58A5-92E5-E4C5C80690C8

##### Notes

Kwon (2021)

#### Hydropsyche kozhantschikovi

Martynov, 1924

09FA3BFB-3644-508F-BA6B-FD89CE906F2F

##### Notes

NIBR (2021)

#### Hydropsyche orientalis

Martynov, 1934

F6D6FB53-3738-5AA9-8AB3-CEF8D2DFE42E

##### Notes

NIBR (2021)

#### Lepidostomatridae

7285264C-0779-5291-8168-B4BFFB311D43

#### Lepidostoma orientale

(Tsuda, 1942)

79FF52E5-2F01-55CF-B475-96BFE0D082BE

##### Notes

Lim and Lee (2012)

#### Odontoceridae

630AF039-ED2E-5BD9-A5B3-1039B97C7CC1

#### Psilotreta kisoensis

Iwata, 1928

55B7161B-613A-5B01-9271-70AD7AAD2027

##### Notes

Kwon (2021)

#### Phryganopsychidae

56EE8C97-81AE-5E0E-AA29-C1C2A120B96C

#### Phryganopsyche latipennis

(Banks, 1906)

25CB97FE-5B02-5073-B686-3606D0997FCA

##### Notes

Lim and Lee (2012), Kwon (2021), HNIBR (2022)

#### Rhyacophilidae

C92B2369-CAD3-509A-8A81-8FA2E8C0EFCE

#### Rhyacophila bilobata

Ulmer, 1907

86080A6D-6376-5BD1-8E08-4F072384B48A

##### Notes

Kwon (2021)

#### Rhyacophila brevicephala

Iwata, 1927

02984CD3-FC9B-51F1-9789-F1414C0B7A92

##### Notes

Info and Park (2023)

#### Rhyacophila kuramana

Tsuda, 1942

6DF9F9B6-8AC2-5E0F-A8E0-04DAFEA020FC

##### Notes

NIBR (2021)

#### Rhyacophila lata

Martynov, 1918

563000B5-6FB9-5D48-B1C5-81FA70D58E05

##### Notes

NIBR (2021)

#### Rhyacophila mroczkowskii

Botosaneanu, 1970

A79D7EB7-34B9-51AA-A77E-4189201861E2

##### Notes

Lim and Lee (2012), HNIBR (2022)

#### Rhyacophila nigrocephala

Iwata, 1927

33C9A84C-A60B-5336-AC36-CB6F1CC6A117

##### Notes

Info and Park (2023)

#### Rhyacophila retracta

Martynov, 1914

457608DB-09ED-5B8D-8484-9E12F9928FCB

##### Notes

Kwon (2021)

#### Rhyacophila shikotsuensis

Iwata, 1927

AD472AFB-F26F-51E3-B0B3-7027150CB877

##### Notes

Info and Park (2023)

#### Zygentoma

936E1D63-EF2C-5DB5-89BD-2F4ABDE588BE

#### Lepismatidae

C39D794C-9067-5539-BD32-103DFDB7DC89

#### Ctenolepisma longicaudata

Escherich, 1905

62544257-AEA8-5C3D-8C08-B8D5507A3B7F

##### Notes

Cho (1965), Lee and Kwon (1981), ME (1993), Kwon et al. (1996), Lee and Jung (2001)

### Insect Checklist of Dokdo Island

#### Blattodea

5FA4E1E9-0658-592D-8EBD-57ADD6C22551

#### Ectobiidae

30D5DAAA-9C7C-5B43-8E24-829E2E3F90AE

#### Blattella nipponica

Asahina, 1963

D165A404-3E3B-599B-A2AB-FD985C1D9877

##### Notes

Kang et al. (2013)

#### Coleoptera

0AAF8646-671B-5563-8DD0-4132E416C808

#### Carabidae

E63C042C-9B1B-509A-ADC0-A6A5CD31173F

#### Anisodactylus signatus

(Panzer, 1796)

2ABE1A6B-F939-5BCD-AAD6-595A1AC1A847

##### Notes

Lee and Kwon (1981), Kwon et al. (1996), Lee and Jung (2001), Kim (2004), Lee et al. (2006), Kim and Yeom (2006), Kim et al. (2009), Lee et al. (2009)

#### Anisodactylus tricuspidatus

Morawitz, 1863

DB7FC298-0E3C-52C9-B5F9-87E1D8595DD8

##### Notes

Lee and Jung (2001), Lee et al. (2006), Kim and Yeom (2006), Lee et al. (2009), Park et al. (2010), Park et al. (2011)

#### Dolichus halensis halensis

(Schaller, 1783)

C412418C-75B2-5419-B52B-C0B555B3D266

##### Notes

Lee and Kwon (1981), Kwon et al. (1996), Lee and Jung (2001), Kim (2004), Lee et al. (2006), Lee et al. (2009), Kim and Yeom (2006), Kim et al. (2009), Park et al. (2011)

#### Harpalus jureceki

(Jedlicka, 1928)

6BDE3BBD-9FBD-5089-8321-5770284571BC

##### Notes

Lee et al. (2009)

#### Harpalus sinicus

Hope, 1845

4A2033BE-57F8-58DD-9D1E-6C898B02324E

##### Notes

Park et al. (2011), Park et al. (2013)

#### Stenolophus difficilis

(Hope, 1845)

D4A60D4F-6AE0-5DBE-BDFA-5A5FC04BF8CF

##### Notes

Park et al. (2012)

#### Cerambycidae

2AF84867-65FA-51EB-9ACA-BFB6200145F5

#### Arhopaloscelis bifasciata

(Kraatz, 1879)

28862464-2D5A-59CE-9337-9A405D89C9BB

##### Materials

- **Type status:** Other material. **Occurrence:** recordedBy: Lee, D. Y.; sex: 1 male; lifeStage: adult; occurrenceID: C0CF4E44-DA97-5563-88D0-DF4BFD6AFE6E; **Location:** island: Dokdo Island; country: Republic of Korea; stateProvince: Gyeongsangbuk-do; county: Ulleung-gun; locality: Seodo of Dokdo, Dokdo-ri, Ulleungeup; **Event:** samplingProtocol: sweeping net; eventDate: 2015-06-23

##### Notes

This is the first record of this species from Dokdo Island.

Distribution: China, Russia (GBIF.org 2023d), Korea (National Institute of Biological Resources 2019)

#### Chrysomelidae

AD610636-ED58-5DA2-9E1A-E8F925A2F68A

#### Callosobruchus chinensis

(Linnaeus, 1758)

A7FC3013-81F1-5110-97AF-BE8CF876CDAA

##### Notes

Lee and Jung (2001), Lee et al. (2006), Kim and Yeom (2006)

#### Cassida nebulosa

Linnaeus, 1758

068F1A6D-4AE5-5ED1-B4CC-EE7D1A618733

##### Notes

Lee and Jung (2001), Lee et al. (2006), Kim and Yeom (2006)

#### Cassida piperata

Hope, 1842

B6C970DA-0F16-5157-A568-F82727F140B7

##### Notes

Lee and Kwon (1981), Kwon et al. (1996), Lee and Jung (2001), Kim (2004), Lee et al. (2006), Kim and Yeom (2006), Kim et al. (2009), Lee et al. (2009), Park et al. (2010), Park et al. (2011), Park et al. (2012), Park et al. (2013), Choi (2015), Lee (2016), Choi (2018), Ryu et al. (2021)

#### Longitarsus succineus

(Foudras, 186)

B514ABBA-70B6-50F6-9E0D-4051DCD81F18

##### Notes

Lee and Kwon (1981), Kwon et al. (1996), Lee and Jung (2001), Kim (2004), Lee et al. (2006), Kim and Yeom (2006)

#### Psylliodes punctifrons

Baly, 1874

24D55318-DE7D-5731-9773-5A18FDEDE3B1

##### Notes

An (2008), Park et al. (2010), Park et al. (2013), Park and Jang (2016), Choi (2018)

#### Thlaspida biramosa

(Boheman, 1855)

81C0DED9-2661-53B1-9748-3984ABBA2FE4

##### Notes

Choi (2017)

#### Coccinellidae

9DC19BEF-116E-5AE5-9D35-20D1F21E2DEB

#### Coccinella septempunctata

Linnaeus, 1758

9A0EEC81-415F-5DF2-9F27-9B0F6C10404A

##### Notes

Kwon et al. (1996), Lee and Jung (2001), Kim (2004), Kim and Yeom (2006), Lee et al. (2006), Lee et al. (2009), Choi (2017)

#### Harmonia axyridis

(Pallas, 1773)

1B0EA1BA-315C-5609-A6D3-AD5BCFCF731D

##### Notes

Lee and Jung (2001), Lee et al. (2006), Kim and Yeom (2006), Lee et al. (2009)

#### Harmonia yedoensis

(Takizawa, 1917)

DC5E773B-ED34-5A4A-BA3D-E23DD7DC3982

##### Notes

Park and Jang (2016)

#### Propylea japonica

(Thunberg, 1781)

998777B2-1039-5307-B6DB-ADD129F3AA25

##### Notes

Kwon et al. (1996), Lee and Jung (2001), Kim (2004), Kim and Yeom (2006), An (2008), Kim et al. (2009), Lee et al. (2009), Park et al. (2010), Choi (2018)

#### Scymnus babai

Sasaji, 1971

DA04B29D-54A1-5551-889C-621FD167806B

##### Notes

An (2008), Park et al. (2010), Choi (2018)

#### Scymnus ferrugatus

(Moll, 1785)

80B9F362-A5A7-5F8A-9596-DB2EFAAC4613

##### Notes

Choi (2015), Park and Jang (2016), Lee (2016)

#### Curculionidae

B02B0FB0-C00F-543B-9446-1E1074AA4E78

#### Ceutorhynchus albosuturalis

(Roelofs, 1875)

A0CA46F0-1BAA-55A0-988F-E41DCAFFA8CA

##### Notes

Lee et al. (2006), An (2008), Lee et al. (2009), Park et al. (2010), Park et al. (2013), Ryu et al. (2021)

#### Cosmobaris scolopacea

(Germar, 1819)

8431311E-19C7-50B7-9BF2-5954148C74FB

##### Notes

Kwon et al. (1996), Lee and Jung (2001), Kim (2004), Lee et al. (2006), Kim and Yeom (2006), An (2008), Lee et al. (2009), Park et al. (2012), Park and Jang (2016)

#### Rhinoncus cribricollis

Hustache, 1916

569FE122-9625-5F2F-885D-8C475DA7854A

##### Notes

Kwon et al. (1996), Lee and Jung (2001), Kim (2004), Kim and Yeom (2006), Park et al. (2010), Choi (2018)

#### Rhinoncus jakovlevi

Faust, 1893

4F75FB8F-D1C3-5A78-9F97-DDDEBC29FC0B

##### Notes

Park and Jang (2016)

#### Scepticus insularis

Roelofs, 1873

43461529-4C41-5B09-8583-30F320AEFCF1

##### Notes

An (2008)

#### Scepticus uniformis

Kôno, 1930

0A407361-EBF5-5FCB-B5B3-91EF0F6AF248

##### Notes

Choi (2015), Park and Jang (2016)

#### Simulatacalles simulator

(Roelofs, 1875)

CAE74E89-3221-542D-9D1C-DC3B765007F4

##### Notes

Hwang (2023)

#### Sitona lineatus

(Linnaeus, 1758)

178878FA-C624-5A93-AD67-94E8899E1D01

##### Notes

Lee and Jung (2001), Kim and Yeom (2006), An (2008), Kim et al. (2009), Park et al. (2011)

#### Dermestidae

01570DFD-59E8-5AF6-B04F-AE87E5FD2733

#### Dermestes tessellatocollis tessellatocollis

Motschulsky, 1860

AAB02E43-7282-54CC-BCB3-96CE52C9C5CA

##### Notes

Kwon et al. (1996), Lee and Jung (2001), Kim (2004), Kim and Yeom (2006), An (2008), Kim et al. (2009), Park et al. (2011), Park et al. (2012), Park and Jang (2016), Choi (2018)

#### Elateridae

FFB1A328-18FA-5807-A9C6-DE0C36C642F7

#### Agrypnus miyamotoi miyamotoi

(Nakane & Kishii, 1955)

B36EAEA0-D9B9-50CF-9504-4B101BEA3AA9

##### Notes

Lee and Kwon (1981), Kwon et al. (1996), Lee and Jung (2001), Kim (2004), Lee et al. (2006), Kim and Yeom (2006), Kim et al. (2009), An (2008), Lee et al. (2009), Park et al. (2011), Park et al. (2012), Park et al. (2013), Choi (2015), Park and Jang (2016)

#### Melanotus matsumurai

Schenkling, 1927

A29A2CB1-FC8E-54E5-A1BB-4E767AAB73F7

##### Notes

Lee et al. (2009), Park et al. (2011)

#### Melanotus cete cete

Candeze, 1860

FD16A7B7-FAEF-55F0-BB59-F708CB10DA87

##### Notes

Choi (2015), Park and Jang (2016), Lee (2016)

#### Pectocera fortunei

Candeze, 1873

FA5C3E7F-D63C-50EC-B789-FB03829958E3

##### Notes

Choi (2017)

#### Endomychidae

A90264D1-B7C9-50E4-A0EA-A376D6E68261

#### Ancylopus melanocephalus

A.G.Olivier, 1808

ADEAB21B-87B8-5EB2-9FBF-EAE16B83FA03

##### Notes

Lee and Jung (2001), Lee et al. (2006), Kim and Yeom (2006)

#### Ancylopus pictus asiaticus

(Strohecker, 1972)

FE24AEC1-A803-5784-A03B-3A27F8D964B2

##### Notes

Lee and Kwon (1981), Kwon et al. (1996), Lee and Jung (2001), Kim (2004), Lee et al. (2006), Kim and Yeom (2006)

#### Holoparamecus contractus

Wollaston, 1874

FFCC6E89-4DE2-5264-8F8A-B49424B4F498

##### Notes

Hwang (2023)

#### Hydrophilidae

99AF8B01-0CEC-5BCA-B372-129E836321F5

#### Hydrophilus acuminatus

Motschulsky, 1854

B9057B79-734A-51F4-B132-443C31A8B496

##### Notes

Choi (2015)

#### Latridiidae

3F91500B-8372-561B-A5C6-357395A638B9

#### Cortinicara gibbosa

(Herbst, 1793)

637007DA-9449-542D-A6F9-019A0E9D63E2

##### Notes

Kwon et al. (1996), Lee and Jung (2001), Kim (2004), Lee et al. (2006), Kim and Yeom (2006), An (2008), Lee et al. (2009), Park et al. (2011), Park et al. (2012), Park et al. (2013), Park and Jang (2016), Choi (2018)

#### Stephostethus chinensis

(Reitter, 1877)

B9B8B169-FF8A-5D5C-9B2E-71C2D2D6258B

##### Notes

Lee et al. (2009), Park et al. (2011), Park et al. (2012), Park et al. (2013)

#### Mordellidae

3A9BFB6D-51CF-52A6-A507-875BD447130A

#### Mordella tokejii

Nomura, 1958

06DE3E2D-46A2-5C76-A811-C41479E3CC05

##### Notes

Kim (2004), Lee et al. (2006), Kim and Yeom (2006), An (2008)

#### Nitidulidae

D43E9C3E-6BCD-5FE6-B70E-EFC5B7DE8C7A

#### Omosita colon

(Linnaeus, 1758)

05B9AF7F-D729-576E-9F26-A4B98061F683

##### Notes

Kwon et al. (1996), Lee and Jung (2001), Kim (2004), Lee et al. (2006), Kim and Yeom (2006), An (2008), Park et al. (2010), Park et al. (2012), Park and Jang (2016)

#### Omosita japonica

Reitter, 1874

57927576-0E8C-52FA-9DCE-EC1B694B49BA

##### Notes

An (2008)

#### Oedemeridae

05CA031F-486E-5438-9253-CC0D04F2C52D

#### Nacerdes melanura

(Linnaeus, 1758)

9D27E2AC-D26D-514B-9B28-EAB2AAA05975

##### Notes

Choi (2018)

#### Scarabaeidae

7E4B93C3-D7F1-56CA-AA62-3600FD3D2ABC

#### Aphodius urostigma

Harold, 1862

B2420782-8C33-547E-B245-5B12EED3D521

##### Notes

Ryu et al. (2021)

#### Staphylinidae

8BD20AAF-1447-5710-BFE8-8CCF0236B76C

#### Aleochara fucicola

Sharp, 1874

CEACB820-E13A-50FC-8D67-A8A909542108

##### Notes

Yoo et al. (2013)

#### Atheta tokiokai

(Sawada, 1971)

9783CE27-CCA4-537C-A300-D09F7F8C958B

##### Notes

Yoo et al. (2013)

#### Cafius histrio

(Sharp, 1874)

F2AA6E9B-D78E-5D5B-ACC4-9FA474B9C769

##### Notes

Yoo et al. (2013)

#### Hydrobius fuscipes

(Linnaeus, 1758)

BC659FFC-8743-52BE-896A-5B91FB852F3D

##### Notes

Park and Jang (2016)

#### Tenebrionidae

709DA5EF-32D1-5C8A-BF2F-68DB47CCACD8

#### Gonocephalum coenosum

Kaszab, 1952

CF8DE720-6A96-5E34-A20E-F06E71CA1B60

##### Notes

Kwon et al. (1996), Lee and Jung (2001), Kim (2004), Lee et al. (2006), Kim and Yeom (2006), An (2008), Park et al. (2010), Park et al. (2011), Park et al. (2013), Choi (2015), Park and Jang (2016), Choi (2018)

#### Gonocephalum coriaceum

(Motschulsky, 1858)

101DAEE9-96CE-542F-AA8F-CD832329B09F

##### Notes

Yoon (1978), Lee and Kwon (1981), Kwon et al. (1996), Lee and Jung (2001), Kim (2004), Lee et al. (2006), Kim and Yeom (2006), Kim et al. (2009)

#### Dermaptera

3CDC6B69-077C-5735-AB5B-938C90DA104B

#### Anisolabididae

0ECC24B3-E392-5AAC-94DE-807653F0E8D9

#### Anisolabis maritima

(Bonelli, 1832)

340DE9FE-0CAD-5452-9F55-8B968262E829

##### Notes

Yoon (1978), Lee and Kwon (1981), Kwon et al. (1996), Lee and Jung (2001), Kim (2004), Lee et al. (2006), Kim and Yeom (2006), An (2008), Kim et al. (2009), Park et al. (2010), Park et al. (2011), Park et al. (2012), Park et al. (2013), Park and Jang (2016)

#### Euborellia annulipes

(Lucas, 1847)

2AF7AFBE-C36E-54F3-B32C-7A221D3DFBEB

##### Notes

Lee and Jung (2001), Lee et al. (2006), Kim and Yeom (2006)

#### Forficulidae

82CA65C9-8C68-5128-AEBE-B91B776CFFBE

#### Forficula scudderii

de Bormans, 1880

0371445D-8003-506F-9C29-B587F7ECA7BC

##### Notes

Park and Jang (2016)

#### Diptera

E6FA409B-E0A5-538F-8098-0D42DA925416

#### Anthomyiidae

1588B92C-58DA-577B-8B3B-AE8821110304

#### Delia platura

(Meigen, 1826)

B1225E6C-EC4C-5ABF-BDC0-F6200C01BC9D

##### Notes

Kwon et al. (1996), Lee and Jung (2001), Kim (2004), Lee et al. (2006), Park et al. (2010), Kim and Yeom (2006), An (2008), Lee (2016), Choi (2018)

#### Fucellia apicalis

Kertész, 1908

D7AB326A-C448-5638-AFF0-A0DBFB950525

##### Notes

Lee et al. (2006), Kim and Yeom (2006), Choi (2018)

#### Fucellia boninensis

Snyder, 1965

FA926658-3E11-5FE2-8364-95B4320DAB23

##### Notes

Lee et al. (2006), Kim and Yeom (2006), Choi (2018)

#### Pegomya cunicularia

(Rondani, 1866)

C5D363CB-E231-572C-8C26-89385CE3F298

##### Notes

Ryu et al. (2021)

#### Calliphoridae

6CF716CB-691D-5268-A2D3-0E260D6CBEEC

#### Calliphora lata

Coquillett, 1898

03E03D32-7C7E-50CF-9D2D-3E90EC2532F6

##### Notes

Lee (2016), Choi (2018)

#### Hemipyrellia ligurriens

(Wiedemann, 1830)

AAC8621C-2F4F-5C31-9951-9E9C036591C6

##### Notes

Lee and Jung (2001), Lee et al. (2006), Kim and Yeom (2006), An (2008)

#### Lucilia illustris

(Meigen, 1826)

C1B2F443-1CA7-5764-9BAF-2DDADB8F5C82

##### Notes

Ryu et al. (2021)

#### Lucilia porphyrina

(Walker, 1856)

30FA8C98-FAFB-5558-9109-3F85122241CD

##### Notes

Choi (2018)

#### Lucilia sericata

(Meigen, 1826)

33EA909D-A4FB-55B7-91A7-83E9FC035277

##### Notes

Lee and Kwon (1981), Kwon et al. (1996), Lee and Jung (2001), Kim (2004), Lee et al. (2006), Kim and Yeom (2006), Kim et al. (2009), Lee et al. (2009), Park et al. (2011), Park et al. (2013), Park and Jang (2016), Lee (2016), Choi (2018)

#### Stomorhina obsoleta

(Wiedemann, 1830)

148C9C82-C047-5A25-B5EF-EAC7AC166CCD

##### Notes

Choi (2018)

#### Ceratopogonidae

AC051C8E-8E0F-58C1-851E-96416DAC2BE6

#### Culicoides circumscriptus

Kieffer, 1918

85D7B8B0-731F-5804-8BC1-EAB0756F29B2

##### Notes

Choi (2018)

#### Culicoides dokdoensis

Lee & Bae, 2023

C2005E8E-E545-5FF5-A029-4EB8F60AA3B0

##### Notes

Lee et al. (2023)

#### Chironomidae

DCC7FE28-B942-55CE-8BF0-9BD2C9936B12

#### Chironomus dorsalis

Meigen, 1818

007B2FD9-B0E1-5B92-AE2A-60E9B4CF3781

##### Notes

Info and Park (2023)

#### Chloropidae

482902AB-8702-518C-8F71-AA976D4AC2A2

#### Thaumatomyia notata

(Meigen, 1830)

10C992CB-4D04-5750-939B-FBF18490F129

##### Notes

An (2008)

#### Coelopidae

F664E23B-C5B8-5C8D-B30A-EFFDFE5FF1E2

#### Coelopa frigida

(Fabricius, 1805)

D0E56EE9-0C41-583A-A32E-3198CFDDC4D2

##### Notes

Choi (2017), Lee (2016), Ryu et al. (2021)

#### Culicidae

2CC4D6D9-365A-5E39-8280-14FC4E125FEE

#### Aedes togoi

(Theobald, 1907)

B54CFB5A-B8A5-510F-A79A-5D4EE3EB2DFF

##### Notes

Kang et al. (2013)

#### Culex orientalis

Edwards, 1921

75AF4BF6-064A-52FD-8EBE-3ADA85E47478

##### Notes

Yoon (1978), Lee and Kwon (1981), Kwon et al. (1996), Lee and Jung (2001), Kim (2004), Lee et al. (2006), Kim and Yeom (2006), An (2008), Kim et al. (2009), Choi (2018)

#### Heleomyzidae

184EE06B-C07C-5C4D-95B5-FD5E5AE25BF2

#### Tephrochlamys japonica

Okadome, 1967

D1B695BE-0AF7-5AEB-9C9F-672EC96F539B

##### Notes

Bang et al. (2022)

#### Muscidae

D81E96EE-23B1-565C-897F-DC170C9C968E

#### Atherigona oryzae

Malloch, 1925

6F9F0CC7-B74A-5B3B-BF5A-0641671AE95C

##### Notes

Choi (2018)

#### Musca bezzii

Patton & Cragg, 1913

A93B0E46-E149-59B7-88EF-098E765DB462

##### Notes

Yoon (1978), Lee and Kwon (1981), Kwon et al. (1996), Lee and Jung (2001), Kim (2004), Lee et al. (2006), Kim and Yeom (2006), Kim et al. (2009), Park et al. (2011)

#### Musca hervei

Villeneuve, 1922

FD40D776-76B9-5A0E-9479-0C46C09C697B

##### Notes

Yoon (1978), Lee and Kwon (1981), Kwon et al. (1996), Lee and Jung (2001), Kim (2004), Lee et al. (2006), Kim and Yeom (2006)

#### Orchisia costata

(Meigen, 1826)

912E816C-EF0B-5059-BD7C-CF6397CC30C2

##### Notes

Choi (2018)

#### Phoridae

55F0243E-8668-543D-902E-247718339C87

#### Megaselia spiracularis

Schmitz, 1938

A52861CF-107D-5D1F-9136-2B7E897903A7

##### Notes

Kang et al. (2013)

#### Psychodidae

24B845D5-E55C-5D67-A0A2-810F63FC6073

#### Psychoda alternata

(Say, 1824)

0AC6FBF0-3119-5AF3-BD3D-276D703AE23A

##### Notes

Kwon et al. (1996), Lee and Jung (2001), Kim (2004), Lee et al. (2006), Kim and Yeom (2006), Kang et al. (2013)

#### Sarcophagidae

AB482321-CF75-5604-994C-6F1C12C83587

#### Sarcophaga brevicornis

Ho, 1934

BFA80106-C7F9-587B-9FD8-DF5DF5ED71DC

##### Notes

Choi (2018)

#### Sarcophaga melanura

Meigen, 1826

F7EB6605-9340-5635-8099-4ABC9DDAFD79

##### Notes

Choi (2018)

#### Sarcophaga peregrina

(Robineau-Desvoidy, 1830)

84294C6A-C40B-5F4C-8A16-363470F4C421

##### Notes

Choi (2018)

#### Scathophagidae

637BF9F7-86CA-5BD1-86FD-1FABF5EF7D1A

#### Scathophaga stercoraria

(Linnaeus, 1758)

C3848A18-B5FE-5443-90C4-D53548F6F917

##### Notes

Ryu et al. (2021)

#### Sepsidae

E0CA1CC2-96C2-5A47-8FFA-8C6B69ADDA92

#### Sepsis monostigma

Thomson, 1869

9D4929D5-6EE4-5963-8C7A-9551702831CC

##### Notes

Choi (2018)

#### Syrphidae

43C8A2A4-BD01-5FCD-8AE7-6D56B7EA3611

#### Allobaccha apicalis

(Loew, 1858)

8472BDB8-C1EA-50D7-8490-54EA0434F852

##### Notes

Choi (2017), Choi (2018)

#### Allograpta javana

(Wiedemann, 1824)

1420ABC7-3AC9-5495-B66A-63D7805B0C49

##### Notes

Lee et al. (2006), Kim and Yeom (2006), Kim et al. (2009), Park et al. (2011), Park et al. (2013), Choi (2018)

#### Betasyrphus serarius

(Wiedemann, 1830)

868346D7-106B-5DAA-AD63-0039629ADF9E

##### Notes

Park et al. (2013), Ryu et al. (2021)

#### Dasysyrphus bilineatus

(Matsumura, 1917)

C2FE675D-FCDE-50C3-9AC1-6390F7191EC9

##### Notes

Choi (2018)

#### Episyrphus balteatus

(De Geer, 1776)

A1E5C7D3-C85B-5A64-B274-37F84D59DDE7

##### Notes

Kim (2004), Lee et al. (2006), Kim and Yeom (2006), An (2008), Kim et al. (2009), Lee et al. (2009), Park et al. (2011), Choi (2015), Park and Jang (2016), Lee (2016), Choi (2018), Ryu et al. (2021)

#### Eristalis cerealis

Fabricius, 1805

15ED1749-F0CC-507E-BF77-B4024D7845F3

##### Notes

Lee et al. (2006), Kim and Yeom (2006), Choi (2018)

#### Eristalis tenax

(Linnaeus, 1758)

3AB87A26-0074-5C75-BF25-2419EC08AF74

##### Notes

Kim (2004), Lee et al. (2006), Kim and Yeom (2006), Choi (2018)

#### Eupeodes corollae

(Fabricius, 1794)

463B8043-D462-521B-9526-01E8C7FB6119

##### Notes

Lee and Kwon (1981), Kwon et al. (1996), Lee and Jung (2001), Kim (2004), Lee et al. (2006), Kim and Yeom (2006), An (2008), Kim et al. (2009), Park et al. (2011), Park et al. (2013), Choi (2015), Park and Jang (2016), Lee (2016), Choi (2018), Ryu et al. (2021)

#### Eupeodes nitens

(Zetterstedt, 1843)

E3B3E851-C8EF-5A7D-8D52-CE17C5114A2A

##### Notes

An (2008)

#### Melanostoma mellinum

(Linnaeus, 1758)

FFF01811-843F-5D77-9F90-C09B9C3A861B

##### Notes

Park et al. (2011), Park et al. (2013), Choi (2015), Park and Jang (2016), Lee (2016), Choi (2018), Ryu et al. (2021)

#### Scaeva komabensis

(Matsumura, 1918)

0303E099-2330-5BCF-BDBD-897106A7BA5E

##### Notes

Choi (2018)

#### Sphaerophoria menthastri

(Linnaeus, 1758)

DEEA6C1E-4300-5783-AB46-8FDFC2C49E12

##### Notes

Lee and Jung (2001), Kim (2004), Lee et al. (2006), Kim and Yeom (2006), An (2008), Kim et al. (2009), Park et al. (2011), Choi (2015), Park and Jang (2016), Choi (2017), Choi (2018), Ryu et al. (2021)

#### Xanthandrus comtus

(Harris, 1780)

162BB9D0-28AB-51E7-9D33-0DF9B38FF18F

##### Notes

Lee et al. (2006), Kim and Yeom (2006), Choi (2015)

#### Tephritidae

59729855-FE4B-52B2-B455-B1B3053AD581

#### Campiglossa sada

(Dirlbek & Dirlbeková, 1974)

317583FC-1E5C-5968-A3DB-C20BB06058D5

##### Notes

Ryu et al. (2021)

#### Ensina sonchi

(Linnaeus, 1767)

88949D75-5606-52C1-B256-2F12427DE24D

##### Notes

Lee et al. (2009), Choi (2018)

#### Trupanea convergens

(Hering, 1936)

C9223AB3-3BDF-5B20-91FA-795B44881E58

##### Notes

Lee et al. (2009), Choi (2018)

#### Hemiptera

4BC1A9E4-F57A-5F7F-8127-60B3F8D447E7

#### Alydidae

B6965FF6-F9F5-50F8-BDEF-B49B72EF0BBB

#### Leptocorisa chinensis

(Dallas, 1852)

550C526B-EAE9-5EFB-8805-C63531C46789

##### Notes

Choi (2018)

#### Anthocoridae

85DE8611-3CA4-5734-94FE-92241362AA36

#### Orius sauteri

(Poppius, 1909)

4D63FEC6-F30F-55D7-82CE-DC17EBD9290E

##### Notes

Lee and Kwon (1981), Kwon et al. (1996), Lee and Jung (2001), Kim (2004), Lee et al. (2006), Kim and Yeom (2006), An (2008), Kim et al. (2009), Park et al. (2010), Cho and Lee (2012), Yoo et al. (2013), Park and Jang (2016), Lee (2016), Choi (2018)

#### Aphididae

8E5A10F6-86BF-5965-85F1-12712C89EF6D

#### Aphis nerii

Boyer de Fonscolombe, 1841

42367894-A87A-5B8B-A53E-B2815DA31D0D

##### Notes

Kim et al. (2009)

#### Aphis rumicis

Linnaeus, 1758

27ED0294-5125-5B20-81A5-75883F8933D1

##### Notes

Park et al. (2010)

#### Cicadellidae

23F5C609-7649-53AB-9840-D3EDD0E69CCE

#### Balclutha rubrinervis

(Matsumura, 1902)

BE52C189-764F-5167-84C4-5F3366616C0F

##### Notes

Lee and Kwon (1981), Kwon et al. (1996), Lee and Jung (2001), Kim (2004), Lee et al. (2006), Kim and Yeom (2006), An (2008), Kim et al. (2009), Ryu et al. (2021)

#### Chlamisus diminutus

(Gressitt, 1942)

E5FCB409-2954-5CFF-97CA-879E92D0F41D

##### Notes

Choi (2018)

#### Hishimonus sellatus

(Uhler, 1896)

073F78D0-FA7F-596A-A8FD-992798A5FE8A

##### Notes

Lee et al. (2006), Kim and Yeom (2006), Choi (2018)

#### Laburrus impictifrons

(Boheman, 1852)

4D7A6398-9786-5932-BFB9-BB139481A77B

##### Notes

Kwon et al. (1996), Lee and Jung (2001), Kim (2004), Lee et al. (2006), Lee (2016), Kim and Yeom (2006), An (2008), Kim et al. (2009), Park and Jang (2016), Choi (2018)

#### Macrosteles striifrons

Anufriev, 1968

5A7DC87E-76DB-572C-AB9A-F80C04F24CD7

##### Notes

Choi (2018)

#### Maiestas oryzae

(Matsumura, 1902)

DED61DE5-88D1-5647-86C9-9E110EAC3E10

##### Notes

Lee and Kwon (1981), Kwon et al. (1996), Lee and Jung (2001), Kim (2004), Lee et al. (2006), Kim and Yeom (2006), An (2008), Lee et al. (2009)

#### Psammotettix striata

(Linnaeus, 1758)

16E73978-7D50-50BB-81DB-A2454DA0673F

##### Notes

Choi (2018)

#### Recilia coronifera

(Marshall, 1866)

97EF5FEA-7A02-5396-9799-9D1DEF49BF81

##### Notes

Choi (2018), Ryu et al. (2021)

#### Cydnidae

4B82BB5C-83BE-595C-BA36-CA41BB693416

#### Fromundus pygmaeus

(Dallas, 1851)

5EDF9EB0-8C07-597E-8618-741A33B5933D

##### Notes

Yoon (1978), Lee and Kwon (1981), Kwon et al. (1996), Lee and Jung (2001), Kim (2004), Lee et al. (2006), Kim and Yeom (2006), An (2008), Kim et al. (2009), Park et al. (2010), Park et al. (2011), Park et al. (2012), Park et al. (2013), Choi (2015), Park and Jang (2016), Lee (2016), Choi (2018), Ryu et al. (2021)

#### Delphacidae

B2256D75-A766-5B9F-8B27-12B6F213F515

#### Laodelphax striatellus

(Fallén, 1826)

9856749D-DBEF-5AA0-B238-7779D079BC3B

##### Notes

Lee and Kwon (1981), Kwon et al. (1996), Lee and Jung (2001), Kim (2004), Park et al. (2011), Lee et al. (2006), Kim and Yeom (2006), An (2008), Lee et al. (2009), Choi (2018), Ryu et al. (2021)

#### Sogatella furcifera

(Horváth, 1899)

4722AAD4-0CD9-51E4-B41F-2C29FC60508C

##### Notes

Lee and Kwon (1981), Kwon et al. (1996), Lee and Jung (2001), Kim (2004), Lee et al. (2006), Kim and Yeom (2006), An (2008), Lee et al. (2009), Park and Jang (2016), Lee (2016), Ryu et al. (2021)

#### Sogatella kolophon

(Kirkaldy, 1907)

1477DF58-DFA0-5324-B67C-AB12A4200754

##### Notes

Lee and Kwon (1981), Kwon et al. (1996), Lee and Jung (2001), Kim (2004), Lee et al. (2006), Kim and Yeom (2006), Lee et al. (2009), Lee (2016), Choi (2015), Ryu et al. (2021)

#### Unkanodes sapporonus

(Matsumura, 1935)

D1920999-91A3-5429-88BC-ED10B9453159

##### Notes

Lee and Kwon (1981), Kwon et al. (1996), Lee and Jung (2001), Kim (2004), Lee et al. (2006), Kim and Yeom (2006), An (2008), Lee et al. (2009), Park and Jang (2016), Lee (2016), Choi (2018)

#### Geocoridae

A535E6C8-155E-5145-992D-768C73035A14

#### Geocoris pallidipennis

(Costa, 1843)

5F337D3F-5DE2-59F6-8AE0-A82173D6DFDF

##### Notes

Bang et al. (2022)

#### Lygaeidae

5AB68E57-A890-54CD-9865-C9E62E7A3843

#### Nysius plebeius

Distant, 1883

A2639975-ED3E-5887-A017-4A574A44C147

##### Notes

Lee et al. (2006), Kim and Yeom (2006), An (2008), Lee et al. (2009), Park et al. (2010), Park et al. (2012), Park et al. (2013), Choi (2015), Park and Jang (2016), Lee (2016), Choi (2018), Ryu et al. (2021)

#### Miridae

A1E0CCD6-AE71-56B5-BCC4-104E43511508

#### Campylomma lividicornis

Reuter, 1912

E92EEF04-0CA5-5A9E-A1A1-109B3C44135D

##### Notes

Lee and Kwon (1981), Kwon et al. (1996), Lee and Jung (2001), Kim (2004), Lee et al. (2006), Kim and Yeom (2006), An (2008), Park and Jang (2016), Choi (2018)

#### Creontiades coloripes

Hsiao & Meng, 1963

FBD3C73A-52A5-5762-B88E-D353E4364E1A

##### Notes

Choi (2018)

#### Nesidiocoris tenuis

(Reuter, 1895)

7E008839-22AC-50B2-A5DD-16E4275DA882

##### Notes

Choi (2018)

#### Orthotylus flavosparsus

(Sahlberg, 1841)

13C32DC3-7379-5B26-8C69-0FFE459D4D23

##### Notes

Lee and Kwon (1981), Kwon et al. (1996), Lee and Jung (2001), Kim (2004), Lee et al. (2006), Kim and Yeom (2006), An (2008), Lee et al. (2009), Park et al. (2010), Park et al. (2011), Park and Jang (2016), Choi (2018)

#### Trigonotylus caelestialium

(Kirkaldy, 1902)

62192F76-AE59-5B99-8919-6F7E08962C76

##### Notes

An (2008), Choi (2018)

#### Nabidae

C1660457-96CB-50FF-AD16-F778A51634E6

#### Nabis stenoferus

Hsiao, 1964

EDC55225-0224-54F7-8413-3F7289E84FD2

##### Materials

- **Type status:** Other material. **Occurrence:** recordedBy: Lee, D. Y.; sex: 1 female; lifeStage: adult; occurrenceID: 47EC8D41-1B42-55A8-98D7-4D45BE40D987; **Location:** island: Dokdo Island; country: Republic of Korea; stateProvince: Gyeongsangbuk-do; county: Ulleung-gun; locality: Dongdo of Dokdo, Dokdo-ri, Ulleungeup; **Event:** samplingProtocol: sweeping net; eventDate: 2022-05-18

##### Notes

This is the first record of this species from Dokdo Island.

Distribution: China, Japan, Korea, Russia (Jung and Lee 2017)

#### Prostemma hilgendorfii

Stein, 1878

E43ECD48-4AF5-5E19-94CE-E99179231AA8

##### Notes

Lee and Kwon (1981), Kwon et al. (1996), Lee and Jung (2001), Kim (2004), Lee et al. (2006), Kim and Yeom (2006), An (2008), Kim et al. (2009), Lee et al. (2009)

#### Pentatomidae

1A8CB580-7E8A-561F-8C57-3A5026D88D92

#### Glaucias subpunctatus

(Walker, 1867)

545854BF-D4C2-5F47-AA5D-C903ECD4FA42

##### Notes

Choi (2018)

#### Nezara antennata

Scott, 1874

59CB3301-8FEB-5BCD-B05F-055D5DD80FF1

##### Notes

An (2008)

#### Piesmatidae

E6976A0A-A5A5-52CE-B365-89E74FED406D

#### Piesma capitata

(Wolff, 1804)

73A08D17-3EA6-5B3E-93C2-E950076D5603

##### Notes

Kwon et al. (1996), Lee and Jung (2001), Kim (2004), Lee et al. (2006), Kim and Yeom (2006), An (2008), Kim et al. (2009), Lee et al. (2009)

#### Piesma maculata

(Laporte, 1833)

1287D216-B0B8-5CBC-A4AD-FFFAEEA36C87

##### Notes

Lee and Kwon (1981), Kwon et al. (1996), Lee and Jung (2001), Kim (2004), Lee et al. (2006), Kim and Yeom (2006), An (2008)

#### Rhopalidae

F13D23EC-8549-56B5-9728-E125E3DCC301

#### Liorhyssus hyalinus

(Fabricius, 1794)

A012D9D9-85B9-50BC-807F-C1B491E3A553

##### Notes

Choi (2018)

#### Rhopalus (Aeschyntelus) maculatus

(Fieber, 1837)

CF20A3C3-8529-5717-BB64-E01E1DD97B61

##### Notes

Hwang (2023)

#### Rhyparochromidae

E545B139-612B-5C23-9568-7FEA39C682EC

#### Horridipamera inconspicua

(Dallas, 1852)

6FE5DFFA-319D-54ED-AE24-AA84A7458AC4

##### Notes

Choi (2018)

#### Paradieuches dissimilis

(Distant, 1883)

C69D8A90-3991-5AA6-9546-836984EBD977

##### Notes

Kang et al. (2013)

#### Paromius exiguus

(Distant, 1883)

40A5C075-81BC-54F9-851D-7B1B58D3ADFD

##### Notes

Hwang (2023)

#### Stigmatonotum rufipes

(Motschulsky, 1866)

04505AA0-0409-5D32-819D-14360CA5D2F3

##### Notes

Kwon et al. (1996), Lee and Jung (2001), Kim (2004), Lee et al. (2006), Kim and Yeom (2006), An (2008), Kim et al. (2009), Park and Jang (2016), Choi (2018)

#### Scutelleridae

02945BB1-E720-5252-ADEF-62BA92EF0C46

#### Cantao ocellatus

(Thunberg, 1784)

6271CE8D-277F-5E69-A42E-A6650CB3F3CE

##### Notes

Choi (2015)

#### Tingidae

F7A97D36-EB86-5BEE-BD18-996F8F1FE70D

#### Cantacader lethierryi

Scott, 1874

691F00E7-10E7-5FE2-B643-97CC76C0C332

##### Notes

Lee and Kwon (1981), Kwon et al. (1996), Lee and Jung (2001), Kim (2004), Lee et al. (2006), Kim and Yeom (2006), An (2008)

#### Triozidae

50902774-37E7-58B2-A10C-280CEDCC19E4

#### Trioza chenopodii

Reuter, 1876

4B95AE6C-EF38-52C6-AF07-E4E6294AB10C

##### Notes

Lee and Kwon (1981), Kwon et al. (1996), Lee and Jung (2001), Kim (2004), Lee et al. (2006), Kim and Yeom (2006), An (2008), Park and Jang (2016), Choi (2018)

#### Hymenoptera

79767504-A268-5EA9-A15E-2A3DF4C15AB4

#### Bethylidae

2DEE4005-4639-5E14-B14F-6DC5CA062ECE

#### Pristepyris minutus

(Yasumatsu, 1955)

C4F623E8-1201-5E4C-94DF-2D980C969CD4

##### Notes

Cho and Lee (2012)

#### Chalcididae

A4AEAA11-FA8D-5BDA-9CC1-DC16F08592CD

#### Brachymeria femorata

(Panzer, 1801)

BCABEA6D-A572-5335-BB17-BBBD5F676691

##### Notes

Lee (2016), Choi (2018)

#### Brachymeria minuta

(Linnaeus, 1767)

B0DA2205-53B0-504B-BA44-4BD3C3F606CE

##### Notes

Ryu et al. (2021)

#### Dryinidae

4778DE0B-08DF-5AA2-B03A-56E4E6180E54

#### Haplogonatopus oratorius

(Westwood, 1833)

9CAE278D-E07A-54ED-A011-D27D555DCF25

##### Materials

- **Type status:** Other material. **Occurrence:** recordedBy: Lee, D. Y.; sex: 1 female; lifeStage: adult; occurrenceID: 43AE5048-BFEB-5B3E-8FA5-A8A1C69FAB5A; **Location:** island: Dokdo Island; country: Republic of Korea; stateProvince: Gyeongsangbuk-do; county: Ulleung-gun; locality: Dongdo of Dokdo, Dokdo-ri, Ulleungeup; **Event:** samplingProtocol: sweeping net; eventDate: 2022-05-18

##### Notes

This is the first record of this species from Dokdo Island.

Distribution: Australia, Austria, China, France, Germany, Hungary, Iran, Italy, Japan, Korea, North Africa, Poland, Romania, Russia, Spain, Sweden, United Kingdom (Ghahari et al. 2024)

#### Eulophidae

47C67EBD-D5F7-5BE2-940D-BE410D925861

#### Diglyphus isaea

(Walker, 1838)

B25D07B1-DF19-52CE-83A7-2E68474F0D78

##### Notes

Hwang (2023)

#### Eupelmidae

21D8745F-BE5C-5F7C-A73B-CFA2BFC67F93

#### Eupelmus australiensis

(Girault, 1913)

B806F4A5-1E29-5E57-B19B-EC8EBC916152

##### Notes

Ryu et al. (2021)

#### Formicidae

7E6CE622-0F6E-51CA-96E1-722199DE7CAD

#### Brachyponera chinensis

(Emery, 1895)

39828BBB-66A3-5609-B4B0-00E4922E175C

##### Notes

Choi (2015), Park and Jang (2016), Lee (2016)

#### Hypoponera nippona

(Santschi, 1937)

3107202C-3D7C-57DE-975F-A8CC5BDF3DD0

##### Notes

Kim and Yeom (2006)

#### Lasius meridionalis

(Bondroit, 1920)

123CF249-C3EF-5A21-8FAF-E89C0290A9DB

##### Notes

Lee and Jung (2001), Lee et al. (2006), Kim and Yeom (2006)

#### Monomorium Chinensis

(Santschi, 1925)

F2B32F20-8C67-5006-A872-D3C3D5D515D3

##### Notes

Kim and Yeom (2006)

#### Monomorium intrudens

Smith, 1874

A6F30D3D-535F-5661-A902-B26EC89E2D52

##### Notes

Lee et al. (2009), Park et al. (2010), Park et al. (2011), Park et al. (2012), Park et al. (2013), Choi (2015), Park and Jang (2016), Lee (2016)

#### Myrmecina graminicola nipponica

Wheeler, 1906

71CA6E0C-9179-52C2-8140-766BD30159E0

##### Notes

Lee and Jung (2001), Lee et al. (2006), Kim and Yeom (2006), An (2008), Kim et al. (2009)

#### Pheidole fervida

Smith, 1874

C1DE4CFE-C1BB-5D8B-A3DD-3C1352098B56

##### Notes

Park et al. (2011), Choi (2015)

#### Ponera japonica

(Wheeler, 1906)

AA13F67D-E2D8-5F70-B92B-82654118C312

##### Notes

Kim and Yeom (2006), Lee et al. (2009)

#### Pristomyrmex punctatus

(Smith, 1860)

475265FD-7509-53ED-9B4B-E7AB5A6328CE

##### Notes

Lee and Jung (2001), Lee et al. (2006), Kim and Yeom (2006)

#### Solenopsis japonica

Wheeler, 1928

4D493110-F25B-5B31-BABD-25B6F15D0358

##### Notes

Lee and Jung (2001), Lee et al. (2006), Kim and Yeom (2006), Lee (2016)

#### Strumigenys lewisi

Cameron, 1886

09C071E4-779F-50B1-A307-4113C32EA57C

##### Notes

Kim and Yeom (2006)

#### Tetramorium tsushimae

Emery, 192

BF4E02AE-F3E3-5399-B7FE-9DFB7F6B4379

##### Notes

Lee and Jung (2001), Lee et al. (2006), Kim and Yeom (2006), Lee et al. (2009), Park et al. (2011), Park et al. (2013), Choi (2015), Park and Jang (2016)

#### Pteromalidae

DD203C00-5629-5016-9157-4F6BE875F6AC

#### Halticoptera circulus

(Walker, 1833)

14EF2E61-168D-5E10-B5E5-0D9ED3142F5D

##### Notes

Lee (2016), Ryu et al. (2021)

#### Lepidoptera

B7791706-AF00-531C-8C45-B2E512EF1D2A

#### Crambidae

330755CD-7FF2-5CE6-B49E-474C377078E9

#### Cnaphalocrocis medinalis

(Guenée, 1854)

F5F49C28-7725-5B5E-A6AC-38F8176CB74A

##### Notes

Kwon et al. (1996), Lee and Jung (2001), Kim (2004), Lee et al. (2006), Kim and Yeom (2006)

#### Diaphania indica

(Saunders, 1851)

7CEF4FDF-7082-555F-B9F4-7EC20829A386

##### Notes

Kwon et al. (1996), Lee and Jung (2001), Kim (2004), Lee et al. (2006), Kim and Yeom (2006), An (2008)

#### Maruca vitrata

(Fabricius, 1787)

E1C5068B-E635-56E8-AC6F-9CE56C80ED7F

##### Notes

An (2008)

#### Palpita nigropunctalis

Bremer, 1864

BF1B72A0-F01F-5ED4-AC4E-C39CEF7515AB

##### Notes

Yoon (1978), Lee and Kwon (1981), Kwon et al. (1996), Lee and Jung (2001), Kim (2004), Lee et al. (2006), Kim and Yeom (2006), An (2008)

#### Spoladea recurvalis

(Fabricius, 1775)

B0AA884D-8C22-5FF0-99DF-C4D3C87DAACE

##### Notes

Lee and Kwon (1981), Kwon et al. (1996), Lee and Jung (2001), Kim (2004), Lee et al. (2006), Kim and Yeom (2006), An (2008), Kim et al. (2009), Lee et al. (2009), Park et al. (2010), Park et al. (2013), Choi (2018)

#### Erebidae

B57E0219-9E41-5876-A615-1E1965CECD14

#### Arcte coerula

(Guenée, 1852)

FE269F9E-63F8-5C7E-A2FC-EBFF460B50BB

##### Notes

Choi (2015)

#### Catocala dula

Bremer, 1861

132DDEE4-00FC-53F1-836B-C2266D115D91

##### Notes

Kim and Yeom (2006)

#### Daddala lucilla

(Butler, 1881)

10E41DCC-E35D-5FD3-8A7F-6E9B023BF652

##### Notes

Kim and Yeom (2006)

#### Thyas juno

(Dalman, 1823)

F8D15BC3-AB28-5245-B721-208B1618F6B7

##### Notes

Park et al. (2011), Choi (2015)

#### Geometridae

09B945C7-0BCA-53C3-93F1-1BD5F89EF959

#### Odontopera arida

(Butler, 1878)

4888EDC6-2C0C-5CCC-BD88-91715D7475F2

##### Notes

An (2008)

#### Scopula ignobilis

(Warren, 1901)

0A3167E2-A601-57A5-8E6C-1EAF2CD06EFF

##### Notes

Kim (2004), Lee et al. (2006), Kim and Yeom (2006)

#### Hesperiidae

43FEBA39-EA51-5705-A771-BF5BCA55E78D

#### Parnara guttatus

(Bremer & Grey, 1852)

598F0A29-F654-5E4E-9917-D64B987697C7

##### Notes

Kim et al. (2009)

#### Lycaenidae

31ACD3C3-ED62-58E6-9BEC-9D6CC5A69FBA

#### Arhopala bazalus

(Hewitson, 1862)

47B8DA88-32B3-5FF3-BB59-676379C4AC96

##### Notes

Kim and Yeom (2006)

#### Zizeeria maha

(Kollar, 1844)

3B6D0292-08A5-5E04-8814-9D4D6A64280E

##### Notes

Lee and Kwon (1981), Kwon et al. (1996), Lee and Jung (2001), Kim (2004), Lee et al. (2006), Kim and Yeom (2006), Kim et al. (2009)

#### Noctuidae

E143BADB-0017-56AC-AD9D-0A12575F5112

#### Agrotis ipsilon

(Hufnagel, 1766)

B6955850-D931-5941-909D-C65929439011

##### Notes

Kim and Yeom (2006), An (2008)

#### Callopistria argyrosticta

Butler, 1881

DE45F6D6-CC79-5EBD-B06B-91EEE88590A6

##### Notes

An (2008)

#### Cosmia achatina

Butler, 1879

8FDF55C4-F422-5231-85EE-7872228BCE7E

##### Notes

Kim and Yeom (2006)

#### Diarsia canescens

(Butler, 1878)

029003BB-0734-5265-979F-BD5DF4FF087B

##### Notes

An (2008)

#### Macdunnoughia confusa

(Stephens, 1850)

BD45EFCC-8C82-50B3-A744-31BBE656EDFA

##### Notes

An (2008)

#### Macdunnoughia crassisigna

(Warren, 1913)

5F9E3896-F33A-53AE-912A-390FC0C7A8DC

##### Notes

Hwang (2023)

#### Mythimna separata

(Walker, 1865)

F532B98B-85C5-557C-893C-79143ACB12CE

##### Notes

Lee and Jung (2001), Lee et al. (2006), Kim and Yeom (2006), An (2008), Lee et al. (2009)

#### Nymphalidae

DA3524F3-217C-514D-9753-3BC6C6CE0E0C

#### Vanessa cardui

(Linnaeus, 1758)

ED805516-582D-5A97-9582-26C8BCBEB737

##### Notes

Lee and Kwon (1981), Kwon et al. (1996), Lee and Jung (2001), Kim (2004), Lee et al. (2006), Kim and Yeom (2006), An (2008), Kim et al. (2009), Lee et al. (2009), Park et al. (2011), Park et al. (2013), Choi (2018), Ryu et al. (2021)

#### Vanessa indica

(Herbst, 1794)

7CA536CF-BE2A-5D68-83EB-ED7ABF9861B7

##### Notes

Choi (2018)

#### Papilionidae

3A714D94-B142-5913-B7DF-31FC609C10C3

#### Papilio xuthus

Linnaeus, 1767

61B89E28-041B-5498-B71C-DFCA46760F65

##### Notes

Lee and Kwon (1981), Kwon et al. (1996), Lee and Jung (2001), Kim (2004), An (2008), Lee et al. (2006), Kim and Yeom (2006)

#### Pieridae

1BF7341B-EAEC-5D26-9608-B6512170A366

#### Colias erate

(Esper, [1805])

193924E1-954D-5D93-A29C-7BD591CF4460

##### Notes

Hwang (2023)

#### Plutellidae

46E6909F-DB1B-5446-BDD8-972A58B67346

#### Plutella xylostella

(Linnaeus, 1767)

D3372631-0BF4-5A4E-920A-B4D6B7D670FD

##### Notes

Kim and Yeom (2006)

#### Pyralidae

52E53CF8-FBBC-5635-A208-89D6535AD76D

#### Oncocera semirubella

(Scopoli, 1763)

7615300E-84AF-5C49-848A-C643FB1327DF

##### Notes

Kim and Yeom (2006), An (2008)

#### Sphingidae

97B8D381-B1B2-5061-A40B-932E9E322385

#### Macroglossum stellatarum

(Linnaeus, 1758)

FBBBF8ED-D81A-5DB0-A88F-A2EFEF0386B4

##### Notes

An (2008), Choi (2018)

#### Tortricidae

ED6045ED-8DFF-5212-AF58-6C237EC3BDC7

#### Adoxophyes orana

(Fischer von Röslerstamm, 1834)

33CE218F-5E68-551A-A739-D531C7BEBFF1

##### Notes

An (2008)

#### Archips oporana

(Linnaeus, 1758)

4C3A7988-331B-5E0A-8CD3-9E2A3FA2F21A

##### Notes

Lee et al. (2009)

#### Cochylidia contumescens

(Meyrick, 1931)

89D90202-77EE-59B4-A956-F96C783B4B89

##### Notes

Cho and Lee (2012)

#### Cochylidia richteriana

(Fischer von Röslerstamm, 1837)

FAF84F94-B2C2-5F69-A9F8-79C38C76769C

##### Notes

Lee et al. (2009)

#### Tortrix sinapina

(Butler, 1879)

24CEF0A6-19DE-584D-B1A4-7275AF3544B7

##### Notes

An (2008)

#### Yponomeutidae

97D61CB7-2A11-559E-9D22-A0F1D46CE3EB

#### Yponomeuta meguronis

(Matsumura, 1931)

088D4EBB-DD25-5015-9F14-FDEDEC922413

##### Notes

Kim and Yeom (2006), An (2008), Choi (2018)

#### Neuroptera

856723BD-6F39-59C2-A8AB-B0CA283CEBA0

#### Chrysopidae

CE6EBE18-67F5-5BD1-B9C6-EA384AA63EA0

#### Chrysopa pallens

(Rambur, 1838)

A9F8891F-CAE2-52FC-A72D-6944EE25FA0C

##### Notes

Kwon et al. (1996), Lee and Jung (2001), Kim (2004), Lee et al. (2006), Kim and Yeom (2006), An (2008), Lee et al. (2009), Park et al. (2011), Park et al. (2013), Choi (2018)

#### Cunctochrysa albolineata

(Killington, 1935)

81E0E75C-F4BC-5016-9FAF-B92457BBFEB7

##### Notes

Hwang (2023)

#### Hemerobiidae

33E0289E-7E7B-5F5D-93B1-52F1E818B4BC

#### Hemerobius humulinus

Linnaeus, 1758

17103B6A-09DE-5992-9F0B-DBAF83815C50

##### Notes

An (2008), Choi (2018)

#### Odonata

13B2B2FE-D9F7-5B1F-819C-B7C900A528DC

#### Aeshnidae

DA4EF0D7-ACC7-58AE-B6A1-DD286026C614

#### Anax parthenope

(Selys, 1839)

404F182E-04FF-5DEA-A3D6-8D51141F15A5

##### Notes

Yoon (1978), Lee and Kwon (1981), Kwon et al. (1996), Lee and Jung (2001), Kim (2004), Lee et al. (2006), Kim and Yeom (2006), An (2008), Park et al. (2013), Choi (2018)

#### Coenagrionidae

5F7881A4-3DC2-56AE-9BBB-F2050A6D0EAE

#### Ischnura asiatica

(Brauer, 1865)

BB047702-211E-5F1A-9A81-47DF9953B4C7

##### Notes

Lee et al. (2009), Park et al. (2013), Choi (2018)

#### Libellulidae

33290FF7-A80E-5267-8F80-359D095D649D

#### Pantala flavescens

(Fabricius, 1798)

77410462-06A1-540C-A1AA-42AD0811156A

##### Notes

Lee and Kwon (1981), Kwon et al. (1996), Lee and Jung (2001), Kim (2004), Lee et al. (2006), Kim and Yeom (2006), An (2008), Kim et al. (2009), Park et al. (2011), Park et al. (2013), Park and Jang (2016), Lee (2016), Choi (2018), Ryu et al. (2021)

#### Rhyothemis fuliginosa

Selys, 1883

98C5DDE5-965C-5701-8B9C-33ADF5BB7E15

##### Notes

An (2008)

#### Sympetrum darwinianum

(Selys, 1883)

A98FEB22-506B-559E-AEAC-AB41D8A1A9B5

##### Notes

An (2008)

#### Orthoptera

AA9AE200-0AF2-5023-A589-F1BCB6A8C71F

#### Gryllidae

B8A3CE60-D84B-5C86-9EF7-A6DBDB05E6C8

#### Teleogryllus emma

(Ohmachi & Matsuura, 1951)

1E019596-CB1C-51E7-96FA-C2D40CFFD580

##### Notes

Lee and Kwon (1981), Kwon et al. (1996), Lee and Jung (2001), Kim (2004), Lee et al. (2006), Kim and Yeom (2006)

#### Velarifictorus aspersus

(Walker, 1869)

C7FCF920-8DED-5050-8175-8F9774029A5A

##### Notes

Lee and Kwon (1981), Kwon et al. (1996), Lee and Jung (2001), Kim (2004), Lee et al. (2006), Kim and Yeom (2006), Kim et al. (2009)

#### Mogoplistidae

73A355AD-7820-5ADC-A686-A9818334E32A

#### Ornebius kanetataki

(Matsumura, 1904)

9A0863F7-8D7A-5DF6-B5A0-62F80379AF71

##### Notes

An (2008)

## Analysis

Through the historical compilation and field surveys of the insect fauna of Ulleungdo and Dokdo, any initial records and occurrence records that had arisen from misidentification or reference errors were re-identified, removed or corrected through verification of reference specimens and literature and data reviews. This process resulted in a report of 1694 species across 20 orders and 227 families, including 28 unrecorded species from Ulleungdo and 201 species across 10 orders and 80 families, including three unrecorded species from Dokdo.

**Figure 2.**
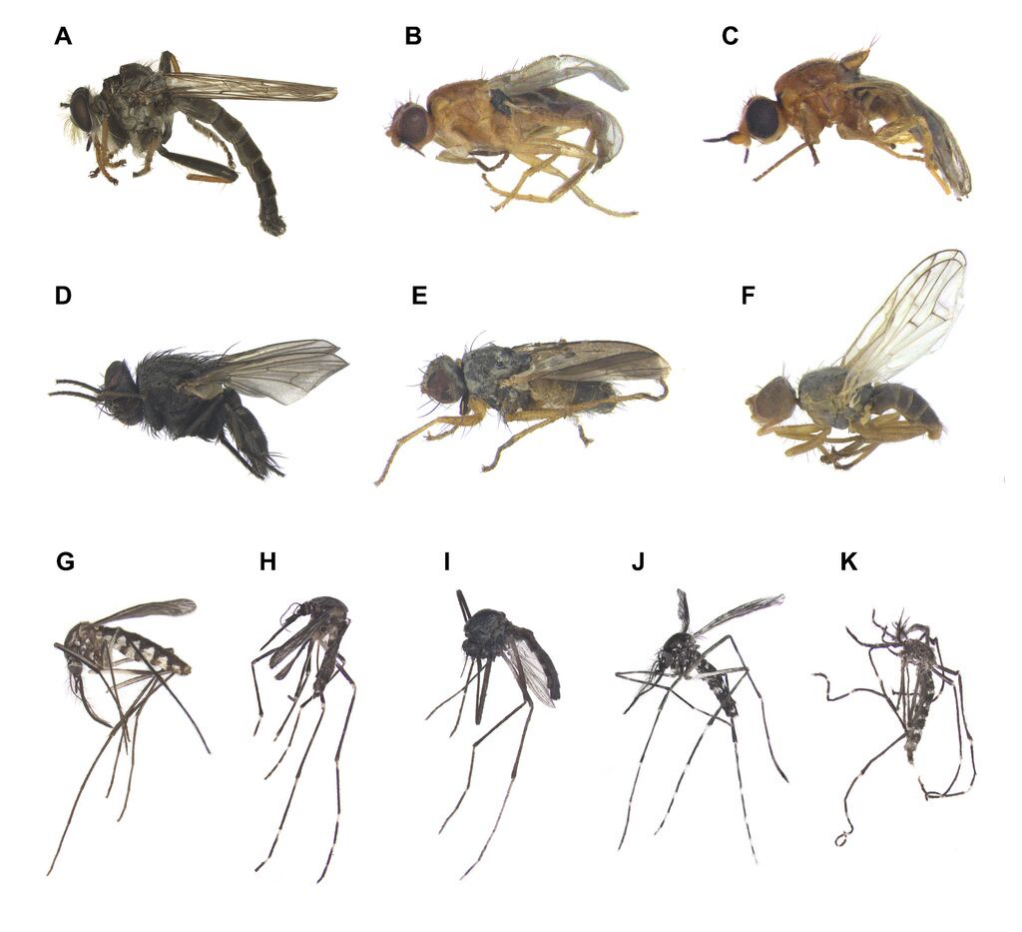
Newly-recorded species of Dipetra from Ulleungdo. **A** *Neoitamuscothurnatusunivittatus* (Loew, 1871); **B** *Elachipterainsignis* (Thomson, 1869); **C** *Elachipterasibirica* (Loew, 1858); **D** *Muscinalevida* (Harris, 1780); **E** *Orchisiacostata* (Meigen, 1826); **F** *Ensinasonchi* (Linnaeus, 1767); **G** *Armigeressubalbatus* (Coquillett, 1898); **H** *Aedeskoreicus* (Edwards, 1917); **I** *Aedesgalloisi* Yamada, 1921; **J** *Aedesalbopictus* (Skuse, 1894); **K** *Aedestogoi* (Theobald, 1907).

**Figure 3.**
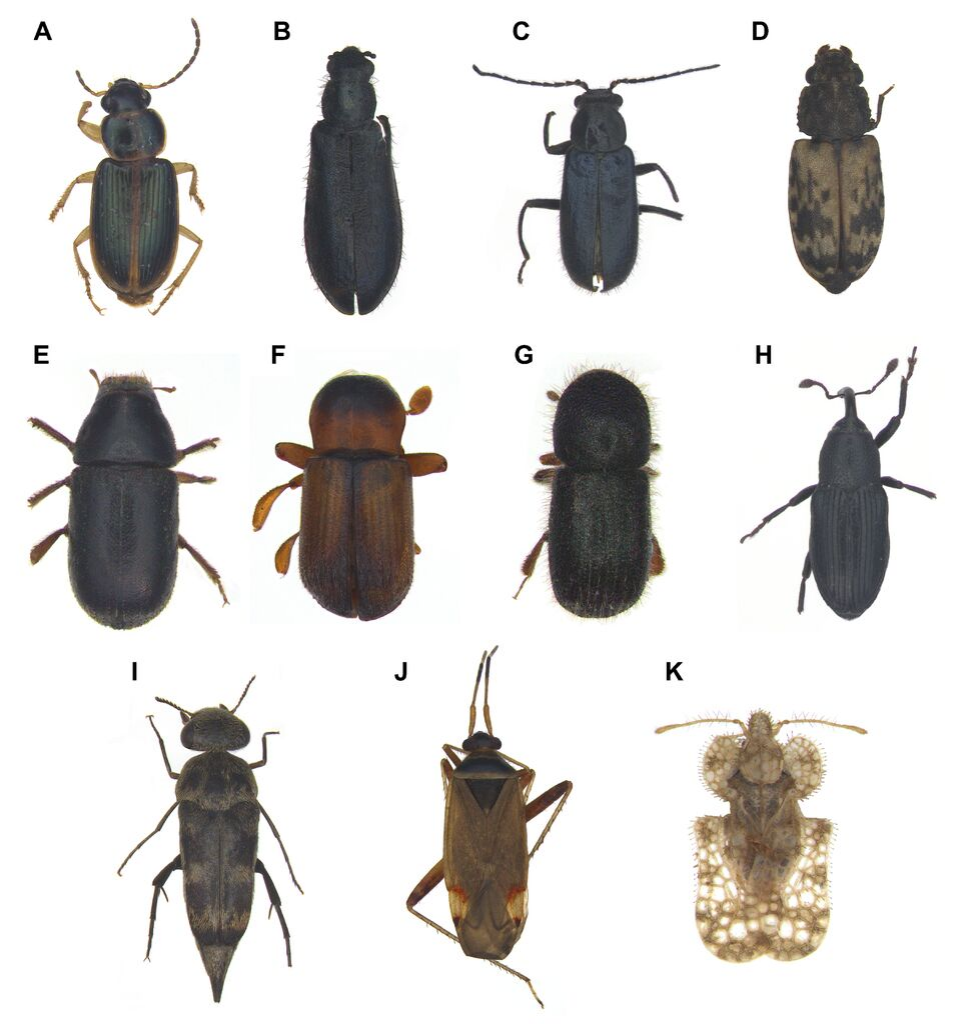
Newly-recorded species of Coleoptera and Hemiptera from Ulleungdo. **A** *Stenolophusdifficilis* (Hope, 1845); **B** *Dasytesvulgaris* Nakane, 1963; **C** *Attaluselongatulus* Lewis, 1895; **D** *Elacatiskraatzi* Reitter, 1879; **E** *Polygraphusjezoensis* Niisima, 1909; **F** *Scolytoplatypustycon* Blandford, 1893; **G** *Ambrosiophilusatratus* (Eichhoff, 1876); **H** *Psilarthroidesczerskyi* (Zaslavskij, 1956); **I** *Tomoxianipponica* Kôno, 1928; **J** *Adelphocorisdemissus* Horvath, 1905; **K** *Corythuchamarmorata* (Uhler, 1878).

**Figure 4.**
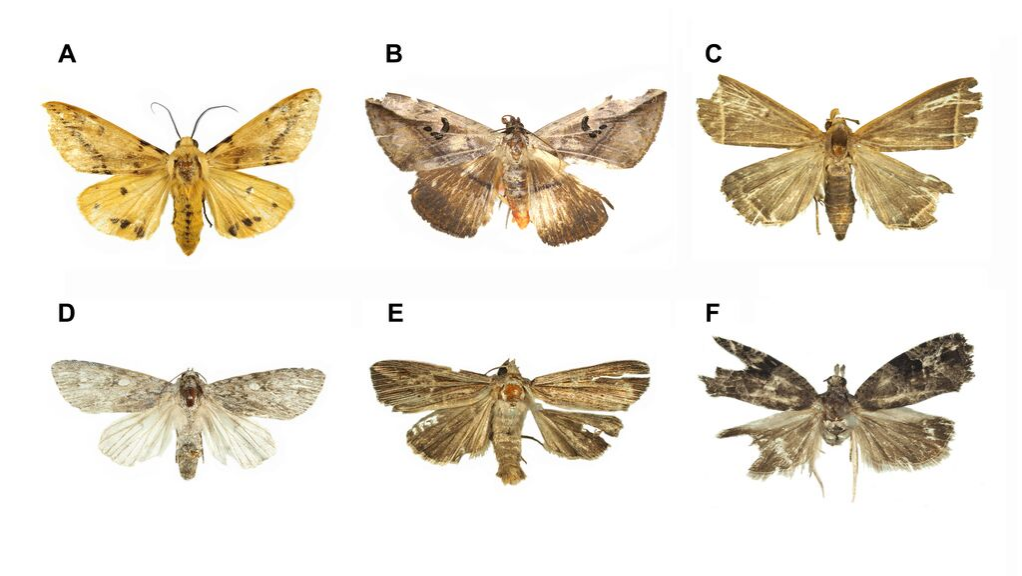
Newly-recorded species of Lepidoptera from Ulleungdo. **A** Spilarctiaseriatopunctatasubsp.suzukii Inoue & Maenami, 1963; **B** *Hypopyravespertilio* (Fabricius, 1787); **C** *Simpliciarectalis* (Eversmann, 1842); **D** *Acronictahercules* (Felder & Rogenhofer, 1874); **E** *Analetiapostica* (Hampson, 1905); **F** *Eudemisbrevisetosa* Oku, 2005.

**Figure 5.**
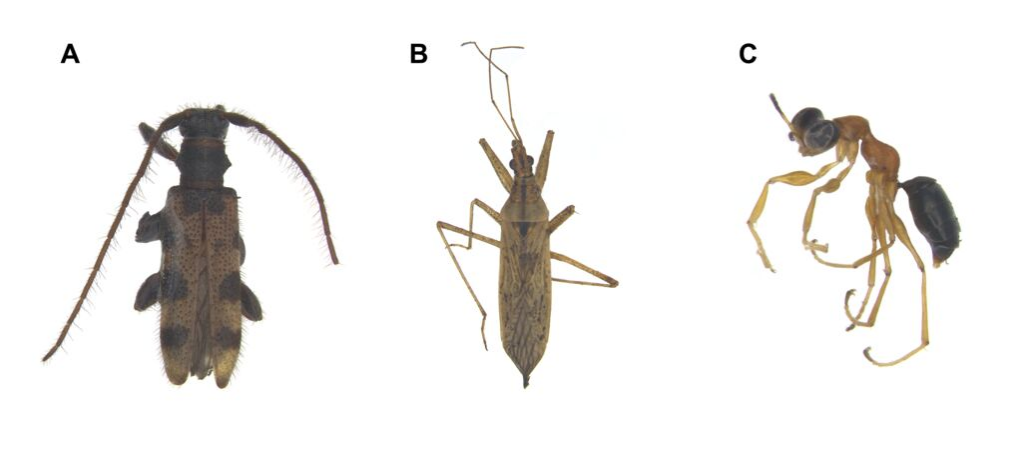
Newly-recorded species from Dokdo. **A** *Arhopaloscelisbifasciata* (Kraatz, 1879); **B** *Nabisstenoferus* Hsiao, 1964; **C** *Haplogonatopusoratorius* (Westwood, 1833).

In terms of newly-recorded species from the two islands, Ulleungdo saw the most additions within the order Diptera, with 11 newly-recorded species (Fig. 2), followed by nine newly-recorded species in Coleoptera, six in Lepidoptera and two in Hemiptera (Figs 3, 4). For Dokdo, three newly-recorded species were added each in the orders of Coleoptera, Hemiptera and Hymenoptera (Fig. 5).

The insect fauna checklist for Ulleungdo includes three species endemic to the island: *Aphisveroniciphaga* Kim & Lee, 2006, *Cacopsyllaulleungensis* (Kwon, 1983) and *Rhomphocalluscoreanus* Assing, 2011 (*Kwon 1983, Kim et al. 2006, Assing 2011*). Dokdo has one endemic species, *Culicoidesdokdoensis* Lee & Bae, 2023 and is the only location in Korea, including the mainland, where *Batracomorphusminutus* (Matsumura, 1912) has been observed.

**Figure 6.**
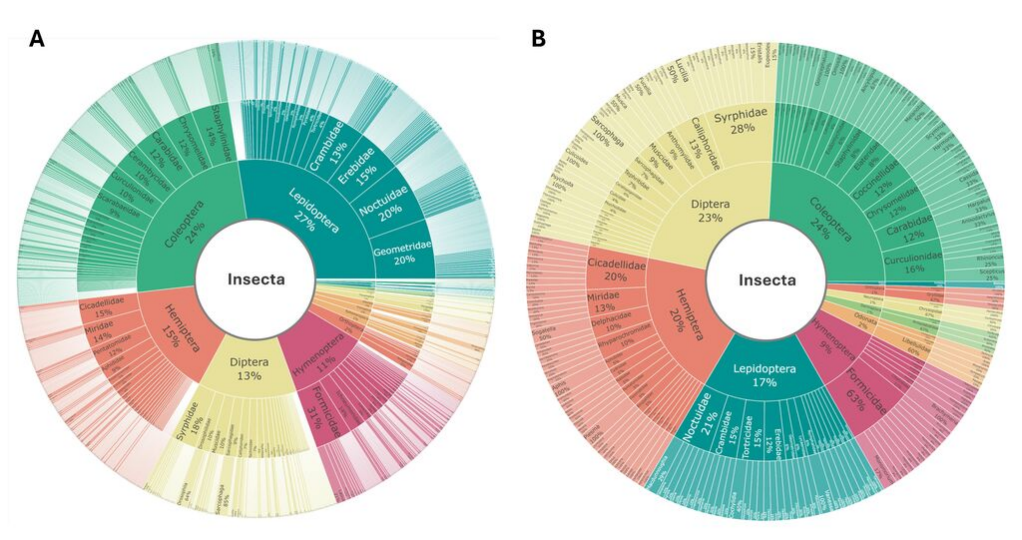
Taxonomic distribution of insect species. **A** Ulleungdo Island; **B** Dokdo Island. The circular charts display the proportional representation of insect orders and their respective families, based on comprehensive species lists compiled for each Island. The percentages are rounded to the nearest whole number.

The top five insect orders on Ulleungdo are, in descending order: Lepidoptera (465 species, 27.46%), Coleoptera (412 species, 24.34%), Hemiptera (259 species, 15.26%), Diptera (227 species, 13.38%) and Hymenoptera (185 species, 10.90%). These top five orders accounted for more than 90% of Ulleungdo's insect diversity, with other orders comprising less than 10% (Fig. 6A). Within the Lepidoptera order, the Geometridae family was the most dominant, representing 20.62% of all Lepidoptera with 92 species, followed by the Noctuidae family with 91 species (19.53%). The Erebidae family comprised 69 species (14.80%), the Crambidae family 59 species (12.66%) and the Tortricidae family 19 species (4.08%). In the Coleoptera order, which accounted for 24.34% of the island's insect diversity, the top five families were Staphylinidae (58 species, 14.04%), Chrysomelidae (49 species, 11.86%), Carabidae (47 species, 11.62%), Cerambycidae (43 species, 10.41%) and Curculionidae (40 species, 9.69%). In the Hemiptera order, the top three families were Cicadellidae (39 species, 15.06%), Miridae (37 species, 14.29%) and Pentatomidae (30 species, 11.58%). The Aphididae and Rhyparochromidae families were fourth and fifth, respectively, with 23 species (8.88%) and 12 species (4.63%). For the Diptera order, the Syrphidae family dominated with 41 species (18.06%), followed by the Muscidae and Drosophilidae families, each with 22 species (9.69%), the Sarcophagidae family with 20 species (8.81%) and the Calliphoridae family with 16 species (7.05%). Lastly, in the Hymenoptera order, which had the smallest proportion amongst the top five, the Formicidae family was dominant, with 57 species (30.81%). The other top families were Ichneumonidae (26 species, 14.05%), Eumenidae (14 species, 7.57%), Apidae (12 species, 6.49%) and Crabronidae (11 species, 5.94%).

The top five insect orders on Dokdo were, in descending order: Coleoptera (49 species, 24.38%), Diptera (45 species, 22.39%), Hemiptera (39 species, 19.40%), Lepidoptera (34 species, 16.92%) and Hymenoptera (19 species, 9.45%). The orders other than the top five accounted for less than 3% of Dokdo's insect diversity (Fig. 6B). Within the Coleoptera order, the Curculionidae family comprised the largest proportion, with eight species (16.33%). The Chrysomelidae, Coccinellidae and Carabidae families each comprised six species, representing 12.24% and together made up the top four families. In the Diptera order, the Syrphidae family dominated with 13 species (28.26%), followed by the Calliphoridae family with six species (13.04%) and both the Anthomyiidae and Muscidae families, each with four species (8.69%), making up the top four families. For Hemiptera, the top four families were Cicadellidae, with eight species (20.00%), Miridae with five species (12.50%) and both the Delphacidae and Rhyparochromidae families each with four species (10.00%). In the Lepidoptera order, the top four families included Noctuidae with seven species (20.59%) and both the Crambidae and Tortricidae families with five species each (14.71%), followed by the Erebidae family with four species (11.76%). In the Hymenoptera order, the Formicidae family significantly dominated with 12 species, accounting for 63.16% of this order, while the other families contained fewer than three species each.

## Discussion

This study conducted a comprehensive historical compilation and field surveys, as well as a re-examination of existing literature and specimens to update the insect fauna databases of Ulleungdo and Dokdo Islands. For Ulleungdo, the species list, last reported by Lim et al. (2013), was updated to include 28 newly-recorded species, bringing the total to 517 species added to the list. For Dokdo, the most recent species list, compiled by Ryu et al. (2021), was updated to include three newly-recorded species, totalling 12 species added to the list. Additionally, re-identifying specimens stored at the Animal Systematics & Taxonomy Laboratory at KNU, including *Saprositesjaponicus* Waterhouse, 1875, led to the removal of several misidentified species from the previously recorded list in the literature. Further, the first records and occurrence records of some species were corrected through historical compilation and additional data verification.

Consequently, this study reported checklists of 1694 species across 20 orders, 227 families and 1165 genera for Ulleungdo, as well as 201 species across 10 orders, 80 families and 173 genera for Dokdo. However, despite the increasing number of species accumulated through ongoing research on Ulleungdo, 18 species have not been reported for over approximately 40 years, indicating a need for further research and surveys on these species (Table 3).

As seen in Fig. 6, although the top five insect orders were the same for Ulleungdo and Dokdo, their distribution percentages differ. On Ulleungdo, the Lepidoptera order was dominant, comprising 27.46% of the total insect taxa, followed by Coleoptera (24.34%), Hemiptera (15.26%), Diptera (13.38%) and Hymenoptera (10.90%). However, on Dokdo, the Coleoptera order was the most prevalent, accounting for 24.38% of the taxa, with other orders following in different proportions: Diptera (22.39%), Hemiptera (19.40%), Lepidoptera (16.92%) and Hymenoptera (9.45%). This shows a distinct pattern difference between the two islands. Additionally, Ulleungdo exhibited a relatively abundant presence of higher taxons compared to Dokdo. While Ulleungdo had 20 orders, Dokdo presented a more restricted diversity with only 10 orders. Thus, Ulleungdo possessed about twice the diversity in insect orders found on Dokdo, highlighting a clear difference in insect diversity between the two Islands. This difference becomes even more pronounced at lower taxonomic levels. Consequently, it is estimated that Ulleungdo has a higher diversity, with 10 more orders, 147 more families, 992 more genera and 1493 more species compared to Dokdo. This higher diversity can also be seen in the number of endemic species: Ulleungdo is home to three endemic species *(Aphisveroniciphaga*, *Cacopsyllaulleungensis*, *Rhomphocalluscoreanus*), whereas Dokdo has only one endemic species (*Culicoidesdokdoensis*). Furthermore, Dokdo displayed characteristics where certain families were disproportionately dominant. For example, the Formicidae family comprised 68% of the Hymenoptera order on Dokdo. This dominance of specific taxonomic groups is presumed to result from the combined effects of Dokdo’s geographical and topographical features, whereby the Island's limited area and isolation impose constraints on insect diversity, while offering ecological advantages to species adapted to specific environments.

**Figure 7.**
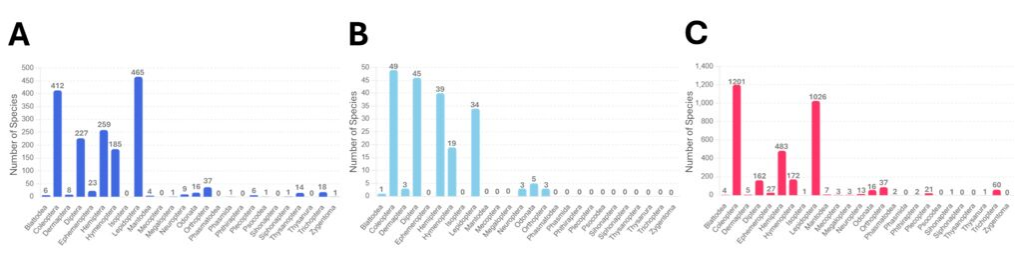
Histogram of insect species diversity by order on three East Sea islands. **A.** Ulleungdo Island; **B.** Dokdo Island; **C.** Oki Island.

Additionally, comparing the insect diversity of Oki Island, which is located in East Sea and the closest island to Dokdo, except for Ulleungdo, with those for Ulleungdo and Dokdo reveals additional insights into taxonomic diversity amongst these Islands (Fig. 7). Oki and Ulleungdo Islands show relatively higher species counts and greater diversity than Dokdo. In contrast, Dokdo exhibits lower taxonomic diversity and fewer species, with a more limited range of orders compared to the other two Islands. Such differences in the diversity of insect taxa between Ulleungdo, Dokdo and Oki are likely due to the different habitats and environmental pressures on the respective islands (Park et al. 2021). Oki and Ulleungdo, with its relatively larger area and more complex terrain, would have provided a broader range of ecological niches to the classes of insects than Dokdo. In contrast, with its relatively small habitat area and characteristics, such as high environmental variability, Dokdo has limited habitat diversity and richness necessary for the localisation and survival of insect species, resulting in lower overall diversity. However, factors such as climate, terrain, location and government policy impact accessibility, meaning surveys on the Island have been highly restricted compared to the other two Islands, which should also be considered.

While this study focuses on the presence and absence of insect taxa across Ulleungdo and Dokdo, it provides a valuable baseline for understanding the insect diversity of these Islands. However, to gain a deeper ecological understanding of the factors driving these diversity patterns, future studies should incorporate comprehensive datasets, including environmental parameters, such as habitat characteristics, climate and geographic distance, alongside advanced monitoring techniques. Such integrative research will be essential to unravel the ecological processes shaping the unique insect communities of these Islands. This study serves as a crucial foundation for future biodiversity research and conservation efforts, contributing to the sustainable management and protection of insect diversity in the East Sea region.

## Figures and Tables

**Table 1.** 2020-2023 Sampling sites and metadata for insect collection on Ulleungdo, Republic of Korea.

**Sampling Site**	**island**	**country**	**stateProvince**	**county**	**locality**	**altitude**	**latitude, longitude**	**sampling method**
Nari Basin 1	Ulleungdo Island	Republic of Korea	Gyeongsangbuk-do	Ulleung-gun	Nari basin, Nari-ri, Bukmyeon	345.36m	37°31'28"N, 130°51'17"E	Pit-fall trap; Molasses trap; Light trap; Sweeping
Nari Basin 2	Ulleungdo Island	Republic of Korea	Gyeongsangbuk-do	Ulleung-gun	Nari basin, Nari-ri, Bukmyeon	369.75m	37°31'22"N, 130°51'14"E	Pit-fall trap; Molasses trap; Light trap; Sweeping
Nari Basin 3	Ulleungdo Island	Republic of Korea	Gyeongsangbuk-do	Ulleung-gun	Nari basin, Nari-ri, Bukmyeon	392.05m	37°31'17"N, 130°51'14"E	Pit-fall trap; Molasses trap; Light trap; Sweeping
Nari Basin 4	Ulleungdo Island	Republic of Korea	Gyeongsangbuk-do	Ulleung-gun	Nari basin, Nari-ri, Bukmyeon	405.86m	37°31'11"N, 130°51'13"E	Pit-fall trap; Molasses trap; Light trap; Sweeping
Nari Basin 5	Ulleungdo Island	Republic of Korea	Gyeongsangbuk-do	Ulleung-gun	Nari basin, Nari-ri, Bukmyeon	453.28m	37°30'59"N, 130°51'15"E	Pit-fall trap; Molasses trap; Light trap; Sweeping
Nari Basin 6	Ulleungdo Island	Republic of Korea	Gyeongsangbuk-do	Ulleung-gun	Nari basin, Nari-ri, Bukmyeon	450.63m	37°30'51"N, 130°51'18"E	Pit-fall trap; Molasses trap; Light trap; Sweeping
Nari Basin 7	Ulleungdo Island	Republic of Korea	Gyeongsangbuk-do	Ulleung-gun	Nari basin, Nari-ri, Bukmyeon	443.92m	37°30'42"N, 130°51'24"E	Pit-fall trap; Molasses trap; Light trap; Sweeping
Nari Basin 8	Ulleungdo Island	Republic of Korea	Gyeongsangbuk-do	Ulleung-gun	Nari basin, Nari-ri, Bukmyeon	448.86m	37°30'40"N, 130°51'45"E	Pit-fall trap; Molasses trap; Light trap; Sweeping
Nari Basin 9	Ulleungdo Island	Republic of Korea	Gyeongsangbuk-do	Ulleung-gun	Nari basin, Nari-ri, Bukmyeon	440.6m	37°30'49"N, 130°51'51"E	Pit-fall trap; Molasses trap; Light trap; Sweeping
Nari Basin 10	Ulleungdo Island	Republic of Korea	Gyeongsangbuk-do	Ulleung-gun	Nari basin, Nari-ri, Bukmyeon	417.12m	37°30'55"N, 130°51'57"E	Pit-fall trap; Molasses trap; Light trap; Sweeping
Nari Basin 11	Ulleungdo Island	Republic of Korea	Gyeongsangbuk-do	Ulleung-gun	Nari basin, Nari-ri, Bukmyeon	398.08m	37°30'58"N, 130°52'03"E	Pit-fall trap; Molasses trap; Light trap; Sweeping
Nari Basin 12	Ulleungdo Island	Republic of Korea	Gyeongsangbuk-do	Ulleung-gun	Nari basin, Nari-ri, Bukmyeon	377.31m	37°31'04"N, 130°52'10"E	Pit-fall trap; Molasses trap; Light trap; Sweeping
Nari Basin 13	Ulleungdo Island	Republic of Korea	Gyeongsangbuk-do	Ulleung-gun	Nari basin, Nari-ri, Bukmyeon	366.69m	37°31'11"N, 130°52'04"E	Pit-fall trap; Molasses trap; Light trap; Sweeping
Nari Basin 14	Ulleungdo Island	Republic of Korea	Gyeongsangbuk-do	Ulleung-gun	Nari basin, Nari-ri, Bukmyeon	392.41m	37°31'12"N, 130°51'55"E	Pit-fall trap; Molasses trap; Light trap; Sweeping
Nari basin 15	Ulleungdo Island	Republic of Korea	Gyeongsangbuk-do	Ulleung-gun	Nari basin, Nari-ri, Bukmyeon	351.08m	37°31'21"N, 130°51'39"E	Pit-fall trap; Molasses trap; Light trap; Sweeping
Seonginbong 1	Ulleungdo Island	Republic of Korea	Gyeongsangbuk-do	Ulleung-gun	Seonginbong, Dodong-ri, Ulleungeup	268.05m	37°29'15"N, 130°53'35"E	Pit-fall trap; Molasses trap; Light trap; Sweeping
Seonginbong 2	Ulleungdo Island	Republic of Korea	Gyeongsangbuk-do	Ulleung-gun	Seonginbong, Dodong-ri, Ulleungeup	314.72m	37°29'20"N, 130°53'33"E	Pit-fall trap; Molasses trap; Light trap; Sweeping
Seonginbong 3	Ulleungdo Island	Republic of Korea	Gyeongsangbuk-do	Ulleung-gun	Seonginbong, Dodong-ri, Ulleungeup	378.62m	37°29'20"N, 130°53'28"E	Pit-fall trap; Molasses trap; Light trap; Sweeping
Seonginbong 4	Ulleungdo Island	Republic of Korea	Gyeongsangbuk-do	Ulleung-gun	Seonginbong, Dodong-ri, Ulleungeup	424.49m	37°29'22"N, 130°53'26"E	Pit-fall trap; Molasses trap; Light trap; Sweeping
Seonginbong 5	Ulleungdo Island	Republic of Korea	Gyeongsangbuk-do	Ulleung-gun	Seonginbong, Dodong-ri, Ulleungeup	462.46m	37°29'25"N, 130°53'23"E	Pit-fall trap; Molasses trap; Light trap; Sweeping
Seonginbong 6	Ulleungdo Island	Republic of Korea	Gyeongsangbuk-do	Ulleung-gun	Seonginbong, Dodong-ri, Ulleungeup	503.46m	37°29'27"N, 130°53'18"E	Pit-fall trap; Molasses trap; Light trap; Sweeping
Seonginbong 7	Ulleungdo Island	Republic of Korea	Gyeongsangbuk-do	Ulleung-gun	Seonginbong, Dodong-ri, Ulleungeup	556.48m	37°29'28"N, 130°53'12"E	Pit-fall trap; Molasses trap; Light trap; Sweeping
Seonginbong 8	Ulleungdo Island	Republic of Korea	Gyeongsangbuk-do	Ulleung-gun	Seonginbong, Dodong-ri, Ulleungeup	600.74m	37°29'26"N, 130°53'08"E	Pit-fall trap; Molasses trap; Light trap; Sweeping
Seonginbong 9	Ulleungdo Island	Republic of Korea	Gyeongsangbuk-do	Ulleung-gun	Seonginbong, Dodong-ri, Ulleungeup	653.06m	37°29'27"N, 130°53'04"E	Pit-fall trap; Molasses trap; Light trap; Sweeping
Seonginbong 10	Ulleungdo Island	Republic of Korea	Gyeongsangbuk-do	Ulleung-gun	Seonginbong, Dodong-ri, Ulleungeup	721.38m	37°29'36"N, 130°52'45"E	Pit-fall trap; Molasses trap; Light trap; Sweeping
Seonginbong 11	Ulleungdo Island	Republic of Korea	Gyeongsangbuk-do	Ulleung-gun	Seonginbong, Dodong-ri, Ulleungeup	773.72m	37°29'37"N, 130°52'38"E	Pit-fall trap; Molasses trap; Light trap; Sweeping
Seonginbong 12	Ulleungdo Island	Republic of Korea	Gyeongsangbuk-do	Ulleung-gun	Seonginbong, Dodong-ri, Ulleungeup	846.15m	37°29'44"N, 130°52'18"E	Pit-fall trap; Molasses trap; Light trap; Sweeping
Seonginbong 13	Ulleungdo Island	Republic of Korea	Gyeongsangbuk-do	Ulleung-gun	Seonginbong, Dodong-ri, Ulleungeup	878.95m	37°29'47"N, 130°52'12"E	Pit-fall trap; Molasses trap; Light trap; Sweeping
Seonginbong 14	Ulleungdo Island	Republic of Korea	Gyeongsangbuk-do	Ulleung-gun	Seonginbong, Dodong-ri, Ulleungeup	911.02m	37°29'46"N, 130°52'02"E	Pit-fall trap; Molasses trap; Light trap; Sweeping
Seonginbong 15	Ulleungdo Island	Republic of Korea	Gyeongsangbuk-do	Ulleung-gun	Seonginbong, Dodong-ri, Ulleungeup	935.4m	37°29'41"N, 130°51'54"E	Pit-fall trap; Molasses trap; Light trap; Sweeping
Taeharyeong 1	Ulleungdo Island	Republic of Korea	Gyeongsangbuk-do	Ulleung-gun	Taeharyeong, Namseo-ri, Seomyeon	121.98m	37°30'26"N, 130°49'03"E	Pit-fall trap; Molasses trap; Light trap; Sweeping
Taeharyeong 2	Ulleungdo Island	Republic of Korea	Gyeongsangbuk-do	Ulleung-gun	Taeharyeong, Namseo-ri, Seomyeon	164.99m	37°30'21"N, 130°49'05"E	Pit-fall trap; Molasses trap; Light trap; Sweeping
Taeharyeong 3	Ulleungdo Island	Republic of Korea	Gyeongsangbuk-do	Ulleung-gun	Taeharyeong, Namseo-ri, Seomyeon	185.36m	37°30'14"N, 130°49'11"E	Pit-fall trap; Molasses trap; Light trap; Sweeping
Taeharyeong 4	Ulleungdo Island	Republic of Korea	Gyeongsangbuk-do	Ulleung-gun	Taeharyeong, Namseo-ri, Seomyeon	213.06m	37°30'08"N, 130°49'10"E	Pit-fall trap; Molasses trap; Light trap; Sweeping
Taeharyeong 5	Ulleungdo Island	Republic of Korea	Gyeongsangbuk-do	Ulleung-gun	Taeharyeong, Namseo-ri, Seomyeon	225.53m	37°30'06"N, 130°49'16"E	Pit-fall trap; Molasses trap; Light trap; Sweeping
Taeharyeong 6	Ulleungdo Island	Republic of Korea	Gyeongsangbuk-do	Ulleung-gun	Taeharyeong, Namseo-ri, Seomyeon	258.14m	37°30'02"N, 130°49'18"E	Pit-fall trap; Molasses trap; Light trap; Sweeping
Taeharyeong 7	Ulleungdo Island	Republic of Korea	Gyeongsangbuk-do	Ulleung-gun	Taeharyeong, Namseo-ri, Seomyeon	307.18m	37°29'58"N, 130°49'25"E	Pit-fall trap; Molasses trap; Light trap; Sweeping
Taeharyeong 8	Ulleungdo Island	Republic of Korea	Gyeongsangbuk-do	Ulleung-gun	Taeharyeong, Namseo-ri, Seomyeon	291.64m	37°29'55"N, 130°49'23"E	Pit-fall trap; Molasses trap; Light trap; Sweeping
Taeharyeong 9	Ulleungdo Island	Republic of Korea	Gyeongsangbuk-do	Ulleung-gun	Taeharyeong, Namseo-ri, Seomyeon	322.8m	37°29'53"N, 130°49'27"E	Pit-fall trap; Molasses trap; Light trap; Sweeping
Taeharyeong 10	Ulleungdo Island	Republic of Korea	Gyeongsangbuk-do	Ulleung-gun	Taeharyeong, Namseo-ri, Seomyeon	348.2m	37°29'48"N, 130°49'28"E	Pit-fall trap; Molasses trap; Light trap; Sweeping
Taeharyeong 11	Ulleungdo Island	Republic of Korea	Gyeongsangbuk-do	Ulleung-gun	Taeharyeong, Namseo-ri, Seomyeon	371.73m	37°29'45"N, 130°49'36"E	Pit-fall trap; Molasses trap; Light trap; Sweeping
Taeharyeong 12	Ulleungdo Island	Republic of Korea	Gyeongsangbuk-do	Ulleung-gun	Taeharyeong, Namseo-ri, Seomyeon	377.34m	37°29'43"N, 130°49'33"E	Pit-fall trap; Molasses trap; Light trap; Sweeping
Taeharyeong 13	Ulleungdo Island	Republic of Korea	Gyeongsangbuk-do	Ulleung-gun	Taeharyeong, Namseo-ri, Seomyeon	397.75m	37°29'41"N, 130°49'36"E	Pit-fall trap; Molasses trap; Light trap; Sweeping
Taeharyeong 14	Ulleungdo Island	Republic of Korea	Gyeongsangbuk-do	Ulleung-gun	Taeharyeong, Namseo-ri, Seomyeon	419.1m	37°29'39"N, 130°49'40"E	Pit-fall trap; Molasses trap; Light trap; Sweeping
Taeharyeong 15	Ulleungdo Island	Republic of Korea	Gyeongsangbuk-do	Ulleung-gun	Taeharyeong, Namseo-ri, Seomyeon	445.65m	37°29'35"N, 130°49'39"E	Pit-fall trap; Molasses trap; Light trap; Sweeping
Dodong 1	Ulleungdo Island	Republic of Korea	Gyeongsangbuk-do	Ulleung-gun	196-1, Dodong-ri, Ulleungeup	43.99m	37°29'04"N, 130°54'18"E	BGsentinel trap (Biogents, Regensburg, Germany); Black-light trap
Dodong 2	Ulleungdo Island	Republic of Korea	Gyeongsangbuk-do	Ulleung-gun	196-2, Dodong-ri, Ulleungeup	16.86m	37°28'56"N, 130°54'33"E	BGsentinel trap (Biogents, Regensburg, Germany); Black-light trap

**Table 2.** 2020-2023 Sampling sites and metadata for insect collection on Dokdo, Republic of Korea.

**Sampling Site**	**island**	**country**	**stateProvince**	**county**	**locality**	**altitude**	**latitude, longitude**	**sampling method**
Dongdo 1	Dokdo Island	Republic of Korea	Gyeongsangbuk-do	Ulleung-gun	Dongdo of Dokdo, Dokdo-ri, Ulleungeup	10.55m	37°14'21"N, 131°52'06"E	Sweeping
Dongdo 2	Dokdo Island	Republic of Korea	Gyeongsangbuk-do	Ulleung-gun	Dongdo of Dokdo, Dokdo-ri, Ulleungeup	11.44m	37°14'21"N, 131°52'06"E	Sweeping
Dongdo 3	Dokdo Island	Republic of Korea	Gyeongsangbuk-do	Ulleung-gun	Dongdo of Dokdo, Dokdo-ri, Ulleungeup	14.84m	37°14'21"N, 131°52'07"E	Sweeping
Dongdo 4	Dokdo Island	Republic of Korea	Gyeongsangbuk-do	Ulleung-gun	Dongdo of Dokdo, Dokdo-ri, Ulleungeup	16.92m	37°14'21"N, 131°52'08"E	Sweeping
Dongdo 5	Dokdo Island	Republic of Korea	Gyeongsangbuk-do	Ulleung-gun	Dongdo of Dokdo, Dokdo-ri, Ulleungeup	18.77m	37°14'21"N, 131°52'09"E	Sweeping
Dongdo 6	Dokdo Island	Republic of Korea	Gyeongsangbuk-do	Ulleung-gun	Dongdo of Dokdo, Dokdo-ri, Ulleungeup	22.96m	37°14'22"N, 131°52'10"E	Sweeping
Dongdo 7	Dokdo Island	Republic of Korea	Gyeongsangbuk-do	Ulleung-gun	Dongdo of Dokdo, Dokdo-ri, Ulleungeup	25.18m	37°14'22"N, 131°52'10"E	Sweeping
Dongdo 8	Dokdo Island	Republic of Korea	Gyeongsangbuk-do	Ulleung-gun	Dongdo of Dokdo, Dokdo-ri, Ulleungeup	25m	37°14'23"N, 131°52'11"E	Sweeping
Dongdo 9	Dokdo Island	Republic of Korea	Gyeongsangbuk-do	Ulleung-gun	Dongdo of Dokdo, Dokdo-ri, Ulleungeup	25.94m	37°14'24"N, 131°52'13"E	Sweeping
Dongdo 10	Dokdo Island	Republic of Korea	Gyeongsangbuk-do	Ulleung-gun	Dongdo of Dokdo, Dokdo-ri, Ulleungeup	15.64m	37°14'25"N, 131°52'13"E	Sweeping
Seodo 1	Dokdo Island	Republic of Korea	Gyeongsangbuk-do	Ulleung-gun	Seodo of Dokdo, Dokdo-ri, Ulleungeup	22.4m	37°14'27"N, 131°51'55"E	Sweeping
Seodo 2	Dokdo Island	Republic of Korea	Gyeongsangbuk-do	Ulleung-gun	Seodo of Dokdo, Dokdo-ri, Ulleungeup	21.56m	37°14'26"N, 131°51'54"E	Sweeping
Seodo 3	Dokdo Island	Republic of Korea	Gyeongsangbuk-do	Ulleung-gun	Seodo of Dokdo, Dokdo-ri, Ulleungeup	25.31m	37°14'27"N, 131°51'53"E	Sweeping
Seodo 4	Dokdo Island	Republic of Korea	Gyeongsangbuk-do	Ulleung-gun	Seodo of Dokdo, Dokdo-ri, Ulleungeup	35.8m	37°14'28"N, 131°51'52"E	Sweeping
Seodo 5	Dokdo Island	Republic of Korea	Gyeongsangbuk-do	Ulleung-gun	Seodo of Dokdo, Dokdo-ri, Ulleungeup	25.11m	37°14'28"N, 131°51'50"E	Sweeping
Seodo 6	Dokdo Island	Republic of Korea	Gyeongsangbuk-do	Ulleung-gun	Seodo of Dokdo, Dokdo-ri, Ulleungeup	23.86m	37°14'30"N, 131°51'50"E	Sweeping
Seodo 7	Dokdo Island	Republic of Korea	Gyeongsangbuk-do	Ulleung-gun	Seodo of Dokdo, Dokdo-ri, Ulleungeup	20.95m	37°14'31"N, 131°51'50"E	Sweeping
Seodo 8	Dokdo Island	Republic of Korea	Gyeongsangbuk-do	Ulleung-gun	Seodo of Dokdo, Dokdo-ri, Ulleungeup	16.87m	37°14'31"N, 131°51'49"E	Sweeping
Seodo 9	Dokdo Island	Republic of Korea	Gyeongsangbuk-do	Ulleung-gun	Seodo of Dokdo, Dokdo-ri, Ulleungeup	17m	37°14'30"N, 131°51'49"E	Sweeping
Seodo 10	Dokdo Island	Republic of Korea	Gyeongsangbuk-do	Ulleung-gun	Seodo of Dokdo, Dokdo-ri, Ulleungeup	12.19m	37°14'31"N, 131°51'48"E	Sweeping

**Table 3.** List of insect species that have not been reported in Ulleungdo Is. within approximately 40 years.

	**Last observation year**	**Reference**	**Species**
1	1934	Kim (2012)	*Onthophagusviduus* Harold, 1875
2	1955	Cho (1955)	*Horridipameranietneri* (Dohrn, 1860)
3	1965	Cho (1965)	*Silphaperforata* Gebler, 1832
4	1971	Kim (1971)	*Ammophilavagabunda* Smith, 1856
5	1971	Kim (1971)	*Ostriniascapulalis* (Walker, 1859)
6	1977	Hong et al. (2011)	*Pellobarismelancholica* (Roelofs, 1875)
7	1978	Suh (2019)	*Anthomyiaplumiseta* Stein, 1918
8	1981	Suh (2019)	*Pegomyacunicularia* (Rondani, 1866)
9	1981	Park et al. (2012)	*Tropideresnaevulus* Faust, 1887
10	1981	Hong et al. (2012)	*Orchesteslateritius* (Morimoto, 1984)
11	1981	Hong et al. (2012)	*Orchestesharunire* (Morimoto, 1984)
12	1981	Hong et al. 2012	*Orchesteshorii* (Kôno, 1937)
13	1981	Kim (2012)	*Agrilinusuniformis* (Waterhouse, 1875)
14	1981	Hong et al. (2011)	*Rhinoncussibiricus* Faust, 1893
15	1981	Lee and Kwon (1981)	*Proboscidocorismalayus* Reuter, 1908
16	1985	Kim (2012)	*Bodilopsissordidus* (Fabricius, 1775)
17	1985	Kim (2012)	*Planolinoidesborealis* (Gyllenhal, 1827)
18	1985	Kim (2012)	*Colobopterusquadratus* (Reiche, 1850)
